# Comprehensive analysis of angiogenesis pattern and related immune landscape for individual treatment in osteosarcoma

**DOI:** 10.1038/s41698-023-00415-7

**Published:** 2023-06-29

**Authors:** Zhuangyao Liao, Ming Li, Guoming Wen, Kun Wang, Dengbo Yao, Enming Chen, Yuwei Liang, Tong Xing, Kaihui Su, Changchun Liang, Zhen Che, Qing Ning, Jun Tang, Wenbin Yan, Yuxi Li, Lin Huang

**Affiliations:** 1 Department of Orthopedics, Sun Yat-Sen Memorial Hospital, Sun Yat-Sen University, Guangzhou, China; 2 Guangdong Provincial Key Laboratory of Malignant Tumor Epigenetics and Gene Regulation, Sun Yat-sen Memorial Hospital, Sun Yat-sen University, Guangzhou, China; 3 Department of Orthopedics, The Eighth Affiliated Hospital, Sun Yat-Sen University, Shenzhen, China

**Keywords:** Tumour biomarkers, Tumour angiogenesis, Bone cancer, Tumour immunology

## Abstract

Postoperative recurrence and metastasis are the main reasons for the poor prognosis of osteosarcoma (OS). Currently, an ideal predictor for not only prognosis but also drug sensitivity and immunotherapy responses in OS patients is urgently needed. Angiogenesis plays a crucial role in tumour progression, which suggests its immense potential for predicting prognosis and responses to immunotherapy for OS. Angiogenesis patterns in OS were explored in depth in this study to construct a prognostic model called ANGscore and clarify the underlying mechanism involved in the immune microenvironment. The efficacy and robustness of the model were validated in multiple datasets, including bulk RNA-seq datasets (TARGET-OS, GSE21257), a single-cell RNA-seq dataset (GSE152048) and immunotherapy-related datasets (GSE91061, GSE173839). OS patients with a high ANGscore had a worse prognosis, accompanied by the immune desert phenotype. Pseudotime and cellular communication analyses in scRNA-seq data revealed that as the ANGscore increased, the malignant degree of cells increased, and IFN-γ signalling was involved in tumour progression and regulation of the tumour immune microenvironment. Furthermore, the ANGscore was associated with immune cell infiltration and the response rate to immunotherapy. OS patients with high ANGscore might be resistant to uprosertib, and be sensitive to VE821, AZD6738 and BMS.345541. In conclusion, we established a novel ANGscore system by comprehensively analysing the expression pattern of angiogenesis genes, which can accurately differentiate the prognosis and immune characteristics of OS populations. Additionally, the ANGscore can be used for patient stratification during immunotherapy, and guide individualized treatment strategies.

## Introduction

Osteosarcoma (OS), a malignant tumour predominantly occurring in children and adolescents, has the characteristics of low morbidity but high mortality^1^. Primary osteosarcoma mainly arises in the long bones of the extremities, with a high rate of metastasis, mostly to the lung^2,3^. Currently, combined treatments including surgery, radiotherapy and chemotherapy result in an overall 5-year survival rate of 60–70% for patients with nonmetastatic osteosarcoma. However, the 5-year survival rate of patients with osteosarcoma recurrence and metastases after initial treatment is only 16%^4,5^. Owing to the heterogeneous causes and complex tumour microenvironment (TME) of osteosarcoma, traditional treatment is prone to drug resistance. Therefore, a novel predictor is urgently needed to stratify OS patients and develop individualized treatment plans.

Due to the growing and proliferating demand of the tumour, osteosarcoma needs to generate new blood vessels to obtain more nutrients. Angiogenesis has been reported as a typical characteristic during OS progression, including proliferation, migration and metastasis^6–8^. As an important regulator of angiogenesis, the vascular endothelial growth factor (VEGF) pathway is activated by multiple inducers in osteosarcoma, such as WISP1^9^, CCL3^10^ and CCL5^11^, suggesting abnormal activation of angiogenesis in OS. Based on the tight relationship between angiogenesis and OS, antiangiogenic drugs have been considered as potential treatments for OS patients. Several clinical trials of anti-vascular agents have been conducted in osteosarcoma populations with promising results^5,12,13^. However, the process of angiogenesis is regulated by multiple factors, including a variety of immune cells and cytokines^14^, and a more accurate classification of OS patients is conducive to the selection of a more appropriate anti-vascular treatment plan or a treatment plan combined with other drugs.

The tumour immune microenvironment (TIME) refers to the interaction environment formed by malignant cells in the tumour and various surrounding immune cells. The mutual regulatory relationship between angiogenesis and the TIME has been described in many studies. Regulatory T cells (Tregs) can directly release the proangiogenic factors VEGF and basic fibroblast growth factor (bFGF), or secrete specific cytokines, which indirectly induce the production of VEGF and bFGF to promote tumour angiogenesis^15^. Natural killer (NK) cells are also essential for the induction of VEGF expression under hypoxic conditions. Previous studies have demonstrated that some decidual NK cells (dNK) can induce the formation of capillary-like structures in non-small cell lung cancer, colorectal cancer, etc^16–18^. Furthermore, abnormal dilatation of tumour blood vessels can upregulate the expression of chemokines to accelerate the recruitment of Tregs into tumours^19,20^. Circulating VEGF impedes dendritic cell (DC) maturation and function, allowing tumour cells to escape immune surveillance^21^. Hence, assessment of angiogenesis-associated profiles in OS patients also helps to evaluate the TIME, which may contribute to improving the efficiency of both antiangiogenic treatment and immunotherapy.

In the present study, we integrated expression profiles from public datasets for a comprehensive evaluation of angiogenesis characteristics in OS. The angiogenesis signature exhibited an overall phenomenon closely related to immunity, including the infiltration of immune cells, the cancer immunity cycle and the expression of immune checkpoints. Based on the angiogenesis signature, we developed an angiogenesis scoring system called the ANGscore to distinguish overall survival (OS) and the tumour immune microenvironment, which were validated in conventional bulk RNA-seq datasets, single-cell RNA sequencing (scRNA-seq) dataset and immunotherapy-related datasets. Our findings suggest that the ANGscore is a stable and efficient predictor to characterize the angiogenesis state, prognosis, TIME, likelihood of metastasis development and the response to immunotherapy in OS.

## Results

### Study workflow

**Fig. 1:**
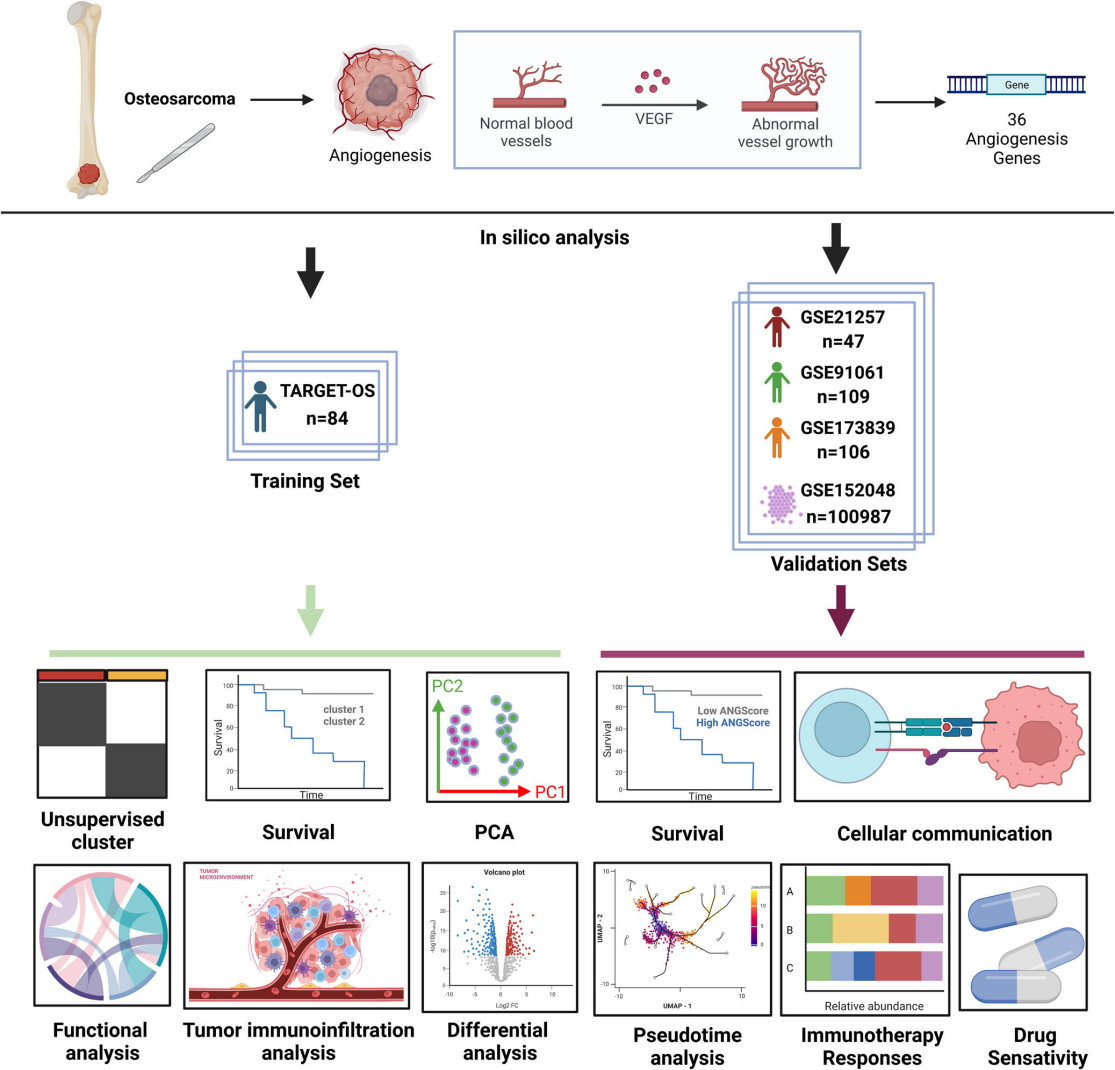
Flowchart of this study. Flowchart for comprehensive analysis of angiogenesis patterns in patients with osteosarcoma (OS). (Created with Biorender.com).

For the training and validation cohorts, we included 84 patients from the TARGET-OS database, 47 patients from GSE21257 for the RNA-seq data, and 11 patients from GSE152048 for the single cell RNA-seq data. A total of 109 patients from GSE91061 and 106 patients from GSE173839 were included to validate the predictive efficacy of the immunotherapy response rate. For scRNA-seq data, we performed quality control and normalization, and 100987 cells were included in subsequent analyses. In addition, 36 angiogenesis genes were included in the analysis (Supplementary Table 1). The flow diagram of this study is shown in Fig. 1.

### Identification of angiogenesis related patterns in OS

The overall correlation and interaction of angiogenesis genes in the TARGET-OS database are shown in Supplementary Figs. 1, 2.

**Fig. 2:**
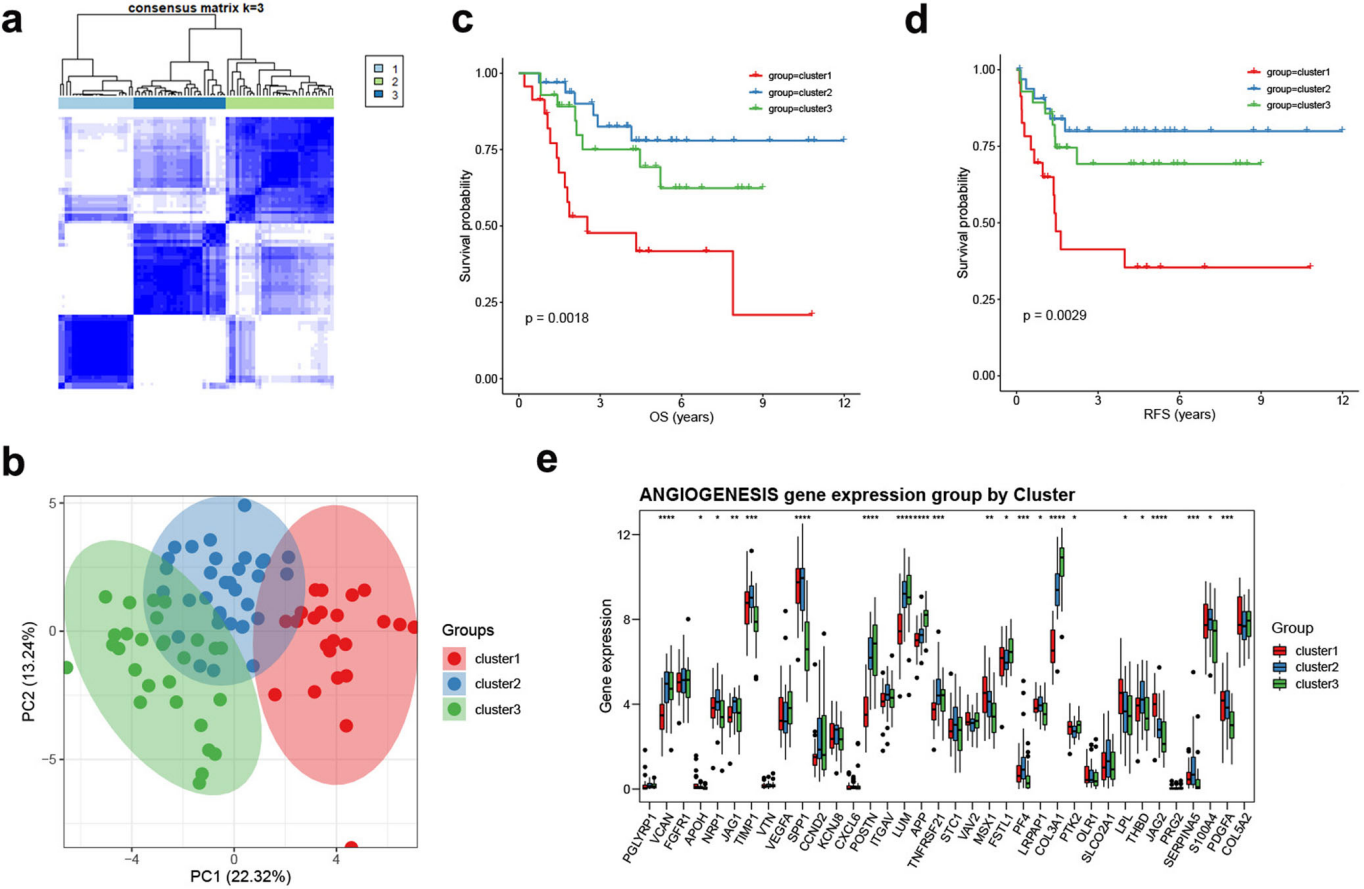
Angiogenesis patterns in the TARGET-OS cohort. **a** Consensus clustering matrix at *K* = 2. **b** PCA analysis of three angiogenesis clusters. **c** Survival analysis of overall survival (OS) for patients with three clusters using Kaplan–Meier curves. **d** Survival analysis of relapse free survival (RFS) for patients with three clusters using Kaplan–Meier curves. **e** The expression of angiogenesis genes among three angiogenesis clusters. The asterisks represent the statistical *P* value (*p < 0.05; **p < 0.01; ***p < 0.001; ****p < 0.0001).

Based on consensus clustering of the expression of the 36 angiogenesis genes, we identified three different angiogenesis patterns, namely, Cluster 1, Cluster 2 and Cluster 3 (Fig. 2a). PCA analysis confirmed the cluster division (Fig. 2b). Survival curves exhibited that Cluster 2 showed significantly improved overall survival and recurrence-free survival (Fig. 2c, d), and the expression of angiogenesis genes among the three clusters had overall significant differences (Fig. 2e).

**Fig. 3:**
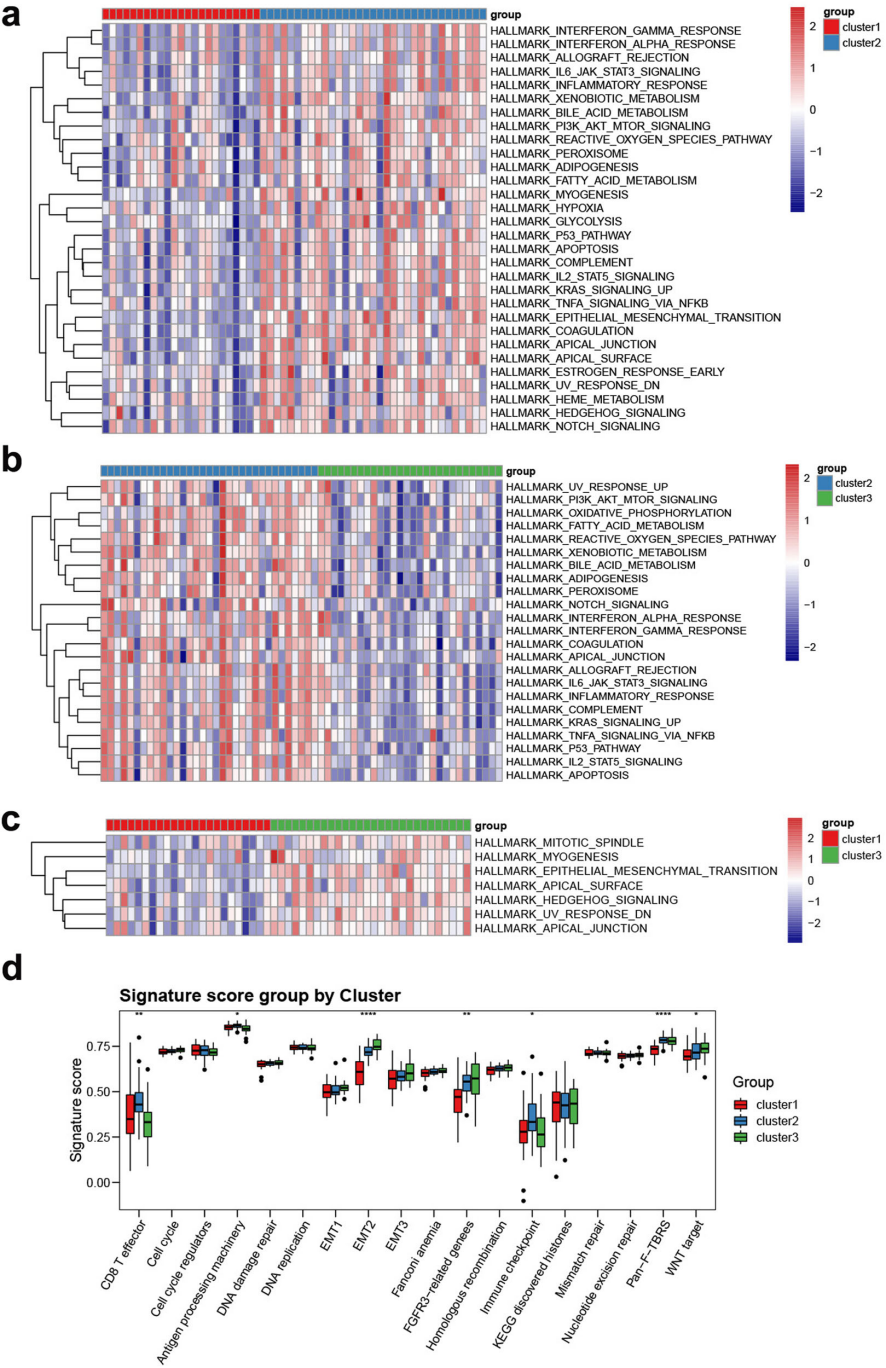
Biological characteristics of angiogenesis pattern in the TARGET-OS cohort. **a**–**c** Heatmaps showing differentially enriched hallmark pathways among pairs of the three angiogenesis patterns by GSVA. **d** GSVA analysis representing the significance of differential enrichment of common cancer-related signatures among the three angiogenesis patterns.

To explore the underlying biological functions between three distinct angiogenesis patterns, we conducted GSVA with the MSigDB hallmark gene sets. The results indicated that Cluster 2 was enriched in circuit-activated immune infiltration-related functions, including allograft rejection, inflammatory response, IL2/STAT5 signalling and IL6/JAK/STAT3 signalling (Fig. 3a, b). In addition, oxidative phosphorylation, the reactive oxygen species pathway and fatty acid metabolism, which represent biological oxidation processes, were also enriched. Previous studies reported that activated T-cell mitochondrial metabolism and oxidative phosphorylation could improve antitumour performance^22^, which suggest that Cluster 2 patients may be in a state of compound immunity. Moreover, Cluster 1 and Cluster 3 showed prominent associations with biological processes related to immune suppression (Fig. 3a–c).

We then performed ssGSEA algorithm to detect the common cancer related signature in the three clusters (Supplementary Table 2). We found that Cluster 2 OS patients had higher enrichment scores in CD8 T-cell effectors, antigen processing machinery and immune checkpoints, confirming our conjecture (Fig. 3d).

### Differences in the TME for the three angiogenesis patterns

**Fig. 4:**
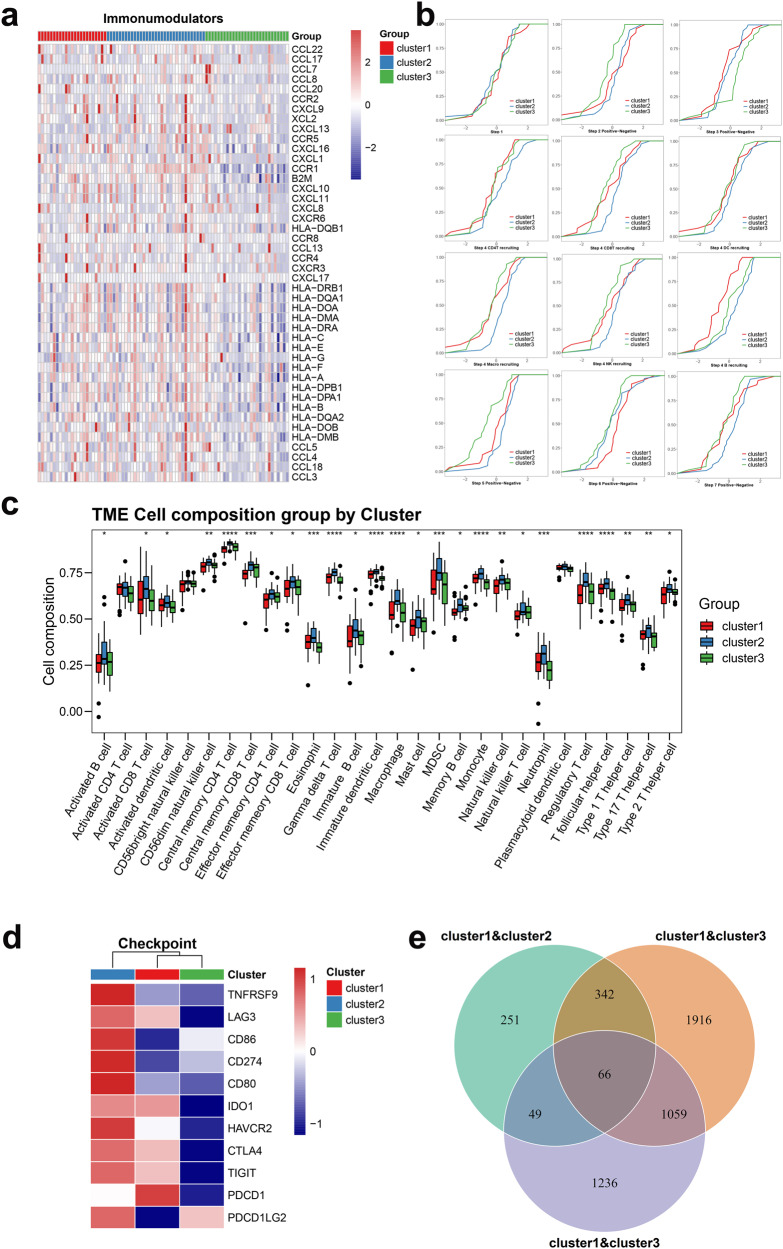
TME and immune characteristics of the three angiogenesis patterns in the TARGET-OS cohort. **a** Heatmap showing differential expression of chemokines, receptors and MHC molecules among the three angiogenesis patterns. **b** Differences in seven steps of the cancer immunity cycle among three angiogenesis patterns. **c** The abundance of TME components in three angiogenesis patterns. **d** Heatmap showing the expression of immune checkpoint genes in the three angiogenesis patterns. **e** Venn diagram showing common DEGs for the three angiogenesis patterns.

To explore the immunological characteristics of the three distinct patterns, we estimated the abundance of immune components, including immunomodulators, immune checkpoints, immune cell infiltration in the TME and activation of the cancer immunity cycle. We found that MHC-I and MHC-II components, involved in antigen presentation and processing, such as HLA-A, HLA-B, HLA-DRB1 and HLA-DMB, were upregulated in Cluster 2. In addition, the chemokines and their specific receptors, promoting the recruitment of NK cells and CD8+ T cells, including CCL4, CCL13, CCR1, CXCR3, CXCL9 and CXCL10, were also upregulated in this group (Fig. 4a), which indicated the activated immune microenvironment in Cluster 2.

Due to the complex chemokine-receptor network of dynamic interactions, there are limitations in solely explaining the process by individual chemokine expression. Therefore, we comprehensively elucidated the cancer immune characteristics of different populations by calculating the cancer immune cycle to quantify the degree of activation of each step. The results showed that most of the steps were highly activated in Cluster 2, especially cancer antigen presentation (Step 2), immune cell recruitment such as CD4+ T cell, CD8+ T cell, dendritic cell, macrophage, NK cell and B-cell recruiting (Step 4), infiltration of immune cells into tumours (Step 5) and killing of cancer cells (Step 7). It is worth noting that Cluster 1 showed significant upregulation in the recognition of cancer cells by T cells (Step 6), suggesting its dysregulation of other immune process, such as decreased recruitment of immune cells in the early stage, the reduction in effector cytotoxic T cells and decreased abundance of immune cell infiltration, etc. The overall activities of Cluster 3 showed a significant downregulation in Steps 2, 4 and 5, which may contribute to its immune suppression phenotype (Fig. 4b). Thereafter, we used ssGSEA to qualify the infiltration of 28 immune cells in the TME. Activated CD8 T cells, natural killer cells and natural killer T cells were abundant in Cluster 2, confirming its enhanced antitumour status. Compared with Cluster 3, Cluster 1 had a lower enrichment in natural killer cells and natural killer T cells (Fig. 4c), consistent with its worsening prognosis. We also compared the expression of the common immune checkpoints, and Cluster 2 had higher overall expression of CD274 (PD-L1), PDCD1LG2 (PD-L2), CTLA4, LAG3, HAVCR2 (TIM-3), IDO1 and TIGIT (Fig. 4d).

Taken together, the above results suggest that the three angiogenesis patterns had distinctive differences in the cancer immunity cycle and immune cell infiltration in the TME, especially the immune-inflamed phenotype in Cluster 2.

### Classification of OS subtypes by angiogenesis-related gene patterns

**Fig. 5:**
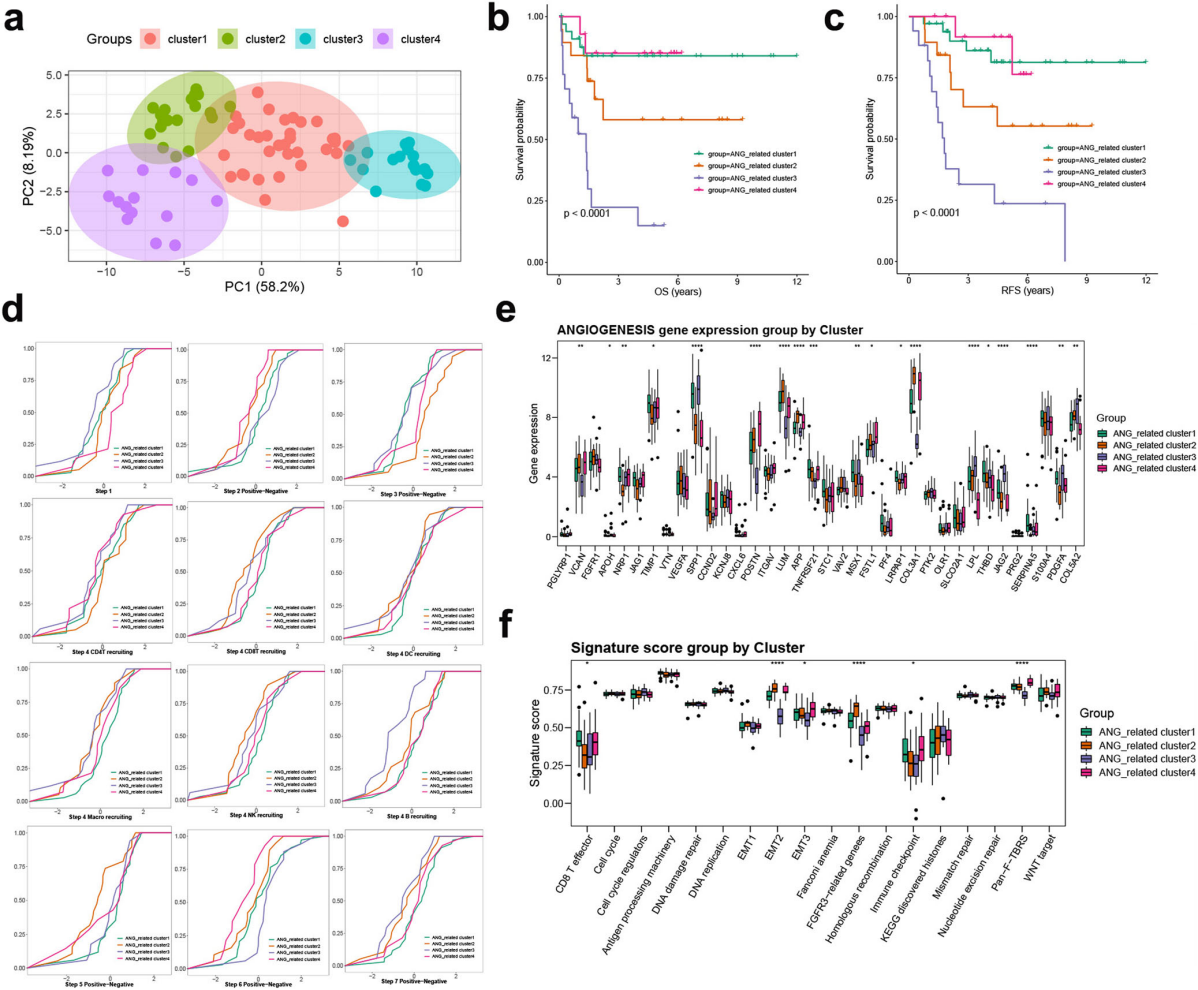
Classification of OS clusters based on angiogenesis signatures in the TARGET-OS cohort. **a** PCA analysis of three angiogenesis-related clusters based on the expression of the 32 angiogenesis-related signature genes. **b** Survival analysis of overall survival (OS) for patients with four angiogenesis-related clusters using Kaplan–Meier curves. **c** Survival analysis of relapse free survival (RFS) for patients with four angiogenesis-related clusters using Kaplan–Meier curves. **d** Differences in seven steps of the cancer immunity cycle among four angiogenesis-related clusters. **e** The expression of angiogenesis genes among four angiogenesis-related clusters. **f** GSVA analysis representing the significance of differential enrichment of common cancer-related signatures among the four angiogenesis-related clusters.

To explore the underlying genes modulated by angiogenesis genes, we conducted differential analyses between each pair of clusters of the three angiogenesis patterns. We identified 66 common DEGs (Fig. 4e), and the univariate Cox regression results showed that 32 candidates were significantly prognostic (Supplementary Table 3). Based on the above 32 angiogenesis-related genes, consensus clustering was used to divide OS patients in the TARGET-OS database into four subtypes, namely, angiogenesis-related gene Clusters 1-4 (Fig. 5a). The overall survival and recurrence-free survival of angiogenesis-related (ANG-related) Cluster 1 and Cluster 4 were better than those of the other two clusters (Fig. 5b, c). In addition, a majority of the cancer immune cycle was activated in ANG-related Cluster 1 and Cluster 4, especially in CD8+ T cell, macrophage, NK cell and B-cell recruitment (Fig. 5d). We found that the expression of some genes in ANG-related Cluster 1 and Cluster 4 was different from the other two groups, such as TIMP1, COL5A2, etc (Fig. 5e). Previous research has reported that TIMP1 is involved in the mechanism of anti-angiogenesis in patients with diabetes mellitus^23^, and the function of other differential expression genes may collaboratively suggest that these two populations are in a negative state of angiogenesis^24^. Consistent with angiogenesis Cluster 2, we also identified ANG-related Cluster 1 and Cluster 4 as having higher enrichment scores for CD8+ T effectors and immune checkpoints (Fig. 5f). These findings suggested that the angiogenesis related gene signature showed a good classification effect and the ANG-related Cluster 1 and Cluster 4 were associated with immune activation in the TME.

### ANGscore system can predict OS patient outcome

**Fig. 6:**
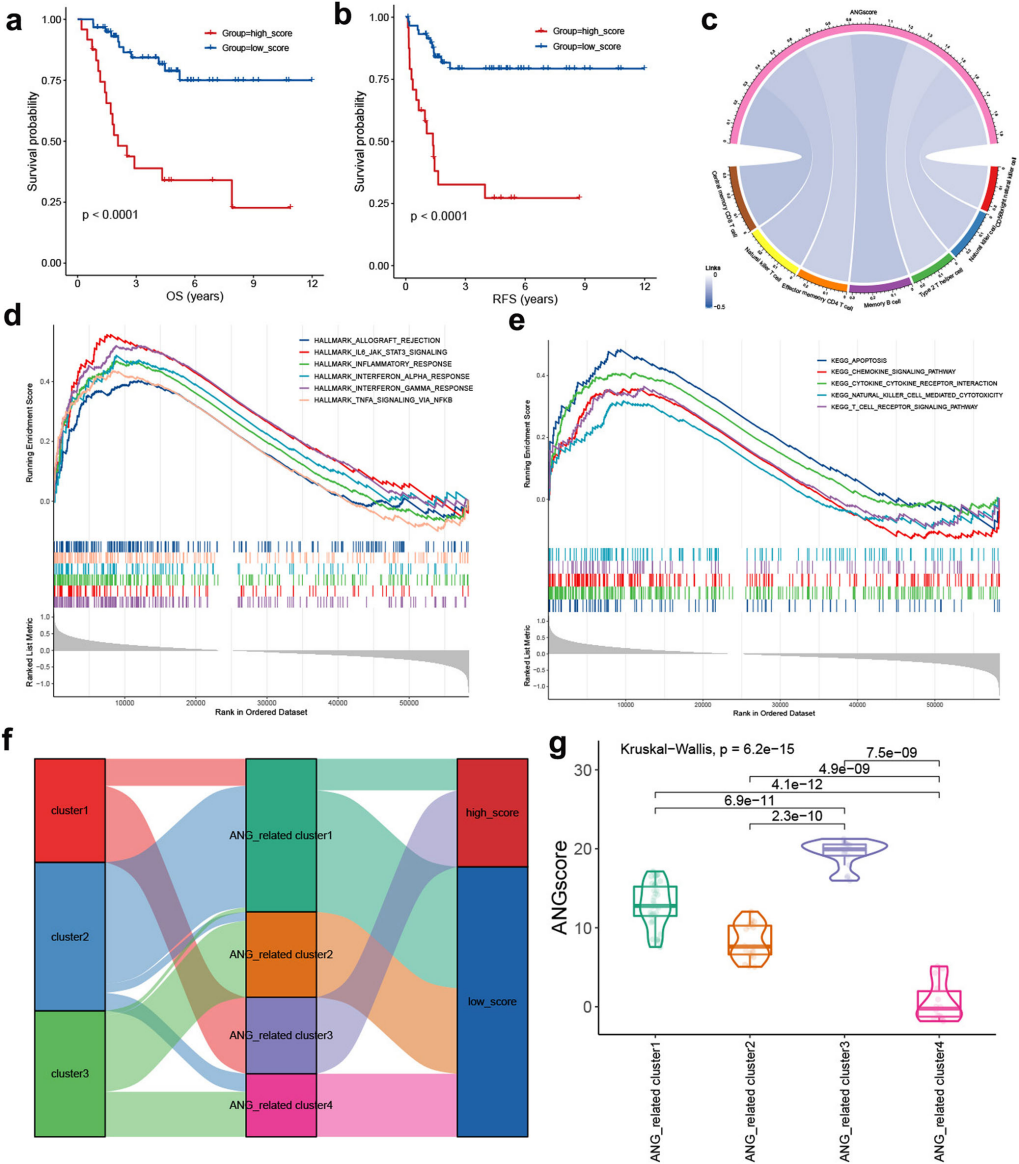
Quantification system based on ANGscore in the TARGET-OS cohort. **a**, **b** Survival analysis for overall survival (OS) and relapse-free survival (RFS) for patients with high and low ANGscore. **c** Correlations between ANGscore and TME components. **d**, **e** GSEA analysis of hallmark and KEGG between the high ANGscore group and the low ANGscore group. **f** Alluvial diagram showing the connection of angiogenesis patterns, angiogenesis-related clusters and ANGscore. **g** Differences in ANGscore among four angiogenesis-related gene clusters. The Kruskal–wallis test was used to compare the significant differences.

To improve the classification system for clinical application, we first performed PCA on the 32 DEGs, and the ANGscore was calculated for each patient using the formula based on the PCA weights. We divided patients into a high score group and a low score group, and found that the high score group exhibited worse survival than the low score group (Fig. 6a, b). We also explored the connection between the ANGscore and TME components. Pearson correlation analysis showed that the ANGscore had significantly negative correlations with NK cells, NK T cells, central memory CD4 T cells and CD8 T cells (Fig. 6c). We subsequently conducted GSEA between the low and high ANGscore groups based on Hallmark and KEGG pathways and found that the low score group was significantly enriched in immune related Hallmark pathways, including allograft rejection and IL6/JAK/STAT3 signalling (Fig. 6d). In the KEGG pathways, the low score group was also enriched in chemokine signalling pathway, cytokine to cytokine receptor interaction, natural killer cell mediated cytotoxicity and T-cell receptor signalling pathway (Fig. 6e), consistent with the outcome of TME immune cell infiltration.

To understand the attribute changes between the clustering classification and ANGscore system of individual patients, we used an alluvial diagram for visualization. Interestingly, Cluster 2, with a good prognosis, belonged to the low ANGscore group, while ANG-related Cluster 1 and Cluster 4 belonged to the same group (Fig. 6f). On the other hand, we found that ANG-related Cluster 3 had the highest ANGscore, which may contribute to its worst survival among the four clusters (Fig. 6g).

### Correlation of ANGscore with the malignancy of osteosarcoma cells

**Fig. 7:**
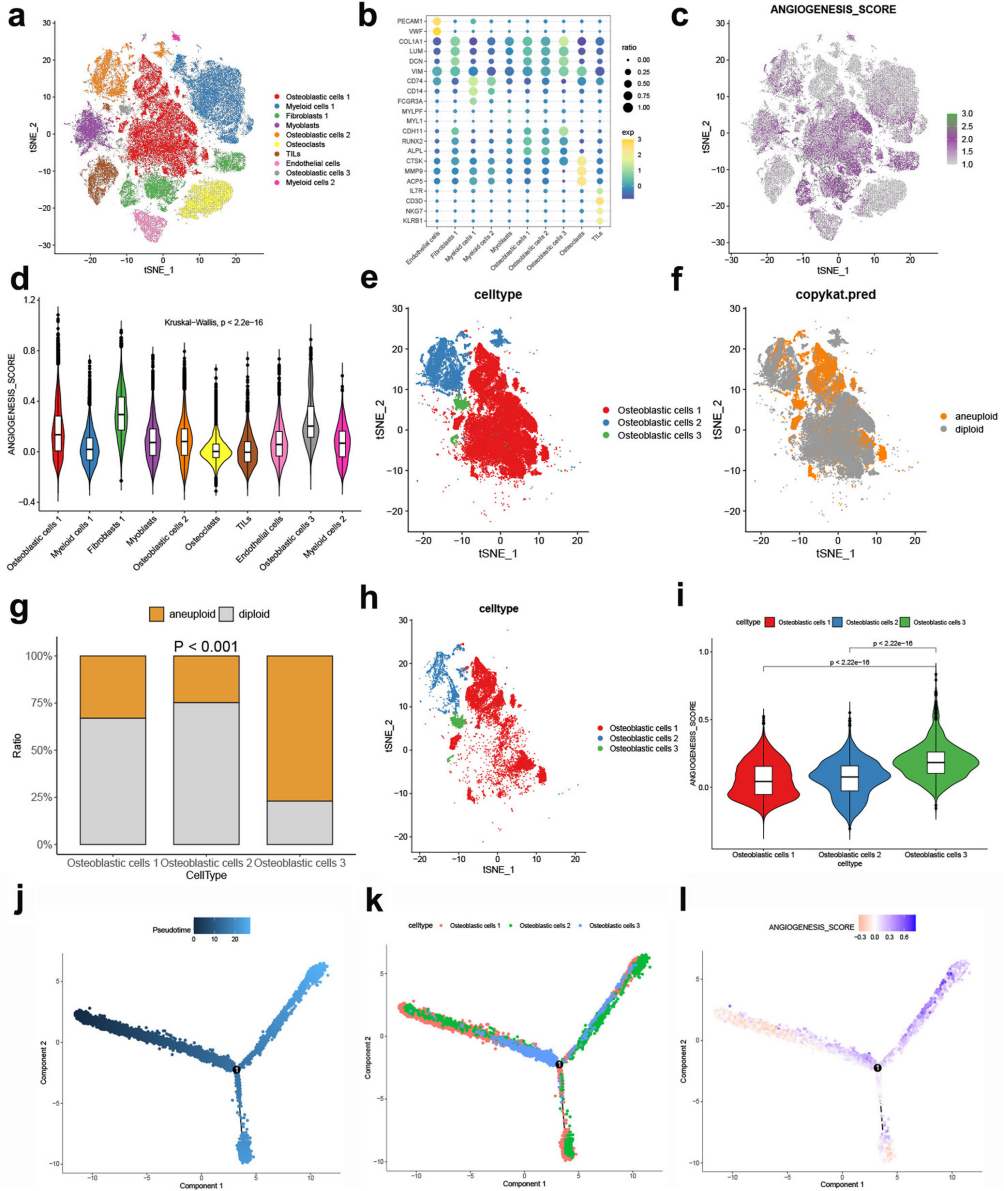
High ANGscore showed an elevated malignancy phenotype in the scRNA-seq data from OS patients. **a** t-SNE plot showing the composition of 10 cell clusters. **b** Bubble plot of the average and ratio expression of marker genes in different cell subtypes. **c** The dynamics of ANGscore in the t-SNE plot. **d** Violin plot of the ANGscore in 10 cell clusters. **e** t-SNE plot showing the subpopulation analysis of the osteoblastic cells. **f** t-SNE plot showing the somatic copy number alterations analysis of the osteoblastic cells. **g** Proportion of malignant cells in the osteoblastic cells. **h** t-SNE plot showing the aneuploid from the osteoblastic cells. **i** Violin plot of the ANGscore in the osteoblastic cells. **j** Pseudotime analysis of the aneuploid from the osteoblastic cells. **k** Pseudotime analysis according to the osteoblastic cell clusters. **l** Pseudotime analysis according to the ANGscore.

We next used scRNA-seq data to identify the underlying modulators in the TME that contribute to the angiogenesis patterns. After quality control, removal of double cells and processing to eliminate batch effects (Supplementary Fig. 3), we clustered cells into 10 main clusters, including osteoblastic cells, myeloid cells, fibroblasts, myoblasts, osteoclasts, TILs and endothelial cells, based on the reported cell markers (Supplementary Table 4, Fig. 7a, b). We found that most osteoblastic cells had overall higher ANGscores than other cell types (Fig. 7c). Considering that osteoblastic cells are one of the major malignant OS cells in clinical and its high ANGscore (Fig. 7d), we then employed somatic copy number alteration analysis in osteoblastic cells through the R CopyKAT package. Aneuploids were identified as malignant cells (Fig. 7e, f). Interestingly, Osteoblastic cells 3, a group of relatively small and independent osteoblastic cells, were almost all malignant cells (Fig. 7g, h). Osteoblastic cells 3 had the highest ANGscore among the three osteoblastic cells clusters (Fig. 7i), indicating the robustness of the ANGscore system in different datasets. To understand the evolutionary trajectory of the three malignant osteoblastic cells, we applied pseudotime analysis and found that osteoblastic cells 3 was mainly located in the middle and rear segments of the trajectory (Fig. 7j, k), which meant osteoblastic cells 3 had a higher degree of malignant potential. ANGscore also gradually increased as trajectory progressed (Fig. 7l), suggesting the validity of this scoring system.

**Fig. 8:**
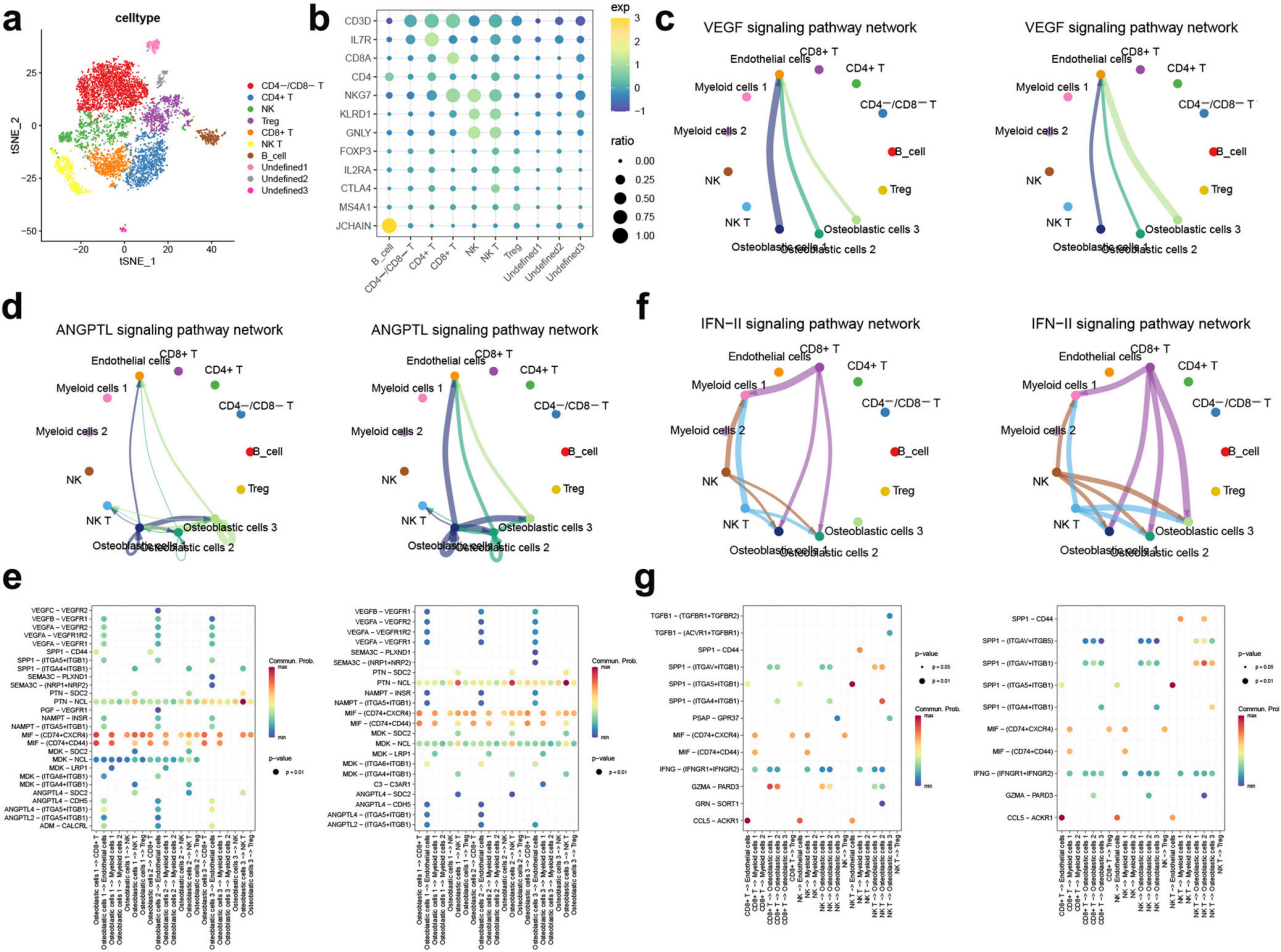
Cellular communication analysis in aneuploid between the high score group and the low score group. **a** t-SNE plot showing the subpopulation analysis of the TILs. **b** Bubble plot of the average and ratio expression of marker genes in different TILs subtypes. **c** Circos plots displaying putative ligand-receptor interactions between osteoblastic cells and other TILs cell clusters from the low ANGcore (left) and high ANGscore (right) group in VEGF signalling pathway. **d** Circos plots displaying putative ligand-receptor interactions between osteoblastic cells and other TILs cell clusters from the low ANGcore (left) and high ANGscore (right) group in ANGPTL signalling pathway. **e** Bubble plot showing the receptor-ligand binding interactions from osteoblastic cells to other cell clusters in the low ANGcore (left) and high ANGscore (right) group. **f** Circos plots displaying putative ligand-receptor interactions between osteoblastic cells and other TILs cell clusters from the low ANGcore (left) and high ANGscore (right) group in IGF-γ signalling pathway. **g** Bubble plot showing the receptor-ligand binding interactions from TILs subtypes to osteoblastic cells in the low ANGcore (left) and high ANGscore (right) group.

To characterize the complex TME in both the high score and low score groups, we used CellChat to assess interactions between different cells. We first extracted TILs and then further clustered and annotated this group of cells (Fig. 8a, b). Consistent with previous results, pathways associated with angiogenesis, such as the VEGF signalling pathway and ANGPTL signalling pathway networks, were notably strengthened in the high score group, especially osteoblastic cells 3 (Fig. 8c, d). The analysis of ligand–receptor interactions showed that the communication signals of VEGFB-VEGFR1 and ANGPTL2-(ITGA5 + ITGB1) were significantly enhanced (Fig. 8e). VEGFB is essential for the survival of blood vessels through regulating the expression of many vascular pro-survival genes via VEGFR1^25^. ANGPTL2 has also been reported to promote lymph node and distant metastases in skin squamous cell carcinoma through tumour angiogenesis^26^. Interestingly, the immune-related IFN-γ signalling pathway network was also strengthened in the high score group (Fig. 8f). In osteoblastic cells 3, there was upregulated signalling of IFNG-(IFNGR1 + IFNGR2) sent by CD8+ T cells and NK cells (Fig. 8g), suggesting that IFN-γ signalling plays a crucial role in the progression of OS and regulation of the immune microenvironment. These results further validated the universality and effectiveness of the ANGscore scoring system in the single cell level.

### ANGscore predicts prognosis and clinical response to immune

#### Therapy in OS

**Fig. 9:**
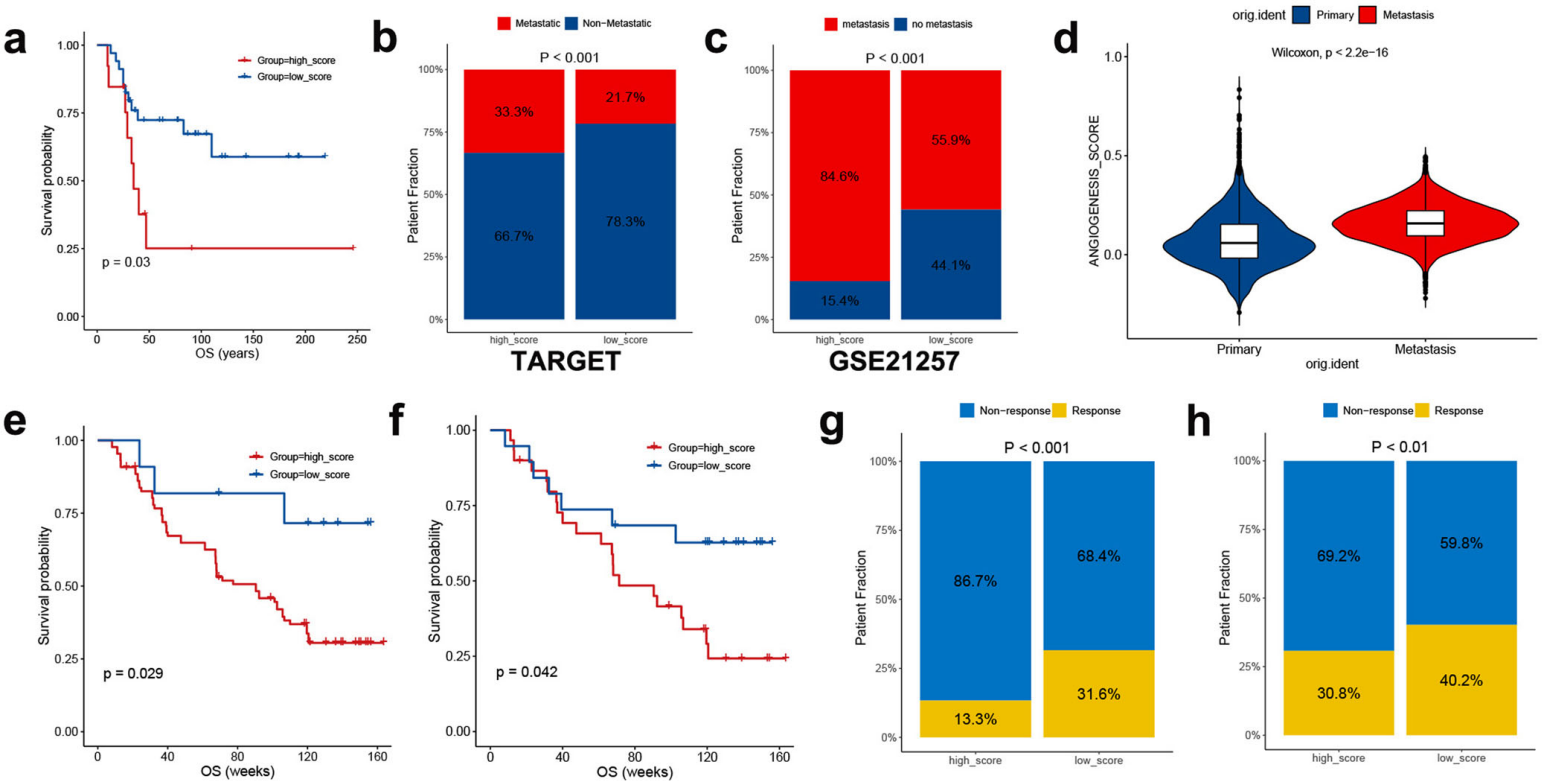
Implication of the ANGscore and its role in the prediction of response to immunotherapy. **a** Survival analysis for overall survival (OS) for patients with high and low ANGscores in GSE21257 cohort. **b**, **c** The proportion of patients who was metastatic in the low ANGscore and the high ANGscore groups in the TARGET-OS cohort and GSE21257 cohort **d** Difference in ANGscore between primary and metastatic samples from the GSE154884 dataset. **e**, **f** Survival analysis for overall survival (OS) for all samples and samples only during the treatment process with high and low ANGscore in GSE91061 cohort. **g**, **h** The proportion of patients who responded to immunotherapy in the low ANGscore and the high ANGscore groups in the GSE91061 cohort and GSE173839 cohort.

To validate the robustness of the ANGscore, we calculated the score for each sample with complete clinical data in the external validation set (GSE21257). We observed prognostic improvement in the low score group compared with the high score group (*P* = 0.03) (Fig. 9a) and patients with metastasis in the high score group had a higher proportion in both the TARGET-OS and GSE21257 datasets (Fig. 9b, c). At the same time, the ANGscore of the cells from metastasis samples was significantly elevated in the single cell dataset (Fig. 9d). Based on the relationship between the ANGscore and immune cells, we explored the prediction of the ANGscore for prognosis and immunotherapy response rate in another external validation set (GSE91061), which included melanoma patient RNA-seq data and clinical information containing prognosis and response to immunotherapy. In both all samples and samples only during the treatment process, samples with a low ANGscore had a better prognostic benefit (Fig. 9e, f). Patients during treatment in the high score group had a higher percentage of non-response (Fig. 9g), which was also reflected in another GSE173839 cohort (Fig. 9h). Together, these findings indicate that the ANGscore is a potential and stable predictor of the prognosis and responses to immunotherapy in OS.

#### Efficacy of the ANGscore in predicting drug sensitivity

**Fig. 10:**
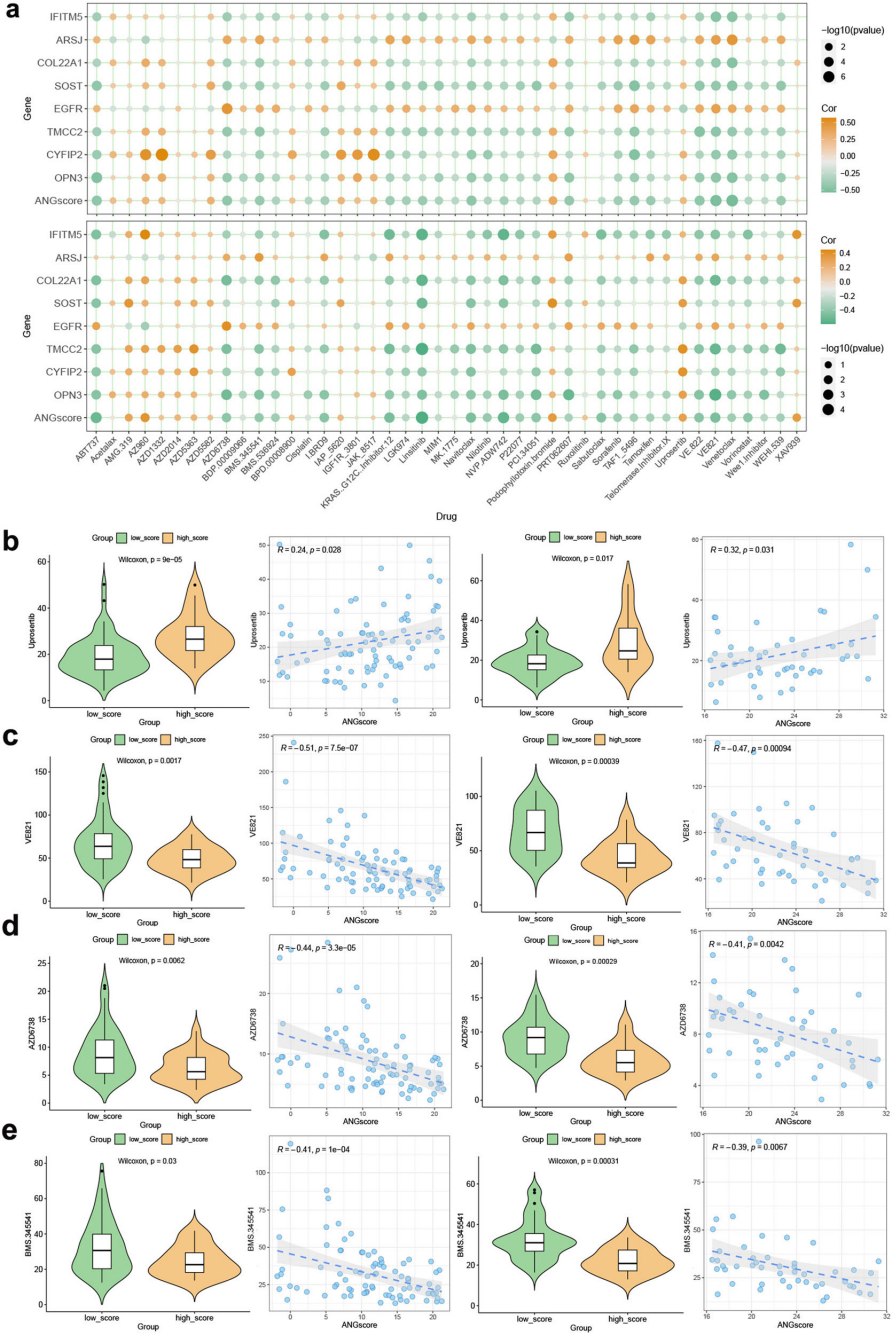
Efficacy of the ANGscore in predicting drug sensitivity. **a** Bubble plots of the relationship between ANGscore, angiogenesis genes and drugs in the TARGET-OS (up) cohort and GSE21257 (down) cohort. **b**–**e** Boxplots of the comparison of IC50 of drugs between the low ANGscore and the high ANGscore groups, and correlation between the IC50 and ANGscore in the TARGET-OS (left) cohort and GSE21257 (right) cohort.

To explore candidate drugs for patients identified by the ANGscore system, we calculated the half maximal inhibitory concentration (IC50) value of drugs from GDSC (Supplementary Table 5 and 6). The landscape of the correlation between the ANGscore and IC50 value of drugs in respectively TARGET-OS dataset and GSE21257 cohort is shown in Fig. 10a. The targets of these drugs are listed in the Supplementary table 7. As a whole, the IC50 of most drugs was inversely proportional to the ANGscore and the expression of some angiogenesis genes. We explored the drugs with the highest correlation with ANGscore in both TARGET-OS and GSE21257 datasets and found that the IC50 value of uprosertib, an inhibitor of AKT, was positively correlated with the ANGscore in both two cohorts, and there were significant differences between the high score group and the low score group (Fig. 10b). On the other hand, the IC50 values of VE821, AZD6738, BMS.345541 were lower in the high score group (Fig. 10c–e). These results suggest that high score patients identified by the ANGscore system may be resistant to uprosertib, and the other three drugs may be potential candidates for the high score population in OS. Finally, the overall mechanism figure of this study is shown in Supplementary Fig. 4.

## Discussion

This study analysed angiogenesis patterns comprehensively in OS for the first time, and constructed an angiogenesis scoring system, which was identified to be correlated with the infiltration of immune cells, the cancer immunity cycle and drug sensitivity, in the TARGET-OS dataset, and further validated its robustness and effectiveness in other external cohorts, including GSE21257 (Bulk RNA-seq cohort), GSE152048 (single cell RNA-seq cohort), GSE91061 and GSE173839 (two immunotherapy cohorts).

Angiogenesis is considered an extremely complicated process in tumour progression, that attributes to the imbalance between proangiogenic and antiangiogenic factors^27^. The perturbed equilibrium of angiogenesis will subsequently generate an immunosuppressive microenvironment. Hyperactive angiogenesis can elevate differential pressure and downregulate endothelial adhesion molecules, which results in a reduction in the number of tumour-infiltrating lymphocytes^28^. Consistent results were found in our study. In the training set, among the three angiogenesis patterns based on the clustering of angiogenesis genes, Cluster 2 had a higher abundance of immune cell infiltration than Cluster 1 and Cluster 3, especially in CD8 T cells, NK cells and NK T cells. In contrast, the expression levels of proangiogenic genes VEGFA and FSTL1 (Follistatin Like 1) in Cluster 2 were the lowest, suggesting the lowest angiogenesis activity of Cluster 2. The immunity cycle was also activated in Cluster 2, especially in NK cell, macrophage and CD8 T cell recruitment. Increased vascular activation in osteosarcoma suggests a lack of immune infiltration. TIMP1, highly expressed in Cluster 2, was reported to interfere with the TGFβ induced decidual-like and pro-angiogenesis phenotypes in NK cells and was envisaged as a modulator of NK cells^29^. In addition, FSTL1 was involved in bone metastasis by generating a microenvironment of low-activity cytotoxic lymphocytes^30^, which was consistent with the phenomenon of its higher expression in Cluster 1 and Cluster 3. The ANGscore system, which could comprehensively quantify the angiogenesis state for OS patients, was found to be significantly negatively correlated with specific components of the TME, such as NK cells, NK T cells and central memory CD8 T cells, further indicating the interaction between the tumour immune microenvironment and angiogenesis.

In recent years, the continuous development of single-cell sequencing technology has made it a better technique to explore tumour heterogeneity, immune cell infiltration, and intercellular communication than bulk RNA-seq technology. We identified a novel cluster of osteoblastic cells with the highest ANGscore by analysing scRNA-seq data, and classical angiogenesis signallings such as VEGFA and ANGPTL signallings in intercellular communications, were activated in the high score group. It is worth noting that in the high score group, the osteoblastic cell cluster received higher IFN-γ signalling from CD8 T cells, NK cells and NK T cells. Another recently published study of the scRNA-seq data for OS, which also identified an IFN-γ dominant cluster of OS patients with immunosuppressive phenotype and worsening prognosis^31^. Activation of the IFN-γ pathway may accelerate phenotypic induction of tumour cells by immune cells in OS. Previous work has established that IFN-γ plays a crucial role in tumour angiogenesis suppression^32,33^. Studies have reported that CD8 CTLs can release IFN-γ to impress the proliferation and migration of endothelial cells, thereby restraining tumour vascularization^34–36^. Activated NK cells can also secrete large amounts of IFN-γ to activate downstream signalling pathways, such as signal transducers and activators of transcription (STAT) and control the infection. At the same time, IFN-γ released by NK cells could also restrain tumour angiogenesis and stimulate T-cell responses in lymph nodes^37–39^. However, IFN-γ can also promote angiogenesis under specific conditions. NK cells in the first-trimester decidua (dNK) mainly produce a great deal of IFN-γ and proangiogenic cytokines, including VEGF and angiogenin, and show weak cytotoxicity compared with peripheral blood NK (pNK) cells^40–42^. There are also reports that demonstrate that NK cells infiltrating tumours exhibit a decidual-like phenotype^42,43^. The specific marker CD9 of dNK cells was also detected in tumour-infiltrating NK cells^44–46^, which indicated that tumour infiltrating NK cells may be polarized into a decidual-like phenotype and thus reduce toxicity and promote tumour. An enhanced IFN-γ signal of NK cells against osteoblastic cells was also observed in our results. The above results demonstrated the association of IFN-γ signalling with malignant features of osteosarcoma cells, suggesting the potential of drug therapy targeting this pathway.

With the bottleneck encountered after the standardized treatment of OS patients, basic research and clinical trials on immunotherapy for OS are increasing. Blockade of PD-1/PD-L1 interactions in a mouse model of metastatic OS can improve the response of CTL to OS, and also enhance the chemotherapy effect of cisplatin on osteosarcoma^47,48^. The overall expression of immune checkpoints in cluster 2 was also elevated, suggesting that this population may be more sensitive to immune checkpoint inhibitors. In addition to immune checkpoints, cytokines can influence the efficacy of immunotherapy by regulating the activity and composition of immune cells in the tumour microenvironment. The cytokine TGF-β is an important cause of metastasis and chemotherapy resistance in osteosarcoma^49^. Compared with TGF-β blockers alone, TGF-β blockers combined with DC vaccines showed stronger CTL activity and antitumour effects^50^. Interestingly, TGFβ can polarize CD56dimCD16+ pNK cells to an immunosuppressive, proangiogenic CD56bright dNK phenotype^42–44^, indicating the possibility of TGFβ-mediated angiogenesis. This suggests great potential for future combination therapies combining cytokines and antiangiogenic drugs for OS patients.

Additionally, nearly a third of clinical trials for OS patients are currently conducted with the drugs targeting specific molecule or relevant pathways, including PARP, tyrosine kinases and the PI3K/AKT/mTOR pathway, etc^51^. However, we found OS patients with high ANGscore may be resistant to uprosertib, an inhibitor targeting the PI3K/AKT/mTOR signaling. The ANGscore signature assessment can help patients choose other more sensitive targeted drug therapies. ATR (ataxia telangiectasia and Rad3-related), the common drug target of VE821 and AZD6738, is a serine/threonine kinase and DNA damage sensor, which is essential for promoting deoxynucleotide synthesis, initiating replication forks, and repairing DNA double-strand breaks^52^. Notably, combined ATR and PARP inhibition has been reported to destabilize stalled replication forks and exhibit synergistic toxicity to tumour cells^53,54^, and it may be a potential targeted therapy for high ANGscore osteosarcoma population.

Several studies have developed related signatures for OS, but there are shortcomings, such as only prognosis prediction, too few datasets and too shallow mechanism mining. Our analysis demonstrated that the ANGscore is an independent prognostic evaluation index for OS, providing an ideal and potential predictor of prognosis, metastasis and therapeutic response for OS patients. However, there are remain limitations in our study. Although the ANGscore exhibited excellent performance of in two bulk RNA-seq cohorts, one scRNA-seq cohort and two immunotherapy cohorts, prospective cohort studies are still needed to confirm its reliability. In addition, we performed comprehensive pseudotime and intercellular communication analyses of single cell sequencing data. Nevertheless, only one scRNA-seq dataset may still have population limitations, and more single-cell data need to be integrated in the future. Moreover, some angiogenesis genes have not been investigated in both in vitro and in vivo experiments, which are essential for an in-depth exploration of the mechanisms underlying the angiogenesis pattern of OS in the future. In conclusion, the ANGscore system developed in this study is a practical predictor of prognosis and therapeutic response for OS patients including postoperative patients, which has great potential for in the assessment of clinical outcomes.

## Methods

### Data acquisition and preprocessing

The log (FPKM + 1) expression data of the TARGET-OS dataset were downloaded from the UCSC Xena data portal (https://xenabrowser.net/datapages/) and transformed into transcripts per kilobase million (TPM). The expression profiling of the array dataset (GSE21257), the single-cell sequencing dataset (GSE152048) and two immunotherapy-related datasets (GSE91061 and GSE173839) were downloaded from the Gene Expression Omnibus (GEO) database (https://www.ncbi.nlm.nih.gov/gds). Briefly, patients with fully equipped clinical messages, including survival time, grade, and metastasis status were included in our subsequent analyses (Supplementary Table 8).

For the RNA-seq and microarray data with probe annotation, we performed quality control and normalization before analyses. For the scRNA-seq data, we first performed quality control with the R package Seurat^55^, removed doublet cells with the R package doubletfinder^56^ and batch effect with the R package harmony^57^. Subsequently, we performed dimensionality reduction, and cell clustering based on the t-SNE algorithm. CopyKAT was used to detect somatic copy number alterations to identify malignant cells^58^. CellChat was used to analyse the intercellular communication networks between different cell types^59^. Monocle was performed to construct the pseudotime trajectory revealing the progression of malignant cells^60^.

The angiogenesis gene list was downloaded from the MSigDB database GSEA (https://www.gsea-msigdb.org/gsea) hallmark gene sets.

The work has been reported in line with the REMARK criteria.

### Unsupervised consensus clustering

We performed the consensus clustering algorithm based on the k-means method using the R package ConsensusClusterPlus with 1,000 repetitions to identify distinct angiogenesis-related patterns^61^. Subsequently, we used the R packages survival and survminer to explore the prognostic difference between clusters.

### Gene set variation analysis (GSVA) and single sample gene set enrichment analysis (ssGSEA)

GSVA enrichment analysis was performed with the R package GSVA^62^. Pathways of hallmark were also downloaded from the MSigDB database. Signature gene sets were collected from previous literature^63^. The key gene list representing the process of the cancer immunity cycle (Supplementary table 9) was obtained from Mellman et al.^64^. The immune step score indicating specific activities was calculated by the ssGSEA algorithm based on expression of the above genes. Immune cell infiltration in the tumour microenvironment was also estimated by ssGSEA based on the specific genes summarized in a previous study^65^.

### Identification of differentially expressed genes (DEGs) and univariate regression

The log (TPM + 1) expression data were analysed to detect DEGs between each pair of the three angiogenesis-related patterns based on t tests with the R package limma^66^. Common DEGs were identified by interacting DEGs between each two clusters of the three angiogenesis patterns. Subsequently we performed univariate Cox regression on above the DEGs with their expression and overall survival, and genes with a false discovery rate (FDR) < 0.05 were defined as angiogenesis-related genes.

### Development of an angiogenesis-related signature

To construct a quantitative score to predict the angiogenesis pattern and prognosis of patients, angiogenesis-related genes were extracted to perform principal component analysis (PCA). The first two principal components (PC1 and PC2) were used to explain the importance of each variable. By referring to a previous method of constructing model, we developed a scoring algorithm called ANGscore to quantify the state of angiogenesis:

$${\rm{ANGscore}}=\sum ({\rm{PC}}1\ast {\exp }_{{\rm{i}}}+{\rm{PC}}2\ast {\exp }_{{\rm{i}}})$$

The exp_i_ represents the expression level of the angiogenesis-related genes, while PC1 and PC2 are the first two principal components generated by PCA. Each patient was calculated for an ANGscore to estimate the extent of patient angiogenesis.

For scRNA-seq data, we calculated ANGscore through the Seurat function “AddModuleScore”, which averaged the expression of angiogenesis genes in each single cell.

### Drug sensitivity analysis

The OncoPredict R package was applied to predict the candidate drug responses in cancer patients by fitting the expression of specific genes to obtain the half-maximal inhibitory concentration (IC50) of the cancer cell lines^67^. The drug list was downloaded from Genomics of Drug Sensitivity in Cancer (GDSC; https://www.cancerrxgene.org/). The expression profile of the reference cell line was downloaded from the Broad Institute Cancer Cell Line Encyclopedia (CCLE; https://portals.broadinstitute.org/ccle_legacy/home). The sensitivity and resistance of candidate drugs between the high score and low score groups were analysed using the Wilcoxon test with p < 0.05 as the threshold for statistical significance.

### Statistical analysis

Correlations between variables were estimated with Pearson correlation analysis. For continuous variables, *t* test or Wilcoxon test were used to compare differences between two groups, while Kruskal–Wallis tests were performed for multiple groups. Survival curves were generated using Kaplan–Meier method in each dataset and compared with the log-rank test. All statistical analyses were implemented via R software (v.4.0.2). Two-sided *P* values < 0.05 were considered statistically significant.

### Reporting summary

Further information on research design is available in the Nature Research Reporting Summary linked to this article.

## Supplementary information

Supplementary materials

REPORTING SUMMARY

## Data Availability

The data in this study are available from the corresponding author upon reasonable request. The expression profiling of the array dataset (GSE21257), the single-cell sequencing dataset (GSE152048) and two immunotherapy-related datasets (GSE91061 and GSE173839) were downloaded from the Gene Expression Omnibus (GEO) database. The expression profiling of the TARGET-OS dataset was downloaded from the UCSC XENA database (https://xenabrowser.net/datapages/).

## Code availability

The underlying code for this study is not publicly available but may be made available from the corresponding author upon reasonable request.
