# Supplementary material for: Comprehensive analysis of angiogenesis pattern and related immune landscape for individual treatment in osteosarcoma
Source: NPJ Precis Oncol. 2023 Jun 29;7:62. doi: 10.1038/s41698-023-00415-7 (PMC10310742; doi:10.1038/s41698-023-00415-7)
Supplement: Supplementary file 1 — Supplementary materials [file 41698_2023_415_MOESM1_ESM.pdf]

Supplementary Table 1. Hallmark of angiogenesis.

Gene symbol
APOH
APP
CCND2
COL3A1
COL5A2
CXCL6
FGFR1
FSTL1
ITGAV
JAG1
JAG2
KCNJ8
LPL
LRPAP1
LUM
MSX1
NRP1
OLR1
PDGFA
PF4
PGLYRP1
POSTN
PRG2
PTK2
S100A4
SERPINA5
SLCO2A1
SPP1
STC1
THBD
TIMP1
TNFRSF21
VAV2
VCAN
VEGFA
VTN

Supplementary Table 2. Common cancer-related signature

Gene Signature	Genes Symbol
**CD8 T effector**	CD8A, CXCL10, CXCL9, GZMA, GZMB, IFNG, PRF1, TBX21
**DNA damage repair**	ALKBH2, ALKBH3, APEX1, APEX2, APLF, ATM, ATR, ATRIP, BLM, BRCA1, BRCA2, BRIP1, CCNH, CDK7, CETN2, CHAF1A, CHEK1, CHEK2, CLK2, DCLRE1C, DDB1, DDB2, DUT, ENDOV, ERCC1, ERCC2, ERCC3, ERCC4, ERCC5, ERCC6, ERCC8, FAN1, FANCA, FANCB, FANCC, FANCD2, FANCE, FANCF, FANCG, FANCL, FANCM, GTF2H1, GTF2H2, GTF2H3, GTF2H4, GTF2H5, H2AFX, HLTF, HUS1, LIG1, LIG3, LIG4, MBD4, MDC1, MGMT, MLH1, MLH3, MMS19, MNAT1, MPG, MSH2, MSH3, MSH4, MSH5, MSH6, MUTYH, NEIL1, NEIL2, NEIL3, NHEJ1, NTHL1, NUDT1, OGG1, PALB2, PARP1, PARP2, PARP3, PCNA, PER1, PMS1, PMS2, PNKP, POLB, POLD1, POLE, POLG, POLH, POLL, POLM, POLQ, PRKDC, RAD1, RAD17, RAD18, RAD23A, RAD23B, RAD51C, RAD9A, RECQL4, RECQL5, RIF1, RNF168, RNF4, RNF8, RPA1, RPA2, RPA3, RPA4, RRM2B, SETMAR, SHPRH, SMUG1, TDP1, TDP2, TOPBP1, TP53, TREX1, UBE2A, UBE2B, UBE2N, UBE2V2, UNG, UVSSA, WRN, XAB2, XPA, XPC, XRCC1, XRCC4, XRCC5, XRCC6
**Pan-F-TBRS**	ACTA2, ACTG2, ADAM12, ADAM19, CNN1, COL4A1, CTGF, CTPS1, FAM101B, FSTL3, HSPB1, IGFBP3, PXDC1, SEMA7A, SH3PXD2A, TAGLN, TGFBI, TNS1, TPM1
**Antigen processing machinery**	B2M, HLA-A, HLA-B, HLA-C, TAP1, TAP2
**Immune checkpoint**	CD274, CTLA4, HAVCR2, LAG3, PDCD1, PDCD1LG2, TIGIT
**EMT1**	CLDN3, CLDN7, CLDN4, CDH1, VIM, TWIST1, ZEB1, ZEB2
**EMT2**	AXL, FAP, LOXL2, ROR2, TAGLN, TWIST2, WNT5A
**EMT3**	FOXF1, GATA6, SOX9, TWIST1, ZEB1, ZEB2
**FGFR3-related genees**	FGFR3, TP63, WNT7B
**KEGG discovered histones**	HIST1H2AG, HIST1H2AI, HIST1H2BL, HIST2H2BF
**Fanconi anemia**	APITD1, ATR, ATRIP, BLM, BRCA1, BRCA2, BRIP1, C17orf70, C19orf40, EME1, EME2, ERCC1, ERCC4, FAN1, FANCA, FANCB, FANCC, FANCD2, FANCE, FANCF, FANCG, FANCI, FANCL, FANCM, HES1, MLH1, MUS81, PALB2, PMS2, POLH, POLI, POLK, POLN, RAD51, RAD51C, REV1, REV3L, RMI1,

	RMI2, RPA1, RPA2, RPA3, RPA4, SLX4, STRA13, TELO2, TOP3A, TOP3B, UBE2T, USP1, WDR48
**Cell cycle**	ABL1, ANAPC1, ANAPC10, ANAPC11, ANAPC13, ANAPC2, ANAPC4, ANAPC5, ANAPC7, ATM, ATR, BUB1, BUB1B, BUB3, CCNA1, CCNA2, CCNB1, CCNB2, CCNB3, CCND1, CCND2, CCND3, CCNE1, CCNE2, CCNH, CDC14A, CDC14B, CDC16, CDC20, CDC23, CDC25A, CDC25B, CDC25C, CDC26, CDC27, CDC45, CDC6, CDC7, CDK1, CDK2, CDK4, CDK6, CDK7, CDKN1A, CDKN1B, CDKN1C, CDKN2A, CDKN2B, CDKN2C, CDKN2D, CHEK1, CHEK2, CREBBP, CUL1, DBF4, E2F1, E2F2, E2F3, E2F4, E2F5, EP300, ESPL1, FZR1, GADD45A, GADD45B, GADD45G, GSK3B, HDAC1, HDAC2, MAD1L1, MAD2L1, MAD2L2, MCM2, MCM3, MCM4, MCM5, MCM6, MCM7, MDM2, MYC, ORC1, ORC2, ORC3, ORC4, ORC5, ORC6, PCNA, PKMYT1, PLK1, PRKDC, PTTG1, PTTG2, RAD21, RB1, RBL1, RBL2, RBX1, SFN, SKP1, SKP2, SMAD2, SMAD3, SMAD4, SMC1A, SMC1B, SMC3, STAG1, STAG2, TFDP1, TFDP2, TGFB1, TGFB2, TGFB3, TP53, TTK, WEE1, YWHAB, YWHAE, YWHAG, YWHAH, YWHAQ, YWHAZ, ZBTB17
**DNA replication**	DNA2, FEN1, LIG1, MCM2, MCM3, MCM4, MCM5, MCM6, MCM7, PCNA, POLA1, POLA2, POLD1, POLD2, POLD3, POLD4, POLE, POLE2, POLE3, POLE4, PRIM1, PRIM2, RFC1, RFC2, RFC3, RFC4, RFC5, RNASEH1, RNASEH2A, RNASEH2B, RNASEH2C, RPA1, RPA2, RPA3, RPA4, SSBP1
**Nucleotide excision repair**	CCNH, CDK7, CETN2, CUL4A, CUL4B, DDB1, DDB2, ERCC1, ERCC2, ERCC3, ERCC4, ERCC5, ERCC6, ERCC8, GTF2H1, GTF2H2, GTF2H3, GTF2H4, GTF2H5, LIG1, MNAT1, PCNA, POLD1, POLD2, POLD3, POLD4, POLE, POLE2, POLE3, POLE4, RAD23A, RAD23B, RBX1, RFC1, RFC2, RFC3, RFC4, RFC5, RPA1, RPA2, RPA3, RPA4, XPA, XPC
**Homologous recombination**	BLM, BRCA2, EME1, MRE11A, MUS81, NBN, POLD1, POLD2, POLD3, POLD4, RAD50, RAD51, RAD51B, RAD51C, RAD51D, RAD52, RAD54B, RAD54L, RPA1, RPA2, RPA3, RPA4, SHFM1, SSBP1, TOP3A, TOP3B, XRCC2, XRCC3
**Mismatch repair**	EXO1, LIG1, MLH1, MLH3, MSH2, MSH3, MSH6, PCNA, PMS2, POLD1, POLD2, POLD3, POLD4, RFC1, RFC2, RFC3, RFC4, RFC5, RPA1, RPA2, RPA3, RPA4, SSBP1
**WNT target**	EFNB3, MYC, TCF12, VEGFA
**Cell cycle regulators**	ATM, CCND1, CCNE1, CDKN1A, CDKN2A, E2F3, FBXW7, MDM2, RB1, TP53

Supplementary Table 3. Prognostic analysis of 32 angiogenesis-related genes using a univariate Cox regression model.

ID	HR	95% CI	p.value
WAS	0.601	(0.405-0.893)	0.012
OPN3	1.46	(1.15-1.85)	0.0022
CYFIP2	1.38	(1.09-1.76)	0.0077
GNA15	0.68	(0.471-0.982)	0.04
MMP2	0.711	(0.509-0.994)	0.046
CYTH4	0.651	(0.449-0.944)	0.024
ANXA13	2.62	(1.63-4.23)	0.000073
CDK6	0.542	(0.366-0.803)	0.0023
PTN	0.809	(0.679-0.963)	0.017
ITK	1.6	(1.18-2.18)	0.0028
NCKAP1L	0.558	(0.355-0.876)	0.011
WDFY4	0.335	(0.114-0.986)	0.047
LOXL1	0.722	(0.572-0.911)	0.006
GFPT2	0.705	(0.501-0.992)	0.045
TMCC2	1.61	(1.14-2.26)	0.0065
EGFR	0.635	(0.433-0.932)	0.021
ARL11	0.316	(0.117-0.852)	0.023
TSPAN18	0.645	(0.463-0.9)	0.0098
CHMP4C	1.31	(1.04-1.65)	0.024
WDR72	1.72	(1.28-2.31)	0.00033
SOST	1.23	(1.08-1.39)	0.0015
GNG4	1.44	(1.14-1.84)	0.0028
COL22A1	1.36	(1.11-1.67)	0.0031
COL8A2	0.809	(0.661-0.989)	0.039
GNG12	0.679	(0.482-0.958)	0.028
SYT12	1.56	(1.14-2.15)	0.006
ARSJ	0.546	(0.305-0.978)	0.042
EPHB3	0.606	(0.41-0.895)	0.012
HIST3H2BB	1.81	(1.12-2.92)	0.015
CACNA1E	2.48	(1.2-5.11)	0.014
IFITM5	1.24	(1.03-1.48)	0.022
CTB_4E7.1	1.52	(1.19-1.93)	0.00072

Supplementary Table 4. The Anotation cell markers of single cell data.

Cell type	Markers
osteoblastic cell	COL1A1, CDH11, RUNX2, ALPL
Osteoclast	CTSK, MMP9, ACP5
Myeloid cells	LYZ, CD68, CD74, CD14, FCGR3A
Fibroblast	COL1A1, LUM, DCN, VIM
Myoblast	MYLPF, MYL1
TIL	IL7R, CD3D, NKG7, CD2, CD3E, CD3G, GNLY, KLRD1, KLRB1, MS4A1, CD79A
Endothelial cell	PECAM1, VWF, EGFL7, PLVAP

Reference

Zhou Y, Yang D, Yang Q, et al. Single-cell RNA landscape of intratumoral heterogeneity and immunosuppressive microenvironment in advanced osteosarcoma. Nat Commun. 2020 Dec 10;11(1):6322.

Liu Y, Feng W, Dai Y, et al. Single-Cell Transcriptomics Reveals the Complexity of the Tumor Microenvironment of Treatment-Naive Osteosarcoma. Front Oncol. 2021 Jul 21;11:709210.

Supplementary Table 5. The IC50 of the corresponding drug for TARGET-OS samples.

Drug	TARGET-40-PASUUH-01A	TARGET-40-PAUTWB-01A	TARGET-40-PARJXU-01A	TARGET-40-PAPWWC-01A	TARGET-40-PAUUML-01A	TARGET-40-PAMHLF-01A	TARGET-40-PAUBIT-01A	TARGET-40-PASFCV-01A	TARGET-40-PARDAX-01A	TARGET-40-0A4I4O-01A	TARGET-40-0A4I48-01A	TARGET-40-PASEFS-01A	TARGET-40-PARKAF-01A	TARGET-40-PATUXZ-01A
JQ1	20.7556324	7.349135518	9.637550422	19.46672164	10.44009028	8.339992538	13.89395607	12.62621602	4.169394907	6.346487241	10.48912109	6.122300354	10.55420787	3.841502486
BMS-754807	0.923588635	1.150347587	2.662442343	1.899050939	1.392799568	1.93460562	1.312767787	0.903733711	3.009140354	1.622852359	1.070174451	0.522153832	1.535531534	0.654005152
AT13148	60.17150964	32.12012012	105.5923048	38.14651381	53.2739456	50.40794588	38.94566882	29.84368411	62.05511096	40.18140522	54.09048712	34.96413545	34.11232105	47.34701572
AZD6482	27.71280259	23.16330746	27.48827532	24.53772926	29.90169297	20.17322172	25.41575205	21.84584518	26.16711863	22.24590904	25.08593476	31.7495661	21.67978834	21.79588625
VE821	39.311164	49.23056126	77.60245508	41.32526029	70.93393174	60.11822288	58.5758274	87.23069035	54.33186024	68.21162319	57.98248359	51.65824171	48.71665854	39.31183652
GSK591	92.07788941	93.80792539	98.2029291	82.59997276	108.1328158	89.23606129	96.04476062	81.69684828	103.9673295	122.5079184	139.2558792	71.11305565	95.11821703	99.11578294
AZ6102	11.44447729	11.19423463	11.64297788	10.86109753	16.80414452	16.43433639	13.73793401	11.03727651	10.50373428	12.26497052	16.4014972	9.108736457	9.494956197	10.07359414
LJI308	137.2052177	135.134868	193.3083776	201.5017766	198.5768712	172.0021316	192.6572858	159.2487084	152.7861488	188.9504725	226.6326976	145.3265384	124.2296523	142.0212544
Uprosertib	37.33398815	15.35470274	40.51956616	25.37869079	16.72904708	21.88294197	13.98254109	30.02329248	19.37500001	11.44231445	16.08643262	8.755576904	15.72479591	18.30757566
VX-11e	24.31222995	11.7643312	24.05045577	11.78010991	42.58934878	34.05178496	16.64106799	13.60724045	11.91836274	31.30909891	13.0462939	18.78368088	12.14156025	12.68793441
Vinorelbine	0.021184954	0.037178762	0.068002506	0.041215726	0.034322003	0.058476959	0.035306511	0.032392937	0.092510292	0.026946303	0.125215464	0.030303534	0.029034615	0.064491422
Ulixertinib	7.636379773	5.811614127	17.05472294	7.239503196	13.04053319	13.31610091	5.518688284	7.770999749	6.03504253	13.44030425	9.600144345	2.605514996	9.766153888	9.146767345
MK-8776	16.80341725	23.38703784	20.01849922	22.84374337	60.30145169	25.06199029	13.07878742	20.44790023	48.15535365	12.73941574	16.85433772	10.71985874	26.66411057	28.17377193
AMG-319	138.192822	97.41254424	181.1097764	159.6660594	163.1411614	110.5945457	137.3720559	85.82009989	169.880782	113.713328	116.1767059	113.6093867	118.7519397	169.1057391
Pyridostatin	24.45509293	24.92547243	31.77200141	41.10333063	27.19541419	22.19709648	18.69163736	27.55092711	39.64392259	25.65875973	38.5066093	18.56130398	29.7885473	25.91320489
BIBR-1532	126.245781	117.4855941	203.7877704	123.2226352	116.4247554	150.0963881	127.6531341	151.1157083	170.3032975	139.8018949	161.4620826	74.35599971	103.7064109	158.8417992
Foretinib	3.29875793	2.256297192	3.221025165	2.870801502	2.010089489	2.190976014	2.144518424	3.271110767	2.698340418	1.947379654	2.312301279	4.446748425	2.459551541	2.926778451
BPD-00008900	82.90501824	76.45877851	147.690932	129.0483625	67.64196263	105.6679462	69.77770586	71.25338291	140.1626103	73.04168631	84.67351	80.61932422	111.334205	124.1279983
WEHI-539	27.47683934	42.00996089	64.08239698	49.28377581	44.32391206	50.91699909	35.18985741	65.20453272	39.27376413	33.57877396	58.71933396	8.102196475	41.05712086	21.03701781
MIM1	38.16842597	46.93244979	79.94351017	50.24852602	59.64842219	53.40938693	35.49305884	48.09552374	43.15586734	25.76891182	58.46788232	33.73653359	32.16584105	37.16222904
Sepantronium bron	0.009368419	0.01436781	0.06106797	0.015391506	0.013947691	0.015763511	0.01443767	0.008708216	0.008292988	0.007674994	0.021848998	0.003818004	0.016540446	0.007923816
WIKI4	36.85654696	31.02675388	58.47772505	49.66377591	47.96327516	46.95775479	48.9317929	37.95745255	37.47492102	29.62946641	38.46519698	56.61508759	34.21465825	49.52214855
UMI-77	21.93281589	16.08934869	21.95475467	15.80777759	10.89218561	10.63644346	14.20449393	13.7179885	8.282833062	19.46029872	20.81432926	8.208548395	11.24723132	10.77302009
Savolitinib	11.98631329	11.96910214	12.63321221	11.1022577	12.58995693	14.49080628	13.72521563	12.12115209	10.04835746	10.03599856	20.91734291	16.84648168	10.73583497	16.51972257
P22077	57.00939883	74.20655236	212.9145809	58.86378741	104.6709338	128.2460003	112.0399014	87.96557929	45.47191404	72.37770538	120.3232383	36.2020066	57.8260675	66.07270549
NVP-ADW742	21.38519174	17.30801552	18.42694148	25.76523885	7.929388411	23.82458421	12.89551962	23.24343913	10.93599335	21.6416516	12.33183254	10.82508829	11.21693706	9.205057874
MIRA-1	260.0661996	195.1232482	286.3388138	233.9851931	209.4890761	249.2588636	205.7996514	251.2645965	252.0636804	205.463481	283.4254098	113.581843	218.1372113	263.5433522
Telomerase Inhibitc	1.431523475	1.582536529	1.855922849	1.290587652	2.010728307	1.8062506	1.874404969	1.201136913	1.186490055	1.904835336	2.1871715	0.876343277	1.095992089	1.529123573
I-BRD9	73.72513511	70.44963043	122.2885929	86.45295204	50.20866978	102.0970825	89.08513008	110.2392309	65.19504567	104.0515992	85.47250834	56.55465007	72.85317358	65.47916908
GSK2578215A	112.5518769	130.0036594	241.8797471	111.3227743	154.1439609	140.1831462	120.4848663	122.0513987	165.9141124	131.7181095	145.3858139	61.8084839	135.1279639	143.7074664
GNE-317	1.819820444	1.621073876	2.967503736	0.860304687	3.232496255	2.169134783	1.439190001	1.823441321	1.674794585	2.399365645	1.946603123	1.768658323	1.276651835	2.287925413
GDC0810	121.0984376	119.336823	198.7042533	91.25407709	178.2734731	144.619594	140.9210524	128.7739718	133.6651944	157.057083	160.6325981	77.48044976	136.4711891	166.8084958
Ipatasertib	71.52014708	29.58417833	78.68844539	40.86407922	23.71518277	44.44000094	22.6184029	51.06704285	43.85515495	33.99008723	33.0598326	19.44490076	27.69022494	43.24765705
Cediranib	13.82002581	8.42262822	10.21826276	8.63827551	8.854891388	6.739566487	6.635151675	6.617264977	12.31217803	8.296301793	8.379277925	7.479655982	9.219838289	7.988467982
Osimertinib	7.332183391	3.729071425	11.4624486	3.878817807	7.22619629	5.388110126	6.900626638	5.581405921	6.447408014	5.714970375	4.808779072	2.228690769	3.315209141	4.361046281
AZD8186	79.26915617	20.95851411	30.20310783	27.72369358	33.90727012	22.22606599	32.64893457	20.166265	34.46768216	32.3011116	20.86021683	57.24998618	13.9479446	39.45643459
AZD6738	4.599314489	6.519073729	6.684195306	5.131380987	13.4405597	10.50111626	8.136612797	9.527706	9.228730902	5.084009712	7.85423423	4.753773181	8.047983769	9.415319664
AZD5363	41.73243988	16.34530447	36.67270312	30.00526351	12.51361738	21.28699615	16.35653743	19.28683006	30.66395072	15.47357914	16.00146446	21.72596158	18.67920599	34.66575252
AZD3759	16.03153145	12.52857852	17.52543511	16.35518829	19.98624297	11.67643751	22.72412961	10.15624467	17.90826015	18.70796024	17.3507871	11.9950395	9.317019711	16.15594291
AGI-5198	109.9631438	89.28143965	149.3907964	97.07838985	106.5726112	124.1866511	105.2578776	107.2818138	129.8531193	92.10024852	108.5463411	87.90721574	72.54807766	125.795578
Afuresertib	28.66256951	12.58358964	21.93192304	24.4912848	6.362097373	15.16156577	9.251243367	21.58416212	15.05593169	11.97875014	10.1376953	9.42042187	12.00966813	16.02782307
Dactinomycin	0.006611822	0.005652239	0.019880131	0.006377718	0.012686518	0.011117544	0.007609798	0.005267986	0.009786847	0.007407268	0.015317511	0.009844992	0.003283664	0.011821123
ABT737	11.51062644	7.960082321	28.06727316	15.21693812	5.238290627	13.01217031	26.62362378	24.0952611	3.80518226	9.482894694	11.35772282	3.571726528	3.482107125	8.313304758
Venetoclax	8.164982911	7.480391328	12.97536802	9.076271656	8.343097534	8.343877746	12.04454249	10.5521369	5.756603032	10.26595647	7.629282074	11.63017128	5.237361226	11.22316308
Ulixertinib	15.0948492	7.687106217	24.29476249	12.16424332	34.23716737	30.03585049	15.29470541	11.34534619	14.86429655	17.84436402	11.71156058	15.20596271	9.091206446	15.939123
Buparlisib	2.658184816	2.290580924	3.152556148	1.76860426	2.584293432	2.922231107	2.718275494	2.666483552	2.246699843	2.681130304	2.810798001	2.657867846	1.886465237	3.323701807
BDP-00009066	9.301237954	9.968703205	13.67279835	11.18043379	11.3377169	13.09705787	7.029059378	9.546955733	13.48481693	9.345824078	9.946483733	11.13906329	8.627551179	11.4509056
MG-132	0.25479661	0.224184126	0.270861203	0.218879111	0.213879374	0.190878683	0.185268476	0.175106924	0.23608539	0.31954354	0.258545881	0.182967495	0.170619468	0.19274931
KRAS (G12C) Inhib	61.83257769	75.16914502	206.1458872	56.26561323	100.642847	109.1709768	48.98092351	109.4657162	92.04636356	84.02016121	100.6831921	16.99917896	73.96318325	65.31072402
MN-64	107.6469455	88.47617132	134.8505022	110.1257975	105.2308959	118.5171879	79.73867959	93.17615101	134.8652082	104.5778816	106.3215805	51.80090689	103.3157513	157.7786529
OF-1	65.82177855	49.63218733	96.13143834	38.30356516	75.70053516	78.40812454	47.73759238	65.83943362	51.51274902	73.04349125	70.22212808	29.79233717	57.39848858	91.46425485
LY2109761	174.7099332	126.9441837	211.3741478	155.0847545	179.7491239	176.56092	128.5305695	196.9614343	156.1875813	132.9788417	169.1077107	112.6054748	146.907461	153.4048524
Sabutoclax	0.363982627	0.863467724	0.927909102	0.420149935	0.89120916	0.787287945	0.513320287	0.709266841	0.389725044	0.417252331	1.21230933	0.367774371	0.540398778	0.481072509
Sinularin	53.25494712	32.64253504	41.15019388	37.67666489	23.40937801	52.85832669	26.64679654	37.52965896	41.36156758	38.03316328	36.27151925	21.0130224	41.25158923	43.8350554
Elephantin	24.45145263	33.18079858	62.19344215	43.31768153	28.00986852	42.34302442	20.31015071	24.01117319	50.26687057	39.78301323	50.32467622	16.91020219	36.51808764	47.04804467
Gallibiscoquinazole	10.66367066	12.10369403	16.9062209	12.76982907	13.17245974	12.36949763	12.0642397	12.92981574	14.12599284	14.38836446	18.50759836	9.036859638	15.34135235	17.28848292
Dihydrorotenone	2.331396793	2.074279355	3.564991421	1.46157667	2.839686266	3.227211902	2.108454991	3.602009991	1.704385418	3.458468701	2.372348909	1.349491063	3.520661719	2.373401366
Podophyllotoxin br	0.598217815	0.454775568	0.605359671	0.45619109	0.402963109	0.450275428	0.362494513	0.261159056	0.675118122	0.539347081	0.599403036	0.228331898	0.540836716	0.891866214
Docetaxel	0.044826503	0.073125523	0.096309226	0.091025655	0.020249784	0.058168477	0.062774652	0.042888244	0.174075988	0.061657716	0.205716049	0.034357132	0.076991847	0.329319191
Vincristine	0.154473376	0.145870316	0.286032345	0.203020618	0.075376046	0.144536424	0.05692509	0.070132595	0.275696803	0.145059744	0.323132762	0.049816819	0.115440598	0.258526445
Fulvestrant	60.86100245	81.80117354	124.4902251	95.07576476	76.63998119	99.25421094	86.13904313	108.1648957	89.10572161	77.65483795	113.7385025	43.54526726	105.3956896	95.09027005
Nelarabine	601.4709362	356.4502483	385.5355641	605.7044837	332.3839107	415.3359176	330.6141609	314.1591913	463.863776	471.5253303	438.3687175	291.6670421	391.9545655	565.6112672
Fludarabine	231.5559718	152.0911288	124.3584524	189.3663351	178.0220728	181.4768393	134.9152717	114.0398356	220.2600253	152.9662114	180.2161172	215.2119223	83.79267174	218.3267782
Dactinomycin	0.064332136	0.072859092	0.117574406	0.082466008	0.107065425	0.113352399	0.073145624	0.056397031	0.146744085	0.08991981	0.128745801	0.052114637	0.055978075	0.106287138
Mitoxantrone	1.773785582	1.466537136	1.740904545	1.939650953	5.141234061	2.174995994	2.61142445	1.294188126	1.583646872	0.950169965	3.512735136	2.904058692	0.74316475	2.664459176
Teniposide	0.846290079	1.401512514	1.935424343	2.08834674	4.69119195	1.564016443	3.43955189	1.296963969	2.268597754	0.840404493	2.928091486	1.807642096	1.258669577	3.255678264
Topotecan	0.60130712	0.950810787	1.706008146	1.091953533	5.612965631	1.296241417	2.140883669	1.136044726	0.660478896	0.683297044	2.036609478	1.543064886	0.528169001	1.042855101
Carmustine	571.2969754	412.9753728	617.5201633	497.4160893	471.8892318	489.6419712	337.1776046	392.6090259	452.4535847	593.6451218	592.3984421	207.1291438	338.4603265	520.1937244
Oxaliplatin	150.2251798	99.61318751	234.3113698	171.8994739	212.8666264	223.8499768	166.8630979	87.83613522	236.4407638	111.9348277	178.2020141	136.9840668	66.71833136	255.7640216
Acetalax	226.3209219	88.25173439	227.5498607	263.716752	39.44153579	66.20838638	54.1967895	120.6086553	186.5188352	68.43567872	220.2397276	103.962292	133.7776978	144.1577207
Zoledronate	41.81417506	34.48316979	66.36905904	41.7174693	34.62119476	49.13179001	42.56269068	43.56458854	43.87821841	42.75201928	45.06884721	20.03964848	32.49927102	45.52103301
Ibrutinib	91.77625219	76.75673396	177.3043746	111.369399	85.01713178	102.0448828	75.0670828	91.48444279	184.6212318	59.85403812	83.60604286	22.80611789	78.77642527	81.53757974
AZD4547	14.33206615	17.90087862	19.45565101	15.70851347	11.32343102	15.75978498	20.59755164	19.3456017	15.65882424	9.958965522	21.47470784	16.01259174	14.96253618	22.72670134
JAK_8517	28.86751058	22.10076366	7.312267216	26.29744336	14.23849916	13.15120972	10.6378168	14.54250897	27.71434059	10.96621691	13.82192518	15.90305755	13.49680458	27.81341167
IGF1R_3801	7.773812434	5.900094211	2.481683843	9.193779482	2.925158892	4.812468703	2.778487598	5.39126406	5.763736098	4.141388393	4.967430189	2.809922977	5.885402784	6.836569564
Selumetinib	99.42894947	25.51659899	34.46205692	126.9256584	157.7271421	124.2567151	56.29063	29.07149848	76.12560378	96.78064237	12.03947253	165.2456404	29.41016502	72.77407142
VSP34_8731	8.106570203	9.605821584	16.4144295	19.91905328	9.131057808	12.02778249	13.81024059	9.975643668	13.22105028	7.151501514	8.186382301	13.33472925	7.931821444	16.12427763
ULK1_4989	10.02812716	15.71187643	8.4910642	34.55470729	6.186124289	8.767190925	3.607851718	7.119687564	12.53956375	5.248773463	11.80997004	22.16364978	10.51373977	8.456731973
TAF1_5496	44.79541499	43.19332414	70.92084082	38.62582198	45.16549145	72.74126693	48.64766874	69.95008604	25.28876523	49.05857768	42.66674717	17.13138563	48.08075609	37.5816348
PAK_5339	11.07067619	9.384243691	16.44195333	13.21765381	11.43781305	10.48420658	8.058443701	10.99574416	14.74317648	9.591267909	9.83112326	8.951551961	8.728638554	9.771866499
AZD5991	44.46829155	53.98233556	115.1336344	82.58529196	4.188590082	66.17374238	54.92651535	88.64151749	25.19879308	89.90344998	78.57742286	26.28102152	76.53939719	115.5020902
JAK1_8709	72.4537682	47.79780314	51.52546773	83.92633421	59.21917216	72.35412526	88.400468	65.83444532	39.99088611	85.54703619	45.87534778	85.16829	47.04992358	75.93170894
IRAK4_4710	123.5099804	144.9824506	138.5272422	160.5960429	125.3127959	170.6992884	99.39552247	142.172654	121.1691615	139.9051125	135.1900159	141.4858414	131.151725	192.8008046
ERK_6604	39.0297326	18.00377153	17.15724087	37.36940882	55.16065523	52.79597423	19.5936942	14.86717813	31.99365943	63.186598	14.92553082	50.17680936	23.35309706	27.46941419
ERK_2440	22.63858625	9.646785067	6.814038044	19.84306594	19.42791244	23.84330692	10.29202593	5.901335981	19.43935823	21.77854531	7.243120011	28.58242284	9.805224043	22.6258274
Eg5_9814	0.028858443	0.03655008	0.028528096	0.046329595	0.01448376	0.036768926	0.036213669	0.043678359	0.054306096	0.020073966	0.054429786	0.045649709	0.042289347	0.070023838
CDK9_5038	0.127016886	0.076978651	0.10204304	0.19213339	0.066256575	0.076042361	0.045262883	0.0715525	0.084903883	0.084392517	0.068044831	0.11078126	0.06685497	0.112654298
CDK9_5576	0.762261101	0.524786814	0.732298409	0.87852145	0.572378895	0.658760094	0.449625149	0.422167411	0.747693822	0.615796393	0.501001859	0.743179862	0.412225261	0.928732866
AZD5153	6.32170932	4.149622709	7.041638189	11.28130237	2.764283833	5.697199502	4.935909075	4.866297897	3.539391739	11.81550168	4.548836564	4.11881725	5.177143206	7.152217066
Picolinici-acid	147.7345759	145.1769938	249.9298635	165.3068702	187.0781757	201.9729782	154.9881279	144.919711	215.4385817	197.345698	151.93094	137.5400106	148.2285361	204.4900507
AGI-6780	49.032314	63.08631113	82.68873171	55.11367188	57.88041246	62.92155093	53.36944602	56.80626362	46.05380587	59.09542067	64.11899659	33.0129984	64.13136168	66.91268238
Ribociclib	39.9954254	34.99934532	66.02545674	42.34966742	60.41285091	49.29407846	39.17465743	40.05028634	53.80406322	41.91001877	37.5478283	53.39576219	41.71762113	36.17812078
PRT062607	27.45349454	18.75471503	32.20357354	35.01382149	24.89569925	32.27947884	22.54430277	23.99817268	33.03624477	23.42939099	16.09344008	29.24260122	22.74169763	23.32584217
Entospletinib	43.18241638	31.79481197	41.9243641	43.71245175	36.95774393	34.6065188	27.86625453	24.30660579	71.4210616	28.69861305	27.56533418	80.71694674	40.89678419	65.41499117
ML323	57.94087289	71.21653506	179.0678139	83.22098238	98.15112156	119.0366473	75.33448641	91.04800129	65.49945571	87.21382338	90.16639994	38.39642296	99.47658438	99.75166085
GSK343	18.03917388	13.77027669	19.93329168	20.99194206	15.11206443	17.36916718	18.02725594	13.5521094	18.85583026	18.26460876	15.2519474	14.40277227	17.10857138	18.64505771
OTX015	14.55025707	9.010619283	10.59010901	36.10239143	5.591307075	11.66841562	15.53205865	8.857184785	8.969546096	25.69745198	8.060187832	13.58011117	11.45531675	15.17510823
RVX-208	131.2499637	95.04477384	123.8817576	174.624472	101.233185	115.6929415	133.4173237	85.14904432	127.2759403	154.357268	107.7376703	121.2468133	109.832094	146.3521969
I-BET-762	29.07738918	22.65390217	35.89225414	52.46385112	19.37840333	29.31347695	36.33034996	21.60341889	28.57556216	45.58191257	24.16976992	27.71939125	24.70095729	33.77435566

TARGET-40-PAMHYN-01A	TARGET-40-PASEBY-01A	TARGET-40-PANMIG-01A	TARGET-40-PAKFVX-01A	TARGET-40-PASNZV-01A	TARGET-40-0A4I4M-01A	TARGET-40-PAMYYJ-01A	TARGET-40-PAVECB-01A	TARGET-40-0A4I42-01A	TARGET-40-PASYUK-01A	TARGET-40-PALECC-01A	TARGET-40-PAMLKS-01A	TARGET-40-PANSEN-01A	TARGET-40-PALHRL-01A	TARGET-40-PANZHX-01A
13.7901607	10.4217637	9.166760779	25.9459726	6.133336854	10.03505806	13.74094011	11.44548182	8.824693775	4.173182586	16.65075657	59.68125757	8.972288719	37.56683187	15.61838487
0.957307023	1.604561515	1.634212195	2.880282169	1.545718562	1.188880715	1.87744566	1.405803167	1.250254635	0.472879023	2.518437916	2.086200208	1.045142541	1.753958571	1.528351025
34.70653454	54.89450289	31.78742121	42.12669249	39.01248833	34.2050641	48.8574318	61.77968339	87.20288723	20.5262653	37.13220578	18.11022023	38.63369488	27.17393865	28.63213553
17.93706667	24.24740948	21.93735349	20.2622036	22.05479467	28.02899766	28.13609858	35.03545208	27.30383942	15.88748551	21.52531569	46.31304261	31.03327774	19.66392635	20.13758871
56.89204914	62.94830786	145.7525746	57.05745948	64.77715958	75.27837103	77.59731877	240.9763286	66.2710846	37.8308874	77.2488743	36.62660512	77.45767223	36.38955485	57.40964063
78.60440027	125.1720133	108.95054	72.64180018	83.94233176	141.4792073	111.1902764	178.0399178	143.8000172	69.77746604	102.2865196	63.45692391	109.6100194	65.82329739	82.14165912
13.01343147	13.7283994	11.11377158	9.553741435	10.5692534	13.76752268	10.30811059	16.66527684	15.88416236	8.852674158	11.33347986	6.589887676	12.26588115	12.39844253	9.978389905
148.4816366	176.2418095	202.2961481	156.3110666	107.6155226	166.5628427	178.5092304	271.1771436	228.2500777	105.0648348	215.7487994	107.6142878	153.9476497	178.4869992	114.2365499
9.920385002	25.69436349	14.33763161	21.75320102	25.63170016	25.14516617	25.90468876	35.0239212	32.57584054	11.68104834	18.31440599	7.987546607	13.59260569	8.679485773	20.60430562
14.76719018	16.79690901	28.85268735	6.040958709	15.14795181	16.8864162	20.74788931	24.19518158	21.60964238	14.71836606	16.56582207	3.771462977	23.58207028	14.49288654	11.56029969
0.03533177	0.116932689	0.102693159	0.019291288	0.031111171	0.110577653	0.066439217	0.201478321	0.20255506	0.018218529	0.067480505	0.009994183	0.100100002	0.011399184	0.025007728
9.488479361	14.55003288	17.08268754	8.225429005	8.441667648	11.08241289	9.936470361	13.7518672	12.25220266	8.736773187	14.39919306	2.664413306	12.37444579	7.883764131	8.60304812
12.61716109	28.21595398	54.01202619	26.59680395	37.49814149	26.8925636	46.75943899	53.33178939	29.97899425	10.23973201	32.56872509	14.33256746	23.35397495	21.55177315	20.24166453
72.99354175	156.4504038	144.8231835	173.4612759	108.7547721	151.6801362	166.7070218	170.3094981	190.0323384	64.31311515	183.0847705	162.6800513	114.0482569	163.5285905	113.5034798
31.21669296	42.06316855	34.37324418	31.3865877	30.47029646	48.07983501	27.30606503	55.29300487	31.36438179	24.80370679	43.42064116	29.76466486	28.2643908	26.01325588	32.67649896
108.1149926	218.6719192	190.3855564	147.0961275	111.9945886	231.4364337	160.1193693	207.9504341	187.6954315	108.3695984	211.9260711	119.4239217	147.9521212	164.3433762	159.586008
1.246816433	3.151546458	3.462857344	2.16746324	2.527925469	4.488793676	2.298706085	4.344677115	4.84115104	2.50076265	3.632297492	1.221232437	1.877640193	2.167552611	2.334068235
79.22128558	133.5244585	92.92993077	109.1703243	88.33073988	144.5071683	99.15692665	146.3924888	161.928185	55.99464159	103.3645231	80.36551811	100.7374259	88.21631606	81.62712603
35.21492853	41.51487676	41.87085867	51.61586669	21.5433435	40.05157121	37.85631876	79.11694764	42.81838139	24.85047815	57.89788502	19.32680421	46.17755054	37.67350648	30.57414617
58.2968932	89.58008261	65.77105882	45.26287089	31.24422432	66.45936455	69.22872742	99.06917614	69.60852251	36.41349495	68.08356366	36.76688294	77.63883868	44.23439172	48.81423585
0.032318432	0.03090785	0.017517963	0.011680857	0.01032005	0.019486802	0.016524205	0.02136808	0.013768322	0.02480059	0.015392251	0.007002411	0.022436583	0.008124011	0.018180906
42.97377577	35.15831143	51.55275852	47.3020945	34.9189806	41.68848422	37.06756406	45.11094111	63.03966299	38.86724429	45.15856936	28.81774613	37.94686492	45.3112917	40.36760643
14.7612098	19.27391885	15.18241494	16.79560189	11.95835983	22.6541792	19.78461568	19.88561887	12.79237902	21.07325898	28.7397882	15.98549642	13.82858132	29.79799729	18.92060072
10.35770704	17.6802499	15.25843983	11.46672693	8.938847429	18.68908132	16.65661826	20.88079968	25.12237528	11.85178327	22.17640841	13.10790587	11.88776327	15.51026837	11.08238064
69.50710923	156.9087629	116.9193411	77.68373803	54.80895913	158.3712347	78.34719489	230.3689139	134.0030753	95.11460162	197.5533402	41.75487224	82.89686514	101.5247338	88.33860636
13.81235819	20.87836334	19.04902406	10.59296598	12.09321566	34.67468039	10.84778173	37.97917445	18.13837376	16.41288156	25.23749272	7.278069266	15.43192012	23.54026069	19.98611239
230.5061313	313.5553189	299.9948295	211.117466	198.9377082	370.506581	199.931651	447.025385	324.4223933	161.2004005	317.5321172	145.8895365	242.4845555	291.8452715	204.830236
1.928325913	2.759926394	1.835862284	1.061808436	1.345949507	3.876713633	1.502505738	3.784845384	2.166341535	1.677022238	2.0544312	0.860336916	2.585395605	1.398264934	1.543106681
80.30103828	116.3660846	108.644039	85.33943467	61.54429632	125.3865924	65.05232641	200.1030322	103.3214547	63.73922062	102.9763855	37.43094343	78.66803032	85.83749902	66.50761434
125.8841985	182.6183152	153.7049204	121.3675253	123.3731392	163.6207935	128.5706075	203.9364216	202.5693898	106.5386458	180.9351712	86.67308852	136.0230564	102.9162241	130.2052923
1.378903895	1.546560785	2.285968141	1.409019862	1.265596407	1.650409894	1.60775615	2.767604411	1.540116745	1.316882691	1.753422737	0.825992972	2.933848331	1.360122149	1.177182838
116.2451389	173.7332504	158.3135518	95.075162	106.304123	167.6914113	117.9286644	250.6905035	197.0525054	100.7603167	229.9810709	58.00763286	153.2931663	98.77498123	128.7560914
21.99906006	35.1923097	34.45669121	45.87401749	30.52711552	59.24711787	29.92536055	90.35784927	46.38298206	25.62223439	40.20279228	24.37382151	30.87540551	37.6227839	31.53221567
5.626677201	8.594127541	7.628648878	8.372062858	7.628392933	14.49040385	8.242694714	16.0814859	11.87401798	8.485734786	10.24528565	10.86690214	8.00491252	11.38803354	9.036098318
4.985487251	4.528724547	6.821793092	6.184788412	4.025694533	6.819189491	5.714692705	9.898892731	7.820794865	4.33521728	7.897953231	5.727204197	4.42237597	8.847143819	7.561684049
16.79844172	22.14596233	23.16110418	27.18935188	20.94823135	36.72963764	27.02879811	33.1320424	26.93783419	16.67876787	29.5429508	42.22332884	32.88836088	36.63843839	29.07930851
5.874699788	6.178271021	20.50139919	6.033321563	8.273505742	9.809736703	12.88028231	27.25433255	8.938628013	6.478962142	9.511108413	2.669425765	14.10476929	5.317911847	8.418317512
11.97102244	26.907946	13.51662434	22.9258686	22.58314491	32.31655838	15.337418	31.2178495	27.28648705	12.88090023	20.85652271	16.86370139	20.15920399	17.83532742	18.70812297
8.480721774	14.70513087	14.07043818	11.08998555	11.83239886	20.56969648	18.41298489	23.84544257	24.6621586	9.802843506	17.03555285	12.61616634	12.68157664	16.53332853	12.85340793
85.78234994	104.2722368	129.0541059	93.93851007	81.68232724	124.2700934	101.6787656	172.2649633	145.4042759	72.5267416	109.1436115	78.79415196	124.5165946	114.130794	86.0607755
9.495409566	20.26296325	9.199677033	16.8830096	12.24107394	22.39421534	12.59357249	22.24116789	18.65655601	8.623747412	17.07884364	8.75168083	14.5588987	13.88989268	13.15546754
0.006172604	0.012790909	0.010233506	0.003450129	0.005379793	0.013152673	0.006760746	0.020557352	0.018642832	0.006199014	0.009415858	0.003152059	0.013806023	0.005638665	0.005024378
2.368879152	6.554068741	9.546585222	23.50932785	3.598845973	11.219125	16.09587065	12.7924287	5.662966047	4.953590067	20.26669664	4.51327961	6.2215208	23.5819422	8.639823152
5.172581826	9.421696806	9.960190605	9.420936399	4.55408396	12.17342141	9.153494199	13.60639897	11.31158046	4.532405837	11.05998333	8.227962656	14.49967661	9.403257185	8.306042875
14.52821589	13.4390546	28.78813529	9.525156439	12.81026656	17.60264118	20.67815966	24.51730045	23.78548581	14.32202857	19.89316605	6.062370338	23.47555272	17.25502765	12.77015011
2.101118607	2.939367229	3.173340952	2.005282441	1.773167784	3.555828029	2.476094053	4.484164989	3.792421179	2.385916266	3.596643013	1.282562905	3.468429936	2.195514654	2.182880243
13.53819119	16.65419394	13.17612299	10.32303589	10.19849773	15.72406397	12.36665897	20.79377292	13.76397115	6.641837027	10.69062365	8.720763978	16.38770433	11.97104009	9.96127261
0.162497256	0.20422515	0.199331494	0.229686428	0.168513578	0.296071725	0.216521925	0.196004302	0.308843123	0.207718106	0.221297389	0.166801472	0.174659821	0.18650279	0.178712175
89.17321083	93.59552899	130.9859259	98.75152876	65.88298153	95.35697311	72.47066156	224.0617537	125.4415517	40.57761562	154.3953491	34.71644407	115.7524915	105.0598407	93.89824414
97.69107083	128.8589768	107.925353	181.8770964	104.8492161	122.9286245	108.2577707	136.854097	216.9781828	64.76764017	178.1917769	81.88326403	73.72235356	192.5450964	94.71226468
41.14841536	79.61272904	82.50059356	55.18273095	40.87378937	70.29623798	48.29424971	104.721889	94.48385543	36.15612201	73.43827677	36.98953721	65.23696741	73.76373357	50.59140705
154.1405456	185.2863018	179.3139265	211.4396686	128.2377223	213.7950789	125.8071753	329.7012877	235.8327626	119.5839018	227.1506487	237.5999115	161.8395666	382.3950352	198.7507416
0.693155963	0.998169663	0.814841448	0.638592604	0.618163152	1.016504551	0.642778055	1.128128381	1.041336444	0.472169261	0.84930233	0.391761832	0.729804995	0.61243326	0.7960333
48.17424373	52.25131762	40.75078878	40.26890038	28.70695418	42.56867388	29.30927697	59.66328483	49.22125981	27.71004475	48.93147629	24.26440037	39.24239036	32.55494314	32.63992746
36.29753859	61.4027902	28.12924628	40.73512359	40.6789846	37.92762602	27.40686659	49.54600436	61.52519682	21.62265878	60.51488479	14.66448395	39.64909346	23.48748816	30.71993108
12.65745045	22.7110916	17.34491647	12.10251069	9.5740013	18.5476528	14.57533823	24.47529768	25.80723174	9.967891799	16.35355099	8.509203272	18.49448662	9.944812635	12.33385695
2.47250499	3.673889598	4.762117117	2.150322849	1.358349103	2.94617975	1.448466612	5.308096137	3.114624694	2.189444414	2.834819587	1.154380115	3.158581288	1.590813403	2.308839838
0.347342263	0.853418468	0.735387763	0.385124155	0.533149402	0.667397863	0.707563335	0.479730247	1.761718657	0.236846327	0.754513252	0.187615837	0.570090824	0.345172607	0.38596139
0.062713388	0.228278036	0.177989885	0.035854285	0.060551406	0.507744023	0.169980683	0.354536081	2.701647868	0.029376835	0.26103541	0.009025154	0.218142636	0.024230218	0.069311419
0.143725619	0.406386031	0.424296103	0.099831524	0.139067449	0.344802828	0.28383243	0.318372007	0.986162693	0.035613444	0.355405763	0.037731807	0.24279521	0.109265676	0.116601074
74.61037061	126.1460711	144.7072862	80.07386483	88.48087461	110.7184698	83.02417999	204.8828383	128.6607172	65.546283	136.8789315	34.49533224	95.5597512	57.53176614	95.91466823
379.4751956	563.2003575	462.9581415	505.6071746	302.8950595	519.8428945	426.6401265	669.7379598	641.073048	223.7781609	636.1971236	511.3699462	453.7600518	627.2531191	352.8466357
198.2330219	174.4756438	264.9870271	197.202323	135.7900026	183.6704084	170.1526676	197.9364096	272.0447854	46.39949	177.3684943	149.2998556	179.2232284	200.3907626	104.170132
0.088816911	0.144153273	0.102465261	0.044843762	0.075490118	0.134258525	0.077460917	0.162597771	0.170124332	0.036661553	0.093179734	0.034082333	0.127492118	0.049226046	0.053254547
1.306028701	1.318141561	1.976988292	1.709892127	1.047351625	2.518937506	1.769556487	1.982907227	3.458218812	0.326785424	1.503789787	1.095929517	2.205045428	1.648441188	1.000835098
2.155539792	1.81205211	2.509856964	0.704700773	1.091732069	2.32558405	1.309515999	3.364986027	7.251796182	0.443189958	1.108475267	0.74522875	3.302766032	0.803606958	0.54700572
1.086757366	1.329183013	1.479791746	0.912936777	0.605809931	1.868508948	1.290307081	1.467388145	2.304404543	0.394617393	1.058932539	0.417032083	3.450582857	0.60292943	0.700125688
448.9851998	636.1403202	522.4240448	455.075159	316.0858442	651.4237197	435.4674209	947.1078927	594.5501476	299.9792055	584.9760807	355.2759931	545.9663586	593.3026089	407.2863623
159.2012225	183.037975	143.0377048	78.41247061	113.769955	185.5866279	186.854611	261.5400614	290.6595405	53.37252425	107.3645253	70.08231429	369.659178	108.1885671	95.74778828
74.85272446	155.3363066	106.2695515	301.0299452	210.643867	272.3486343	234.4777552	88.11631869	307.6697387	135.4118893	246.3489663	198.3024007	77.50833112	197.1366749	208.707965
43.62611122	59.24254908	51.78382625	50.59735748	32.53511389	48.65608686	45.95614263	83.73937115	58.01202001	26.32748289	59.89293012	46.03254744	48.04766015	55.98441754	38.22370165
69.16623645	115.7899869	85.72195008	113.3667958	73.6769132	116.0652608	75.34382024	182.6629853	153.1786572	45.31934234	108.9165096	63.94961542	61.6747096	132.2744772	117.8333325
11.30729613	32.11158556	22.41264891	18.81845174	12.14879703	31.65674859	15.44492008	33.05041222	33.05165026	15.40415733	22.56895896	13.89868138	19.75768498	19.30158852	14.55121071
22.59315869	30.43078429	16.51813214	36.53853341	18.33199285	45.29521029	14.79309679	25.24861025	64.53823155	8.126885068	44.46384023	23.13917186	20.41084287	37.12459996	23.72850691
6.97138903	8.864240771	4.983326006	6.821262716	5.529636818	10.73472029	4.682776298	5.944396491	23.4635198	2.889035395	12.24875288	2.121307459	5.294625025	7.437622793	6.752921277
64.93870915	75.47238139	67.15026441	23.73545102	48.4618762	56.00443435	115.0761438	44.95002632	97.84428381	45.46199982	59.52465814	100.6549951	95.29743866	123.4396649	48.61900326
17.02135883	15.5988888	11.90728559	8.902876833	11.32424431	10.9702406	12.05543744	22.40962026	14.88361811	7.823448739	12.35523687	7.06706127	13.65231421	8.565248043	11.28092976
27.51072781	26.27825978	8.456108573	7.425520492	9.663546608	17.31525025	11.51740598	13.53497374	13.43915131	7.015141044	11.99221549	11.88212414	9.820417092	6.658949712	11.95013047
46.48488035	67.4184389	74.9713832	38.30737562	27.39853987	68.3234491	32.53085984	93.67988418	45.36228796	38.1117613	59.65035927	24.13525452	61.54957102	40.42064179	48.97600765
12.73106901	11.73074392	14.42136545	11.05181787	10.32190086	12.51384762	10.70316333	17.4886344	13.20377366	7.749774315	12.87214455	7.959079793	10.27288052	12.1865242	13.46725332
84.59822956	193.5412597	144.0601541	95.06375659	32.55668151	188.7431305	78.1666788	228.5025908	88.13921312	41.35504532	247.3147555	44.30776148	55.3217059	83.49074708	84.02806009
62.45229734	75.56599183	67.73538884	61.46589304	37.82474448	87.60393799	41.51858749	94.76356076	98.66119318	30.78319979	103.3359392	63.60955943	63.05555711	73.5253077	43.60978452
160.8104421	239.3992331	141.6057261	145.3630695	103.645733	214.942716	86.98880504	191.929438	246.0262974	99.00258767	156.4852773	109.0695278	142.4433213	118.068076	113.1741852
42.35315205	40.47977508	32.39367896	16.69071077	27.70422983	39.89325666	29.43397831	17.40786909	57.9336225	27.72814907	30.73006707	28.58699482	46.92258254	33.62566195	22.05078845
20.20090972	21.718972	12.19490668	7.50789375	16.64806815	17.40674531	10.53657157	6.15241287	35.27170887	12.50281115	18.00133192	12.64014071	13.17109454	17.52185738	9.940809692
0.042523978	0.08534475	0.088375505	0.035787134	0.036892889	0.081649186	0.051985253	0.113292732	0.173356884	0.023005528	0.085572695	0.016320156	0.050433378	0.023815135	0.034182892
0.162859558	0.173796444	0.117141378	0.137490757	0.083234876	0.190727137	0.105341292	0.206378522	0.218090256	0.043320969	0.110883531	0.111128874	0.186050111	0.149745339	0.089878504
1.084317974	0.905634291	0.86003169	0.824680605	0.542071338	0.940300043	0.654924101	1.157920864	1.191258501	0.3700348	0.810327604	0.681525927	1.168037851	0.935024982	0.689230102
8.263959535	6.4010308	6.360212953	4.762510362	4.007501745	7.23814641	3.38050795	5.841962251	8.917549185	3.280302626	9.272963029	1.099637191	4.3103318	6.587474132	5.084812461
133.2596839	193.5693396	205.1002888	169.5058323	142.1903083	184.947053	192.0386684	261.8828248	217.7592252	100.772546	203.7594007	152.0775805	192.5835853	196.8700736	135.4856381
46.36646941	86.38464843	76.87981473	60.96996232	46.52830432	77.33490132	73.90997597	89.14208662	92.67129869	41.93740962	85.43349251	33.40346604	72.2233292	49.32008882	58.55487451
34.95131016	35.04101105	57.82623595	47.40219859	39.10272438	37.60242	44.12376221	71.08340743	39.7724365	25.8724017	40.44596208	62.36775	47.22617913	39.51586657	35.69916562
26.47374021	21.98044312	23.66387717	22.94449061	24.47230578	33.74598749	23.12142283	45.60925039	22.64317767	14.37841676	24.97439064	21.30082717	31.21319105	34.26422538	24.73256739
34.82044775	44.19510816	41.9001835	38.3875027	39.69660959	48.44917755	32.37739851	51.84207717	72.30162839	18.39726084	42.85671669	44.3272274	36.91715043	34.2558206	32.31296521
79.3292232	115.3900348	148.9564857	73.7780407	65.91588996	107.0656823	76.87539202	183.529171	115.7466396	58.71457966	124.9547474	35.40778138	99.35617334	67.52518501	81.68172311
14.99654857	19.06569767	17.19726147	16.92040772	13.62329554	18.57291804	17.10739502	20.21728229	21.32522315	10.14342421	19.59136016	14.98151778	15.14214702	19.93283657	14.85311157
13.6083505	13.58287822	9.507114959	9.210254365	9.457633244	16.59477554	8.847446286	12.08464546	21.64673004	6.662579837	17.40924834	5.11073722	9.924643487	14.42121485	11.06650616
111.9123168	128.5892036	129.229446	132.2779514	84.96544095	129.2268486	105.7224886	138.3293253	164.4000103	56.78844736	148.7054477	101.0324831	121.1393524	151.2726383	94.24644667
25.45830386	30.75359488	30.20697221	26.13339251	21.59916974	36.18200971	25.92384846	36.75158276	43.74948216	15.78838059	38.02258536	10.67761524	23.59302768	31.72911769	23.19387133

TARGET-40-PALKDP-01A	TARGET-40-0A4I65-01A	TARGET-40-0A4I6O-01A	TARGET-40-PALFYN-01A	TARGET-40-PATPBS-01A	TARGET-40-PAPXGT-01A	TARGET-40-0A4HLD-01A	TARGET-40-PAUYTT-01A	TARGET-40-0A4I0W-01A	TARGET-40-PARFTG-01A	TARGET-40-0A4HX8-01A	TARGET-40-PAUTYB-01A	TARGET-40-PATMXR-01A	TARGET-40-PAPKWD-01A	TARGET-40-PAPNVD-01A
5.203789399	10.31476653	11.1124286	11.49558654	7.324623597	14.04655526	4.780517169	12.54435067	8.108380799	12.91639929	2.760182935	12.88877839	5.942731531	22.6055915	3.022944134
3.275571094	2.524363407	1.040781493	0.96503535	1.305942246	1.852958476	0.731043136	2.593650759	1.687255012	2.196174458	1.06543801	1.630456285	1.892904039	3.001561938	0.323581301
28.48165319	63.83096455	49.10752587	45.37980885	23.75070644	47.20877886	44.50705474	67.95109122	52.41979073	38.50610578	50.30996081	28.45291609	41.28903822	46.98755716	53.05678005
23.43748581	30.05923499	25.37680615	22.68187236	18.98249707	21.92696219	21.9652742	23.49156229	25.60639957	21.41542742	26.91315883	25.55193016	24.09869829	35.75103962	24.1132254
68.69267444	62.42958449	114.6471353	102.2601662	40.45662476	78.44694338	50.31433888	138.5685383	186.2149582	59.87720961	58.87552419	36.55232775	32.28406825	48.7069563	59.88354339
128.705213	140.8953842	125.3504807	151.8687909	67.7260874	127.6046469	69.21612448	109.1345409	188.1523638	94.9958311	190.6523405	70.8178735	84.20232176	101.4349299	117.7295409
9.561632175	12.07709424	12.09186974	15.56882034	11.14968585	14.57180542	12.75942174	23.53668584	14.10794614	10.63374209	9.973467707	8.155649028	9.324845373	9.526095608	15.11843566
190.4060281	190.0762215	176.7198069	240.1457242	119.3012536	199.403347	181.1620905	297.6266725	279.8399466	173.2695244	197.1695528	104.0520203	134.1814749	164.483001	149.9583188
29.20251553	21.04997645	14.32188978	33.14082228	14.89452118	14.21288504	10.02403611	29.253025	15.55432043	22.12371032	6.970962883	8.762317509	10.4574345	29.19575942	13.52015553
32.3202943	43.28147414	26.02886949	25.67426406	18.75554006	18.44599209	35.57809422	52.61989577	27.31017607	8.629619009	26.16949516	5.106977356	18.62936349	10.59448236	10.91726878
0.047989187	0.073216781	0.093437505	0.04853648	0.045678617	0.206635823	0.035038901	0.344413044	0.145296783	0.080484717	0.065853195	0.014432346	0.063736272	0.044669182	0.047885986
11.95298521	18.02719628	16.28121985	17.25421912	9.78111319	10.58940526	18.90104759	21.97679343	16.43060945	7.367837245	9.128970405	5.064352025	9.512148748	4.373294321	7.99021843
50.57837763	24.07947412	38.97759461	27.16208127	25.69675868	32.46071286	10.46325686	39.92917616	36.16904105	51.27292622	13.36503612	23.94152225	24.73398822	21.1858355	38.07775386
221.9455776	166.8110922	151.0483262	149.868319	117.2238134	97.70300694	112.3062571	213.1243895	140.0323575	149.5136547	103.8099472	90.97057514	104.4252126	177.151305	188.9581714
44.02890597	25.28605587	31.63237165	45.75962023	25.77532701	52.04983089	16.86635548	33.50964447	44.66059308	45.83797295	24.90548795	23.26603796	33.23624098	34.1716185	22.85753137
150.5614176	185.5276657	230.2285978	217.459496	133.3969782	209.140983	118.1422991	256.4018131	212.2858685	184.9419643	97.19025101	117.3655457	111.6978084	114.133444	162.0943752
2.95761269	2.975374723	2.666764076	2.739174021	2.812598295	4.132299292	3.42605829	3.907739767	3.839341122	2.119741072	3.147030866	1.491152277	2.276846468	3.399932545	5.026790217
93.09116832	116.4477092	144.06372	113.4062313	85.99720243	137.5468173	88.15306952	147.6341828	124.8699891	137.3516141	92.7976807	85.04994233	94.45302652	107.8319796	155.7364674
31.72439094	27.52190189	92.21565539	49.97703656	29.80580008	40.40444904	43.74524502	43.13160413	90.65415811	67.37648429	8.599710025	27.31325512	20.4760017	14.74583974	10.7141349
44.4585283	81.43056461	80.66977861	86.21219573	44.64156519	85.35974687	53.32954388	82.95758468	76.56198134	62.65199042	40.27491338	35.54272825	34.27169273	39.95570106	39.30215574
0.012215559	0.02073443	0.022558557	0.018617852	0.01467109	0.027477926	0.022482703	0.02327889	0.024947605	0.017815136	0.009925671	0.009546041	0.009421805	0.002907095	0.008990527
41.59310854	39.85752272	56.005695	47.67654721	42.60391931	41.44559747	40.51861904	74.0598005	37.15255521	48.04088741	34.18767016	34.47802502	32.53397506	28.95314652	33.34556626
8.709355479	14.7951938	18.4512249	28.36129546	12.76915754	24.84730787	20.89764169	15.66999899	19.25319567	14.80574444	9.093516525	16.73399069	11.89198909	9.727384652	15.99649522
13.36474504	15.89113723	7.120598289	21.49680814	17.06065603	18.79177213	16.03154935	12.84754077	16.50171689	20.39002284	14.81814058	10.89212217	9.619360792	18.24837145	22.00358609
119.0143871	130.1913667	117.2476768	145.3105972	64.17944154	121.2354439	84.75941727	163.5616656	254.7331301	118.7008963	67.91966256	52.86884845	57.16309516	60.08806254	81.59164419
7.053779018	12.34861694	25.94144005	26.21947706	14.31230093	21.09782415	15.07508612	38.69659978	21.39766213	20.90082832	9.770811256	13.10911793	8.801239507	9.226515019	15.46705037
207.6487994	234.7359314	324.2152459	343.9927708	219.3096003	310.2856891	189.3465455	367.9459923	375.179645	329.2973309	184.829164	211.1480699	171.9763417	183.3716397	249.1483767
2.255791886	2.589417121	2.237327586	3.313779634	1.257807316	2.542978507	1.591484342	3.389867102	3.203806017	1.53099679	1.457654906	0.969543549	1.281207907	1.267661066	1.534757464
46.35778799	62.40879466	108.1968271	156.5638836	70.40939992	86.39035357	68.66534441	196.0128444	157.0191236	100.8587572	79.46528254	59.20983981	43.05468756	57.70620009	58.80466208
133.7605348	153.2151439	204.7314369	170.2823737	117.2370576	150.1460008	95.73019868	240.2161285	184.2899655	164.9579395	139.6166372	135.9661389	106.3826577	123.0621495	151.9052975
3.102411907	1.389121872	3.417857873	1.554873638	1.718496449	1.283481071	2.813733456	3.134967756	2.446217269	1.070275694	1.74894899	0.935745801	1.47886288	1.515072843	1.469208463
133.4909345	184.7362111	182.9680634	183.4282102	139.3534286	190.3433301	113.15897	205.9284115	267.341602	180.7549953	137.6288975	120.1280735	127.6185655	98.17878076	149.4354268
34.02153215	24.54316626	31.42937907	54.97744866	24.19525796	24.13803089	33.97253679	84.46992258	51.14155264	43.98751873	17.91822212	28.80359733	20.59797524	44.30548023	37.20437945
7.619446903	8.504669398	11.26818557	11.59733246	6.600066791	9.703090126	13.15585285	14.21131236	11.57142357	9.953693358	6.857908899	9.809364878	9.180567108	10.5290993	10.34562261
4.632922634	6.336440383	8.843166183	8.856723986	4.573560289	5.986564139	5.369232557	11.37211447	10.78807395	8.489040073	4.123686007	7.321680221	4.796019119	5.464766688	3.527645973
27.18922677	23.81408474	26.16546932	28.76743637	18.74253767	12.65315246	42.61403274	59.69491448	28.8347267	27.33467491	24.34000011	22.55941294	12.78846025	40.50428555	50.31561699
17.85403694	8.126940865	25.91154729	12.5251629	7.36250761	9.139398267	11.31193902	18.78231905	15.01137119	7.384047091	6.57510307	4.146945499	5.201897689	5.042685725	6.073399412
22.64726046	14.16121053	17.66034773	25.18772307	11.89434286	16.59906016	17.62902802	50.56650405	17.5601774	30.91119631	8.822740408	18.07928729	16.04922534	35.85521137	28.93922192
12.99035801	23.19990023	13.60264796	20.3209541	10.31234393	16.50952412	16.20391934	21.8276031	21.0675262	16.03709923	18.42223871	12.81171368	12.893419	16.09578059	15.53579331
107.2654912	126.0802592	117.7224259	116.1250982	99.10093648	97.39492345	102.0641177	175.1893682	141.6396092	110.6834695	114.7369575	88.67228369	85.22826379	90.58383593	106.5712001
11.89826646	9.886273602	9.202450533	21.1477684	9.974462664	12.59913759	11.84455801	26.42488084	9.214871994	20.87183102	4.5202272	12.7040284	9.04582377	18.87397074	11.56199531
0.012616244	0.015839469	0.011689692	0.013587919	0.007751748	0.022721613	0.015910338	0.023156784	0.015761407	0.008039573	0.019131163	0.003665585	0.008817984	0.006082312	0.00973092
8.06941964	4.05437262	17.13610512	15.98419026	7.429851377	3.701189141	22.31699086	18.07455664	19.40761689	10.1639554	2.520020134	3.432467334	2.966650345	4.493261496	2.83687134
6.215840846	6.841122723	13.17565014	13.80717292	7.239606532	7.077629243	10.29671999	10.32093544	13.19960073	8.031966869	5.475818956	4.286182008	6.790145879	10.01489846	8.416182168
30.47388077	33.60749117	33.99245901	24.1170156	17.94961183	16.18845014	37.0935732	72.98500369	28.14379677	9.117765838	24.43621512	8.677641526	21.14702566	8.037666532	5.93985873
2.700981183	2.772667254	4.009948643	2.912189605	2.469058919	2.929601656	3.425039975	6.435117184	3.504956467	2.249097747	2.789372656	1.994211928	2.267598975	2.404778131	2.403962509
11.10870366	11.55348134	16.29751827	13.68146916	7.378831984	13.82337902	10.87677197	24.67848077	17.52839052	11.67204655	10.83563934	6.368931814	9.70595222	10.4154915	9.440788712
0.246496643	0.246778008	0.150223095	0.201492199	0.151632615	0.214358988	0.214752168	0.248398927	0.238232973	0.201587898	0.174792926	0.15838662	0.259864162	0.219297974	0.196414061
128.9221553	123.8470841	145.6964668	168.9529031	63.3090455	88.73053366	61.33068803	170.7629844	206.0767218	81.89342092	61.02936103	42.33561406	77.12419289	51.7997138	34.45306882
114.2718391	124.8496578	127.9006355	192.8102593	103.1173796	106.1323942	94.11463613	173.777747	154.8518919	173.2849983	72.25450465	112.9920984	125.7081176	143.3840131	117.18287
46.19879593	80.7129431	88.01642202	100.3403725	64.39662809	72.64915266	80.18861004	96.83935581	96.6914162	65.7154067	45.05565161	60.6551396	47.98009341	39.11366185	56.90010921
127.9102178	154.542399	250.1387695	314.5795444	139.6318416	191.2434254	144.212443	216.7462117	251.4028787	205.2010227	90.20116682	190.707056	110.908899	176.4786893	245.930942
1.300084809	1.18854467	0.515064334	0.838415958	1.111352649	1.010792006	0.387117258	0.873740634	0.968727491	0.450453476	0.46851662	0.408380251	0.391128503	0.729392546	0.579803694
26.9284507	32.31447363	55.5632281	61.99173268	30.35850293	51.17721108	27.75173766	43.13699806	55.44853118	53.27088523	24.79554938	35.19934013	35.20711998	27.71897742	48.86769174
91.54630365	42.33226868	46.88219994	31.2615334	24.32221487	48.68725687	21.29053473	55.10635635	50.15327599	48.76752639	16.54708755	19.10147012	40.67407536	26.88168343	38.07021816
10.62162091	16.44694612	17.47973947	18.67806322	12.93421674	18.82781612	12.5018733	13.57152104	19.88868648	15.77124804	9.652781463	11.88725194	14.46335663	17.30391716	21.87106449
1.928046148	2.744022868	3.557508047	3.5540837	3.415531378	3.354373782	2.280633145	3.560873861	4.700061453	2.543158317	1.525742076	2.122085842	2.628357775	1.030857929	1.42975355
0.465329636	0.648165839	0.656690388	0.483976605	0.566898021	1.136092376	0.366570937	1.688404886	0.575096435	0.704179784	0.495563774	0.41728134	1.056818822	0.557310768	0.99523417
0.067907404	0.177060361	0.375816877	0.117993431	0.126517214	0.883464894	0.043398853	0.651647493	0.240740442	0.129985828	0.197009747	0.043039596	0.319053841	0.077251417	0.195520206
0.094420823	0.185196069	0.247822502	0.165146927	0.171198811	0.549029058	0.062168676	1.806908314	0.296100789	0.356855545	0.13433664	0.098767603	0.346383157	0.185700605	0.199489841
97.08684355	104.7448664	133.5660178	130.4279816	114.7005789	128.4405684	50.58335453	143.0248856	129.5205721	109.8318019	67.13554893	78.78671938	96.00877173	70.89084805	68.29755705
333.3766135	408.4483	511.2687655	612.7943821	347.5417859	502.958893	310.5815232	502.6983375	452.7392504	544.5440975	291.1541159	386.1570681	443.1707676	476.6542334	775.4736896
160.5486805	112.2365263	144.7299742	200.2282553	87.17918155	113.2077998	180.3467852	433.904286	160.556122	140.2008891	203.5707158	78.95658948	167.3673515	256.8086964	298.6292734
0.115887515	0.124863091	0.090415419	0.086084685	0.073563856	0.184839247	0.061189759	0.181150942	0.100504052	0.085345484	0.104370487	0.064274256	0.096972241	0.092144565	0.153202767
2.640683728	2.537120581	1.525070571	1.23072788	1.828672803	2.462880807	1.021656204	2.626390753	1.663881077	1.488643253	2.556140373	1.062246031	2.287970112	3.858903823	2.088827874
2.418865663	2.889961257	2.085752568	1.617505494	1.826783494	4.083018244	1.058821851	3.531047041	2.181037752	1.468422573	1.769730927	0.721023936	2.987297978	1.728501022	1.541639959
1.505529765	2.248076739	1.723184776	1.309233681	1.024584141	2.637253736	0.762299714	0.88328919	2.639628771	1.226532233	1.020369567	0.275045346	1.484094156	1.33403878	0.441790911
326.9890551	507.8304459	620.3541826	775.1172361	367.6029997	740.5815407	342.2164499	665.6899562	669.8923311	415.1142514	308.1516636	443.2470959	408.4295315	346.3518475	483.5817984
260.5148743	185.019931	208.8642173	193.4046049	127.2263313	301.3913119	87.55445687	336.7475349	168.4646575	125.9869801	115.8138807	86.32315438	222.3751999	157.7276643	308.187414
123.2035036	301.7963749	153.6799909	233.9730162	145.9641469	151.9626411	140.4756429	549.0822091	182.0282726	121.0928916	102.6547495	167.2747723	118.9583571	62.94245411	120.9287169
37.56302165	44.81752492	67.94399699	66.30182086	39.52965485	53.72004884	38.23707626	64.14736749	62.59071007	49.95453898	24.63553304	35.3420506	33.04321834	42.04888661	51.26540672
66.46747193	178.5433318	137.0714268	180.3209283	75.93610057	151.734424	67.92355231	155.5280611	177.034628	184.5448707	49.39929746	90.56369259	120.5151166	111.7636713	59.44011396
12.50020768	19.22963241	24.78808119	25.08019867	19.99407171	37.09599704	22.54729566	25.49822817	28.25227568	25.4300828	13.51269181	15.667588	14.63640381	31.90210883	26.58390152
19.91503749	15.36638942	32.90480053	21.9886977	16.40727186	69.72191419	8.539655565	29.5721329	20.78560872	29.33001795	8.16675279	15.78135177	25.97524913	41.42842014	42.806926
4.280950084	4.786850395	9.016279712	5.454339521	6.507208513	14.61905253	4.096947507	5.888427743	3.339425984	6.182380572	1.475396992	4.575403267	7.734449019	6.143128433	10.04282642
101.0278044	115.8116056	119.3897083	92.55382928	55.37665804	76.53912988	116.0492515	172.6381246	50.6651353	70.48249154	76.33530169	24.75432468	139.8250574	78.57198319	80.6726374
12.51103204	16.68775306	12.85525644	20.60561574	8.273881562	16.37213263	7.798111074	14.85387147	16.41963974	13.51239666	10.69161328	4.518134217	9.395486674	12.61383169	15.84282418
4.100299072	13.96463166	7.903424109	14.18973938	6.076242541	26.54210939	9.074906179	18.38251345	12.48171026	24.13874991	8.891640764	6.681579552	10.09814747	14.49095488	17.77644224
32.06289155	54.9298086	74.59931339	84.44962085	54.88445908	74.91498978	33.2530746	104.076616	90.2666947	50.68618058	28.76990424	38.56065093	32.11286856	16.87959069	21.56959832
11.15515993	11.48040941	13.74832189	16.63504332	10.1954269	11.0095664	9.748057654	20.61799594	14.82442421	13.96366785	8.65651296	8.066319668	11.68418648	9.373048527	10.32353364
20.27779694	130.6086845	88.22425645	377.024017	57.72785337	122.3080427	68.47207414	178.1646313	245.8914691	129.5395282	41.01530471	46.90258776	37.33533757	63.02608815	96.12872027
60.64296261	54.07361812	101.4293862	87.73126756	51.58688096	108.5987015	40.74369681	111.2906018	87.14724241	80.76243198	51.63005032	26.63401775	70.8503825	105.8779505	111.5508684
114.2242382	130.5353148	183.8328801	209.4129007	109.326448	195.2914077	126.9317512	204.7242058	173.8901298	185.5391407	108.1935291	85.1210874	140.4914043	181.3451314	194.6519238
30.08147453	35.66496695	50.48256531	34.5857082	31.80659488	40.50034728	64.51065431	81.78634881	22.45350487	24.85946593	31.9487996	14.39782935	57.39306959	31.18737106	37.30261941
19.27727573	18.0579807	16.54134088	15.24179724	12.8570907	26.10415863	27.06022131	26.38443711	8.476518518	13.66057626	13.69887592	7.61577318	31.18517288	16.55986779	28.90070381
0.014895603	0.042780528	0.103594299	0.062927643	0.063724499	0.103052449	0.023878846	0.115292183	0.067707525	0.080925648	0.048518306	0.017599919	0.084433545	0.0696515	0.071890613
0.139868843	0.091966247	0.147117336	0.179824032	0.099007991	0.203935507	0.056825568	0.211619787	0.160705972	0.136988981	0.049241529	0.04030819	0.090851592	0.103856484	0.123435991
0.789832878	0.647657304	1.086921237	0.989212442	0.516100513	1.137782127	0.556218034	1.29999578	0.900458569	0.679113354	0.512462636	0.299040701	0.578418609	0.824736931	0.715851361
3.409145352	8.029229844	6.076803947	6.942243404	7.998122829	10.98223336	8.39914429	11.10752328	4.088000511	4.296559724	5.322161746	2.946055256	9.554263441	2.559469133	3.293233055
181.0566359	199.4954207	202.39452	203.9131883	148.413997	199.7009052	151.8790007	243.15614	224.2051466	194.5865674	118.5425816	134.4272812	156.6142085	200.1016622	180.1835549
54.92876121	100.0472477	63.58556138	83.17648538	65.88378084	99.96944926	62.14811988	95.53125601	78.21858397	66.61709425	40.14601286	50.72604389	57.49663283	48.84726884	55.80860873
62.90919089	52.29551599	49.51903247	33.33979766	38.51317656	35.46131206	31.28137047	65.8878368	59.23809813	40.87867794	49.87328862	40.25157963	41.17812486	60.38777136	29.37043434
31.43900342	21.15582155	40.78880711	25.25921565	23.80171101	24.76634353	13.84016501	49.25067963	35.19806962	26.74786771	20.73417788	16.65072187	20.10507068	27.28539827	23.39167392
43.2100491	45.43801966	51.52397766	37.17829061	34.10210702	42.96740922	46.38538857	76.83276918	52.0137107	51.55394802	57.42962105	32.26295258	48.80351451	63.68095737	105.7060539
107.9995791	109.6213422	125.6440054	111.1089408	112.9726801	144.8010868	75.52433201	183.8947443	140.9850952	104.3196766	55.29169223	71.56175273	78.58388281	54.73239348	59.08475883
14.20752845	18.32564987	17.03338591	19.41568722	14.85228679	20.70551326	13.44845932	25.33411006	17.4775575	21.06097945	14.78575939	15.24443177	15.39748669	18.60322472	22.67558688
4.792909679	17.30904023	12.87288231	13.51063246	13.31981486	25.32256729	17.28597282	34.762621	9.388158991	9.437010036	18.032699	10.24552582	17.79441894	6.990623212	12.68237164
78.01809173	112.5437134	121.9020557	108.3499047	101.598003	165.948956	120.6590417	230.4829124	116.256975	137.3933318	114.5904746	106.579131	127.6001561	104.668919	163.5691524
20.00397017	37.62435942	22.95539294	27.85604751	25.33499717	43.04598061	44.28527986	68.32447238	29.95066013	23.97848948	39.4287952	24.33615278	36.59064181	16.46068537	26.8364615

TARGET-40-PATMPU-01A	TARGET-40-PAPIJR-01A	TARGET-40-PAMEKS-01A	TARGET-40-PAVDTY-01A	TARGET-40-PANGPE-01A	TARGET-40-PAMJXS-01A	TARGET-40-PAKZZK-01A	TARGET-40-PALWWX-01A	TARGET-40-PASKZZ-01A	TARGET-40-0A4HXS-01A	TARGET-40-PATEEM-01A	TARGET-40-0A4I0Q-01A	TARGET-40-PALKGN-01A	TARGET-40-PASRNE-01A	TARGET-40-PARGTM-01A
3.636147114	11.1868032	8.485051392	15.93240232	9.007304956	15.2052045	6.818950696	19.65306539	8.389951284	13.3984342	5.291896533	13.36400687	23.99308813	5.871856574	14.87855321
1.881608315	1.095376087	2.757517373	1.955946183	1.632169423	2.304733251	1.228462608	2.644895267	1.275315851	1.28206163	2.231624097	0.959108269	1.447125485	1.511798927	2.858667114
16.42873695	35.48182843	10.97396897	32.38915214	40.49914762	67.68644186	28.19708641	49.4921295	36.17049266	25.09323401	34.89803031	34.56529228	20.65397646	25.1961753	29.41892073
19.16293401	26.01106768	23.33722569	25.29513804	19.94980567	19.66146118	20.42211237	28.66797661	23.01854987	27.26590114	18.70123591	20.26394306	30.68310586	17.09733703	16.06779765
21.52402081	44.89613148	61.95412165	39.76278894	89.05807591	42.71756293	32.57958671	64.42734054	64.66870639	48.48742184	85.68197412	97.37451393	53.87655256	30.01957731	45.75863998
81.72902317	102.0795768	70.33800856	97.54973857	126.8706423	94.20839662	69.65744691	93.28331033	85.82056538	75.38647607	90.50242785	67.62017968	184.1692295	79.07132441	100.4009367
8.508302095	9.964409068	7.570124378	10.02847937	10.54387038	14.7289681	14.27287334	12.68498507	9.825047518	9.635366597	11.20181731	9.874321325	11.19613744	8.921795435	9.66717297
129.4048599	141.2131638	107.6037235	140.912217	181.429114	219.2154199	119.3952165	216.472362	137.2842045	123.4920428	180.7597201	141.3916829	167.9853573	111.7069207	140.705494
6.548539176	15.3008835	7.38050023	21.5205154	13.9079767	19.60963273	11.78337336	52.3808184	11.33457685	15.31971635	26.95049902	9.042235213	15.84059854	13.29343644	9.280456673
13.75135716	8.09861663	20.93412909	11.16147781	21.26249384	19.4722671	19.74498217	26.07214431	16.7498321	14.42762519	34.78744715	16.72852771	8.995682085	12.91003227	8.180993165
0.027093732	0.034223272	0.013222377	0.046488327	0.058426052	0.041737129	0.032317477	0.017247953	0.045187261	0.020517483	0.082216632	0.020276266	0.031601004	0.026332194	0.03801703
5.600806468	5.554469089	10.25896349	8.444212002	11.93035533	11.11915758	11.80090294	13.42558949	10.54075768	6.500609876	8.758276938	10.62530205	7.161344181	5.840444516	7.559330343
9.645075597	10.70817494	31.18630994	13.39579336	22.02451629	11.99706471	22.30911786	22.70039652	22.18636744	14.3408619	50.02565156	39.28090385	16.90459205	12.61622694	8.871765155
81.98303656	127.0062388	96.26713458	101.622902	131.6097548	135.2899851	111.8017674	158.6124075	102.5971131	94.84143012	141.3245453	126.8390152	152.3816637	101.0505352	114.2169455
15.18308333	28.34505292	16.33104609	25.42327294	34.52513805	30.9757961	23.73459247	28.67866456	27.1802351	23.53260569	32.31631618	27.84890875	35.94059083	17.23075499	30.64784413
83.15173239	167.6731683	116.2976928	122.8770859	189.1261943	137.414131	98.85802811	145.7112423	133.6328193	151.306122	176.9727726	156.9233129	203.3577376	101.656006	118.0803795
1.662369425	2.148897947	3.741692315	2.046527719	2.438408874	2.943235127	2.526530325	3.849734524	1.879528262	2.284393402	4.580441958	1.716580152	1.732753503	2.910525695	2.13974052
96.07168396	72.4977984	57.24674909	75.45821788	105.9480237	82.08060412	84.29539845	66.55468352	80.504624	55.68418546	159.300283	81.61961619	119.833833	69.97024166	71.28968243
19.61397193	52.09281904	33.41891769	38.53347532	44.786663	19.66957776	29.76206849	48.56140805	32.42120944	23.83512899	39.63994507	58.14026849	45.83993991	13.59094418	40.63758589
22.25484977	49.12198794	42.33865441	52.87060771	44.62702163	41.7719536	38.22356797	44.24164728	54.45040302	49.28020775	66.11799153	60.68694381	62.6584437	31.88020257	47.30858181
0.006760716	0.020522076	0.007460928	0.01497543	0.021490129	0.013017533	0.02398911	0.007488195	0.023585194	0.021897536	0.011888355	0.031850223	0.014698113	0.008488791	0.015601958
27.74593429	39.04216583	32.48441768	40.77036118	38.66773685	45.04777319	31.46614959	45.83137182	41.07968974	42.65106335	55.35717252	48.98948118	31.82194845	40.45152043	48.17473524
5.932808397	17.4013763	9.228734408	11.99113323	16.40933917	11.04822068	16.26931928	17.10322526	13.24624107	18.18831425	16.24665502	21.1792992	30.1269742	9.555642114	12.98837663
8.686058056	13.50338546	11.89333271	10.1893771	10.39271083	13.64705276	9.115871111	7.241238094	13.24699853	18.87224351	19.49236811	13.05029929	24.95632537	9.456049901	10.47437891
32.68899751	122.2657029	72.36059579	66.17786188	151.3853267	86.9367072	75.08694654	47.77730009	102.317616	123.1354961	88.26725127	164.6545397	91.19419179	56.1082234	74.53208464
6.368366046	16.22159792	9.872672708	13.41642268	16.71204019	14.07526189	14.08744764	28.31191375	12.46114303	16.41156129	41.4522857	14.08576696	20.64077386	12.29107155	10.28944346
121.34266	262.7770795	120.6718786	156.1591992	279.5000302	214.9663814	209.083677	212.8831771	201.5759188	177.7994014	246.2652876	284.2003068	244.3973359	183.922921	211.3256146
0.92774603	1.447911906	1.015219874	1.345368887	2.181078295	1.548117542	1.86878869	1.230766403	1.312417486	1.480715877	2.295868025	1.910442132	1.579086696	0.908747281	1.200146447
34.412567	84.11964272	67.31926393	103.746238	113.5734394	68.15587336	66.7028704	79.18180011	92.44532801	59.92740036	95.11939567	83.60389667	131.4060341	68.31899926	81.4347788
94.66114632	157.5086879	117.7320591	130.9101503	148.9221914	117.8326451	119.0396953	131.6704121	137.9184194	110.1731543	145.1159812	151.5501408	141.3682171	115.7339473	129.2238214
1.208985452	1.907612935	1.609982204	1.105317929	1.659792269	1.529811124	1.821218902	3.71525133	2.26648047	1.305026735	2.209980372	1.390355943	1.193282914	1.40536412	1.074396652
90.91213658	144.9419378	128.5471046	110.2783846	151.2635751	133.7763624	149.3034503	117.3849456	157.9136283	129.1220311	112.5419157	167.7976234	158.1276413	138.077708	101.6071004
10.94614108	42.05528799	13.8189909	49.87999032	35.45782731	29.72985621	27.12481462	58.39881168	31.15626043	32.64114255	34.52070593	24.31767979	47.77190142	23.63878142	18.01290847
5.279643152	9.726559175	6.011322588	6.61826434	7.362317638	7.147005008	8.480352996	10.94966897	5.990878525	7.341937849	9.051028935	6.838992588	8.624330405	6.133142424	7.177475011
3.159810272	5.63917184	4.308165929	6.026367785	5.656873822	4.841503642	4.662433213	7.789922867	5.685459831	4.801536845	7.126955087	6.49911689	6.77967057	4.274689614	6.619221504
11.2944978	26.35437252	14.93545526	22.80782143	21.01130024	18.34235468	20.58601881	54.11392863	16.59066845	19.55961678	39.17803689	16.67929597	30.79449518	22.50624374	13.28446891
3.405889009	4.327321773	9.012895266	3.840127601	10.53540033	3.930187464	6.729466475	8.501734411	7.428227817	5.841486359	21.0170941	12.03525399	4.661842457	4.390592088	4.347230246
10.38978428	22.18951028	9.152958291	26.4301954	14.57823491	15.77522628	17.384987	32.15353038	13.30803524	20.74995725	37.71977132	10.79198888	18.60374988	13.55647331	10.28806283
11.41488788	14.3648188	8.642386946	12.91418856	12.98391501	15.02956616	14.01706084	15.87184903	11.12183497	10.93908792	20.88874681	13.74375808	16.87426862	11.09007819	15.02951166
76.08623728	95.04526428	96.35768859	97.97649249	108.8854952	101.5564592	84.04709176	100.5707675	110.5435465	81.80303307	108.4656157	123.0471408	101.0157749	98.05839685	99.93411896
4.714208716	16.93509457	6.742382394	22.5020945	11.52102858	12.47865629	10.63560484	28.65102531	8.327569276	11.2474413	17.6319512	6.794697981	15.20577475	9.603178496	8.783160371
0.004169363	0.006364736	0.004549679	0.004084753	0.009389398	0.009009216	0.007400733	0.006650202	0.008289648	0.007280207	0.025590139	0.00585668	0.004050174	0.005311541	0.004920624
2.339521426	7.418366013	9.199423272	8.46315958	9.479007198	6.711606512	5.519054695	33.68385264	6.455497447	10.31752787	16.30084468	14.03028103	20.892993	4.789741575	11.94617535
5.278678402	10.38768537	11.39822529	9.254974143	9.035419027	7.053586975	5.573515369	12.63723131	8.628056979	9.868640792	8.439528828	10.29255364	12.78558325	7.586654391	10.84331349
9.502789273	5.866028535	23.7693375	13.7827517	19.21822004	14.37754751	12.92925659	24.11045908	18.34282199	15.5963149	27.22556217	19.78133862	11.03068142	14.38863262	9.914858115
1.758747892	2.808765944	1.836035988	2.559312609	2.596420889	2.294999036	2.4070259	2.807909173	2.726753575	2.127089631	3.505116522	2.081292204	2.050327852	2.304843729	2.079331739
7.898427659	8.12315895	7.275201052	8.069425086	13.95432846	9.087593083	9.125417015	9.019507825	10.19160983	7.659138217	14.35719017	10.70162502	12.08353818	6.672424666	6.895242612
0.193430192	0.200103259	0.125910502	0.135428403	0.188090695	0.217624659	0.234259982	0.157195119	0.156009124	0.150994806	0.223354202	0.177080854	0.155732653	0.174084788	0.203844052
33.26552718	94.57239457	111.8102665	108.6750623	158.9639942	75.97792634	50.71589001	119.1638223	108.2716448	48.55039921	51.68984911	96.83278342	111.5161689	47.91129437	81.11198777
82.3813592	131.0047633	89.09395215	147.5763844	126.3836907	115.3616948	78.89523356	82.66264087	93.91945543	83.7652216	119.7952038	143.514684	148.1866753	102.0870799	130.0097777
42.53072758	83.55242095	57.92440169	61.45794093	64.83199097	63.36593323	42.80052545	57.32023487	75.72190541	68.60670404	46.85725708	78.32161814	62.12854451	58.42725495	56.93867754
106.5678111	206.7150006	189.9552774	175.0427654	177.3574575	148.3480505	114.4624397	212.1329017	157.1011592	176.969168	175.2426813	245.4555709	259.5794336	133.4475986	137.4839153
0.55703584	0.691580805	0.956033923	0.820888135	0.835314387	0.722433602	0.682888589	0.611517799	0.584326278	0.507663381	0.843348128	0.55966668	0.734578247	0.586156726	0.748786591
27.14284202	40.71067444	28.78234262	34.41204227	44.56484524	37.27974985	32.1233796	30.28519606	34.19186112	22.55205864	37.59228068	45.97204139	45.31122258	32.50157966	31.73678124
31.32199317	30.22920124	26.35761877	32.00033666	51.60115957	30.80081388	43.31263499	26.98674438	23.33844632	16.49009862	51.30725409	33.76604865	19.34339617	28.48625638	26.71158348
13.45043525	15.41670931	10.71720732	10.89138103	13.3078569	13.27400396	11.92826215	10.6433806	13.42159297	9.516254227	14.19320934	13.95304639	20.77735465	9.467214078	13.5960168
2.443364052	3.527485637	3.426609168	2.835024994	3.139482975	2.493740458	2.063847512	1.707760312	4.491917752	1.981039463	1.557102597	3.235036228	1.788363989	2.779060207	2.709072907
0.722592558	0.396232713	0.248882963	0.542284316	0.61214327	0.574907763	0.554015548	0.290769225	0.411228544	0.30891732	0.64782382	0.448638342	0.333307314	0.559192469	0.488289066
0.125104226	0.059808246	0.03016228	0.228967182	0.125988129	0.07858892	0.062968883	0.024843197	0.10778029	0.018568567	0.452314731	0.033571836	0.116146918	0.083788334	0.191675297
0.179089704	0.084374455	0.042315865	0.340923748	0.24809948	0.158891252	0.151726246	0.056762556	0.13899738	0.088758217	0.262425155	0.113102159	0.124540227	0.102390225	0.176532584
77.7391267	85.3235101	132.2019755	115.6035279	118.7985332	94.23802648	93.70829315	90.01216259	90.76038867	63.70923652	103.8351537	92.40222772	84.14591497	105.0822298	128.877416
220.7548177	504.7078693	239.0274732	386.8759401	411.8325516	463.2681996	351.6918696	439.1672153	343.689973	245.9777735	503.9910115	453.9906833	652.6162299	287.1826316	410.9127503
96.67397975	141.8640248	116.3080108	112.3690335	167.9249079	190.7425587	73.6179601	174.0556583	91.30440415	125.9393316	228.5776812	184.5532058	227.7241434	96.16895836	223.6237205
0.058065204	0.072856108	0.068571795	0.091551071	0.112922389	0.095906649	0.088726065	0.056581896	0.096683532	0.053771516	0.12310688	0.077862524	0.093902395	0.088515437	0.073045161
1.101247188	1.172510198	2.191060365	2.246336482	1.451083007	2.099484543	1.567619144	1.561374603	1.361265533	1.272772865	3.377447388	1.297628994	1.588295472	1.690921576	3.87603276
1.19477885	1.381825872	1.532711496	1.625492615	1.434004173	1.841936499	1.030734097	1.378998246	1.855022131	0.774140179	2.781005847	1.310820977	1.363161675	1.144127313	5.385966007
0.750518021	0.797754598	1.431237834	1.242215505	1.143571403	0.895905069	0.986669945	0.817164366	0.918920816	0.821807378	1.078641658	0.949647145	0.705189566	0.851138755	1.974496953
252.1346353	602.5531072	345.8782534	451.5401509	503.3215048	459.6131892	411.0685368	382.0319898	444.8553664	268.325785	440.0400958	537.3575182	682.1919397	333.7506014	394.0522549
115.3917194	112.2461298	222.3740721	141.6872819	168.2627523	164.870419	156.220005	156.9149996	132.956796	60.55326094	505.4445691	148.009466	162.6371719	154.9702063	172.4226337
48.4715912	214.4453783	93.24094091	84.07818457	275.726311	189.2908135	82.85377195	178.8477019	111.754816	25.50417448	360.5792855	136.4489352	120.8980374	285.7071013	167.4585918
27.60748703	47.69724557	31.87274459	41.80233657	50.98710664	43.21554407	31.17517703	39.20287591	44.34489238	35.42440727	45.24317106	56.39247851	57.2635458	29.346973	46.34308294
56.57540551	97.53296418	113.7251772	123.8759015	97.26663026	80.99266931	81.42794987	79.39821755	104.4762033	50.66892294	145.8963535	107.4884577	95.39185389	64.15793606	90.15245352
10.63962071	22.57892201	14.41608523	20.5542822	16.31802376	15.84442613	12.6603874	10.01974363	13.75258223	12.60967817	18.00574871	15.96939236	14.41819565	21.73865588	25.10577294
20.87987483	21.30454681	12.90976305	15.45635353	16.01114979	18.87660403	14.99329245	16.07394584	11.70320831	8.791742175	70.30937296	14.43737214	27.21079355	14.01492059	25.13895301
6.402958871	6.158712851	4.025005525	6.201189655	4.623509557	6.390791353	4.743687098	7.242706324	3.13908792	1.661280335	14.77491495	3.467762319	6.678967616	6.181542383	9.439035653
56.66654873	15.05619763	129.4670344	41.24997798	63.32732724	53.19017116	62.9338316	89.49189429	29.60557999	49.55723088	300.294251	94.35137966	38.63132443	36.20190914	32.59825326
8.589961863	9.732916284	10.57164773	8.444253075	12.51717439	13.48829694	11.3778884	13.27725289	8.057404527	5.413541748	14.79013073	10.26103745	13.63762718	6.329900548	9.483736896
4.797762583	10.40911766	4.685117796	11.72382524	9.749298231	9.444276306	8.917878219	5.443780162	8.425265318	6.46654462	15.55382295	7.994156429	11.19723426	7.085979658	8.892451953
31.63352997	68.81938194	54.78906591	54.19432557	64.43305444	46.83767878	39.10201101	45.96805647	71.87462203	52.53833369	37.73215013	58.58127052	40.03469164	53.89151777	47.84304805
7.589896231	9.955566994	11.72176799	12.78158864	13.19807306	12.26351728	9.127350824	13.55295589	11.28364993	8.521477865	10.76271043	11.41773553	11.15071727	9.045410513	10.22008506
27.61627215	130.9160513	25.76244108	111.26409	139.9141243	87.24108098	33.05025754	40.22502568	66.36022339	51.30045167	92.26437349	74.07466022	171.0705872	58.46597136	65.34946872
55.20613114	65.25671571	77.93413282	56.50726511	70.9319217	60.98610909	46.83916809	64.63120442	42.99600667	45.67368828	111.2422568	81.21396499	43.16270245	51.64630752	96.15906724
160.0862229	176.2217162	142.6732062	144.0254668	138.3451359	154.1440988	123.893413	88.05499087	129.2687566	99.42365401	165.2684989	154.6283447	135.7056525	128.7548961	122.0222074
51.08149381	15.21404063	32.65301485	21.53781816	21.98212005	25.71587662	41.97705901	32.89944086	18.71875972	23.64363832	65.30611316	37.11450409	16.18676849	23.58774658	21.51889024
26.8449607	6.954300776	12.4563694	11.3905379	14.92291484	11.43250193	17.01289686	12.77967898	6.4069766	11.20113391	53.13461526	15.10277984	6.285408699	15.67196896	11.2255247
0.069916307	0.042506827	0.028030955	0.056429354	0.044481465	0.038170319	0.050633368	0.039237735	0.047524768	0.021314723	0.107144368	0.036054564	0.052729135	0.036898275	0.050690556
0.068494908	0.072664907	0.063654796	0.109190332	0.138397789	0.097080498	0.077776157	0.103016505	0.055878142	0.038535926	0.11866949	0.082195302	0.105399563	0.050856672	0.091192561
0.580197006	0.616075127	0.50318693	0.629540433	0.891859234	0.724114719	0.543209745	0.787568378	0.445404169	0.334871937	0.924472338	0.687770021	0.701779954	0.408169877	0.529981919
3.43076734	5.660555541	4.53916527	6.965656699	7.225101702	7.738366892	8.486586123	7.342945505	4.958683822	1.998227811	8.069774206	3.821535993	3.919798338	7.961546502	6.788810899
141.6229014	159.6638403	140.625355	174.3513782	192.8273478	176.9549779	142.7535949	158.8568503	155.9028289	133.3979308	204.9313607	173.3071921	168.8417184	127.2587004	162.2363631
50.27046703	72.36638004	82.86093321	56.7024563	75.27422412	71.87942908	53.81647272	60.27550379	63.28425486	41.38481745	75.86655961	61.58341749	69.0607642	53.02342349	81.39326604
39.55856215	36.98585161	62.95469432	49.41640642	45.43461579	38.92071987	41.99801786	51.10507664	49.10892907	46.16800909	38.38558509	41.22806236	41.53291371	41.22017864	34.57742591
16.77422924	16.23499911	35.30828521	24.81204726	26.24307963	23.52264886	23.76284263	37.33463299	20.00116781	18.71904357	29.18054578	29.18054578	32.56874488	18.94741691	25.3692904
35.90826863	34.90229713	36.23122114	40.98327864	43.68650696	35.34618721	30.37100927	30.55720455	30.56622527	31.27158528	74.87685289	40.71355569	31.46454361	36.55771595	37.47984171
64.85298539	97.75615351	95.44146261	96.08843541	129.3865458	87.84559037	68.89037229	60.8445351	131.164698	89.8540875	81.01131309	111.7918821	77.79208507	84.80822685	104.4139166
12.79976561	15.46896383	12.59783015	16.30716343	18.65859043	16.99410302	13.29070617	14.40338887	14.041919	14.01096699	22.94483861	16.69074039	19.2767542	13.35631602	17.60434799
4.989517848	11.77655524	8.59160642	13.7323301	11.80054209	17.58015629	16.4892927	12.73369345	9.243187899	6.351422832	33.65651065	9.618198038	16.15571153	15.89194181	17.06869984
88.63956225	122.6340358	86.62577835	125.2051838	112.368336	98.13335425	110.3845086	97.13412769	109.5908787	66.74098406	189.4260281	112.5768099	141.9089164	107.1612431	115.312194
14.67582623	25.68417202	21.93099139	29.74548664	28.8507372	35.93856002	31.9129944	27.71875777	24.99674552	16.45459948	57.41423584	21.59790656	36.73641776	30.35584062	33.09610214

TARGET-40-PAUVUL-01A	TARGET-40-PANGRW-01A	TARGET-40-PAVCLP-01A	TARGET-40-PATAWV-01A	TARGET-40-PARBGW-01A	TARGET-40-PANXSC-01A	TARGET-40-0A4HMC-01A	TARGET-40-PAUXPZ-01A	TARGET-40-PATJVI-01A	TARGET-40-PAMRHD-01A	TARGET-40-PALZGU-01A	TARGET-40-PANZZJ-01A	TARGET-40-PATKSS-01A	TARGET-40-PATMIF-01A	TARGET-40-PANPUM-01A
10.4942637	8.469137578	12.22417334	10.31750629	15.94256133	7.028556918	7.992822177	9.2627638	10.63665364	5.938127148	11.24128885	16.99418759	10.51790481	12.69969655	7.537388271
2.064053502	1.2070205	1.880367206	2.790353861	1.520251372	1.106333451	1.273233213	2.266077605	2.734458954	1.578537681	2.618026562	1.946242959	1.525020725	1.277853369	1.770075303
55.94748126	30.54274357	23.46992076	38.46385887	29.47228361	29.46369907	19.90460427	70.5556716	31.61356025	23.16734395	28.8934975	41.60136643	42.59623158	31.18949384	22.30044533
23.44927109	18.45086512	27.4938717	22.24884297	22.43471839	20.85423385	22.76675915	23.90884172	15.91072359	22.20231987	28.28305744	25.21338434	39.35557299	19.40483058	28.23521385
29.36416302	76.22588088	61.85994941	78.05586258	48.61887674	49.39427769	38.641125	87.78666457	66.5589614	47.90054785	47.30682544	80.18151438	34.27597881	32.60113093	35.46800883
87.88616942	89.01910628	101.4988936	86.89179195	90.98726081	98.739321	91.72903231	104.7003624	58.33084896	72.60634852	71.19148185	87.56433441	101.0671269	72.45460867	59.22601236
14.37772236	8.483655596	8.468239293	20.83466267	6.008171844	10.69892423	7.697750178	11.46594051	30.94014686	10.87711029	13.49270468	12.72999909	11.38041666	10.45137745	8.130735625
128.2890175	131.9051586	113.1634952	222.7474082	88.87030611	201.9274341	127.1600746	172.7385582	213.0399081	129.7410906	170.2314658	173.0900006	128.8433175	121.8137834	111.646486
16.16357728	20.62898601	6.930535818	43.51730203	9.017385819	15.92412707	10.76451105	11.23715259	79.13585149	12.58289926	20.86313709	20.37480878	9.860590378	21.27572951	4.360704073
17.71643638	12.64647012	11.58751063	50.64498716	4.946951008	21.35924568	7.051785686	16.02123661	12.63350066	12.96575266	16.51904395	16.99767956	21.0158925	6.773654226	13.29100868
0.064032632	0.050398908	0.02455153	0.060660837	0.020019265	0.040563134	0.026016725	0.07051303	0.029946994	0.033902957	0.060390495	0.049265136	0.033731244	0.023878068	0.014583584
13.87235733	10.38443051	8.548915083	9.806670082	6.579256705	7.745137719	5.482712152	12.54880739	3.998530585	8.254754588	9.455249058	11.56163845	6.740809933	5.048632456	5.065034418
13.36500507	28.85956199	24.70561786	23.43081831	22.14628684	17.13613386	10.98674794	47.79552796	32.82900024	31.22624079	27.39332655	34.95564932	21.91545039	8.598431593	12.79111959
146.4836987	143.9163153	92.51886987	127.4259027	143.4781493	106.6393058	119.8751137	172.7994493	126.1609252	121.1007964	119.3234149	139.3839568	115.8659292	94.1851371	89.02820411
37.66130973	37.38575811	23.27639905	32.78715357	27.98730459	26.33396562	33.60789137	34.02205686	18.15319981	29.0660174	23.37760291	37.30451769	13.23396947	23.73138228	13.64028536
116.3023672	142.6926848	165.6460917	94.81627534	129.2362421	139.1191811	129.6687403	166.8965741	139.1005101	134.2592736	115.2424445	154.1967781	127.7900289	110.9392516	84.29084583
6.619157048	2.283903012	1.974184977	4.419104332	1.698736802	2.505138183	2.596297252	3.486057811	3.251417336	3.265740678	2.499867409	2.088997418	2.455323735	1.91664027	1.733334726
108.2414514	104.4617083	73.207973	72.39550339	103.1875894	82.72079471	55.26064912	121.5616389	69.76273457	91.23610753	79.63580385	97.93357308	87.21047611	60.39824789	62.36361257
17.66882128	25.74395678	31.91034288	24.8947835	18.57085006	32.9202865	29.36185721	37.15033028	19.65165229	48.33782845	14.86350451	67.84840952	21.89479116	31.06797642	31.9619084
35.19581895	50.74037785	50.12115696	49.00083475	43.19841709	40.74269215	39.80893824	49.02348367	49.84327876	37.22979864	35.18926271	66.24805596	40.94698565	37.51592919	38.66563865
0.013942121	0.017680078	0.015752859	0.009759788	0.016137656	0.013109356	0.007000259	0.02771452	0.006478633	0.007818548	0.009888948	0.021588009	0.013438543	0.009692874	0.016648146
33.50401559	48.19806178	33.51646291	47.40736187	34.1797442	36.52594142	32.41850956	34.43061784	71.87289451	46.85668696	38.10523926	44.48152936	45.81881209	40.29712365	39.22298555
9.781898439	19.0880416	15.60173788	19.38740786	17.38152064	18.06138851	14.49407665	16.63332022	9.819115628	13.38571717	9.890556154	23.41961275	7.213158564	16.24626508	13.07562915
13.86123206	12.08056674	9.506504341	14.01096535	11.02804339	21.13847473	16.52418442	12.69169293	17.41705416	13.17984053	14.11987481	16.77989113	14.01210685	10.4698483	15.95049629
64.24678302	99.76143632	65.16700216	60.28271151	79.25217851	83.88192603	76.16448639	117.9563356	80.14618565	75.9962797	44.10491401	145.086232	54.41853279	57.26620226	65.82939557
14.06303996	21.18762332	15.09757308	24.14381012	9.93094016	12.73250164	14.07545853	10.00733599	27.15374275	17.34075048	14.83390942	27.55411281	9.820794929	14.44523856	8.109933102
205.2575232	246.6247443	189.2095957	173.6005305	221.1843792	228.4876189	185.1693789	262.0954443	162.6829227	274.8663718	241.6090322	276.7112273	185.4229742	190.0341014	118.9132417
1.569342952	1.265857751	1.208318409	2.43082721	1.232027997	1.780732371	1.258796082	2.466891973	1.25083563	1.232871937	1.760893791	2.032812782	0.955729297	1.346353527	0.944459775
70.65144163	107.2873681	66.72833489	80.71713007	75.23650609	80.00169353	61.09283267	92.89333414	63.59687171	76.73379904	57.11989559	88.13581892	56.93250527	79.56959754	43.76043844
107.3091507	134.2215679	140.5352825	126.8372807	132.2898134	120.8081542	121.6766395	174.2173763	127.2068699	146.206133	154.7966356	113.0815384	107.9215946	100.4249571	106.9048398
1.350990088	1.781392379	1.041724051	3.711263267	0.901517765	1.597092986	1.212288462	2.06263414	2.394637524	1.884637663	1.30075275	2.10229091	1.239205206	1.730696093	1.065237376
127.7853717	119.2203777	147.3438066	125.1385838	135.4933056	121.420267	107.2426057	173.8442799	101.5204461	141.2041614	108.6241465	129.0409351	120.2024356	125.5371236	98.41235665
31.83038174	58.71745643	23.16986964	42.94631917	23.79655266	50.99646793	25.82184352	22.54814461	61.47599241	36.13465086	24.12380506	47.95175126	23.2712588	45.61375527	18.5061352
13.71944169	7.096807658	8.813576854	14.37215407	8.080950384	8.008617293	7.744759036	8.006338343	8.474749872	10.41282886	9.702961685	7.619577557	7.393935256	7.333687093	5.841234295
5.58121343	9.030125907	7.161856162	6.204042027	5.494543087	4.830578942	3.013325714	3.850616618	7.281720938	4.929350298	4.205572961	7.154756416	5.146790327	5.90471911	4.622969735
31.21975235	30.17601315	16.28681934	41.78907855	19.37947458	32.84796735	21.07386098	15.29280343	46.60490378	23.91523502	22.86065597	35.93718067	35.64746466	29.92163609	16.39453609
2.366298992	11.11151475	7.549855338	9.52339038	4.304242669	5.305578828	3.66603203	11.28593206	7.018057358	6.92730911	4.06695085	13.07294313	4.951001869	4.009920741	4.645100196
18.61362551	19.7021334	11.18839309	22.77364393	15.56599061	22.33777302	10.73446801	18.11894144	37.59952521	16.85383212	19.50336902	21.36191114	16.91405328	25.04003552	10.35556172
13.32675788	13.77675223	13.62740651	15.07095906	10.80761765	16.64895317	11.04221416	11.28354839	14.57871439	11.04714465	13.70643632	13.14982888	14.45862271	13.04893014	12.94365934
101.9712117	94.28087145	93.82244651	86.07480354	88.70102749	120.7254576	108.4537779	111.202201	120.5027593	106.4824253	104.5926857	94.92655116	104.9014049	88.48289061	104.6327882
11.86081356	15.334184	9.402467353	16.00383004	8.072430732	16.44565439	8.883557468	8.584822668	28.23354178	11.79446872	13.35600045	14.03290818	10.80347272	21.01955666	6.104280059
0.00756852	0.006502025	0.004145112	0.010869135	0.003877754	0.007144323	0.004376067	0.009350141	0.008498836	0.006416451	0.004888925	0.010486488	0.007121949	0.004693169	0.003621529
3.354192724	7.647620948	4.242568829	6.254439092	3.480705096	9.755415563	10.53070383	9.143369058	16.29309632	8.672737557	4.638535995	17.33588858	4.693230893	8.577273301	19.21070105
6.596784731	6.654066469	7.653043103	6.717867748	8.556604174	10.17797275	9.700125707	7.670443513	9.395567996	6.450173258	7.216397649	7.728841819	11.36860288	8.947335294	9.027945153
17.9141514	13.3893789	13.60714262	22.00147087	6.133346684	14.93418138	6.668434572	15.7714734	7.69662441	13.24609274	14.30194505	22.86630391	19.90123016	9.169696199	16.59684704
2.80480613	3.092098804	1.952539007	3.918353986	1.861073111	2.528089651	2.199522345	3.213416319	3.228439376	2.826766375	2.33414766	2.945436459	2.3407423	2.50405927	1.664666588
8.545330902	10.8227178	9.003335351	12.63501235	7.839666066	9.55921854	6.291991516	10.83343558	8.197915585	8.412834486	8.916894174	13.54570367	8.707926149	7.673682648	6.01675905
0.234511175	0.232014772	0.144735057	0.234974766	0.185737052	0.272286365	0.171964687	0.235964771	0.134546935	0.215769372	0.183911322	0.204788129	0.125507078	0.190448574	0.184485962
40.48296246	67.07215091	90.91938326	48.12072416	69.41580605	80.30404362	56.9335885	107.4117328	87.67797871	44.42965061	72.73942038	128.0935793	79.78353296	69.53243865	40.44227383
104.0969897	115.6513559	99.01211445	105.0803191	129.2386447	105.6464467	102.0941314	123.6743812	238.3403923	140.835875	104.3138138	132.2454675	105.3230055	94.81752579	81.17976109
35.79300868	54.92076198	74.72479032	39.39336867	45.84006008	61.71198277	68.09123275	85.17776934	74.40438301	60.89463064	56.51051458	60.97896397	81.00977495	48.7888495	44.68830523
150.7961379	166.3571964	203.1510905	149.746179	215.8151912	167.2472336	147.1755533	138.0228948	417.8793012	163.9534884	171.5693318	189.145472	189.1979269	162.8115745	95.56134264
0.586252861	0.637591866	0.639971328	0.826106918	0.53792679	0.716429214	0.720746392	0.912024865	1.828094159	0.390898641	0.85015074	0.731799206	0.508203227	0.59165865	0.428233872
38.62240231	38.66457824	32.37369272	26.47733779	40.18444847	34.71905575	26.27544766	47.23400181	29.89795381	32.18453583	26.87091042	46.96023304	25.13298325	35.06844904	22.42551916
31.38091427	25.90345332	19.66436921	27.28180695	39.16860568	31.48575755	16.72218564	42.31663057	20.37223126	31.03052637	28.21984655	41.36166944	18.46752923	20.15528924	18.25174925
12.28175801	12.95465478	11.09130117	13.34068918	15.238209	14.1940673	13.15181917	16.44634327	14.74922251	12.26238756	11.89171742	11.9316405	10.00356238	12.18769212	7.600752502
1.710039809	3.349978242	2.476461359	2.56068475	1.783296287	2.906725958	2.277278242	3.588040822	1.883657991	2.599479045	1.81651668	2.314844602	1.820459585	2.461426418	1.444758357
0.540437536	0.377044593	0.366513863	0.485279067	0.498568056	0.666633427	0.313059001	1.166399579	0.448223996	0.462567076	0.934185946	0.407635279	0.634308645	0.383245877	0.29718224
0.080559021	0.098408229	0.047962417	0.204982571	0.086645245	0.133410034	0.047412691	0.162008656	0.046745901	0.099489852	0.167073675	0.082027046	0.067193916	0.077971855	0.033630457
0.120292046	0.15921372	0.086414264	0.090213146	0.146629305	0.220438451	0.064016137	0.466636615	0.13520777	0.122689397	0.357378463	0.16776228	0.212079986	0.101924994	0.058497056
74.01568012	109.6166372	100.7188007	126.3469199	115.381974	81.31319038	89.92262341	104.8483649	133.1338114	84.40015026	98.78772176	105.5140394	75.94516971	87.2922793	68.28311026
366.2082624	480.9552614	327.5021383	426.3085437	442.4496908	452.4674122	369.8066647	395.3691265	417.9837177	381.3836234	396.8399145	497.1283816	390.723747	353.164571	234.9373506
183.490112	159.8425372	109.9437364	84.36295365	117.3640178	174.5921167	127.6927328	134.1784857	129.0245793	98.45986109	129.3967117	130.2941443	217.4372078	102.4661493	130.4990518
0.088134987	0.084098906	0.056706474	0.055319142	0.079155075	0.095674455	0.04998017	0.133857427	0.061614889	0.065617207	0.088218552	0.091500373	0.120087689	0.063289915	0.048387521
2.028633796	1.596902478	1.176659431	1.340458996	1.887593138	1.114408433	2.047522399	2.663275618	4.424676611	0.830187568	1.594816516	1.396936572	4.997777487	1.287236061	1.1409397
1.014571621	1.580740936	0.764115109	2.076997735	1.676232399	1.247745525	1.461947492	2.028168644	1.532998165	0.715743351	1.591585964	1.400890391	2.626585066	1.1670274	0.633005746
0.658654433	0.688246655	0.558011026	0.994184316	1.104227971	0.581147776	1.059341783	1.501361689	1.521683177	0.404776434	0.728468635	1.048529894	1.786539591	0.624812291	0.593520236
426.8006782	459.3403162	427.689581	332.5097697	465.6056859	531.5044707	383.8983498	582.9052889	433.7728952	414.6952701	396.7286018	593.9297633	407.652307	345.6371144	291.187273
166.43058	103.3727065	95.7859514	241.6856684	88.06230672	206.3206062	67.93036636	189.0027404	194.0577677	84.4988796	191.5736348	144.0831788	253.2867255	108.4179017	74.27824197
145.7583221	266.8430252	53.66851969	119.4190814	143.73126	272.6879749	109.3301259	226.0543654	209.2153097	278.9605567	88.44510232	107.6547546	35.80447604	94.29832862	42.67344577
34.19654041	44.14171179	36.49312971	39.01776879	41.58181963	46.82455287	38.5145298	51.03693883	64.33805853	38.18450277	41.39331975	52.22452599	40.38909751	38.03342994	29.0404157
78.03131165	93.79669011	130.6736406	100.0919184	113.2022846	71.30873572	39.57588916	106.5126124	181.1352891	73.73492608	98.59587689	102.2908745	110.9236964	60.27932967	44.32185587
19.71299038	14.09111402	18.42087421	13.09892304	18.5107613	15.41725268	20.74232238	25.44768966	30.92530699	18.74500203	18.92191086	11.90791469	25.3395909	18.68820553	12.02678723
32.33718814	17.34781281	16.00411327	21.32687076	27.13457287	20.3270579	16.92453304	27.28344429	25.92197532	24.92536175	31.12100257	17.96201393	17.27818506	16.79548858	11.5526187
13.50290872	4.753199949	4.324432499	6.868596023	6.084748419	4.501572092	5.14202906	5.053026359	9.216272007	6.16926914	5.726311102	4.432828372	3.506370965	8.993807342	1.65041375
74.11684479	43.51794382	28.19145401	162.7452419	13.74409534	83.44521416	13.90220834	29.60068431	53.29028999	39.93060874	104.3780742	108.1501396	159.3830304	30.14645769	79.22023259
4.193494416	10.97928772	8.151924254	12.3058151	9.149686387	11.44542393	4.258252708	9.817733396	18.78522061	5.601899161	11.64402768	15.03341039	12.17006515	7.984439322	7.784808332
4.592583947	13.10269988	6.996100204	6.362849949	17.61551469	10.75682434	4.48017252	6.004830902	11.2590326	5.394580059	9.063630405	12.28848096	11.428871	6.997012871	5.295593998
23.09962776	48.34534534	55.85758946	31.74067525	31.84892743	66.12684585	46.16359083	75.60300077	49.53914924	45.9613989	52.47169014	50.1497645	55.62187363	40.95269579	42.78598753
9.049900545	12.3419816	10.29717831	11.59337627	11.33785337	12.08472067	6.330194049	11.07740547	15.06777293	7.184318207	9.634898984	11.3501313	11.39575808	8.715396777	6.687264938
32.62842071	150.1612926	98.12163284	62.80204404	111.87245	123.348774	113.2642881	124.5964055	162.3557544	45.23118024	98.10645773	92.08212877	51.88392807	44.58228796	38.30615583
47.91043791	54.02058319	49.73714285	50.98966263	57.1827993	65.36680366	44.03292538	60.55083166	117.6858652	39.79831748	57.32550512	65.35440191	83.98334772	68.8797823	59.06926465
105.3222569	108.6746497	118.100513	102.4218169	159.2903223	160.831359	117.720261	175.3267032	178.8780566	120.1300894	137.8227926	121.9736161	124.8833135	106.2696369	103.188623
44.04492335	19.63872978	16.90244998	63.4934332	16.59762801	33.17478624	9.55435864	27.38962251	16.87414595	17.21071681	50.04072911	29.11072947	54.76820476	19.88419286	30.67617324
21.63988658	9.827832078	7.820585914	19.82943122	9.258545525	20.91142641	5.325288682	9.57496385	8.80550739	8.63235817	21.6284941	16.72421159	23.80053091	9.825721206	13.06772356
0.056086081	0.049721502	0.033537162	0.081727398	0.03987666	0.046719507	0.036210945	0.045773973	0.045457474	0.029783203	0.063715232	0.044924902	0.044962695	0.058676112	0.028593159
0.047838631	0.084565609	0.048570875	0.107125782	0.110758149	0.137237148	0.049530469	0.075583827	0.186445749	0.052407429	0.122881579	0.127249205	0.047211651	0.090838299	0.024512894
0.36417598	0.613214309	0.456313495	0.654704038	0.586748311	0.946455718	0.386322668	0.750958479	0.907522981	0.350611593	0.724295396	0.834801856	0.562044806	0.568808072	0.326094044
4.924699813	6.967162594	3.331551323	6.653404864	4.423625064	5.704603664	2.957715547	7.051522894	6.191437371	3.047010655	6.486374891	6.808022691	6.801378481	6.700489264	2.282611529
163.6851613	155.960472	161.819482	159.5317124	155.4981035	185.7595855	124.3717274	168.3634857	169.2652867	147.9742492	172.4478317	181.6216567	235.3970614	118.5194092	135.4947578
37.25043683	47.36260098	68.3483211	58.60487202	69.28056131	72.72654129	65.27245024	81.10775011	55.12024437	50.2933292	72.07356712	60.37989274	55.66784644	48.42617385	51.36866469
45.17052955	43.73693952	46.18983386	40.49104709	48.13144762	35.85256301	33.66322839	51.75129287	34.49912829	40.86878244	39.27104542	46.16541235	54.73865826	30.63559021	51.2662609
19.35698667	23.14251126	22.27324963	37.04012042	28.50761955	18.62356422	10.57282064	23.14436357	52.31062089	22.16902311	29.92038692	37.71805879	30.18229959	20.02433194	18.95799402
48.27669276	35.39389733	37.17122673	33.65131906	48.84913198	33.88261877	21.86716949	49.63769901	41.89352485	33.42159735	38.49993307	40.51953548	67.09253391	22.18171845	35.40276612
57.53264806	93.75923843	108.5266297	62.53217222	96.51293149	84.48440809	72.44959469	140.270947	66.71403584	70.52320908	68.49236052	112.0561973	80.80024953	57.81362042	71.11248477
15.50887415	15.90121179	15.51645616	15.07997512	17.48655882	17.78116047	12.36189314	18.4809279	16.03938027	13.13228608	16.20182837	16.04185161	20.76085954	12.80525543	15.32149053
10.49307148	13.83747086	8.707393859	12.64421628	13.88321358	14.22160958	5.413436382	11.5502516	11.50839636	6.322588346	12.08256514	14.55849787	18.51082042	12.08070649	7.339122381
103.0011676	125.429043	101.8685502	83.30978062	130.5202469	118.566369	81.33807702	149.3583872	103.9570897	85.0647028	106.067602	116.553244	167.6148408	86.01230297	102.7429759
28.50133246	33.74539239	24.5592875	20.47015982	31.20387981	30.14211836	16.55481436	30.10359654	21.87449568	14.90740858	20.7868328	32.10974922	34.32427069	23.47122033	21.24962149

TARGET-40-PAKXLD-01A	TARGET-40-PASSLM-01A	TARGET-40-PAMTCM-01A	TARGET-40-0A4HY5-01A	TARGET-40-PAVALD-01A	TARGET-40-0A4I8U-01A	TARGET-40-0A4I9K-01A	TARGET-40-0A4I3S-01A	TARGET-40-PANVJJ-01A	TARGET-40-0A4I5B-01A
21.43312256	9.108258345	13.88739401	20.28851233	16.12654418	6.573465605	13.15736008	5.440863476	11.31005241	5.27998469
1.073909718	2.295251434	3.094196463	2.024653482	2.498627686	0.722551886	2.179298556	1.296390096	1.420382782	1.00143488
47.6119064	50.95283768	31.87847517	37.27017768	88.57868914	31.02582297	46.49042392	73.73005972	45.35328942	27.72631043
38.45333406	32.17808121	27.30671021	33.32203572	34.79732006	22.56946974	16.77368199	26.50903233	20.62439942	18.65488399
107.2603818	72.75196077	125.1207016	49.21077402	69.64733487	33.11501656	75.9808952	109.2340339	131.8069663	25.44479912
67.46603626	100.7513552	95.25009102	83.26840811	109.5898327	87.80887075	129.9639469	205.6338099	107.9345285	75.25613085
9.679659536	10.64048905	10.14633942	8.955999895	20.91392266	10.34992931	11.52590015	11.8072783	16.84147988	8.628543596
190.1045875	171.9987588	159.0024474	116.0417714	218.2722791	125.3804614	201.8412715	221.3663912	189.2628619	108.9388205
8.579035674	26.52703616	14.54242165	17.01578603	18.22580602	13.73440359	6.50362008	18.31638818	25.68547099	14.8948109
18.6510473	25.38791826	28.69168397	11.04610218	17.84306949	19.13921734	12.4753606	27.17008551	63.24129768	14.60714027
0.038824345	0.025833171	0.056775448	0.01495787	0.052029768	0.030799745	0.119203184	0.060763212	0.050901419	0.017032606
9.19645716	12.18225488	19.15771022	5.152960323	8.771392897	5.764157215	8.664477212	7.40274127	14.95592552	6.893868367
50.20828724	29.9653451	57.02894063	20.70366053	35.12555656	12.9647125	28.71648763	14.44030282	43.71371571	10.16995126
143.332855	110.1644621	197.9017894	85.52796184	182.9717766	93.26186237	97.18277109	104.182509	99.29351224	85.63194379
24.62081937	22.10207083	35.06335017	20.50511179	19.63965214	18.40454254	36.41981342	34.87803352	25.93660177	22.51325781
135.6344828	101.5719531	127.1090582	124.9353584	127.3891843	102.1313848	182.2020602	142.7201534	165.8673576	101.661315
1.408389546	2.438440501	1.453689478	2.130228296	2.736980201	2.497757829	3.119443475	2.808032657	4.170594203	2.42428803
80.40085053	61.90583982	97.9490565	49.27333397	98.8372091	68.3629	93.74335451	120.0133293	89.39950525	65.80982229
41.13422565	9.836704411	35.58987921	16.96912058	39.74964733	31.32677949	37.59097869	41.7066037	42.80388309	15.26722689
83.71350582	35.22287936	52.09884065	32.07011399	42.3007466	37.44654431	59.74020056	41.07680905	51.44811403	31.53922222
0.010819772	0.006114466	0.014641111	0.008946791	0.015278804	0.013782387	0.025071435	0.005628414	0.013494822	0.012987079
50.17135816	40.10850261	43.47804043	27.59006271	35.01652858	34.61808886	48.74833639	34.36936909	37.98204675	29.18489681
15.78393543	11.74831823	17.39501725	13.35572783	13.03044198	12.73347069	16.35252121	12.38179483	14.8718744	10.10799624
15.3748179	7.530183018	5.865575685	7.257520097	12.62238479	14.62981102	20.57626003	13.6415466	19.89987276	10.39610891
99.93794869	61.06318316	49.95305602	41.4619583	83.91760997	93.40443904	217.4327524	79.92002253	182.5987617	54.05128302
9.341368342	13.8186275	12.36934199	13.2055899	17.09118808	16.01758158	25.24356913	21.55112802	25.54256278	9.565500263
264.3608277	154.8553375	294.053903	163.7417192	314.2086827	174.2142236	313.5925056	238.3113603	215.8500469	137.392213
1.455801023	1.385486817	1.772673051	0.928193817	1.859464313	1.392461217	1.640145887	1.496257905	2.214601566	1.338924804
73.46359612	49.31125286	90.10244616	38.13125914	94.34396762	57.66901273	107.435304	84.44285864	86.10858056	36.87961064
138.9012305	161.0093819	175.3480166	108.6977743	114.5554903	98.90981143	167.0986851	145.2488927	134.9572183	90.17761125
1.664491307	2.447052424	2.577220877	1.295133562	2.819541867	1.400561136	1.15055141	1.923382999	2.696497708	1.357310846
155.0493712	117.9261138	162.8384359	114.5670167	109.059157	117.9654699	212.2115364	178.8183178	151.712793	100.3076668
36.07427086	33.88615727	34.40173948	28.6217844	36.15462311	33.58112076	19.9694606	61.19877219	27.37410883	20.20177394
5.85169485	8.331467945	7.788841896	6.885453115	8.446320303	6.924628346	6.552261864	7.132666457	6.015559522	6.963560105
3.448611365	6.878860523	5.670001954	5.510817071	6.596710286	3.254470162	6.678853498	6.468713477	4.248446387	2.936433766
28.88051358	46.46399623	38.95229908	22.28188097	47.07008163	27.50571836	11.36324148	28.02393098	26.88760971	17.43936494
14.24662204	8.192744513	10.77417874	4.462262264	12.22431378	5.027180421	12.66309022	9.37406902	28.44113913	3.382035739
15.5041765	19.29634829	20.13476142	13.97613239	18.68035266	13.76928301	9.382772334	21.02047146	19.72345912	12.82590965
9.411288106	18.27200949	12.35078793	13.57797978	20.89204035	11.78685598	12.28405051	27.77560511	11.59704704	11.13571256
138.9773326	98.73690224	87.80189966	89.11150893	95.12754906	92.79887899	103.6341115	95.59622324	126.4926912	83.12669468
9.945495747	13.46341746	10.5244634	7.772707698	12.48496342	12.61866582	7.710531783	12.83394032	11.11772268	9.658829037
0.00609135	0.005547044	0.005382447	0.003232639	0.009654238	0.004147216	0.014439733	0.008849079	0.012480274	0.006715478
11.0398632	4.336669501	7.43897392	7.758161055	19.26310648	6.931399311	6.487503473	10.3435866	12.35882791	3.259612031
13.88871139	5.214902865	7.627273079	9.105689863	11.9106694	8.432959813	10.88880744	10.32095862	11.3571371	5.227973323
17.63114418	19.63127398	21.81433773	8.675862786	18.4599454	12.56667212	16.14358916	12.699955	41.2675255	12.15611563
2.707899555	2.679241376	2.349434307	1.603454881	3.164654735	2.463663978	3.168498757	2.623477705	3.435634324	1.780076846
12.57696824	9.839184214	13.25453741	6.233844957	15.23045023	8.065311075	14.25665729	14.06085855	15.4263953	8.01837814
0.17119169	0.187478928	0.233574019	0.112301404	0.198241112	0.245039476	0.129134167	0.253810742	0.233902562	0.192500382
82.08391962	61.81882356	106.3538897	37.33233714	78.61889109	48.36007103	174.0753091	65.05622639	167.1214167	46.67314218
117.1624704	102.3242181	63.41387105	66.65490522	98.98278117	65.05216082	158.1375193	116.7937728	117.4754115	64.80338639
83.97397847	67.83018059	34.95446349	53.69187748	45.34488729	36.77079511	97.55245426	73.92175693	83.73158206	38.67003824
200.4007193	110.3212882	137.4232446	153.7040684	176.1405982	106.8804669	322.0810105	152.3488423	258.2836223	95.05695244
0.551722505	0.474320067	0.505864488	0.482088913	0.619954257	0.496283044	0.937470672	0.61854536	0.78791836	0.630968567
27.79837014	22.19513047	33.6367237	24.03112919	42.05381385	31.37665331	46.29852315	40.51977679	52.65110067	26.66269351
25.10830377	22.41531659	54.43228136	14.7564079	30.28438174	28.40937321	29.55723356	36.95495346	40.66128552	28.13953283
11.58944833	9.988070276	9.960261818	9.937916266	12.63686535	11.35169219	18.60154689	17.2613626	16.0022454	10.91371667
1.957996691	2.408052345	2.332397854	1.652070532	1.810480223	2.056574064	4.060381645	2.989263027	3.608431057	1.66842257
0.5840176	0.488512578	0.633964286	0.282049872	0.692817097	0.475942622	0.676163104	0.528670921	0.505908575	0.324560907
0.079478831	0.091009352	0.055687919	0.020476232	0.04353447	0.044011992	0.241452551	0.136271728	0.103284432	0.033606948
0.257022714	0.101723352	0.251513329	0.049982165	0.250844713	0.120636243	0.402614551	0.106082632	0.163276227	0.047352889
94.33050193	75.38338034	95.69039686	62.94373436	70.93204757	68.43643245	168.9422717	86.10489092	121.2426135	64.21366266
413.2832671	286.5516591	354.1462018	345.0061004	471.3785744	336.8907205	534.9733125	482.0128762	439.2294939	256.8641553
224.7400823	111.8334145	132.2484901	134.2162073	206.4724646	147.1769702	202.5966722	124.2782022	151.6746565	119.6344866
0.091700907	0.061026706	0.055842065	0.036171121	0.094333645	0.084295219	0.131191591	0.100556226	0.131844552	0.066616119
2.030079307	1.095391575	2.195525802	1.197045937	3.595874842	1.27968005	2.958089729	1.98203443	2.379214865	1.565095255
1.522232933	1.061402492	1.611687066	0.698160382	2.796561855	0.864696476	3.643177774	1.477419567	2.250144551	0.874691392
2.265079681	0.516251591	1.48066112	0.764334812	2.869475663	0.592125452	2.608535461	1.577168762	2.443213723	0.708039835
480.902038	310.1197302	392.1658681	319.2001617	495.450547	387.3828455	584.0505639	440.0016919	518.068536	327.7783055
125.5207644	117.3056447	117.0330039	79.12851727	191.9246937	90.37424301	169.7431454	127.0819057	199.735652	132.99676
91.16940056	171.2479139	54.19186224	31.51630809	66.97565521	90.57317452	57.31852875	92.31835632	170.217614	124.3965455
49.49316063	31.49523521	42.73675604	31.56583067	46.46023785	31.91512669	58.32491115	42.50298147	57.71327973	24.61881756
44.10646809	91.55083867	52.2780655	55.59388368	63.64569764	41.55150162	136.8600824	75.05498323	132.6027727	49.65642152
23.59189609	13.01595824	8.229775003	11.8922198	13.84534083	16.17354609	36.12312591	14.30580112	19.00787178	10.86457427
16.40819619	15.40043306	13.25580803	16.09984658	10.17580502	9.401629502	22.06698712	16.9004573	16.76770644	12.91792768
2.537064997	3.085470153	2.624275527	2.984063725	2.961398354	2.451392962	11.3975642	4.715387274	5.439393773	4.365252499
96.48824538	145.9555753	177.2324862	36.71054218	106.8672825	64.56035868	19.36326361	54.65928612	218.6725492	49.61909953
10.65186852	8.92904953	20.10877443	6.164304016	8.64566352	5.960980554	10.36964825	14.87725463	15.06474939	7.117740202
12.0498055	5.70318095	10.06612895	5.666747941	5.963581991	7.634732353	11.78164773	21.0539048	11.52343803	7.28727284
64.39872498	51.90590713	46.3690716	38.52517138	29.48276363	42.47826051	80.8340571	44.4268245	76.25325094	25.72777323
9.807341879	9.491775777	13.93841518	8.800161815	8.948205532	7.861152188	13.53528804	12.52228464	13.90909649	7.993582536
73.07106242	44.1115718	45.98754583	65.48864536	28.86713093	40.5044576	199.0624554	117.2076576	115.7522737	20.99428505
87.52610828	59.12728556	81.85241702	60.81713847	63.46448613	42.08173124	95.99089139	74.65664828	67.84040036	44.96038865
192.9982581	111.5380793	103.3161137	112.5275951	97.19994997	113.7769139	172.3086944	163.1261949	247.6103198	131.7974221
40.76345329	58.48886924	110.4384065	20.79975064	31.43983747	21.43995597	13.22553749	28.4368457	74.17597169	31.7291268
14.33154467	20.02562198	37.75517513	6.339186584	12.11291815	9.523925882	7.044924339	17.47252364	28.57159549	17.39514549
0.06001792	0.028212039	0.065543604	0.044170705	0.04487018	0.028327752	0.054170308	0.037613314	0.046186171	0.022856943
0.122144674	0.142314541	0.210761134	0.076811595	0.10821671	0.036441622	0.065370894	0.08606533	0.17770768	0.071518492
0.958332519	0.84723249	1.455424048	0.553782478	0.806139746	0.367039611	0.601031959	0.875088734	1.312695366	0.475775948
4.288876052	7.214119848	13.44025983	2.158716553	4.882399625	4.421676944	6.810488652	7.140334603	7.615226431	3.736708691
219.144151	163.4626519	161.3279077	159.0841916	165.7497417	152.0991003	181.0073748	195.5588355	182.1926025	119.773426
90.70643139	68.13409399	41.42010747	65.35617145	45.5769425	43.04785994	75.18985816	74.87972573	78.3460382	49.26275629
53.83285638	55.66040463	69.29821619	46.41064505	54.22669727	38.098093	40.20122206	45.84995375	47.4827611	31.05833119
29.73000713	21.47810971	46.31725266	23.79259178	46.94056913	18.3638576	23.0255056	34.43872132	36.77903377	16.485429
60.2864912	47.11916671	36.59725186	41.05880052	54.35103906	35.03954179	44.1736575	64.66398988	40.88793669	31.65987771
110.0342275	85.02743935	75.11850175	72.10226447	68.61683539	67.83105367	147.0688807	96.90888787	116.5371684	54.47677758
18.60763658	15.57224309	16.14366064	14.17298125	15.56265117	15.67774741	20.12418369	19.3529728	17.90600322	11.99340489
9.189111438	11.41920336	14.70480245	4.285749288	9.195775081	10.92948946	15.61924147	22.69318069	13.50822033	8.13328122
131.6582104	107.6655948	135.5248418	66.1674164	139.4862108	122.8344266	152.6951016	127.2725707	104.5923655	65.17161916
21.02541481	26.24957323	34.92022932	13.67451761	22.8530438	27.73084568	36.10932324	41.05737495	26.14649408	20.26746138

Drug	TARGET-40-PASUUH-01A	TARGET-40-PAUTWB-01A	TARGET-40-PARJXU-01A	TARGET-40-PAPWWC-01A	TARGET-40-PAUUML-01A	TARGET-40-PAMHLF-01A	TARGET-40-PAUBIT-01A	TARGET-40-PASFCV-01A	TARGET-40-PARDAX-01A	TARGET-40-0A4I4O-01A	TARGET-40-0A4I48-01A	TARGET-40-PASEFS-01A	TARGET-40-PARKAF-01A	TARGET-40-PATUXZ-01A
Wnt-C59	51.58574913	61.80171983	132.5806269	60.72165817	73.85032913	76.53379839	70.43825157	68.63688842	68.34087588	76.3558822	53.1801114	31.57835955	74.73703711	67.11106146
PCI-34051	100.4454342	100.219406	138.1052659	85.58930944	96.16473276	89.63467166	88.31278525	69.48651571	88.09252534	86.85048521	94.98258216	47.89723815	56.79071356	74.87149323
PFI3	186.6091892	175.3302123	253.5110166	198.6772185	168.1738942	175.364182	166.1096135	154.8632522	183.2022886	178.683813	181.9753176	152.2191784	174.854627	202.4415999
GSK2606414	50.19883641	34.59830389	74.49212043	43.29778636	42.10914648	31.90675639	36.1255488	24.03152687	47.7596412	43.12406571	28.66190498	49.38009266	35.77838931	57.60103241
AZD5582	6.637356225	8.998378343	7.944577824	77.00331863	3.253537708	3.352591863	6.766860951	4.023808777	19.39608686	14.37729889	8.166895557	10.62949593	19.83292528	11.22590638
CZC24832	149.3863301	118.8771779	223.6091968	181.6989296	173.0371322	166.9286198	156.1248178	111.1822922	165.213853	164.6785077	125.1148517	165.2437691	139.1087983	144.6799953
WZ4003	36.53065384	30.55156028	46.92252741	59.12520347	55.78104858	46.45218415	39.02242566	30.98003625	63.94520428	45.83983057	27.05816629	31.25077424	36.12932323	45.75890879
VE-822	26.24526764	21.77053318	31.04148204	31.41652264	24.65727676	35.01673519	26.07956859	40.64188079	24.79421951	24.81502531	25.17626259	13.69843319	17.30758812	22.60105162
LGK974	47.3650788	45.72867253	97.37419168	45.84893052	52.79085094	69.91001661	44.78685079	57.37307665	41.41305002	51.07829486	45.74815974	36.81039539	57.23968304	85.22700199
OSI-027	105.0242672	117.8447717	197.8269682	118.5962803	61.81240061	171.7589579	69.89009542	109.0853975	198.7669114	139.6564565	68.84713865	162.7445312	124.0346267	174.3309625
Entinostat	14.68316729	8.895823184	8.163471634	14.79635955	6.654317629	13.84685866	14.39402106	10.18711091	5.874418078	12.70887691	8.355270541	5.720959491	6.727315977	5.521657257
Leflunomide	82.0205505	143.6145558	240.1687333	108.1939097	201.2561235	197.4324884	152.15866	186.6353147	124.4910985	118.6227455	174.5066256	78.92749169	145.0314411	173.6204275
IWP-2	12.29753237	13.47817929	19.19710016	18.19634017	13.60851785	17.0432532	15.85170883	16.61305808	16.10469958	16.43144607	13.06577797	8.991752743	18.34457067	17.28620808
SCH772984	20.09771014	6.365401103	12.63837376	12.07695994	47.15558234	23.54492581	9.142080408	6.503777694	14.72218378	16.62176593	6.464001276	18.82210891	6.65006554	15.58997386
EPZ5676	299.2150604	223.0569275	405.7911371	262.6477034	236.2174286	242.9852016	193.1752515	235.6465338	289.173046	254.5628688	280.3814129	213.4933927	190.0819648	356.8841797
Taselisib	25.03893701	7.418055371	11.11114083	10.14743838	2.804177136	6.604154655	5.820924295	10.6310129	13.01512984	9.56167239	4.516247733	6.636735258	6.901390158	10.38565257
Alpelisib	80.41209842	31.41979635	28.91940708	48.81901402	13.97043079	23.53027262	29.94349655	46.6477615	29.87655632	45.04686639	24.81745601	28.30381496	45.72065398	28.57642986
Luminespib	0.126691272	0.089401345	0.063829057	0.088431912	0.189700062	0.116782315	0.101136511	0.084721798	0.115473302	0.070577909	0.113536538	0.218729946	0.063134536	0.079064428
Lapatinib	17.50424049	15.24285763	32.93003305	26.57638229	19.53959006	25.81189	30.90214313	33.65293082	30.80698044	22.38574982	19.19518594	11.07537039	11.65216732	11.7568752
LCL161	145.0799502	131.4723315	198.8042991	159.2585367	111.9332139	97.85441077	159.9557689	121.0882506	137.0303938	134.7643623	145.1644634	157.7808269	128.1574325	236.9058
Uprosertib	49.98684648	15.88264296	23.9000505	39.50661291	9.85744426	23.61958257	16.19014195	23.96641106	24.47858102	13.42934599	11.56781205	11.29165386	27.87703288	30.69807521
Sapitinib	57.72133425	32.26573318	107.1238078	44.38879891	100.9462512	51.21252075	71.66820727	40.78960523	107.9194299	23.7850655	44.92339729	43.84371823	36.79830856	68.14042694
Pevonedistat	0.801053697	1.2129955	4.288725267	1.147331206	5.447394179	3.778299702	1.677463464	1.785654307	0.973328842	0.747458937	1.867163609	1.076192034	1.267958819	2.64675793
Cyclophosphamide	184.658998	143.547821	186.4319991	154.6609196	167.4791197	187.2113718	144.3468236	151.942144	205.9890183	210.7427618	188.1708601	95.22046034	135.4917507	183.0358843
Epirubicin	0.324402632	0.28119531	0.693881373	0.355990225	0.90674705	0.432604382	0.332465133	0.239343345	0.470082433	0.247417332	0.754887238	0.416731055	0.193435102	0.461353931
Linsitinib	35.30597531	35.42051198	58.74824358	67.7595911	19.91434868	52.75777124	39.35677716	70.35031162	33.68157519	38.97470804	41.74194012	17.68949037	42.258769	26.21010226
Ruxolitinib	160.1748624	110.9659107	130.8435402	133.6322867	143.8223346	136.4623274	127.6127458	108.0873384	116.583025	117.4947021	102.9640101	142.131511	133.7272511	142.8782584
AZD1332	54.97800197	45.13677312	48.50855156	56.57682586	46.4063713	36.64178902	35.84551457	42.75868027	86.52939048	38.02437135	28.76492688	43.48234074	38.80719774	67.60954234
AZD1208	250.4278731	158.508932	208.2656005	392.3533197	135.977995	226.8680715	172.329075	197.0735634	171.7399647	233.8559296	153.1948675	144.4168066	188.5713097	223.4353538
AZD2014	9.159581404	5.683562525	12.00941417	6.896320898	10.42569673	10.17581654	7.513145111	6.295081199	10.17363732	9.794333818	5.673537306	7.469034691	6.31103782	9.956236243
IAP_5620	191.976691	171.2673663	201.6251237	404.0571422	123.9748544	95.84470347	180.4241686	136.1380311	210.9962184	206.124522	168.725912	213.603018	179.8953154	314.3328252
AZD5438	6.79131873	7.337905377	10.243116	12.17681897	9.379311841	9.468894999	7.358576562	7.73370874	10.10658522	7.082378163	8.132435214	7.617316918	5.438000508	16.92588446
Temozolomide	274.4468684	280.4756614	505.9279635	405.3901424	503.4033052	445.2453945	374.4300704	337.538438	465.3353595	334.4321082	377.2511043	411.9736073	428.4715245	464.5585613
Dabrafenib	68.21437678	58.3841223	257.5056812	127.3865156	143.1224096	136.6451388	70.89674689	79.0657311	131.7978924	89.31366221	97.64528191	151.0943608	50.18806844	98.10964686
Trametinib	4.264811868	0.767285032	1.046822279	0.894333991	9.367338009	4.511693109	1.250020544	0.612344463	1.742856746	4.16794886	0.64573409	5.100211171	0.714424915	1.460253431
XAV939	71.14347025	66.6954515	69.92696938	68.79875334	102.4672195	93.82219972	77.31266686	74.50684137	86.31341228	69.19208536	68.88265938	91.41016499	94.32193678	86.90781302
Talazoparib	31.01051072	17.35633423	14.14424633	17.09891155	69.59916487	27.96470002	53.64687084	22.03757986	11.46491106	19.56211138	52.61187296	45.00912871	11.02838483	36.16884327
AZ960	6.807871047	5.614645047	7.524731291	10.00590237	9.018529915	4.851224784	5.055753415	3.660782454	10.62969885	5.855628346	7.04064723	7.729957876	7.549219039	13.25352424
BMS-345541	22.82970349	21.97298515	47.89363103	34.61711316	23.62023218	44.84361792	20.12564824	35.11702231	19.56620312	22.7264263	17.99633632	14.83628524	28.95676915	18.65066736
Daporinad	0.011246524	0.01650012	0.009827083	0.005498923	0.011756483	0.013454025	0.009819936	0.021117342	0.00661289	0.010288041	0.020614645	0.002756523	0.026776403	0.004303747
YK-4-279	7.217317585	7.91122419	10.0659394	8.154110242	7.235384329	10.93471324	9.146560188	5.176277819	11.90654641	6.964447436	13.29410827	5.563401926	7.524827617	16.59539707
EPZ004777	178.6279832	143.9631537	241.4878469	188.5940108	145.8763365	163.8041933	145.4146598	170.4008173	206.3308606	148.1004713	162.5485275	126.1888763	142.2947036	219.8880041
Fulvestrant	17.01599115	13.87810905	27.63111121	22.42378215	19.28484183	19.64547026	16.92837575	18.28491862	18.3193511	17.81431065	19.23034473	12.98269419	17.29249543	20.84202721
Tamoxifen	35.13960204	27.3252129	78.78811666	29.40567905	34.74841974	48.79916905	39.4521116	43.67537854	40.4312382	41.10141156	38.48595437	20.40561884	24.05432905	53.66815568
SB505124	10.97161415	10.23509985	9.968770612	9.4212227	10.83434939	9.638350187	9.813828653	9.891533647	11.04206422	11.16417766	11.32126528	9.783421692	9.291371534	10.83060502
GSK269962A	17.7087033	14.54866001	31.42591116	27.62716064	14.38977239	19.03690468	14.04072574	20.01935083	19.21505458	14.66831208	15.71956154	21.67070119	17.46266148	11.89314996
Bortezomib	0.009133575	0.008317996	0.011565327	0.00542602	0.009369776	0.008666178	0.006082596	0.00571778	0.010806455	0.013426	0.010302353	0.006840613	0.005252368	0.009409947
Gemcitabine	0.437876274	0.389038067	0.412331491	0.260301329	3.262303524	0.79097101	2.189840395	0.793904084	0.295925005	0.690952317	1.910457154	3.081184469	0.135030713	0.67964696
Dinaciclib	0.06049	0.052818694	0.061121171	0.073789172	0.040452817	0.044967495	0.033937738	0.037084459	0.049173894	0.059264693	0.050826404	0.048880593	0.065401006	0.083040265
MK-1775	0.948207415	1.850614879	1.689769943	2.030508143	2.36995269	2.081097434	1.402893593	1.557719637	1.643815184	1.104221352	2.37795438	0.53060399	2.272714345	1.857407939
Niraparib	67.12835742	63.20928627	66.34552286	81.60631436	98.51274741	67.95828641	69.40160369	79.40472739	69.62835313	64.47706968	108.2225763	55.7039083	61.31650555	70.00529248
Erlotinib	13.6335166	11.65110086	16.81147317	13.49658385	23.58956699	13.85034852	21.88586286	10.03450407	10.90437763	11.55469553	22.58432123	12.6244402	7.11427294	18.19402002
PRIMA-1MET	84.11560475	73.45974251	170.8246661	123.4248197	100.6732796	126.7108578	113.7212262	79.77223416	194.5681674	150.1032877	142.7485187	36.2188114	150.1009423	143.957047
PF-4708671	27.32799735	34.8348806	92.00950242	54.73726274	48.01343271	52.00382455	34.72699696	48.63244975	46.93783232	40.36355891	26.17788784	69.2983486	45.51873861	28.99792367
Tozasertib	30.4675034	23.92864413	26.37150939	15.51251942	9.428873797	14.20017056	14.84948499	28.65556226	10.61544409	16.75469641	21.05644262	6.933402939	16.71302202	9.0073797
GSK1904529A	59.58316068	53.22980812	126.779241	62.9145836	57.65573729	63.94640264	63.24433103	90.85260909	84.39810135	53.21058117	74.40311329	23.61258344	80.96578909	89.76491316
BMS-536924	7.175773354	6.984945366	10.08736931	9.383330213	8.112083189	11.74934812	7.555530552	7.440170884	7.901672963	8.831009769	7.046086802	10.78880709	6.055816794	8.707451673
Oxaliplatin	29.84064974	32.320361	95.69822092	32.79438281	87.71249538	61.06437354	68.05398921	22.39068813	46.68048257	48.00882124	55.80208544	38.11144486	18.26241777	56.3382947
Irinotecan	16.85522596	12.68092228	12.7009067	11.27882069	38.19853796	10.72042363	30.82345284	14.27706038	6.422421517	14.95198165	29.99539564	26.39150212	7.181358006	19.895202
BI-2536	4.634280053	2.515546305	1.14879418	1.706202341	0.329687976	0.920965278	1.512469147	2.280100765	0.480525398	0.892861197	3.365901524	0.443849856	0.833023469	0.853178894
Sorafenib	12.95921083	9.17286365	43.19778146	9.071486167	15.05421432	21.07322295	14.23084347	15.64752088	9.40852457	17.65110288	11.5578404	7.066912697	10.79611772	15.43074917
Rapamycin	0.069247206	0.096217054	0.317129928	0.063545367	0.205100094	0.131772957	0.153509931	0.129716443	0.093190284	0.161865496	0.146040378	0.085022649	0.085926333	0.095673609
Crizotinib	29.45003427	19.88022238	19.19791601	35.13061334	11.47667286	23.73543204	18.92749971	22.80204841	22.77828863	23.25954642	21.46501952	20.64015867	24.63712559	22.64262225
Paclitaxel	0.045366747	0.064946555	0.162170852	0.055642892	0.022269494	0.080965632	0.030062129	0.063231348	0.050081111	0.069445173	0.16215507	0.023561979	0.043462373	0.099953536
Dasatinib	6.114972474	3.252635297	9.468163912	4.038394253	8.415385468	3.284863804	4.237614943	2.589636423	16.41446493	4.530154076	5.03612478	25.84079132	4.920620896	8.054024193
5-Fluorouracil	41.4341756	58.73702924	294.0397042	84.10010318	163.4630529	144.5999861	128.457441	108.9954619	147.9443031	102.8202811	82.76414142	264.4765284	99.50116114	202.1891651
Obatoclax Mesylate	3.52268848	3.923384052	3.366087364	4.021559087	5.078267177	4.472742196	3.705311639	2.60242123	5.464789061	5.057413632	5.556783088	4.210525426	3.519025234	4.386278809
PD0325901	1.790672279	0.569164407	1.693482725	1.708130528	3.675445267	2.932981983	2.380427778	1.045645581	1.496100648	2.37384633	0.846960671	3.293402482	0.900847411	2.108548633
AZD8055	0.736646504	0.830620896	0.983845073	0.794359154	0.908016592	0.850678415	0.729421479	0.733118463	0.927554134	0.844319922	0.83373606	0.876991993	0.795796273	0.739268264
Pictilisib	6.878766214	3.556739073	7.673648667	2.762315299	3.490730414	4.238046331	4.258695179	6.779845272	3.138891216	5.56310682	3.423980594	4.965517042	2.982936508	4.381334692
Dactolisib	0.204972197	0.176818558	0.62250233	0.085018457	0.500886917	0.285245567	0.140219191	0.258411311	0.16841523	0.339153454	0.270503175	0.206889394	0.107176695	0.270469047
Palbociclib	16.09608216	19.000147	104.1624724	16.19115093	58.66481724	46.19050694	23.00033017	48.55492132	75.4583846	36.00107114	58.84656821	50.02569269	22.01980513	41.59199691
MK-2206	36.05690236	14.28091422	36.26371027	26.19793047	17.1428712	20.43869326	15.78826661	34.85315428	21.49424206	25.30842118	15.44214278	14.60620101	20.09579293	20.2537773
RO-3306	26.00501888	19.96784759	19.46215745	16.8317707	14.7801127	18.57428103	16.55574757	19.30254739	11.06191644	16.53977521	20.67791692	16.98609874	12.33099757	11.11428853
Alisertib	7.402030036	5.414760867	9.539007836	7.230358697	6.07659527	5.289941689	12.96468308	5.832971897	7.111629066	3.496619617	10.31443413	3.671889952	5.347750279	16.43778166
ZM447439	31.0161796	17.48428853	18.71886047	31.59719488	12.78027832	17.38109564	18.54837149	20.24412323	13.35909167	15.30509569	13.80580822	24.5929179	17.55236394	12.55095659
PD173074	83.37255348	62.39389804	71.68656943	66.98100089	33.19350658	55.0734965	50.62991142	55.17315067	70.22121524	58.81404426	49.48911252	31.37448098	61.6451098	76.6387966
Mirin	110.0643295	90.77808914	137.616858	118.840928	140.2153909	106.2434913	92.8800206	86.7013984	107.9742173	122.3843512	83.46651498	76.92200331	137.3604826	134.5203303
Nutlin-3a (-)	158.9595845	58.769479	88.65646391	63.42572149	222.5603695	120.7223701	79.57609385	61.64379668	114.6830448	125.8914082	193.302317	228.4824304	30.97202212	160.1620707
Wee1 Inhibitor	6.283095396	7.669516792	8.799909577	5.629176709	8.306005302	10.66910184	4.862643564	5.789627681	6.574610405	7.120084506	8.103679178	1.846703393	8.20496983	9.064093007
Doramapimod	117.3031621	83.03063807	96.26046455	124.0194386	81.61031766	76.16141847	96.66378491	87.0859839	87.94393494	84.19066501	91.90160774	87.44280406	86.2751124	81.69097699
NU7441	18.02692577	15.58027087	14.42779543	16.56481114	15.13912638	15.1696979	14.8078777	14.75240406	15.10791008	14.80958435	15.87449182	16.73615423	14.30632782	15.31877572
PLX-4720	100.823424	58.52672054	134.9025175	90.65421613	127.9751961	81.69559459	77.95446803	54.54219112	117.3360038	79.41060918	69.20999642	149.1627117	57.95821715	161.1255189
Staurosporine	0.082821866	0.042747125	0.036021101	0.078581569	0.043323838	0.046054426	0.040470463	0.028518544	0.065959395	0.046077829	0.044505018	0.162254593	0.031519064	0.06291975
Afatinib	6.51613472	5.532052823	13.06587792	6.885658568	6.430863485	5.438090092	6.788703456	4.80361676	8.105088899	5.138402897	6.634507121	3.93677208	3.448257756	4.618477433
KU-55933	127.0488411	84.59004435	62.51269017	99.57893911	86.28808223	70.97437469	62.43944818	53.54675238	83.66475108	72.21382985	73.82873493	178.497622	54.13161015	86.90691457
SB216763	224.6528558	184.6146503	212.5658379	208.9205567	249.5813434	199.0913091	167.4781763	144.6733212	228.910714	234.5351126	202.8238434	190.6875509	153.623256	177.9800547
AZD7762	0.826734623	0.817876359	1.206800484	1.255810769	1.562076973	1.726618737	0.700818663	0.900134328	2.150115889	0.52827309	0.777800507	0.836362028	1.169243201	1.23334153
Axitinib	26.48130847	22.57374472	30.2379414	23.27557161	18.94905013	23.80697321	14.73551177	26.20680757	18.47640378	19.5872657	17.6354528	15.71799972	19.98162273	13.05620285
Olaparib	86.33479819	64.35385562	60.4934244	79.00071887	92.50737236	72.94598609	88.75448699	63.90551427	59.49849089	65.66296527	84.92542947	79.62916964	46.21170406	103.8584295
Nilotinib	39.35369872	33.06644349	94.99797042	34.61759616	32.6829245	52.55090547	24.0236258	48.14993352	35.51720895	31.66383765	45.62913775	10.84314606	24.73980742	27.52010443
Vorinostat	3.863063958	4.000874619	6.198771101	4.642815278	2.531724817	5.401189262	4.038283617	5.074308496	3.382291222	5.769429612	5.215038496	1.958717469	3.650169111	3.024196716
Navitoclax	6.288285391	5.438152388	47.53411395	10.27887346	8.228451776	12.70061557	24.3549765	15.48728012	2.035074012	8.614510748	13.03770422	1.385450266	7.10339566	7.881048526
Gefitinib	29.09216109	24.48121093	46.29537905	28.89938567	28.17399458	25.49853238	33.01063865	24.66774948	24.59549669	29.09781917	23.99475488	18.5873317	12.61809343	22.276176
Docetaxel	0.006428237	0.011290427	0.013807056	0.008555896	0.007563314	0.011887727	0.008205109	0.006951065	0.012228815	0.00656556	0.021823359	0.005913393	0.00801271	0.017957541
Cytarabine	5.862996969	3.377901671	7.566466929	2.826016472	17.62042793	7.184547771	6.87850035	4.546779561	3.709569528	3.92967305	9.918765311	2.386442311	2.665556672	7.559442916
Cisplatin	19.69922011	29.9533063	17.46364718	24.40714571	64.24422785	35.26500226	46.95552704	15.36995834	9.425158367	39.73527006	72.87396119	37.35475759	8.109579155	31.1437584
Vinblastine	0.012811965	0.02016496	0.044107416	0.019265946	0.014875458	0.025028484	0.010057969	0.015027623	0.030193271	0.011663311	0.050086882	0.010984662	0.01626328	0.036140163
Camptothecin	0.076830193	0.069692953	0.080067909	0.07011748	0.288153929	0.087914243	0.255964865	0.086103773	0.053325566	0.092791783	0.160293271	0.220993114	0.041150812	0.171359456

TARGET-40-PAMHYN-01A	TARGET-40-PASEBY-01A	TARGET-40-PANMIG-01A	TARGET-40-PAKFVX-01A	TARGET-40-PASNZV-01A	TARGET-40-0A4I4M-01A	TARGET-40-PAMYYJ-01A	TARGET-40-PAVECB-01A	TARGET-40-0A4I42-01A	TARGET-40-PASYUK-01A	TARGET-40-PALECC-01A	TARGET-40-PAMLKS-01A	TARGET-40-PANSEN-01A	TARGET-40-PALHRL-01A	TARGET-40-PANZHX-01A
82.27021679	94.56111328	106.7581209	54.5347132	54.84302691	72.59808567	61.89665859	134.4705325	72.55833655	42.42621183	88.98260874	33.64576477	85.88621667	47.79666205	62.1403533
100.8076126	122.3191425	121.0359182	87.34754371	63.21269195	97.45989212	108.1671261	128.2820269	140.1817097	41.1964503	136.9735684	59.38275563	106.8807803	100.9835944	87.41051629
174.0182349	251.1901209	220.0025823	195.2810978	138.6920149	237.9173583	225.3241117	266.5567264	267.1323165	131.3291443	233.7035222	179.5172771	213.3136745	218.7109438	184.5237337
36.43807358	55.91485002	55.24647469	40.9749789	25.94821809	45.13888908	31.43743212	67.63377611	81.96260991	18.76648782	59.36025512	47.66323964	41.66026598	44.01274623	28.38121
30.6296525	44.04754156	6.302631644	6.162405865	9.954583817	9.745203736	10.12921318	25.0512608	27.92050077	2.629585728	22.54810137	17.80974241	19.46654092	6.870985906	6.813797573
142.9868133	175.4398172	176.1975706	150.4568825	110.4276373	153.6380892	164.0704776	243.8714896	194.4596201	88.54830577	189.7549904	192.1136581	162.0813011	186.1595996	130.4461499
34.93141765	35.60362403	39.51679375	28.73256816	48.69206338	47.67840206	28.72486065	67.5243309	46.08762022	27.21436724	35.90700254	23.30339046	37.79059552	42.91040538	31.15656038
17.64529026	24.23958057	61.98060588	27.24563077	26.74738385	31.34626684	51.34752951	58.77432258	24.83245734	16.13018641	45.0441742	7.269466778	33.24417918	29.00347918	43.08785135
46.186856	72.63206111	88.97463683	52.17488492	38.7957685	81.93014529	42.20152862	134.4593413	70.07915764	32.6747467	68.20962898	37.37523972	61.61809656	47.5158251	39.29323502
137.8031895	182.6455479	150.1593167	105.8909432	173.4840981	94.57698798	167.9286126	162.0935649	160.9011945	91.90155315	138.5612155	76.40925935	96.98055817	43.75476584	69.3534154
8.872676576	10.40784902	9.293216392	8.75610813	7.078984761	9.070835807	10.03946343	8.778702233	8.593867045	5.277355195	12.57249391	5.471571415	7.404976263	12.76258201	8.254697267
147.7802443	177.2894904	210.11643	151.6500998	151.0256014	174.0910247	164.7092225	252.942959	173.1550209	89.45222994	146.0111113	74.54355431	221.2561767	94.37174439	123.7271209
14.59230696	21.5112893	17.42091894	14.08938728	14.71263201	19.06807519	16.67447574	29.98780213	16.59660808	11.11847914	21.94996278	11.45275054	18.46234825	16.28156598	14.24956184
17.40984005	13.1075614	23.73087302	4.219323291	10.58779559	16.88608661	22.60896017	13.22840335	22.18661728	10.78931519	9.852157055	10.65045808	19.5397011	16.47499068	6.967028789
199.7496222	300.0182657	279.4148478	256.6649577	170.9357564	334.3732282	340.146721	435.2978216	389.6320372	193.9436297	347.4548297	254.8783527	231.3260484	365.920756	229.438213
7.09464675	10.03254021	9.194168796	12.84216411	5.956169575	9.08483698	10.48077244	15.61391714	12.40188219	3.761942681	13.10701363	9.772368681	7.143038197	9.577824695	8.884792374
36.3371111	42.32728448	40.35162093	41.14764029	20.96519471	39.9314681	43.90457976	60.12243778	54.37415445	19.24729503	72.91916615	35.92549325	42.12010475	56.64252374	40.9372073
0.088025203	0.102988956	0.12383981	0.0547042	0.091007642	0.225859372	0.103313152	0.24336378	0.195775807	0.056815083	0.140829018	0.086464675	0.173592721	0.126255124	0.091426576
14.18852045	18.92559654	26.99225776	14.17055278	14.17795525	35.53867613	22.63992682	33.61934079	26.6644496	25.13380911	26.01646202	4.465542729	14.2341962	16.09955131	22.77124273
101.9163037	201.0927641	118.0317094	123.4304723	122.8112286	175.7845344	172.0162529	248.5057073	214.7400857	102.3514604	176.1034443	128.9217157	117.4870524	150.3764091	108.5770213
12.81360926	24.1459436	13.8325643	28.55167284	31.96683896	32.60831598	28.05783654	23.3579821	40.78804173	10.1234928	24.89286815	29.77907789	12.84762906	20.77099435	19.20080654
38.26974824	36.83836184	56.41133799	39.84724885	50.34035672	60.66415458	62.44755028	61.43528759	96.19695145	40.47466594	35.71460953	29.51969934	40.82562757	54.5624809	54.93181079
2.204384618	1.827217911	6.374073105	1.474152579	1.322587155	3.360461047	3.137224744	9.296788489	6.123496536	1.510318343	2.135307435	0.678397268	4.343415064	0.937157565	1.386498318
148.1253567	248.6991686	217.9048388	159.0016923	138.0831128	236.0652549	176.5779839	307.1070359	209.5584395	129.4559962	292.6875314	118.0454041	192.5323861	226.0342508	161.5441667
0.436996634	0.371021252	0.420803355	0.210285514	0.2114354	0.583321388	0.299696555	0.646845314	0.846918288	0.242340192	0.290957765	0.253860382	0.525122915	0.320030527	0.173528908
42.46075037	56.20074277	66.81932393	33.58596864	40.45963854	71.10669526	40.74621717	90.82533807	51.4371794	37.14445157	79.38073363	11.7319478	40.62237707	38.19101911	62.4297173
132.693108	146.9723629	152.8331334	163.9455804	103.9528869	172.0998252	129.0295833	190.9320253	184.9150852	63.74962598	149.0042323	151.9584167	128.9366746	166.1008598	95.26812746
64.34334635	76.87401958	44.94081715	55.74992802	49.4314941	70.14743731	32.75038242	60.55356954	80.13634483	32.53079413	61.69099804	46.70503838	49.06904414	46.65361148	41.11292147
247.7698843	276.3864438	159.8243805	244.8804887	138.0571179	249.6814328	223.7221238	248.5033335	307.073703	126.7746609	297.8935858	271.1654208	211.0426089	340.7506412	217.4790897
8.702506204	7.953104277	7.369026455	8.119448104	7.078352222	6.365173669	7.944827646	7.96032716	11.92313636	6.626685427	7.326416737	6.44879791	9.325190691	8.291174411	4.857110489
204.5787768	351.7748209	126.936987	123.2675769	146.2183732	196.5262832	158.9100888	319.1055981	357.0641261	64.78923878	294.9078935	224.5585703	209.6817366	222.2578362	107.2280957
10.33384471	15.81293445	10.31122663	8.92674487	6.579164896	13.48083585	9.565674507	23.26580131	18.65586116	4.433937535	8.343732859	5.395196177	19.70203659	9.473730193	7.573930117
391.4617918	488.5594482	590.1577735	291.1953629	338.0840748	465.9606631	376.8990544	1029.736656	696.6704986	227.9514506	415.6991416	357.7174446	539.1763728	360.6253941	282.6344264
63.21309238	110.9671998	142.4769651	49.44784343	74.93807371	111.0358324	156.2792453	190.6321685	181.3117151	75.28683447	88.60192198	35.19225598	165.9842994	45.20805006	75.62623901
1.631107283	0.850263011	3.338628386	0.44010497	1.464961698	1.572020857	1.962277646	2.073749089	2.111069038	1.11264746	1.363349094	1.629900786	2.37600431	2.01721139	0.767434424
79.96716088	75.43647818	91.22305408	96.31465386	81.76733689	78.21567801	80.96067505	99.99750848	121.9474805	48.70059277	80.40086301	71.54631389	82.51821831	69.40764081	67.64914614
27.23215635	26.011325	27.50769704	23.24247247	8.504675688	40.49859895	36.61021726	49.11738458	52.06910317	14.2363433	22.49994352	19.35949123	74.44000778	34.44153403	12.92299558
6.608561244	8.311119333	7.396207916	10.86545042	6.16815414	10.12599892	9.814762263	8.928704908	20.4454247	2.854617116	13.07411177	6.268085489	7.745051561	7.582023959	6.742667178
59.21082132	39.90120723	65.88619464	22.13274027	22.93751897	36.87792791	30.49165226	119.456258	40.49811003	17.6000517	39.14594407	13.90876148	63.53685736	38.34414145	37.37427165
0.023166873	0.021408881	0.020717336	0.012478382	0.00986555	0.031030162	0.01155018	0.050585266	0.016383619	0.008352788	0.022188968	0.007000953	0.028241764	0.008823285	0.025497643
5.208513452	16.08645395	16.45598563	4.870520968	5.57152774	18.37860146	14.4789453	34.80276931	28.94501706	4.630605822	19.84183252	2.620756444	19.60591566	6.384943727	6.588064457
174.6459058	199.1200268	180.843154	187.756961	157.8732276	208.722293	185.8501282	294.1841123	239.6624494	119.4455534	211.3988535	162.4881522	189.7355919	242.4590461	161.1890675
13.88938188	21.51365622	23.06460301	18.18928913	15.63279691	25.12168569	18.82329204	34.70216083	24.90670081	13.05720943	24.17541868	12.59313857	19.48638136	21.8844685	18.18795755
23.8372805	31.29242728	39.46717641	29.92930582	26.55126741	49.21980539	26.03402255	83.86613405	42.53059175	31.08210495	40.59437672	11.88338701	34.94981845	29.14865381	31.09677625
9.352168493	9.593453538	9.279864713	9.469191852	9.920877533	10.52534693	10.66839916	9.212211988	9.277017485	11.1080672	9.520042071	9.441042039	9.948437071	10.12400589	10.04952648
14.88931708	16.44400434	18.78639476	18.38804814	16.04801159	16.32085067	18.56096481	23.28215049	17.3484392	15.6622154	19.09659382	22.25447171	15.01025971	15.97754501	18.77081767
0.005584277	0.007415846	0.010166082	0.008169643	0.005803512	0.011572448	0.008697438	0.012352011	0.011909193	0.006244398	0.008255813	0.008222834	0.008339343	0.006402035	0.005730522
0.996111036	0.362828534	1.375865425	0.279566571	0.150851003	0.698390353	0.63523722	1.971813414	1.412526754	0.1904859	0.212983643	0.118888713	1.82580695	0.248008657	0.114039676
0.070302429	0.100988008	0.084608872	0.066420013	0.045527334	0.093842253	0.073337197	0.091260844	0.129658282	0.031096356	0.084930605	0.047022628	0.089257297	0.073198761	0.055714754
1.402488103	2.574825567	4.383932166	0.979986178	1.334093984	2.433340862	1.839872821	7.764022464	3.961818508	1.014402031	2.988149432	0.529921003	3.151513616	1.383754663	1.547541175
84.25039741	85.77772662	81.40663597	99.29547146	57.84714398	97.08382402	91.95705459	132.1851789	113.8015129	30.90412834	95.8567166	91.21935682	110.2901391	79.89722425	74.69908514
7.855641997	14.09763679	14.03929016	8.234062997	9.257215822	21.10816087	13.48073678	17.23031513	26.17159216	8.729167587	11.37815248	8.905288807	13.67903333	17.30870063	10.85566878
98.30707169	165.6255283	67.60383129	143.0865498	140.7412331	117.443988	110.0348893	115.4900887	144.4987922	33.93733639	125.6691804	87.76340482	109.7456515	93.49491793	89.58129777
38.49352932	41.55358883	59.05229121	36.88462483	37.0799738	39.66244819	47.10598805	74.29801157	48.12973935	32.68676711	42.12475052	69.49158061	48.46332926	30.93611241	42.33573619
17.21714381	19.60367411	25.94039864	31.91182656	14.54233289	22.44861453	22.59658178	42.28827705	23.39629317	15.45173517	29.79923261	35.03436351	17.18515278	19.73948479	21.71374589
51.77748079	100.0716928	95.74378754	84.0581405	78.30517856	109.5455108	101.2918515	199.1980146	116.3711462	56.43458411	133.4802458	39.05756721	81.37951893	59.9580513	84.35873452
5.754177025	7.243969631	11.75891401	5.395369989	6.583112007	14.3388493	5.253722469	14.78605153	10.56807086	6.632132001	9.961515805	3.686972837	8.166718047	9.294848123	8.215575945
38.71350502	51.91459562	52.50703199	20.53562089	27.8835301	57.56020577	56.09180217	116.2977341	69.04485783	15.50701439	39.3059095	14.86307784	110.6024085	29.71862043	21.49998324
13.48610037	18.29255234	10.19556648	8.125067577	5.76700066	31.84939915	18.67009833	17.05802214	41.20062831	9.728796796	14.94361985	7.569623285	42.16330756	9.684493578	6.048806866
1.398882203	1.747865365	1.123343452	1.774599874	1.096757702	3.239125622	1.710299223	1.187481504	1.700753431	3.58327342	1.798081732	0.879325755	1.018946187	1.880715527	2.100314827
10.55476971	22.11099743	20.20055597	12.1455468	9.63100912	16.851522	14.2126098	29.80912496	17.45747951	9.874517809	19.50283874	5.337378847	16.79759858	13.28686923	11.42745858
0.092874039	0.101203545	0.222218907	0.102387461	0.072642126	0.104777391	0.127911325	0.359948638	0.179378591	0.073493634	0.112070928	0.058907892	0.161455138	0.061252028	0.056498232
21.92845055	40.35774749	30.23872121	37.34516528	21.8816719	29.43097288	38.61636308	46.17129611	37.4038054	17.7242687	45.5492744	18.35476354	27.51727972	28.39550821	27.18503808
0.064581295	0.164720391	0.135078598	0.041299515	0.030766146	0.267915448	0.118883517	0.379709363	0.289817276	0.036989585	0.108191769	0.010015698	0.121489774	0.037059466	0.034653358
3.219548537	3.110208559	6.620407835	4.594175864	6.812117941	4.261532334	7.792837809	5.632693518	5.799264897	2.207616718	3.760826359	8.357702164	4.122846448	6.012230052	7.115397474
219.0880501	112.1495924	198.0585385	58.80081523	112.1512689	108.9902535	118.5862833	972.9070208	259.0805764	39.43490364	59.18215676	51.14072996	402.1733941	40.68425974	48.78235589
2.938682754	5.629721342	3.245707838	2.384198659	4.333678839	5.557310292	3.858815692	3.547286695	4.966230503	3.665320577	4.06302456	2.577717358	4.978229158	2.920922426	4.362617004
1.808677074	1.327705557	2.123471228	0.599718093	0.98516517	1.252355238	2.450457448	2.268490759	2.059329396	0.985117433	1.370928351	1.648315019	2.310652138	1.897255299	0.850332177
0.734533349	0.8455977	0.864504511	0.767920031	0.833977062	0.839915814	0.896491498	0.870178188	0.836545598	0.685057444	0.784250709	0.867361589	0.869001616	0.671413463	0.734183044
3.112299144	4.953779684	4.992363752	3.825323039	2.883983148	5.695228986	4.014517802	9.252124767	4.27319863	3.347901384	5.543024859	2.128432343	5.124967798	2.904130611	3.561367629
0.12272732	0.184381484	0.260544287	0.166546286	0.139756407	0.25969882	0.157331812	0.715988272	0.182453429	0.168273269	0.196274938	0.068102212	0.41945646	0.136353619	0.111431974
24.66086379	32.68273864	100.4539327	22.42945097	37.06220519	43.40628703	46.92539273	150.6624869	52.39216596	23.35585718	29.38052852	8.133751101	84.54806241	13.8153619	30.31926902
12.2043739	20.68537297	20.49000071	25.31628242	22.80634494	25.59587885	19.43806495	40.25394362	24.27832174	12.82516875	29.13554652	12.7362205	19.07882229	22.10220872	23.20999267
16.28280728	16.99892921	17.36588473	17.64661213	14.76969784	24.09999606	17.76514593	24.03941251	15.1160465	17.78553828	17.1861898	30.58967348	16.92768362	22.5352016	19.29345266
2.950996951	10.15748925	7.923791354	4.72526486	5.380288257	14.68281342	6.858203411	10.49399012	43.43281103	2.263186963	15.22784613	1.349131274	6.753232186	6.851203198	3.463604266
17.71736691	17.35899328	16.23013517	28.81904292	14.3586109	18.59751636	19.89775171	23.63519578	21.2359013	16.38571272	22.00399701	69.90636861	14.90166597	32.01772639	22.24272876
54.26227566	100.5342191	65.21379751	104.6880382	51.53331803	87.853329	48.91701066	105.9768688	89.60875968	34.5945508	99.66297772	73.6430985	47.18576528	88.01376206	63.98370667
122.4122608	209.2532191	133.8948889	75.97758197	130.5173882	172.5618978	113.6806861	143.1039134	215.9807215	64.73309052	175.9132658	91.92361956	161.8593302	110.0320778	121.9496949
93.33013428	107.7590377	142.49454	47.62257536	103.4263954	98.38341231	98.88334622	161.3873833	180.0252094	30.01117985	95.47031752	111.147763	149.2895972	216.3199266	51.05255828
6.141459736	11.900831	14.1648256	4.987313793	6.219718493	12.86116643	8.136875889	21.35037867	10.41305678	4.612746788	11.19491079	2.634590882	10.37705739	6.999782366	8.254402214
80.05594125	96.11763405	70.74304521	128.1438361	82.28852138	88.56488682	106.2118775	76.23520736	80.77763962	74.8216477	98.41634404	150.7020646	76.74136863	144.2134986	104.7403601
14.65558389	15.21793348	14.57366419	15.89814592	15.3529844	16.20114742	16.08338565	14.91084292	15.1944281	14.9173861	14.91864479	18.72147466	14.81670432	17.07167487	15.41115412
47.10807099	90.74063485	72.95145227	62.64227908	67.65602453	100.5504737	106.729351	95.21380459	165.4940927	70.06347517	100.5329379	57.2866306	82.30775916	75.25479671	59.01511264
0.052839077	0.07211183	0.044007914	0.035774484	0.04477156	0.069739496	0.07804121	0.033032446	0.11119209	0.029675643	0.056873378	0.060931831	0.060921718	0.061749481	0.036815782
4.230361361	7.143825434	5.47433634	5.06393255	5.747411269	8.769472207	6.363551592	8.483551579	12.30269253	4.115629843	8.480367529	3.809246487	3.091929323	8.554172562	7.498278973
56.15856917	70.82597589	62.33375666	72.17820081	64.45977425	88.12342062	85.51497672	84.18590142	83.70930381	55.67973865	62.02653664	233.0661459	69.78990985	107.346457	55.86168109
168.3346881	192.1003476	170.3399516	206.466344	167.2894965	192.6439636	228.6778369	198.5071995	208.3047701	102.8233517	179.7171439	296.0697079	179.65363	263.2939821	160.7400092
0.606341822	0.957443816	2.366710244	1.007362082	1.408459346	1.234624285	1.979364878	2.64555765	1.506680047	0.78863644	0.979200882	0.458313215	1.41548123	0.79560155	0.993128583
18.97299365	23.83911941	26.91014185	30.10378411	20.90745182	24.70239314	21.47831576	38.75950755	21.6186352	16.8153424	26.80885567	35.54316268	20.9472946	19.49446519	24.193819
82.81519523	103.7621236	79.7644384	63.13031474	40.02572104	119.2670722	86.34319399	119.857691	120.0941575	41.25254139	118.0032298	93.58631347	100.407246	94.76611001	65.42954968
24.77668085	53.01908243	64.98271404	36.95376569	28.41522353	55.519875	41.47073504	95.14986478	35.50667597	16.46539516	57.18507241	14.76598074	38.20102286	36.56800261	36.03868414
3.874797667	5.440125841	5.210516277	4.006462359	3.704174044	5.903004364	3.788686532	6.326309013	5.653609875	4.185671841	7.791881165	1.579741877	4.391958453	4.323093468	5.558700621
2.642985861	5.764146272	7.775968556	17.94019744	3.837624599	7.170096402	5.361024645	14.10702849	8.030039089	3.669568618	17.42764608	3.893967684	6.96871546	10.89865062	4.436527844
16.82002936	29.50044312	24.9405401	20.67479451	16.74321934	41.68588406	22.9560311	42.99662417	35.47639895	21.66739594	27.2148257	15.07756423	16.58493875	27.8850729	24.51184976
0.007561312	0.017353883	0.017128959	0.004646665	0.007534754	0.03343739	0.014244823	0.035975664	0.04500259	0.009937964	0.014933007	0.003071323	0.016869598	0.004992671	0.008054208
6.651633188	6.566384499	10.32275626	4.698127717	3.550330879	8.337213473	14.36660748	14.46793177	17.27418213	1.953973198	5.255206515	2.139799901	13.39567499	8.250731466	3.727184207
27.8858946	31.52116917	32.13427549	13.64914063	5.886057205	46.07141856	28.1836481	66.28641247	51.96142627	12.87917646	20.09613053	14.6517957	92.88703381	21.23323064	7.73516341
0.015732321	0.061113363	0.054147228	0.012712746	0.01811344	0.073335162	0.036659873	0.085193671	0.087149236	0.013046152	0.034992805	0.004514717	0.049674352	0.013283227	0.020200055
0.094360167	0.111721513	0.08193476	0.055904398	0.039892064	0.166707889	0.094082406	0.152439088	0.229572679	0.05568143	0.076700377	0.038296546	0.303992366	0.0577867	0.037112049

TARGET-40-PALKDP-01A	TARGET-40-0A4I65-01A	TARGET-40-0A4I6O-01A	TARGET-40-PALFYN-01A	TARGET-40-PATPBS-01A	TARGET-40-PAPXGT-01A	TARGET-40-0A4HLD-01A	TARGET-40-PAUYTT-01A	TARGET-40-0A4I0W-01A	TARGET-40-PARFTG-01A	TARGET-40-0A4HX8-01A	TARGET-40-PAUTYB-01A	TARGET-40-PATMXR-01A	TARGET-40-PAPKWD-01A	TARGET-40-PAPNVD-01A
75.52731981	86.46328447	113.8649541	99.4789735	74.72565894	111.082678	51.27286074	133.7475116	103.2751518	95.16388652	39.95667374	52.31682413	70.60966505	49.52145221	46.98370419
84.38289422	111.2683082	142.7924289	123.334513	85.68214014	168.4516509	64.53931352	129.9618546	123.2486668	113.6730327	52.7449124	72.51167106	72.3130126	85.4790577	86.55862033
162.7323832	234.3131298	231.5142162	249.9151987	164.8679201	237.4649306	171.5335375	249.1134178	254.6544851	205.2248058	141.9587313	161.402336	163.8583277	185.8186977	272.3409383
41.66412392	36.89600191	65.51211419	44.51935686	37.02973724	53.43859068	32.03453272	109.1100252	52.53735629	43.26274456	33.79770951	25.89036603	52.90000677	42.76138019	61.22978296
4.04215734	15.70343504	9.332380881	16.74999104	8.717937607	32.91125757	3.001481801	8.393839292	8.213237852	39.56075468	3.445220685	5.379119272	29.30816865	40.7338594	32.26683956
139.8410061	172.0887562	196.6247581	181.2048818	125.7311919	177.0434138	144.01519	200.6146193	199.8127737	175.4465234	169.8562143	130.9418425	125.5087444	172.5208795	290.5145403
71.99054103	36.05649759	56.1239597	41.31223841	46.32277407	50.55534256	24.44044403	79.02667773	52.18036537	40.84602471	35.43514984	29.13649688	53.49139785	39.71927756	73.35697992
47.31031701	36.14610272	45.42409105	54.91190108	21.19621365	42.14434088	20.61704162	61.03107005	65.76692915	28.43141902	15.1281853	17.19244	17.38112575	26.38454264	22.60955069
71.49279494	57.25614522	92.9753879	88.20610902	71.62355745	60.38619279	44.10301753	115.2648192	102.047627	59.64530836	38.23254522	34.11122088	51.16653286	39.55719679	48.59024888
116.8040814	88.87267479	134.052147	88.63791494	124.898706	209.7765637	115.2446229	203.5756119	133.939893	167.8821154	167.8818365	102.2661218	189.1421752	123.8019717	153.5437033
6.253637197	8.047764568	6.614699484	8.767904644	7.215379609	10.93650932	5.416782441	19.20611954	7.63083205	17.1585255	6.855697713	6.111642655	8.310826384	10.835929	6.84917152
155.98301	198.8896313	230.295318	164.0472631	157.7297639	145.5547996	115.313714	195.5567808	221.5369742	145.3143187	113.9641062	99.99414481	144.7344948	122.051672	108.4716339
14.77113866	15.75361189	22.64119337	23.93115113	15.79143815	23.55974126	8.938811653	23.23517266	21.23909218	22.68083296	9.329977615	14.8305956	14.16482303	18.26581051	17.33386612
30.53669149	34.91238103	25.8771146	17.40369814	13.28844603	17.09004605	42.38315034	58.33835132	21.68468579	9.190942413	23.82134145	4.568490109	22.845374	13.91928306	12.43094661
241.4054333	313.7391417	280.6921486	371.4042071	227.7885999	360.8074314	240.2330119	346.5661937	389.5087447	321.337306	213.9477085	206.4307419	228.01755	288.1274036	412.5972536
7.287779553	5.375831906	7.617019866	9.843034879	6.529419515	7.650039519	8.575872952	24.491383	10.24467288	8.436232798	2.289309201	8.165604052	6.150087507	21.91192342	11.10214642
24.28231804	22.87417653	63.0072659	49.86720499	32.20375459	44.22964261	20.83971068	63.6886545	45.52483249	38.67286426	11.59210483	39.59218634	24.92414702	54.84378193	32.97556515
0.050508316	0.125755527	0.278671431	0.09878486	0.076986463	0.142716497	0.077822287	0.375179482	0.236795892	0.090594923	0.143964733	0.046081337	0.072576516	0.126621018	0.298589215
12.44690007	25.75301192	27.85101901	26.61764778	24.60875157	21.33636132	21.35050812	57.30629369	37.1639913	33.75993102	26.45923775	14.49473148	15.19263033	12.23172732	12.41029417
70.66852977	242.502847	124.1847833	210.4145838	104.6132596	218.0019324	110.8511846	136.3331407	207.8356294	190.2671692	134.3244617	133.8157046	210.6564215	197.6846023	332.648328
26.60106231	16.88408282	11.79983833	21.09357946	23.81539691	17.39891206	8.105822589	24.17350608	12.55862294	34.91930846	4.308868718	22.92019164	25.24279886	45.43281342	18.74037016
45.40320925	77.54051239	89.2857796	60.89144066	62.64322404	62.58882378	44.8325648	106.6613827	72.40717059	80.4112603	54.00429159	45.50374585	48.73551685	59.56621365	63.09444077
2.845147133	7.118028545	4.023450506	3.474671286	2.575150171	2.540800364	4.319317963	4.454178986	8.505551896	2.014511458	1.456415452	0.689988669	2.003687752	0.985199965	0.874242996
183.8637266	185.614955	243.4516809	324.2298708	156.3127749	213.3982535	135.0523101	253.6022117	312.2392622	241.2325007	182.0780774	155.3849164	131.4205092	151.0367903	166.6451801
0.964166403	0.460584776	0.41274168	0.443588997	0.300800301	1.04527433	0.624265905	1.084080138	0.654510416	0.438782019	0.610776349	0.214254949	0.46310691	0.284917465	0.329121408
23.22118063	45.23902423	76.05210367	81.22353036	48.52734937	59.724188	35.58956729	77.29931786	82.23289594	55.58256447	26.54267329	32.02425624	32.32872134	31.22649231	29.79723373
88.82099303	154.7484047	150.1646085	162.0015257	109.5983699	170.4031306	74.11849232	218.9773437	178.5559839	193.2967194	78.56925994	135.3817012	122.5083641	179.4995947	121.3959419
62.60259631	48.16939149	69.23182862	37.28928594	38.27506538	113.2097791	34.77984099	68.81592394	43.47973141	51.88458237	23.32143507	32.98293614	46.68728565	88.99718305	84.65667786
153.4525116	185.2439694	277.5208793	344.1291168	147.229603	297.3891469	123.6740675	233.7798334	208.3390462	265.1641674	92.76692909	193.3827955	179.5443877	213.4850478	223.865188
16.28801941	6.925546087	7.439357263	5.587017975	7.99684488	9.080767643	8.31661233	14.56307092	5.411214831	6.78753734	5.856728575	4.382499561	13.57103	12.87549243	7.4706117
72.95432486	265.9145688	166.3646185	251.2745741	121.9623036	292.0805682	77.70149795	175.4056318	214.7189631	315.5133788	105.0835646	145.2372616	331.9402876	348.4819091	546.9071521
12.11264147	11.89075306	12.19669611	16.67995397	7.338945497	17.40804583	8.0157656	15.02457006	18.24217355	7.763391461	7.341195468	3.126482831	8.126147681	12.94546861	14.95057552
465.1476806	378.934398	878.1730194	569.0445777	355.0102153	512.0491338	219.0994035	698.5978019	787.0145104	513.1916037	354.7566383	269.0410756	379.8140502	401.6936744	519.585548
120.5989555	153.2101529	170.239024	110.8414957	79.42787117	124.1435737	92.10363201	232.8490803	177.9867223	124.9421233	152.8811151	40.64774886	123.1185464	124.170075	171.3159436
5.238717278	3.708670356	3.355586388	1.764709278	1.350787018	1.010361757	5.917038614	8.52690145	3.119809546	0.546660044	4.745918422	0.466850603	3.790043775	1.56764856	1.316052982
67.7063398	92.29611966	91.3646953	76.81218791	97.64902871	88.68981353	54.01137611	103.742513	88.43646279	121.1723356	59.6838611	63.99263593	86.33978497	107.6133055	78.71763183
40.95743243	28.0936712	38.68690639	47.47415691	16.93045417	38.65974991	27.5863072	41.62096016	59.37699001	29.66454397	31.16996955	10.0320624	36.62187025	15.79797568	31.61693469
9.305166117	9.725330195	11.10751679	5.677808491	7.390432907	15.64846558	5.654530673	12.72617363	8.493441456	12.05933141	6.040232774	4.95915565	15.12834566	13.39105981	12.12157422
19.6063178	41.80090511	56.80800957	82.76552714	23.74284723	39.03123234	17.24431047	46.60676535	75.58979469	28.97860876	12.51982146	13.89071987	15.98439807	25.3450186	13.61089276
0.009371863	0.013349325	0.048981394	0.032697257	0.013087716	0.015384805	0.014968723	0.015652787	0.019433507	0.007003861	0.003545423	0.005695886	0.008562983	0.007147482	0.005076087
9.348807103	14.08670324	23.25169998	12.22502689	7.943519706	23.77478251	7.268798118	69.06904963	27.14068388	11.93470231	11.05729931	7.193177272	12.44888014	8.046541921	8.918076546
167.4686938	172.5487881	223.7681905	254.5940888	136.1625728	262.1457827	119.5795927	292.5614698	262.4695505	216.70578	125.8727647	166.066721	133.6736602	190.8768076	224.1213772
17.77825147	22.96243677	29.24823557	26.89265905	18.59087817	21.67811484	16.38903383	28.68407604	30.26146348	20.49909639	17.04817996	16.77724338	13.68590712	15.69056698	19.42623074
40.74856474	33.92173646	50.04473689	55.29764397	28.53442267	33.52361904	33.80229969	67.28737903	60.48225974	34.29073413	41.30294086	28.8585667	25.87814801	22.62671386	31.28747202
10.16236936	9.990955303	9.576568875	10.75165798	9.66549465	9.787498663	12.37902871	9.664599538	10.00837063	9.82418432	10.75235431	10.22673517	10.89805187	9.90526529	10.66951277
19.05155306	19.60549295	18.24575257	16.59399536	17.16808122	25.69017118	13.16617382	21.26262666	18.24612258	20.61583386	22.02450913	16.29690136	17.16928692	28.26373069	14.6724119
0.01000581	0.011483562	0.006762105	0.006519586	0.005708329	0.006127371	0.012197287	0.015470401	0.012579674	0.00680581	0.008295977	0.006918059	0.007451192	0.008097992	0.006465453
1.912695657	1.353152725	0.563843624	1.031388917	0.375288691	1.040319229	1.136254444	1.388173829	1.349362378	0.222545109	1.177646186	0.042546938	1.364066211	0.286626755	0.439164628
0.053598375	0.079904012	0.070446438	0.103116073	0.047638808	0.072202562	0.083015489	0.120833689	0.103213796	0.075687967	0.04696225	0.037801442	0.080786356	0.062147171	0.1322931
1.605878678	2.67364357	5.352635425	2.945044674	1.753934642	2.444322691	1.386289052	5.307221601	3.851794995	2.491413877	1.1816526	1.768401305	1.531822111	0.81752493	1.095890462
112.7934813	72.57145863	104.4801032	118.157826	78.1116688	114.9503114	39.06474416	85.50430824	121.379607	100.5771844	52.28528033	36.35730993	92.16133612	96.6767022	72.69636084
11.04545712	21.06806074	13.32608789	19.02940039	11.4561349	18.12496599	17.06484748	25.95205297	20.38316412	16.6058417	17.58915401	9.846778081	12.64177512	11.93264315	14.62439008
186.5115613	117.579012	124.9291653	75.14278572	81.55764892	89.15544984	53.64513874	85.37300335	97.66177883	175.8549597	44.423649	53.84598298	98.35707126	128.1475344	59.76658737
52.5896677	64.39154299	53.00076586	34.16606145	55.03544719	53.3620894	31.81986612	72.60877311	60.18486886	48.01612402	57.28979839	33.78279886	54.95374724	67.83986248	34.12578541
17.09967409	22.43272056	15.62197567	28.48678806	12.59585141	32.25543494	8.610573921	17.03496093	32.49992328	17.84021015	8.933663045	18.5533572	9.885845901	23.65656621	6.451254965
67.65997234	101.4681198	145.0246895	119.9601453	84.87268709	91.43661515	50.05046488	76.40974137	156.9562514	99.65002166	58.8519602	58.84840529	66.12801825	60.03949094	48.00933033
6.523691017	9.291811468	18.49361393	9.472352349	6.053459761	8.121470171	13.83422102	24.17335383	14.43535768	7.361889059	12.64924542	5.637406544	6.751176964	6.791626793	9.526217676
134.5007709	66.84402295	56.82956908	48.81828599	37.32019826	111.5007323	27.08399167	99.07582282	81.47761668	34.8856817	37.01375835	17.92633567	56.9866386	41.83450878	58.32347551
25.40319129	21.65794445	14.54846539	22.33218222	10.25438696	43.57840274	12.56130232	17.0575137	23.00553305	19.52583902	11.7802856	3.405264182	30.73936983	9.858149968	13.20432305
0.490744886	1.397269716	0.742616731	3.325045153	0.76895006	3.104355632	1.180735597	0.741883876	1.016281296	1.052613157	1.246038267	1.641243007	0.59241979	1.010391679	1.19342502
14.73280183	16.77658019	25.35019073	23.87688362	16.91167774	16.07572708	15.47572802	30.61765796	21.66592477	14.0757507	10.36411323	8.62511693	9.801820898	8.607286895	15.18618224
0.16831048	0.240696941	0.180497531	0.132596719	0.091685862	0.097965516	0.196542841	0.284885809	0.352741877	0.083481911	0.199534486	0.043794129	0.099071918	0.12903051	0.075202278
32.81958442	31.63699992	19.49971859	41.55297813	23.57603806	54.41490288	15.61799027	31.22880322	31.25383912	38.0854208	18.33501479	21.67818009	31.68161825	30.56963393	37.21205452
0.061308126	0.128781175	0.118998578	0.149979428	0.052952447	0.145238261	0.053518274	0.275167011	0.379522196	0.104530539	0.092233864	0.034884865	0.051928322	0.030062941	0.070124207
6.497520178	8.22195058	12.44115603	2.172317097	3.407387942	2.994055676	9.536488612	9.811188106	5.10166324	3.902174859	12.08691644	3.667172518	6.077863549	11.29061155	24.75495961
337.2345217	189.3057916	326.524767	147.6738371	98.36716162	241.5514128	71.33381596	392.2095785	411.2327992	87.34913605	181.7631847	40.59310624	116.248926	161.7474073	121.5135448
4.720760347	5.494451941	4.962858173	3.652371663	4.3972312	5.693533478	3.625842619	5.102490193	3.506839145	3.329445133	4.303517258	2.464133241	4.816931519	4.157404483	6.151711098
2.689146494	3.532918629	2.693745732	2.25419771	1.262260261	1.910650602	3.217480275	4.139481968	2.716107009	1.3060116	2.624372145	0.545447177	2.577261463	1.904548825	1.55398813
0.936567251	0.892102776	0.829965792	0.677569366	0.775780897	0.797781955	0.770304633	0.888408596	0.840050601	0.74594038	0.953776795	0.771449467	0.783343505	1.013511972	0.731699723
5.06438071	2.926431175	6.098556461	5.029726009	4.309131323	3.01302005	6.394472713	11.15080096	7.076518749	3.018952394	3.006018591	2.500121013	2.161365984	3.885409693	5.162505101
0.528635921	0.178161621	0.476925137	0.194770544	0.197795788	0.188680723	0.404824605	0.468439114	0.509090636	0.091628276	0.262514725	0.089594319	0.131373611	0.147032081	0.218457068
58.74939462	45.80562832	93.74486027	24.97815536	42.04663945	48.47412367	75.21574327	94.58229524	105.400794	20.90212041	118.8646386	14.34825234	44.53894053	37.09023464	26.8070369
24.50862631	15.68761312	21.35626591	23.7116975	21.54297875	14.40321934	15.55159711	38.86716115	26.13007989	27.2552019	8.547869187	17.10730536	15.06269133	30.42029216	17.49980394
14.08783932	21.45363791	15.82277057	24.26006142	11.75018266	17.48901699	14.57986557	18.85051939	21.34156984	14.07134657	20.55382946	19.17352305	10.40852766	18.97189039	13.31886935
11.61627192	12.98056133	9.83345402	7.89157355	8.658929209	28.70763399	4.138879331	20.36518481	8.396607277	7.159838152	2.348555141	4.620737729	11.05418466	6.690236132	5.946808871
17.46610615	18.05754155	16.52171638	25.25023484	13.17663993	27.08999674	10.67276835	18.71781832	19.15656831	24.80906083	12.32135337	19.62336759	12.34402103	30.12856079	15.36263825
64.89894166	41.50113922	91.94572592	92.33968936	41.14591726	71.32536574	50.18227903	96.08435782	81.72856179	84.13152044	34.48348424	61.32962048	40.14472862	77.97437175	78.39594696
122.5724334	162.7289892	137.6201331	123.8515286	177.3966817	256.7885918	76.85413903	146.9295029	110.5104367	110.6100266	61.88170388	58.70196498	124.0053892	149.1484526	110.6138137
270.9315212	85.54837742	96.35642345	110.5005438	123.375266	184.1611224	174.8273592	126.676644	77.32301816	52.34096476	143.459863	67.94532111	76.94725524	257.928228	258.9973528
7.243832332	10.53067904	17.74089759	15.56652721	5.758015995	11.75186642	5.218058293	19.43471785	14.1727815	10.57510272	4.845680096	7.41322658	5.209850237	3.630776549	7.008319949
96.96789011	81.57410908	73.81933716	106.4963392	77.8826233	103.8905152	78.70269264	90.71893869	73.31461828	106.5786576	64.97766182	103.2121121	78.10498999	116.5811557	89.82811612
14.72392326	15.68692148	14.4417435	15.60729514	14.12416282	15.72060246	15.45491802	15.44637483	14.69602084	15.11889051	16.34999165	16.0889008	14.27332139	17.19417772	16.77563716
114.6344785	81.53405497	120.3084827	87.84670317	59.14122018	147.9978809	78.19813972	174.6790045	107.3421711	112.6266173	83.97936174	51.10281642	99.89912007	132.402023	238.8269797
0.023687206	0.062859871	0.105803175	0.044295147	0.031661876	0.073500454	0.103791545	0.113094021	0.04118553	0.046011203	0.076161498	0.026388907	0.055884529	0.084818051	0.136918054
3.56929059	9.904643791	6.018177471	9.980307325	5.098337928	8.443955358	5.609747911	15.24471404	8.828936409	8.19816684	7.0382697	6.348109105	6.617334662	6.992052462	4.915945047
81.99602359	83.49570209	54.86609269	65.48921444	49.45747551	87.68387062	71.29132287	93.16078571	64.86981264	71.78792193	126.6235883	85.10646647	61.70299892	145.3965241	128.8371205
225.3436496	246.6717743	150.698231	172.7612393	154.8656122	178.2620187	150.2090799	253.0638791	172.5713793	176.5911159	225.9257837	196.8010007	185.9633345	249.5447	159.1996971
1.843490631	1.748398426	3.161810226	1.564418371	1.122348326	1.318047687	1.553512991	2.670924935	2.224791855	1.142242519	1.167741792	0.717618122	0.712458186	0.837489051	1.272968796
27.86386801	25.48540861	19.29843614	25.28371068	17.18118857	26.02679664	8.385073999	29.5252421	35.28827203	23.72006975	20.26869721	21.48760102	15.43055975	27.21864632	10.134662
104.1276308	62.48079611	106.7883627	105.5011421	61.13955302	144.1946183	59.83428488	113.1546077	110.5693594	100.1105715	59.86982829	39.69103354	88.95804361	60.71454809	105.6887696
33.6716377	47.00000178	59.02792167	57.29457357	27.70007638	31.76163205	44.38563862	90.86781813	76.38706361	43.20961331	46.1759803	20.50307815	18.42155167	26.92745802	21.09347092
4.437082215	4.482783463	5.125912042	5.687791431	4.377961039	5.003983589	4.289440646	9.647712222	7.197201892	6.295265468	4.099631643	4.140874695	3.353520184	2.071272859	2.622648449
11.69330254	2.682105164	18.93530487	15.30870751	5.321917816	2.110056236	8.453601644	9.687667715	23.19063764	5.303453945	1.211091543	2.205853786	2.137367573	3.86388027	1.792369699
18.56663832	41.65996665	22.33100786	39.47598716	19.9969322	36.87359045	25.55424144	51.58276391	43.601283	38.19556352	33.23759504	24.00017793	19.22495232	22.57459176	28.01365556
0.013230268	0.016759648	0.015166039	0.013757718	0.010182956	0.029673372	0.011792992	0.045724326	0.026864967	0.012527189	0.020172437	0.005951506	0.013363355	0.006568837	0.015481681
11.60577436	11.3416628	9.676010883	7.999831856	6.023325382	8.901418198	7.276239257	19.79207728	9.918527538	5.267223036	6.812058756	1.795554013	9.666041464	4.05086654	5.029663731
45.50459165	29.18138702	25.48490874	41.27757133	12.02774858	51.06539617	32.87994527	58.06168086	81.11114076	25.30426108	35.89147954	6.435463893	44.78378244	15.0397858	45.4211041
0.019010476	0.035376097	0.048563817	0.033482753	0.021903224	0.092513874	0.01544288	0.211534457	0.069154607	0.032668837	0.031403239	0.012237298	0.022919158	0.017140343	0.024704525
0.174756423	0.175773628	0.11218235	0.141937928	0.063737331	0.251052697	0.097631522	0.115660071	0.251587987	0.073527321	0.106929522	0.031880138	0.196633335	0.065397299	0.078289155

TARGET-40-PATMPU-01A	TARGET-40-PAPIJR-01A	TARGET-40-PAMEKS-01A	TARGET-40-PAVDTY-01A	TARGET-40-PANGPE-01A	TARGET-40-PAMJXS-01A	TARGET-40-PAKZZK-01A	TARGET-40-PALWWX-01A	TARGET-40-PASKZZ-01A	TARGET-40-0A4HXS-01A	TARGET-40-PATEEM-01A	TARGET-40-0A4I0Q-01A	TARGET-40-PALKGN-01A	TARGET-40-PASRNE-01A	TARGET-40-PARGTM-01A
49.70883142	81.40742055	78.71763716	75.27691857	95.88238802	71.20276309	60.10916704	59.91208494	82.06807311	62.20529938	76.14715422	76.68998222	54.33261067	59.37996418	85.2641105
41.24357032	116.4893821	74.92782244	98.8805221	107.1455442	93.40261477	79.97245659	121.4289331	69.3466877	56.43137015	130.4971112	101.1918337	118.4471807	52.13666639	125.5547543
141.2560758	205.8361937	159.8588682	184.7898084	198.7082441	181.3436924	157.4006331	167.519796	166.6580941	153.252111	256.9133805	204.8415257	255.0824696	128.9516389	186.7367771
27.8170476	42.0071971	40.61981952	46.63004326	50.07693519	45.04401292	30.50358826	37.29706374	38.37164841	33.25326218	60.50569663	46.58924546	30.6448291	32.11576039	54.51309372
4.654577712	11.75521121	2.481262126	7.010794126	11.14138729	10.53441702	6.593910362	3.503638235	4.643683982	2.708811546	21.14972857	8.106618035	13.35740535	5.039467311	12.45437111
101.0677691	156.9676249	121.6827572	126.1317883	155.6600753	166.6085826	114.2685212	147.4895104	136.0100788	126.6212176	234.4762318	189.770673	186.593061	106.3327073	143.4552835
31.58023091	35.58443327	56.19325596	32.3703271	46.80246314	47.89782029	49.43675966	55.36462084	31.13864702	23.72334685	81.08912023	45.21951303	33.55597491	35.74728464	43.08837447
10.62113663	18.51739516	39.39423486	23.38544008	37.94107476	22.02337494	23.96907759	44.2446479	22.6699354	26.88797311	77.57407859	42.38607947	30.2007725	19.61323978	24.56772853
42.24726817	57.97215529	67.21557487	69.98961554	82.08780679	51.84261261	45.88274495	44.26404554	83.5392798	38.69606297	43.56573946	65.62232909	48.74908175	50.59809829	73.19979658
125.964742	126.6084605	109.188323	103.5923102	158.65692	89.59958189	86.59207646	86.40269589	150.8505222	95.17626632	308.5378164	128.7750597	80.47664254	153.410056	124.5357602
5.98953998	7.610331597	6.83772002	8.277552741	7.721819692	10.64007406	9.520600668	8.349342998	5.664543167	8.329677499	23.01806958	8.734450607	13.99914381	6.366644555	11.02769943
155.4105994	143.494998	169.6550658	161.0405747	184.3383983	138.4881691	137.6961699	127.1134117	184.4098967	105.4970773	121.7864829	150.9326154	125.8351013	142.6280437	197.2293265
12.76600302	13.80817428	14.38680934	17.4856783	19.50027013	15.4880577	13.20031611	13.14150703	14.69385923	16.12457111	18.00229327	18.23732362	17.50478535	12.62703766	18.20957341
9.798923494	3.122426946	22.94872905	8.801633148	11.63004997	10.51387611	18.88711734	14.99002347	11.00415978	8.393875744	44.73044406	20.78363289	7.938198154	7.672555233	6.453441186
189.2585759	262.6412232	175.968075	198.7033963	301.5818026	249.4310589	206.2902363	200.6413568	189.5622893	143.4351036	370.7679851	246.9399649	321.0821956	181.7290932	209.7863827
2.95932688	7.411294179	2.713084729	18.22592292	6.323716228	5.841459548	5.539461138	25.47008765	5.130066827	3.915582285	17.29976745	3.69560442	9.426780843	7.872648435	7.291885704
14.07517674	40.60558054	18.55641802	65.52278914	35.06835773	28.08656944	28.18008579	95.53275852	22.42261953	19.86820757	66.96979884	20.12579757	57.97315099	28.5772948	47.58805992
0.043964432	0.06622126	0.086249269	0.056024823	0.093754809	0.064628448	0.066916226	0.142663381	0.093345946	0.084125459	0.311638808	0.151865512	0.148197908	0.069983235	0.068200221
9.375220288	17.66513187	11.50233064	20.10278919	19.13566152	21.08006089	23.77221506	26.41166333	17.82955729	9.740779952	75.46069175	19.54561069	20.35094655	19.57839135	21.16072609
75.31833971	178.6117425	110.0192155	124.3399987	137.5850015	118.8457192	155.0515208	88.34762481	110.8834015	111.9084871	236.3103251	149.5421986	170.2494015	89.07032557	126.183343
14.46441197	18.04034771	15.79891463	43.20922621	14.61999107	21.48969406	16.88805559	26.68153853	9.667674753	14.09300797	34.24269282	17.87726841	24.21176926	19.6641451	17.95754238
31.66588587	43.82077486	53.11560437	39.8258688	47.85589943	52.12586264	47.94060762	55.58830503	57.58836189	30.60097426	152.3083558	65.5873906	33.48401151	59.20523834	63.58737814
1.38567398	1.423014487	2.513779201	1.222434343	3.803469591	2.095612003	1.486121609	1.290534641	3.82172621	2.041142217	2.128763341	3.752282832	1.153706154	1.042966325	2.166935651
116.5238287	178.6349924	132.1188303	142.315158	222.3399348	176.4722206	132.8490471	165.4681263	174.1998396	155.4762452	192.3763876	195.235512	258.6411776	120.2578756	158.293269
0.16177187	0.251158843	0.211176725	0.201531175	0.327776364	0.38025109	0.319769011	0.318060833	0.298744821	0.225706883	0.654219216	0.284143733	0.231400128	0.225013569	0.435942563
24.84842842	42.00137135	46.89809943	53.98930167	56.65034065	37.3746295	29.84228217	82.43347182	38.28880991	37.45842985	80.93684981	38.61798337	56.8081507	45.11221951	64.40739413
116.6536877	113.6308051	104.3968787	144.5622144	116.7969427	113.7448977	96.85593956	92.93692172	103.7847113	89.3035177	145.4084896	147.231407	170.0955474	97.43617142	144.2239413
48.0865573	51.45246536	49.19153411	33.78020885	50.81229188	47.5010065	38.32730051	40.68118416	32.12659425	29.19996795	97.03419966	52.21322387	27.84692717	45.5154435	55.61787167
148.2030832	218.4050725	103.0820773	201.5867718	227.9728477	221.4562278	175.0515279	208.2792549	128.8618231	158.9190733	248.3410581	191.826353	258.791302	149.7273533	191.2741201
10.79270954	6.972635868	7.583174473	7.582392151	7.834313471	8.653061978	6.306210005	8.748759507	8.578850404	5.453973972	14.56191851	9.183227359	4.284810482	7.185128076	7.031499139
100.2614814	218.8071751	73.30841824	106.6295252	183.874359	176.2955539	156.5135596	124.8187465	125.8518024	138.5552129	222.752532	191.3965863	212.4159705	116.942473	132.5565569
7.984759541	6.836554424	8.226320829	6.751677221	11.60272964	12.47473452	6.90565879	11.24892115	6.781892917	4.292864237	15.60819266	8.08614868	10.26207393	4.784982309	8.428441036
304.3798816	347.572697	420.9234329	433.804769	491.7189574	324.4941893	338.4865588	370.4596045	390.776046	322.4726125	408.4315829	491.2106994	359.9180381	256.1084411	344.6944319
57.66278656	43.03454971	106.6031699	113.537893	116.328389	74.2583596	95.34802199	98.86857118	84.20453444	120.7907497	187.4530768	85.42473886	75.02187078	81.83316101	95.21263773
1.678810782	0.233680656	4.420986865	0.607209219	1.471880782	1.340347169	2.045467525	3.098875797	1.261297754	1.428405153	5.0744045	3.022323424	0.582320098	1.56312771	0.56051281
88.62245203	67.62320047	82.36021082	86.83882047	85.46333052	86.32563666	67.24493839	58.4635812	71.684769	58.85642463	97.32202018	94.81365086	56.58225355	93.26215309	97.97119492
11.89295161	25.39548495	21.79210336	12.47513636	19.7042692	25.48837544	22.33221189	35.41441264	21.438135	11.74674041	35.55560047	23.26880575	33.34928767	10.7013074	31.57918697
7.640111736	5.600213334	6.129327171	6.982354109	8.805853469	7.22459689	5.79618722	5.298716667	5.552240283	2.591236813	15.70383824	5.905260654	8.161417935	6.382012967	10.58490941
19.27254692	21.24560591	30.5774862	42.20371956	47.11972976	27.51685489	17.7500301	23.95502375	32.21832905	20.3270168	33.8900153	47.95481641	36.63368353	16.89135809	22.07769116
0.005086002	0.013466889	0.010751862	0.016297977	0.020859466	0.018363987	0.017371417	0.023307301	0.013054494	0.008651129	0.003978301	0.007541995	0.028406821	0.006940709	0.015839984
5.24386737	8.327084866	4.007358031	10.48138403	14.47948202	8.102399505	8.691713721	4.363850843	10.51965067	6.145973373	13.18134803	7.855491923	10.00820056	6.905134	5.996248147
108.9524251	163.4329912	108.2885107	165.2720718	242.315508	147.3943122	123.2671235	162.5990075	141.8788271	136.0087334	198.681424	159.9976033	234.0225912	117.7027216	144.6364063
11.70321122	16.94637022	15.19948104	19.1265287	23.57528981	19.59591879	16.24312875	19.95965889	18.1947373	14.03714175	16.74030893	18.62827179	22.04662796	14.57843282	17.81901262
24.24256084	37.51585745	33.60316725	30.42233682	39.15619968	33.44941891	41.53200101	42.80551258	44.34256563	26.80540064	39.11463934	38.62868416	42.89819238	36.80875604	27.97399292
9.711530875	10.25742444	8.644010854	9.249339506	9.587521803	9.830291739	10.42532212	11.13696473	9.944848888	8.712401301	9.967113639	9.46575838	11.29027696	9.757270993	9.490420183
14.55346661	17.59963023	20.71043311	17.46570724	18.04630834	16.82653109	13.52233609	18.33737555	19.03243703	22.52807723	18.82024785	18.73216101	15.41710558	15.77790752	17.96277966
0.006035401	0.007284452	0.004884536	0.005727333	0.008075168	0.006438258	0.007576464	0.005025887	0.007508715	0.004661692	0.008073613	0.007223694	0.005426817	0.006712375	0.006244793
0.297155962	0.235588047	0.634102793	0.323330585	0.70085225	0.513308029	0.418630574	0.550067527	0.40030569	0.097205515	0.77444599	0.191935132	0.305738876	0.305066332	0.748967235
0.04648795	0.055981981	0.041173281	0.063543191	0.081738083	0.06537257	0.062220124	0.060272895	0.04386456	0.030266688	0.069420717	0.048546929	0.065016277	0.039710892	0.065012796
0.672715114	1.309782104	1.134814784	1.910576788	2.281783241	1.251680236	1.643848303	1.307011558	2.775480645	1.579105954	3.005439166	2.023685626	1.26130071	1.069334472	1.167698901
51.07329477	81.64421136	107.6040045	79.43963989	91.59488554	93.80735311	60.73734303	79.23322609	60.63951234	32.28586296	89.31556606	68.33742244	112.6711694	37.78799083	119.7101928
9.656551453	14.5367326	11.58236316	13.07095951	12.58495447	14.56395522	12.12755166	10.23849812	13.09251201	17.57703772	19.99872292	14.42862571	12.74453706	11.52217524	16.35081044
107.0474577	81.84310336	102.6067387	144.5185396	165.5538303	113.6744129	102.2315728	76.91929718	65.27628613	60.32381228	139.7263176	96.81954033	91.25618588	69.85291576	153.8488561
40.25165657	40.64437365	88.51507125	51.37196093	52.36975153	39.27075127	36.60964649	42.36460177	59.17842398	53.18855035	58.61512169	62.30380796	32.28128188	38.11187252	45.19885515
12.76781254	29.46904278	19.03359578	32.6379378	20.27324458	15.81528711	11.49702978	19.15726946	15.86014934	26.80802851	8.33531855	15.43588125	30.16132507	10.33023678	17.15418972
58.52225962	85.6145366	79.45658087	110.6915066	100.775027	70.09401732	60.67432157	72.67177487	85.18828043	55.00835935	59.69916981	87.72082449	82.54603513	78.5471983	108.9562227
4.415836772	6.498333965	6.084063206	7.374242694	8.345309932	5.818279289	7.352765707	11.04741189	6.63780144	7.802852876	16.78928246	7.345272282	7.577868951	7.107648767	8.829238304
29.68738886	35.490963	80.02551083	34.15901283	55.58244552	47.70096427	40.03080089	30.75367665	45.64167188	27.69691033	117.791112	34.02472067	42.30868078	42.86585624	47.98527208
9.068322687	15.94136607	9.671560212	6.436235776	14.62597154	13.69670854	10.63090754	9.81427068	10.22085551	6.002608029	20.49252324	9.261684045	13.21191952	9.846263408	20.11285378
0.52748893	2.500378298	0.443538392	1.67023247	1.014259106	1.534371841	0.927790626	1.733859792	0.928323425	1.859295677	0.884213098	0.799786846	4.211500135	0.948780777	1.108867452
9.328350643	11.66602592	13.94267165	12.87548491	19.3755569	17.87434978	10.86139436	17.52930408	16.61630167	15.43301833	15.66058069	16.41532013	11.7987942	11.62620232	14.95419436
0.095365598	0.142094738	0.094392959	0.087549066	0.122357962	0.108783353	0.087464632	0.145305366	0.188456582	0.065735172	0.073174672	0.136047619	0.080114285	0.091453504	0.076310004
16.59764511	19.9249446	18.67579986	25.66044879	28.67452283	31.32210098	20.38430849	18.21938704	16.86750358	20.74614315	53.18269026	14.99432614	29.21396823	14.63763873	26.46429013
0.019549663	0.063687256	0.018610736	0.07864253	0.095473092	0.055068715	0.060729356	0.027388555	0.07573134	0.020494377	0.120024093	0.030110096	0.154605703	0.032464188	0.041371458
2.631197231	2.636715535	2.087738978	2.546502716	5.574233994	4.53680486	5.275528177	7.235522074	4.213093359	4.236353375	16.18220229	4.781123876	4.049082798	3.198692118	3.738959369
99.54734216	53.23545803	299.8251104	74.54713425	139.8610425	95.48833656	83.27556984	71.72185436	139.8240452	27.51733212	356.1362518	79.0575184	116.9345718	69.57303756	121.6628124
4.25311519	3.698112516	3.534694704	3.51533818	4.719954413	3.904020148	2.905026581	3.311313477	3.262915941	4.128399895	4.954164413	3.680768283	3.52450839	3.23663212	4.281881759
1.038952206	0.376323268	2.40434879	1.191951879	1.771189767	1.412016466	1.553224149	2.118986756	1.152794397	1.269548393	4.621526938	2.468399649	1.502918903	0.802508984	0.92153943
0.850555656	0.794760229	0.808578422	0.832221582	0.788406996	0.788218022	0.772288606	0.839573027	0.817254331	0.875294934	0.81772621	0.765950825	0.791530507	0.753613698	0.813985665
1.780958724	4.519956347	2.438211586	3.869671402	3.895402489	3.800219082	3.330631268	11.25358673	4.228506781	3.068241311	6.071255713	2.618539678	3.204876336	4.114899494	2.910037514
0.128061955	0.277150048	0.156315153	0.125877357	0.197506215	0.173268188	0.182265763	0.332638419	0.328486528	0.148424277	0.225977362	0.19811676	0.139740293	0.134754671	0.091941892
21.48956082	21.87250992	56.08012441	26.85632457	55.34476552	33.41857729	36.94840273	65.86399033	47.77995903	20.98061997	88.52779218	31.89820578	22.09330476	27.20149519	28.92855449
10.74797417	25.47594763	12.75184623	21.64244243	20.12192424	19.88456965	18.39060497	58.47021123	14.21876852	14.42824589	30.22505688	12.56987929	19.00820934	18.91913456	14.68712856
12.40099166	19.30639037	14.91551079	21.12979195	16.2602268	14.35328191	14.03704988	21.52406737	16.55794803	23.59380021	12.52729873	15.49513857	33.54033259	13.30407814	11.43682847
4.93626549	7.174857658	5.491771715	7.366150306	5.172451899	8.800373159	5.220220683	4.667041353	5.565495887	3.50665025	18.69940138	4.032982151	4.674136969	5.234293443	11.08029557
14.07861608	24.12523087	15.02641365	19.99214552	17.22486826	18.40715324	11.84578006	18.61675711	15.02607832	23.23282294	14.41759442	19.4898113	23.63204569	13.874708	21.68400365
34.95732754	84.3311888	30.986765	67.40411685	63.22326849	49.44343865	38.07320775	52.69223563	47.26375024	31.2795489	48.61394724	60.87620018	77.20246879	52.27738734	65.67632879
114.5977772	89.21243487	116.5463215	103.3150843	171.0447848	107.6383149	97.57181302	86.34058247	88.21056378	91.49563435	197.6586799	117.6837027	58.73287002	96.44447031	125.1018706
73.81046697	86.13336397	91.97125124	55.26552866	121.3312311	128.5553273	113.6607213	236.3330064	63.77531254	61.09873531	216.9728653	40.35947471	91.80250794	92.74120772	97.21104056
2.948727605	5.655217281	4.474426296	6.063050337	11.61150993	4.759408794	7.089287636	4.205365628	9.026993817	7.807567878	12.69885941	11.19771151	7.55644972	3.543863497	3.783606269
76.4056313	91.599833	74.73143762	88.47582496	83.9191625	109.6014322	78.4166035	125.0998007	63.61541785	86.5617092	85.73293064	79.73155372	120.7310653	75.07184424	97.6126119
14.31009992	15.59427096	13.82548313	15.38258763	14.55225107	15.20048002	14.86138965	15.95992863	14.52972594	15.99684155	15.1065269	14.58538971	17.35800285	14.57596811	14.17551654
71.41905957	104.9733393	53.69680092	67.50348469	100.1732878	81.19113736	71.46998301	90.46734216	45.68428365	52.92735995	183.5314659	83.8662779	66.31431305	76.60753614	95.49677907
0.023726633	0.039069062	0.012009611	0.036936767	0.044314163	0.048232983	0.034685251	0.055987059	0.035236207	0.048418627	0.028862882	0.038077254	0.050424033	0.028859317	0.034257506
3.926310753	6.003179405	4.347581298	8.34498298	6.467120966	5.694102243	5.416884843	6.378349735	6.040871838	6.240634004	12.43779105	5.011316884	6.407810708	6.320566647	5.394412694
62.43688747	81.08370764	65.39060306	80.24643247	63.08950343	62.83660501	61.5033353	73.40367727	65.0944044	97.46641173	75.87599912	60.13170482	110.9742789	58.87201896	50.59935577
184.9843825	168.5476173	171.6807728	209.4506348	189.9148988	209.5704231	157.188825	232.0935082	181.6476513	181.7813554	171.4973198	146.2187211	285.4985034	149.689529	184.3081456
0.399428854	0.489074255	0.990229874	0.650152524	1.360590141	0.746472318	0.771328969	1.054317061	1.164710701	0.772829492	2.35855735	1.803506243	0.718781117	0.59293006	0.686052582
19.88345306	24.65950693	29.57537984	29.52477536	29.01192749	22.27148975	14.77516534	21.52498318	22.52745028	30.72646565	14.26368589	18.56162518	24.59483693	20.11436282	21.51017618
41.60732609	99.91677429	69.62399526	52.44789956	79.94834148	71.53258973	57.67359681	73.89836891	63.16981251	42.72042516	93.21920161	87.53829584	92.78961805	46.39485418	99.03807829
14.2042064	34.22234602	19.80398964	55.02001371	65.54110678	35.0856599	26.71360547	48.26758916	35.08360073	24.90354232	28.36730423	38.57509038	44.18671338	31.18522403	28.60666888
2.237768944	3.947761277	3.768524279	3.925334698	4.943781958	5.048876937	4.325758098	5.764432713	4.182329861	3.271600488	8.696172214	4.294162018	5.563200656	3.308570196	4.680985419
2.217231087	9.416917981	8.601365343	7.348519019	8.456346652	6.351153409	4.167944159	23.47583215	8.161107287	15.32506918	7.054861014	10.27629009	9.028116452	4.225452529	7.762895379
19.11819829	26.93325983	17.24059602	24.35379303	24.71343036	24.12309689	21.82583381	21.9693843	24.1983395	19.18479569	37.85439397	22.93902267	28.12018157	22.41884455	22.63468664
0.006798558	0.010665862	0.004384124	0.009154959	0.012354903	0.00898067	0.010644297	0.004995589	0.011202768	0.00570556	0.025432413	0.005166206	0.0105194	0.008496231	0.00807446
1.359203347	4.222490132	3.151521392	3.210032574	7.014421726	6.25642524	4.676408284	5.324892568	5.393728707	3.148290885	8.176784768	5.537144006	7.274424833	3.967508179	8.566528336
11.98631705	33.35999322	14.01452813	13.10600067	20.51590151	30.58217286	22.97606751	14.56561321	15.4890794	11.74673245	24.67938014	22.74725376	24.48131889	11.94999663	29.82096704
0.013694047	0.014753248	0.008757843	0.032477999	0.038667551	0.022360383	0.02090774	0.009428223	0.028065603	0.014164822	0.032480438	0.015402895	0.024172031	0.012770872	0.013572977
0.061116774	0.080475946	0.085931016	0.061392618	0.085182206	0.077909113	0.096723276	0.06509921	0.070630284	0.046872097	0.150486154	0.066579994	0.090000814	0.068296574	0.102745592

TARGET-40-PAUVUL-01A	TARGET-40-PANGRW-01A	TARGET-40-PAVCLP-01A	TARGET-40-PATAWV-01A	TARGET-40-PARBGW-01A	TARGET-40-PANXSC-01A	TARGET-40-0A4HMC-01A	TARGET-40-PAUXPZ-01A	TARGET-40-PATJVI-01A	TARGET-40-PAMRHD-01A	TARGET-40-PALZGU-01A	TARGET-40-PANZZJ-01A	TARGET-40-PATKSS-01A	TARGET-40-PATMIF-01A	TARGET-40-PANPUM-01A
43.67927427	67.00704565	86.61764187	68.98823475	73.34304946	58.05948076	44.20205074	83.49951055	53.78288223	48.55648709	72.93303908	82.47390813	73.40517164	47.37503701	51.36754673
73.38840182	64.68517568	106.8438865	120.9276992	67.90856915	97.4635537	54.76171088	105.9340478	133.4582353	53.74368374	85.17964272	140.9138571	89.43287889	70.86014376	71.35163976
153.739376	177.1180939	177.0975542	181.3960389	209.4672165	227.4001275	172.2180869	209.2217152	217.0230072	158.5509448	189.8588378	208.3576325	186.03925	156.5492435	153.8747336
46.03618358	39.29533163	39.0614574	37.8420809	44.43696154	47.34560256	28.71935039	67.49920797	38.92834311	34.89983887	38.20606803	38.11069488	55.35977793	26.35331373	42.19106476
9.31430914	10.71532062	5.757970824	17.86369316	21.1194532	7.18363895	1.929662948	5.108359676	6.69067632	2.785042453	5.385758159	13.05114692	14.12496362	3.434498094	3.16495284
160.1939936	169.636044	135.1355837	190.0384367	155.706831	159.5894621	123.9307441	148.1275714	193.3710149	126.9248827	140.5892214	183.5993103	209.1167885	110.2227084	152.0294613
37.57992925	29.76519911	32.11986294	84.88674699	35.42547708	37.84165869	21.1352552	37.4734759	117.9387844	38.41549831	61.78887974	57.22891143	35.45051876	28.149046	27.5471494
9.992784095	40.49883798	40.57082813	26.65620158	26.83531218	18.74625485	12.349627	35.74195062	57.48791689	19.68779695	22.030263	40.90902484	36.26897098	15.08867708	21.41811946
41.28380167	73.02343123	47.06741965	70.88615806	56.73890417	55.96917179	49.96427806	78.58401438	64.96351043	51.70700837	41.75571662	67.99421035	51.65251754	44.88361518	45.09871248
74.27329569	139.0533969	81.56763407	213.8951056	108.0180076	133.6040928	70.3329083	141.5178945	158.6810371	130.314339	61.60272746	114.3657218	96.66262884	99.57034589	95.16923122
3.928116146	8.259469033	6.069295555	11.42298004	6.735094632	9.186380803	5.331052398	6.924821916	16.37391672	4.089398896	24.44203682	9.5619044	18.55711377	6.365645904	9.876478506
144.2110899	130.0471228	142.4026508	122.1476395	145.1480187	113.9429783	116.9009706	211.7830208	119.7280754	122.0255993	137.5598904	147.628001	200.0954678	116.3514679	117.096794
16.9235604	14.79190932	16.97186909	16.81170529	19.65380049	14.26231533	11.59209598	18.92152983	18.00954761	13.82103402	15.75501306	17.86787901	19.21817891	12.78603652	12.9129033
24.03469452	11.47262112	7.628908637	27.63058044	4.699250543	13.33734641	2.590772132	13.34186951	6.027383869	8.20959956	14.87857489	18.60382731	21.99378966	4.787553974	11.06366769
295.3662067	269.7185629	181.5653266	266.7460736	261.717059	308.6358715	240.3481589	229.7008789	271.3815831	233.0150754	213.9022994	270.2075295	220.2348592	183.3270735	151.9395973
13.5897917	21.61122411	3.396127694	17.82482008	7.89612544	7.757910047	5.868512168	5.122246453	23.52426413	10.19239461	7.514655717	10.42987087	6.103563222	16.66155464	1.908219372
39.16768012	65.91626041	27.29977671	62.23967773	38.28642206	34.20137833	23.76446178	20.41585466	118.1989187	45.09363279	26.27971613	44.26185511	38.18768394	62.95376514	11.84310728
0.139788889	0.094688029	0.061858455	0.100435149	0.08419129	0.112652737	0.039147684	0.061420315	0.060546621	0.06181701	0.040638388	0.090699122	0.11545261	0.101325226	0.058247182
10.39906005	49.15276236	20.48537501	32.24753032	11.07087488	17.96567189	9.887867685	14.32822052	33.12891938	26.9071581	16.28890516	29.85788992	14.99880131	18.70169796	16.26509103
125.7144476	146.3921264	148.8155462	156.2530881	213.4528292	118.9190422	76.92338717	135.2783935	148.1039189	86.35588054	90.66122613	123.8920722	176.0634026	132.2851781	96.40426461
23.02292919	19.03838466	12.72339858	31.86460226	32.20889894	22.25013935	8.863237485	14.46714142	50.2418016	13.94446094	21.65338091	24.19037477	16.06410409	33.62602628	8.709175988
66.12749141	67.83560024	53.33442646	60.85279948	48.87634624	36.15924792	13.69813675	31.66457286	79.47731764	44.09364915	46.21379549	50.15311221	88.11347264	35.00523359	41.07855341
1.010032119	2.342125885	1.688278594	1.879461051	2.687564166	1.113541448	1.264903706	2.933889965	1.228377323	1.183759796	0.884310489	3.124874513	1.297812051	1.029260734	1.116320599
125.7181272	180.5764299	172.570553	143.4230005	146.8310991	195.2984131	138.6247975	171.4859505	141.3080108	178.263106	148.1248001	210.0462915	150.8039146	129.5789006	121.6859842
0.401623996	0.308296952	0.171751571	0.471039824	0.213098408	0.347575091	0.262474848	0.504322021	0.342150548	0.277107999	0.283361966	0.416170802	0.301514729	0.23008224	0.163447553
37.83315008	62.58341582	65.63729251	38.78467615	46.83100687	27.48410463	32.95275183	25.97008722	75.63129651	45.68219034	35.9068179	54.93462908	33.40782491	49.05226048	27.16743717
150.3518388	112.066011	123.3404378	83.06627184	188.06067	116.8125996	86.57274829	146.3590908	89.97691914	102.1267078	133.756498	113.1667563	182.6048821	98.09185613	103.3382822
88.28595446	40.20577819	43.36085162	46.78645559	41.80722859	46.67491901	33.99758666	60.96976133	49.79256998	58.15245525	67.39556782	32.03551654	47.1365013	47.6829645	23.60830549
156.8720382	189.0341476	134.0207733	187.5390296	175.2239755	256.7008417	198.2699316	170.0216753	350.6383865	217.6718933	270.9900415	230.8423827	178.9158745	192.8848826	122.4091507
6.666820032	8.714997957	3.426541822	17.71009129	5.676213546	12.31878697	4.283422618	13.03760502	16.64789337	6.936546415	11.60654982	6.152251232	8.945066755	8.428345962	5.488325617
167.0984835	155.6862249	133.7095073	129.8199198	242.9778951	159.6906266	82.13679119	145.0768424	172.3841713	97.44746676	91.02382777	187.0412614	277.9328629	132.4782174	89.92811001
4.550596637	7.307621135	6.371881941	9.194628834	7.320147543	12.78263842	5.324354048	10.43269786	17.92697253	4.63138849	9.966807086	9.59353847	8.295035102	6.680248294	5.246947227
344.481281	386.1127064	306.9667407	370.525832	336.4001715	438.3354456	286.9051123	470.3501911	366.9236527	477.3756992	376.7659228	424.9530505	411.8946105	249.5474554	270.1744474
83.28213761	91.1210872	63.21983042	155.4503765	46.07732605	87.93596903	57.7467178	88.93861427	64.49799004	79.25699418	89.20345358	114.8932552	125.5926796	46.77071893	104.6412659
3.629956194	0.757966829	1.318080432	4.380951086	0.290047618	1.488184636	0.296357121	1.726490205	0.271318735	1.167427505	2.011676587	2.321392047	4.313096465	0.390259841	3.312360139
92.95953184	69.51909859	67.79870756	97.84603233	87.27288551	65.57776244	61.40199635	76.06785945	108.059375	80.26748758	84.80722335	80.18507424	119.5601588	64.54163455	87.23186802
18.32533532	16.35961556	11.3266807	32.71195644	14.7560909	24.76292579	22.65014228	33.17211012	27.86812944	13.02630798	9.648771118	27.60968385	19.12508776	18.24981048	7.381310508
9.735816388	6.894974261	5.231037937	11.67416764	10.91785199	6.810071163	4.287508008	11.23744063	7.28814326	8.233268774	6.681370713	7.948706033	6.570061646	5.405836282	5.133246806
19.10643645	32.99605986	30.64970272	32.03224621	33.31000441	31.87771984	12.84109146	33.77756302	22.01307465	14.21960808	22.42977478	46.59786119	32.34609128	17.49630948	19.17198873
0.012775075	0.011725741	0.02384346	0.006216283	0.009038726	0.006455166	0.010501989	0.011411227	0.01144701	0.007835496	0.011054109	0.011388688	0.005857909	0.015750021	0.002889167
13.00411216	9.733176718	7.770336716	9.993460326	6.910502242	9.792430153	5.161491563	25.9640704	4.755250544	10.36079462	7.34420092	11.13018616	6.35154019	4.790076485	5.670234839
179.9437998	147.2588596	137.5790177	178.6312879	183.8317783	170.2040322	143.2949109	166.9968859	172.6934386	159.9574214	165.3848668	206.0679587	160.1825435	125.244364	105.2411543
18.59601855	20.90505075	17.75300439	15.63388552	17.17581017	17.90204244	15.96278706	19.12470197	22.66277934	20.06322968	15.54681872	18.79047073	17.40752384	16.40497189	11.61696677
33.09421261	39.0102496	35.82606367	38.45825802	29.40025139	32.06993207	30.20779993	45.05532324	50.68220562	38.57553301	32.56174899	37.52730997	30.58189727	31.53442331	33.52869639
10.47615051	9.959746959	9.969103238	10.03457585	9.084628246	10.60651722	9.912568397	9.511654564	10.06994465	9.56187954	10.73385758	10.14618549	10.58347077	10.40270377	9.497009657
22.68954686	16.79236085	21.61541124	18.25477508	19.35228283	16.76686154	13.49928614	21.93117545	14.80487136	14.56060398	18.82791316	17.15545435	24.35959921	14.9540849	20.2095192
0.007400246	0.009530995	0.005343907	0.008006782	0.006767166	0.009798762	0.006711509	0.01176797	0.005269249	0.0105265	0.005956975	0.009874786	0.004899185	0.00571023	0.007705988
0.465478632	0.364244794	0.144722442	0.797393813	0.093877755	0.623416248	0.658930073	0.590345214	0.290810181	0.126644278	0.301678688	0.318440282	0.704223675	0.407521258	0.163810123
0.042308414	0.05385907	0.046069539	0.063728328	0.065202899	0.069592922	0.042418376	0.073848149	0.084357267	0.043075163	0.065788296	0.06679225	0.040337177	0.046783882	0.025815721
1.093437479	3.012525068	1.764737201	1.701058401	1.267968757	1.047816641	1.042507498	2.786415497	1.523722442	2.105725733	0.946308939	3.499065829	1.419458411	0.946757473	0.946989174
66.63042602	66.91443978	59.4928338	81.2554644	74.54095648	73.58090867	56.71504719	93.95227797	139.3044187	35.11118999	44.79344752	106.0429564	57.06400906	62.83737489	27.05014359
8.473214765	12.22193964	12.7445405	10.71230638	8.87751755	13.61427052	9.045934594	10.61220806	20.85702277	9.394255041	14.19262551	12.8630656	19.84597214	12.2478169	15.79348774
87.7302403	84.41020896	60.36167681	50.09292885	77.12554307	110.2416026	44.80530936	113.0488185	83.19383709	84.81826775	98.03490487	131.1777497	102.2463168	102.6918951	55.66505357
54.91271945	49.02739105	45.87734084	64.39078572	48.20302999	38.93606227	33.10226358	46.2818021	39.67066751	37.47801451	50.75335375	49.46595174	74.42459991	34.24638812	56.09532542
18.57261889	16.42451239	24.93543919	11.52445175	26.94525516	20.88447847	24.65607201	20.62145526	11.22179966	12.50792825	15.70695852	24.50713729	12.11075551	20.47719861	14.12853279
50.2552836	86.50291176	89.78744758	54.84101708	113.3946555	50.57528708	71.76823765	98.21194567	82.16832437	72.10568256	42.75569194	82.77766213	62.6653744	67.05925722	50.65734964
10.57720848	10.18223611	7.821457431	6.755494054	5.85027107	4.730819095	6.357476168	6.966203979	10.09988599	10.2744932	5.772540417	10.20007781	9.08585325	8.07953874	6.635918026
58.3287042	34.05665142	22.51123298	60.01455722	25.73033082	46.92054181	19.57456608	64.18779844	49.89971379	20.30067922	31.21004764	38.77861879	62.34839687	31.33460276	17.24825992
10.60617062	7.45687117	4.493250109	22.44942624	11.22026249	14.35570308	16.64987628	16.0067064	8.284076305	6.845842691	9.400834017	8.754187987	8.396921811	11.97831347	4.438817005
1.304339038	1.136885271	2.078731312	0.543939899	1.675156879	2.564839783	2.527352295	1.32020268	1.000152239	0.890421221	2.022025085	1.306403881	0.985406298	2.245551318	1.181805406
16.4079616	13.90049136	11.12523134	18.09836975	9.186080362	13.35754025	10.9106344	20.87965814	14.9552877	10.82873122	13.08597945	12.45373629	14.03967918	10.28019215	9.108383145
0.113965922	0.13150284	0.062839664	0.199441121	0.077937819	0.117347704	0.091536824	0.273138988	0.058559546	0.076554595	0.091376378	0.158283073	0.129798283	0.077026369	0.080522553
31.69231274	27.34211961	18.74140185	38.8716898	27.52536179	34.09773915	22.76564802	32.12369949	25.70366246	20.03327009	28.16923997	28.21831037	21.41057937	15.23010984	18.88567717
0.057619575	0.102812783	0.02019864	0.083761234	0.034089542	0.061711712	0.037143374	0.101261793	0.053838251	0.064790982	0.033755179	0.106240719	0.021892093	0.04131045	0.013183717
10.51408362	13.30656243	3.010920263	6.985612644	3.562074659	4.112845917	1.511129179	3.3684107	3.628454798	5.044354181	2.476679651	4.319016678	9.14015887	3.579117856	2.728240371
97.23117812	118.9517047	35.70682953	222.0491851	82.47199814	118.8431239	36.95466363	103.5926382	105.3242178	71.82418441	60.81092981	109.2967708	125.4108563	53.4643669	37.84936924
3.293509819	3.041608633	3.780826221	4.399971052	2.532345234	4.495099396	3.139928622	4.998017258	4.607709968	2.855366517	5.054661129	4.090251103	6.618646795	3.29291193	3.752494424
2.302160729	1.196555998	0.974655361	3.416901913	0.513930549	1.593906675	0.508412106	1.263483347	0.685209731	0.968441191	1.635298661	2.73787398	2.951249653	0.727572336	1.980013131
0.860933056	0.762717411	0.772722766	0.8733874	0.775937378	0.834080365	0.76197989	0.964058279	0.708770586	0.787771093	0.914159201	0.77903728	0.919905765	0.702775901	0.933366819
4.904550096	8.103970348	2.200466211	10.04172221	2.727268855	4.153065689	2.764032074	3.188326353	8.793240462	4.7006324	3.053391926	5.434060087	3.132881659	6.443194975	1.865319633
0.192511871	0.20605234	0.110533836	0.487679826	0.114758537	0.181844219	0.12891993	0.38930782	0.208797936	0.19525251	0.120614503	0.249176207	0.112724127	0.142991655	0.108168203
42.7592851	47.87737868	33.00457516	62.08321722	16.95285615	29.84337813	20.15821096	61.11799155	35.71368769	45.62725911	31.75656567	57.12577764	39.54638348	21.96465819	19.43072297
24.20002584	30.78684053	14.16586512	44.18207156	16.21822384	20.86342417	16.68833101	14.69820101	59.61208137	19.62373329	20.62098707	25.46977013	18.48784258	21.95859428	8.398755788
18.50816874	14.97636741	20.944537	14.67050186	17.70811015	19.18001147	19.6384275	18.11979486	13.65769379	15.27839223	20.91403913	20.50290887	20.59109295	17.82814456	20.93599873
12.89877992	4.232419699	4.090954649	15.50359655	7.621536147	7.266317477	5.672356121	15.79060627	11.94540753	6.259181003	8.173588774	5.023686846	6.73759532	4.908205341	3.351527087
16.53661233	16.83395617	18.96369173	16.37371765	27.96280003	19.96107821	17.99064673	15.18688525	19.67670825	14.56393017	18.2524013	21.11198997	17.16671168	19.83621501	16.35303605
61.13138895	59.82601424	55.85029834	41.80823252	73.0957196	47.07573828	51.6478349	89.60439039	65.94500462	67.0666751	63.53947479	58.12536182	39.16400532	50.41905107	26.50470728
92.81167871	95.66389683	81.30363926	172.7757212	116.0417135	123.2147474	63.32187825	111.1864958	153.6375237	94.15495791	147.2514808	118.6703516	108.0918349	92.02219665	80.94175129
169.7874668	92.55179342	45.3077252	285.5650417	36.05944565	104.1398335	67.20532359	128.8225208	376.9122362	100.9357948	106.6264632	130.9385996	113.6345049	71.01740577	66.68990386
3.523276586	8.864212245	8.54609667	6.531355671	6.122385754	5.278409914	4.061713266	16.76229743	5.302590169	7.596918865	5.398512333	15.72006288	4.759360669	4.351619603	3.804856072
87.44862577	75.57253166	99.77053323	95.91322283	93.69516737	95.02234446	79.87302474	94.65145095	114.4061052	83.80501134	110.6239054	91.97567299	88.10034732	102.6188844	87.09369283
15.60602431	14.72946749	15.78341078	14.55501375	15.77611794	15.83712478	15.49075106	15.5244375	14.99089948	14.8514189	15.96039277	15.83858017	16.30601264	14.96634496	16.15144651
74.56762935	69.31805959	55.18101738	128.2341111	74.11332112	107.5356544	77.77923345	74.12897925	70.47847172	76.58028931	91.22557226	97.21779592	72.19868122	52.48615718	51.31720922
0.04631438	0.056352696	0.028588239	0.051148953	0.030459365	0.033611913	0.02858605	0.039819279	0.027862069	0.039135246	0.038480378	0.048505503	0.062707366	0.029622975	0.025259246
6.992487465	8.961077282	6.568111869	5.100508852	5.552494512	5.649877405	3.357003931	4.110796216	9.507626117	4.76283458	6.080725616	6.024556252	8.191761428	5.572723211	4.470554732
71.19909748	58.7692592	75.01274916	64.0308439	76.6321031	96.39498995	79.41694867	74.76643957	81.28361212	80.36671186	86.32047497	81.09252849	115.1642387	58.27371004	113.7404613
202.591761	154.4326871	195.3398752	178.9967812	166.138106	196.2624034	160.5322704	207.7668336	190.7378976	172.8049143	229.1818612	183.7466091	251.5461651	167.9746891	197.1220045
0.670801988	1.657892935	0.986863976	1.145994679	0.814827912	0.521001361	0.441831903	1.363660537	1.132691807	0.901089223	0.598847989	1.726590053	0.864133305	0.410123886	0.685391703
22.814915	20.48792286	28.30794161	17.69436945	31.5433705	21.48343013	21.1717225	21.88284073	15.06716029	17.01244523	18.27932231	25.25177333	18.67772599	19.49250292	19.39987762
41.61241298	72.50686219	52.4883832	74.68597335	58.15928454	95.22915285	83.10124227	77.29247115	78.99004281	58.12939132	56.71035639	92.999624	42.56075326	58.19602977	28.53553647
26.01261001	45.05342615	42.24237937	34.85439621	26.584388	36.85988579	34.06749276	61.86640867	34.04580173	25.72792965	29.73665925	53.18967628	47.27667554	17.68195512	25.73965819
2.669245428	4.719144244	3.376161091	5.960802169	3.10527512	3.444579297	3.103686908	4.246619375	5.310848411	4.530633231	5.357849374	5.454364378	2.642142899	3.690684224	2.918202109
3.762452081	6.801735781	1.697347827	9.362630636	4.362804164	4.015253934	9.251637123	8.915284672	17.41328656	9.381110574	2.115093267	13.90888415	2.954512998	5.917875913	5.966181016
23.6760796	32.63655641	25.31104175	22.91124256	21.20948207	27.92882129	16.5803496	18.63442603	32.01357627	20.31851778	32.37577581	25.39343071	31.97473809	24.32911305	18.47840575
0.011183837	0.013436937	0.005905779	0.021644774	0.007031693	0.011577553	0.006620236	0.013972338	0.009094698	0.011872442	0.009304087	0.010676921	0.005785461	0.007521358	0.005290331
4.639396014	4.926740275	2.953997074	2.676254944	2.976171529	5.666878542	3.72648756	10.1631745	4.399465308	3.570154233	3.876151581	7.650246054	5.209470338	4.337356215	1.114766652
16.31123878	17.22377673	8.767859706	38.13250756	20.94238181	38.08590246	34.55138816	48.90095957	20.08172672	9.374383662	21.63481258	21.58210351	24.10014424	12.10643282	11.02436993
0.033499366	0.022485992	0.01956624	0.02283478	0.013817516	0.021374453	0.009861618	0.074700392	0.021532721	0.02349328	0.034000596	0.025777917	0.015925554	0.011691034	0.008913687
0.08175957	0.054617147	0.035218888	0.112676637	0.074593433	0.082720153	0.096893981	0.154570187	0.060622169	0.049567097	0.053557784	0.065052009	0.086658819	0.071135703	0.036623497

TARGET-40-PAKXLD-01A	TARGET-40-PASSLM-01A	TARGET-40-PAMTCM-01A	TARGET-40-0A4HY5-01A	TARGET-40-PAVALD-01A	TARGET-40-0A4I8U-01A	TARGET-40-0A4I9K-01A	TARGET-40-0A4I3S-01A	TARGET-40-PANVJJ-01A	TARGET-40-0A4I5B-01A
78.47664795	66.62611745	86.00585341	58.55461717	49.02648328	58.85760318	90.11409685	81.22757498	97.48205362	41.94707418
82.97473582	81.70661392	90.76258978	113.9596502	121.6001081	66.10323136	148.9755535	85.66639903	97.64964949	60.69384323
211.5645305	162.3808682	178.2711263	168.9871311	177.6834365	150.9748249	220.2823939	230.5168387	204.6709804	146.635319
63.51092992	44.84988499	37.02776553	41.64585831	34.5700506	34.89936018	42.99171923	31.5368133	34.20263548	27.57007822
7.589119596	7.01042902	15.71813747	7.139671468	10.11984082	7.105976878	7.089933098	13.22339339	6.40742707	5.685030037
184.0604829	150.9926794	176.8170272	160.0319267	190.1605657	125.1412919	173.2027198	218.9921225	172.9918445	97.84411714
35.68076887	45.0052814	53.24606121	38.41573899	53.30757575	28.49343664	32.14469538	54.12851478	59.8700648	33.3032147
42.52538517	25.69497535	34.53525775	23.16109826	31.34393293	18.70623927	46.60782429	29.05654325	88.94516416	14.13326717
96.47747829	51.53492118	54.77900967	38.5786788	42.95356325	46.29248521	59.13059717	61.41068305	73.87389284	31.4201946
95.68198122	95.01360411	92.15746318	62.57570946	63.63495235	75.60175896	143.1499031	143.3336376	157.7323984	110.0304505
11.7275034	9.434291965	13.11027884	6.710728317	13.44769349	10.46541998	9.234442893	12.2916445	13.47054521	6.169905079
225.9374514	116.588648	146.3752866	122.3221275	179.5846208	118.455974	211.1885622	218.0741179	211.3608292	100.7349871
20.76930779	13.93247215	15.36574924	15.89547433	15.84667351	12.62260362	20.08491374	14.64830331	16.85915069	9.031964218
21.85422208	24.21607452	27.2754758	5.338496704	18.72454015	13.88390077	5.577580789	14.65322121	48.8939771	9.499956877
235.0375013	199.3834512	192.1071589	186.1919381	269.3216137	215.8945483	336.092044	371.6160075	303.8138451	158.5217986
3.031290279	11.55165751	9.371702252	4.798661757	9.992979902	6.554256037	6.728096156	7.274553845	8.746142506	2.672940419
21.69789928	33.55394072	32.58701431	25.02191955	25.06545316	24.05578399	59.11195589	26.81481755	35.11705383	14.78545706
0.189689999	0.050902684	0.059758964	0.06662796	0.121870786	0.071906032	0.121986471	0.097887896	0.155899972	0.0565209
12.65837572	36.08914217	25.01457533	8.965243188	17.33606487	19.33476144	18.38077742	48.14197652	22.57181188	11.99900534
114.3335689	109.0848446	101.8747809	140.6138994	131.2824011	119.0709422	173.0932729	186.7720507	121.5499979	77.52075266
18.40203968	21.86655344	17.98866445	30.68414482	21.47055435	15.01563426	10.06142861	15.39400983	22.09290392	14.12460475
32.32400532	82.63338756	33.85523898	47.72820865	63.47591762	33.72634168	44.93011094	73.06064372	44.66929796	35.20473759
3.979770823	0.930402488	1.484832579	0.678788336	2.112043265	0.765110767	4.566003615	2.143053857	8.622818694	0.987289555
200.0882954	157.7183178	179.326826	136.4755471	171.1614678	125.1846727	219.5986499	242.5923544	193.6898995	108.0951832
0.310118256	0.212333671	0.452915148	0.166976163	0.387414191	0.171654465	0.598670907	0.329272858	0.479936451	0.285566816
36.19460143	41.28541679	31.43418088	47.21532762	38.52400512	23.82360825	116.3958626	44.00241406	44.51692915	23.34364451
181.2797154	87.39477133	123.2731569	123.0049738	128.3573557	92.49189381	133.6587862	130.1913266	113.5364168	77.95193266
38.57989995	47.8116382	49.83131517	26.97183763	31.36408005	33.29497324	48.15738477	39.18312625	49.00281386	39.89178604
218.5155486	188.276177	188.4317693	164.5463168	145.8970772	129.7439963	264.4034554	167.2103116	242.5029554	134.8788022
8.185435724	10.83810442	11.67975342	3.862023934	8.178744798	5.700293293	5.140063535	7.430728208	8.677237436	6.34811761
159.0261898	147.4963557	129.9599945	145.9786641	172.5954838	133.9807471	213.1492636	212.2878659	181.3894296	102.7132714
12.90942847	8.78898737	9.738780529	6.049817915	11.22395225	4.217195037	8.088151213	14.63396219	19.01476203	6.349494354
692.6506016	195.8290332	519.3302589	211.8814214	481.078538	301.9398765	429.6596837	477.7581684	451.4277708	198.4091358
139.4717728	130.4251144	146.3091848	61.12332134	104.3359592	72.40376082	135.7649404	133.7421416	148.4445386	47.86888501
4.224827452	3.228302971	6.343703691	0.998838635	4.187511508	2.785354464	0.693673129	2.121101101	10.96570122	1.59652379
111.4460746	85.49756445	69.10933551	74.43438601	91.45677008	69.23759204	95.43890567	79.89821974	77.41262959	54.9188178
35.42951461	8.831959259	26.42583969	10.232127	57.6364089	13.1676145	36.01181873	39.85753633	32.56628683	21.00771275
9.061097723	8.125950627	8.545259015	5.492994566	6.560733779	3.939945527	12.05284086	6.399992024	6.382335603	3.768585797
55.39423219	16.27294586	28.14354836	24.82192064	24.22541533	17.45713556	51.5085574	22.74710529	88.15942161	14.81208898
0.009005336	0.00339863	0.007848583	0.01018443	0.005252883	0.008601703	0.038756613	0.007894806	0.037330175	0.0115606
10.18648899	4.584156122	6.156210473	3.807839662	16.67112239	8.584255825	17.93178535	10.84577009	11.10993588	4.346750377
194.5315579	155.0098066	200.8364015	159.6879163	206.1358244	122.9161402	185.9776133	208.2094108	166.6028493	99.77228857
19.51874109	14.18406285	18.31808565	12.55236432	18.87618493	14.36723775	26.5955648	15.75239614	16.41512742	12.03727205
37.43148646	32.63034889	31.61928542	21.89935713	37.10875809	32.12546514	38.32148711	50.9134817	42.86568438	23.30285122
9.765113941	9.588151596	10.21805932	10.25331464	10.25102026	10.37838988	9.301668411	11.29371688	9.603875635	11.04942718
19.39941453	22.03982955	21.43528722	18.60439835	22.20343058	16.01828924	21.18109744	22.5480201	18.3124019	12.52624921
0.00968061	0.007910337	0.009722963	0.003636628	0.011413539	0.008184726	0.004723676	0.007547281	0.010472814	0.006023057
0.810652568	0.211401538	0.701142997	0.092352898	1.848575199	0.424782226	1.032786524	1.023407744	2.188896501	0.332583135
0.073645454	0.078678143	0.084184634	0.056818523	0.063380748	0.03584023	0.049851736	0.061417752	0.082698708	0.051014997
2.357066328	1.117265436	1.304360953	1.152650004	3.167451061	1.433050366	5.679966197	1.585805013	4.157909172	0.688004762
86.3719957	36.30442098	77.15646423	65.04664121	95.46815421	36.06712122	103.6240385	90.23912429	100.8626302	56.42974434
13.23143002	16.15350575	5.681484224	13.47879155	16.98008499	11.1578524	15.39218557	22.33359308	13.92278399	12.16774829
109.7556902	80.65985687	159.0353905	39.54662911	161.4558927	96.75241012	91.35777994	86.1264799	99.11277198	63.13045492
65.55624577	64.8776	62.88457833	59.71439911	65.28726184	41.15644622	49.529773	48.89875204	55.05335822	30.84114104
13.9456329	15.72555436	12.85554981	22.09658211	16.04310496	12.31796155	18.89923787	16.31529036	19.31104597	14.66619605
96.97495746	66.61981668	54.12348975	59.27034954	68.81365567	45.08902782	119.3161826	77.42627016	78.01625386	41.35178188
7.448874674	10.42421769	7.654168429	5.819675996	12.97782424	6.079233513	11.8023486	16.24963925	7.986821305	5.336108212
33.30446563	19.61864389	34.58598097	15.24335653	57.41897381	26.57943138	71.43702297	39.95052168	56.43777683	32.08800296
16.40734795	5.984965302	10.50649281	6.412098007	19.599448	7.591730179	19.56511116	32.64884811	20.25969512	14.65962535
0.945360251	0.673170769	0.28772438	1.25417288	0.585206042	1.381546944	1.733536367	1.363021963	1.372454866	2.326419067
15.87699021	15.69358709	14.45849523	11.4452986	16.07614867	9.508055943	20.60986222	15.14275954	24.52398517	9.089873368
0.192370044	0.187082923	0.183421997	0.083077856	0.225540468	0.099534892	0.082607499	0.196144552	0.162513341	0.068206764
13.14771801	18.38962086	20.84907311	13.21984043	17.52865734	19.19164487	36.91833464	25.2298765	31.91495133	17.95010021
0.046305412	0.031489291	0.040191844	0.01409281	0.050285823	0.042260613	0.094939882	0.137780487	0.088960851	0.025611772
4.569655172	5.704075295	6.644175107	2.410118421	9.305350856	6.427806884	3.83581831	13.28743219	3.271363353	2.678765232
117.3263227	72.4114753	147.9819663	29.05372414	103.7870016	38.1801285	149.6353531	256.6941378	350.995191	48.24513367
4.674151845	4.646147442	4.243160967	3.58848034	4.55298302	3.140598381	5.067356143	6.194616961	5.235528178	3.212001481
2.661703763	2.805273364	3.44675088	1.004060948	3.490870439	1.792762111	0.944606737	2.290517272	5.189273143	0.975051921
0.907764758	0.987450368	0.939324399	0.840577475	0.907029096	0.776081941	0.74532947	0.871654467	0.778215005	0.78276918
2.697220977	6.563398911	4.522659208	2.676529299	6.144267868	3.274527523	3.146862556	5.351851217	5.39089817	2.349623265
0.196152837	0.228978862	0.313651168	0.113186585	0.396208747	0.204566479	0.119489762	0.320917463	0.35204859	0.124414101
40.36175594	40.58610853	53.88942939	22.0188106	60.81051052	28.39463312	70.03883467	79.21932039	65.04234751	24.61256685
15.92852671	32.63480026	31.14502031	20.60787092	26.98275474	18.02079922	12.13385399	31.21900845	23.58234564	11.9383169
22.08220506	18.05145146	15.60002722	24.33400624	21.91280228	15.2490666	14.22922099	21.07965896	21.59129296	16.11204219
6.301532689	3.696034127	1.824547293	3.757153684	4.129399084	2.582230905	12.24751836	5.054998208	8.558590608	4.143399293
24.08898268	21.67204502	17.13591496	21.38757886	19.59669168	15.14963136	16.05098919	22.87430856	19.72392058	13.3318179
53.73027868	47.17721479	58.72677157	40.35401305	48.81725957	37.45624666	77.01783004	70.94119764	42.97497438	27.14883844
176.0660715	118.4414795	155.4167476	68.24408901	122.7592148	71.00468328	132.2236107	72.00662416	185.8809137	72.26562795
125.6350427	143.4574116	133.3738651	83.83248856	254.0813509	43.46752156	97.96809179	108.8959493	100.855654	108.6968893
9.058766524	6.397027191	8.25429719	5.231074095	10.20147845	4.803461415	17.19602639	9.85567325	24.22639114	3.671691248
94.10840207	89.43331338	122.1686317	116.0416561	92.43869949	77.72514706	82.00117541	87.37536544	80.52485227	80.48882522
16.57935025	15.51400525	15.29886379	16.14465883	16.37745596	15.45733773	14.2533649	16.2860627	15.26200553	15.22680835
117.1261373	112.5217507	73.20650597	72.11403166	88.71626365	65.80798219	63.29703328	173.0270419	73.79525625	61.95264522
0.061677535	0.060426777	0.043476928	0.030390248	0.06659357	0.046891473	0.034287504	0.08709378	0.045768389	0.030339058
2.797625589	6.143700562	3.86771423	5.928526305	7.26263788	4.884220901	7.445124976	10.08712188	5.482061738	3.785434942
131.9250211	106.3768832	77.18870356	81.6352094	95.73395366	77.98118578	52.34778175	125.0956438	76.23975627	65.35837259
198.9076043	235.6547795	233.1131413	190.4746697	245.3912768	156.5525555	177.8997855	193.6801118	167.0453708	167.5326811
1.704930841	0.994761075	1.343163652	0.749591987	3.18679969	0.602351939	1.517254419	1.473621172	2.332745329	0.463209514
20.93350844	26.00111729	25.70656028	23.05010928	24.183989	15.51179187	22.83901076	24.00083307	27.43133971	19.06399319
97.46743523	40.97664305	70.65791295	43.11882309	63.85552205	53.38439158	115.6505885	104.5708082	88.35182195	50.56657184
73.03785328	33.25451533	56.37565082	22.47728707	40.57976236	36.35887425	51.69505442	35.95588419	66.43470346	23.55430971
3.639869221	3.306701833	5.32125814	2.054464399	4.370690482	3.082068404	5.332029555	4.309612991	6.768443434	2.706082585
8.682038812	2.245219715	7.079387515	3.605850565	19.14277634	2.80254853	6.190060544	7.804707519	14.72089417	2.585606927
14.3689966	37.56325744	16.74930494	24.55127643	37.17747598	22.81843177	23.27523217	59.56405123	24.7474336	17.52882474
0.009279065	0.008268725	0.006566138	0.004285406	0.009134799	0.007541373	0.014552627	0.018885248	0.015353365	0.006412841
7.184519595	3.165991332	5.329382363	2.716648138	11.43387668	2.644898288	12.74120049	4.240799521	7.350922447	4.551838979
79.44648615	11.68298345	36.12646277	13.46323186	57.3927983	19.25514972	33.2516923	79.76158758	34.44747273	19.90420144
0.023398071	0.012822886	0.013350193	0.006750811	0.035767547	0.020000995	0.051911526	0.02904657	0.030567635	0.00895803
0.124363662	0.037643949	0.06712137	0.043062126	0.152262411	0.049586977	0.152477892	0.161058027	0.184603178	0.06977904

Supplementary Table 6. The IC50 of the corresponding drug for GSE21257 samples.

Drug	GSM530667	GSM530899	GSM531283	GSM531284	GSM531285	GSM531286	GSM531287	GSM531288	GSM531289	GSM531290	GSM531291	GSM531292	GSM531293	GSM531294	GSM531295	GSM531296	GSM531297
JQ1	15.18023474	17.04173079	15.311716	17.07984916	17.37258025	15.30035319	13.97814604	14.39624807	14.48637492	14.65483825	15.78179554	16.59257406	13.59401418	16.75240015	14.52665792	14.61322495	14.54723149
BMS-754807	1.210937933	1.685546907	2.489664955	1.763470296	2.688913638	1.118685654	0.93164219	0.785514457	1.781094485	3.381399849	1.062900022	1.074532825	0.702802037	1.716760649	1.460948137	1.406587974	1.257085427
AT13148	36.1018719	21.93055396	36.74965266	57.0713566	68.35212552	53.6412212	44.55750935	52.84021585	41.08619935	47.84394785	20.5413522	28.41606984	55.1679505	24.70609093	32.43125755	30.27937873	37.35140851
AZD6482	28.33390052	27.64348286	24.58497672	30.74382293	27.93932015	28.00633627	25.88793704	21.06013254	24.29931464	21.03163698	25.24288918	18.36427545	26.07397788	33.43385872	25.14597878	24.22391762	20.15113774
VE821	35.63009564	51.06763867	34.48893242	96.42505344	57.268612	149.7447278	101.6100944	95.00005874	67.66106762	20.94355118	23.20475304	44.48681751	56.57454132	35.44254014	78.5953139	44.38476575	100.7050893
GSK591	104.4037084	92.04275808	89.53293249	101.5424075	125.5944363	139.8166151	155.6486909	97.08758842	121.0518389	80.43803825	55.43128281	75.20374346	104.1735439	56.86041328	94.76540582	98.41329877	142.2160346
AZ6102	10.84052497	8.903121382	8.073297409	12.82697862	8.38544227	17.75866962	9.821502449	11.84032944	14.26302114	13.42964379	5.777673887	11.90293399	10.2113775	7.448022822	10.24512519	8.502156736	9.712821466
LJI308	158.4381119	128.6771999	183.7218324	221.4380565	148.2413864	256.0153828	180.536307	195.2713473	177.1445666	137.1659849	88.73691201	124.6082219	152.4155834	106.9815393	112.3072742	119.6100053	162.0866734
Uprosertib	16.54123887	19.52232994	10.91151289	16.3624594	36.77897062	18.98837762	10.77450273	13.53963618	15.83233227	21.85815258	11.40309236	13.71441604	22.10676876	12.03350347	12.32527356	9.106545248	14.10747146
VX-11e	12.04893117	15.77474064	17.53502418	23.30063338	14.96544288	24.09115779	20.22079853	19.07937075	25.56506893	28.4678757	4.394870422	15.21931527	9.564000414	7.280743537	24.36358288	12.57536282	27.92802767
Vinorelbine	0.023353447	0.018101217	0.026974765	0.048652882	0.074675902	0.088826112	0.063035856	0.054542866	0.080655683	0.038613251	0.010819806	0.052172732	0.056526242	0.023218515	0.029757316	0.035399441	0.05954989
Ulixertinib	7.309334135	6.801580862	9.800655866	9.058552187	10.35065523	9.296402944	11.66686847	12.77196108	8.06818416	9.123919558	4.036039706	9.680596454	7.113945694	3.7112692	12.26489498	7.317136602	11.71143803
MK-8776	14.5562046	23.53362241	20.35248683	33.52781403	35.59127063	48.14098227	21.02115552	17.04443459	43.15575178	11.94410743	10.15305171	19.15972917	19.40665686	18.54198594	33.54259948	12.72734352	24.21598058
AMG-319	116.432841	107.9582575	142.5965648	136.6795794	193.1715179	145.0947637	133.5150087	104.3536605	134.9228127	132.7977922	84.04750511	84.31502469	177.0164439	91.71490119	131.7390865	86.06657322	132.7271259
Pyridostatin	25.36498236	20.88445538	18.14752121	22.43439337	34.5665455	28.63114112	27.04484804	27.83242532	24.58281302	23.53454424	20.52855828	25.53206523	37.63571022	18.43020135	21.41128603	23.24617963	26.4551818
BIBR-1532	123.896698	153.5846108	130.2934309	159.3130958	113.3394308	138.9422664	153.03459	190.0412854	152.195392	82.13320134	77.80125176	118.1589494	156.9338581	74.4586783	101.5695996	119.2134655	174.0769549
Foretinib	1.641658379	2.550649998	2.033383741	2.019373634	2.688209242	2.051523299	1.853717517	1.77292697	3.48183941	3.386285618	1.515213505	3.683589751	1.98835282	1.837193424	1.741851343	2.318935428	2.647920936
BPD-00008900	62.22017478	47.8280767	90.51063328	103.5251899	106.9086218	103.6532057	119.874317	85.50132063	124.8209818	124.479285	63.06605118	77.4333189	114.6264548	48.15842626	105.7686958	55.77996557	99.83917009
WEHI-539	32.35897236	30.2665862	30.18810929	23.15370372	21.918396	39.74956913	31.55360252	41.55591118	57.72326618	18.26293359	18.17538095	28.45799837	41.93184467	19.52414431	20.94781812	30.99266624	45.46908522
MIM1	43.95974587	44.90831728	43.03677053	69.6400896	54.87683123	44.52226675	88.90906703	48.27279547	46.29760702	30.77491938	28.01117932	58.02584199	60.04090906	29.14367448	45.20331203	37.07868023	59.62958807
Sepantronium bromid	0.013951663	0.008770784	0.007955329	0.022466179	0.006899729	0.009303836	0.017591397	0.025852895	0.014340322	0.013578604	0.0121812	0.017135174	0.016020149	0.006497401	0.015114112	0.015797103	0.019752536
WIKI4	37.50829902	38.58019609	35.94350958	40.39522003	37.07686159	41.6720956	44.74337267	38.77106349	41.00876494	33.36180385	36.18842756	42.48826391	44.73927554	32.47430609	35.93145861	36.25849189	42.79746968
UMI-77	14.41420574	9.752679742	9.695078772	14.81102588	10.9384701	11.96062434	18.5787325	16.31315259	10.80244325	7.943624857	10.86606752	17.76364987	12.48136553	10.58970377	11.66546238	12.15182097	15.33018444
Savolitinib	14.31873625	20.61790647	12.92449739	15.56013613	13.43381047	11.67940383	16.54285113	12.81238864	11.90575313	10.30747688	10.82230337	15.62155601	14.14763734	7.970626278	10.06337043	10.57562513	15.86058166
P22077	63.25073172	99.10471363	50.43963974	106.264251	55.96381599	112.6028425	129.7134753	148.1925579	76.07492536	61.64385384	48.56406385	95.75735359	85.9317606	42.33946687	63.56229382	55.21556528	116.3221002
NVP-ADW742	9.717675416	14.2712501	9.049732945	14.22153663	11.19029387	13.6328155	12.70986258	17.88896161	19.94783615	9.540618907	11.44149902	33.64235697	9.897268563	8.790518897	12.18173376	13.22795133	21.22490634
MIRA-1	131.1752922	177.1423377	150.173407	242.6506222	210.0941301	300.205088	223.7938595	260.3575857	250.7547232	164.9464055	145.7835769	231.1953575	234.2540139	137.4175217	234.160497	167.9806701	266.3809972
Telomerase Inhibitor I	1.358818866	1.124999919	1.106650983	1.402395715	1.351184609	1.746223098	1.906155986	1.806563981	1.605911634	1.631722755	0.735010948	1.712966746	1.495950477	1.051939298	1.40425046	1.13836236	1.653511453
I-BRD9	69.68881495	51.06322416	57.71386388	61.41908917	87.49516712	90.79506701	85.411458	88.65358597	91.56160778	43.22969855	49.3258313	86.11930501	86.87398672	36.56986174	62.6099962	51.5663353	103.6553711
GSK2578215A	131.6098927	116.6596036	108.3132695	164.3231679	144.4184824	185.1438293	143.1242459	171.8387322	132.9633709	99.05319472	104.7845421	119.2704031	162.2613343	89.22935813	124.0366837	119.1753611	146.3451939
GNE-317	1.887596092	1.620520667	1.556403842	1.684434604	1.249112919	2.170413759	1.430897875	1.310102952	2.196751874	1.810703597	1.03838078	1.327806643	1.627428033	1.689557549	1.488935243	1.771321118	1.382751769
GDC0810	133.2985273	111.490161	126.1747895	143.3068046	165.7657598	138.8589738	173.9823023	160.2707794	129.8371205	100.8121891	94.76939425	124.438205	172.1173689	73.66119798	115.2523156	110.5783614	136.1996363
Ipatasertib	33.32904609	33.90278955	24.51438078	21.71199787	56.6813057	42.7505222	34.53178451	26.31351033	30.43203151	35.5841771	32.81263906	30.36849875	46.64987054	31.58402251	24.73945953	22.94261675	28.78959829
Cediranib	7.727221541	4.888996865	5.886664794	5.173346425	9.27526421	6.823364118	7.507539309	7.433508591	5.805063365	8.133941489	6.335152665	9.768451543	11.63730691	8.390183049	5.54417383	6.265910516	5.467297211
Osimertinib	4.164383934	4.85035511	3.780031003	3.123699356	6.440655116	8.889307503	6.447753914	7.338030182	5.689466645	3.725135005	4.124365722	6.320289484	5.937205371	4.143315561	3.847005137	3.116624743	4.364619844
AZD8186	27.55481437	35.01898475	24.70592917	28.0212577	31.60607899	27.84147103	23.17305151	13.87050191	26.82518764	30.54987651	31.99669001	22.51397999	29.90751128	35.77539606	26.4057665	21.24855818	19.22745487
AZD6738	4.756350722	9.207785248	4.039657251	9.87228758	5.934477803	15.43669412	9.712580702	6.712802668	13.08735726	2.909830388	3.040216945	7.217790884	5.461581011	5.027020712	8.248729956	5.510521894	7.417545837
AZD5363	20.12918078	13.72058455	17.10335624	16.01268848	37.41767932	22.95430078	17.26637828	14.53514999	24.56880773	27.89983391	16.06767674	16.37745474	22.71590853	17.82053493	15.80580385	12.72617692	14.40432458
AZD3759	14.37801374	10.37131997	10.58240301	12.61356894	17.6059053	21.79243094	21.23673213	18.2183641	13.55457477	12.86841985	11.66811836	12.74805744	18.68851198	12.52088647	11.00981216	9.27092318	13.60032635
AGI-5198	98.54977649	110.7813205	104.1755195	113.4542869	114.44737	137.4775985	125.9937494	125.1793341	106.9208871	99.20493343	82.94712089	95.76073939	120.451555	84.89973779	90.43678648	97.64232217	125.7173465
Afuresertib	14.83453839	13.70980464	11.29706287	11.01429713	24.85289719	10.93560277	11.62956301	7.769722979	12.93793474	13.84309375	11.35660734	14.60045777	18.09801908	10.39779058	8.524314937	8.424762942	11.52819262
Dactinomycin	0.005246607	0.005276055	0.005753312	0.011262359	0.005650944	0.008655235	0.011985311	0.007831365	0.009870554	0.014501489	0.003267889	0.00760384	0.006349718	0.004368442	0.006805461	0.006063828	0.007169769
ABT737	7.631425128	10.71846175	7.074873504	4.641570342	7.665221778	10.82023913	5.253262988	18.1793105	12.48477205	3.235527538	5.127715182	9.224673031	5.340337799	5.101907369	5.990275974	4.98726065	6.711319382
Venetoclax	10.60837103	9.726189188	8.240298294	7.103538154	10.43519352	12.37300794	9.949754375	11.99792196	9.845088431	4.42446477	6.406467554	7.551561341	8.836470412	6.701323856	5.715383542	6.679270153	10.14343419
Ulixertinib	12.6017459	15.68965504	20.1055514	22.6321969	20.13966419	19.15154587	24.5515985	14.3007701	27.84230816	16.94076544	6.519444212	18.71890676	8.759196208	9.437518999	21.68548781	15.3518482	18.20244974
Buparlisib	2.311702519	2.264310304	2.29931998	2.740616137	2.511147874	3.32571505	2.837109576	2.105200088	3.209711373	2.177154648	1.800939248	2.652135503	2.676846073	1.95046451	1.878324002	2.524915164	2.488687719
BDP-00009066	7.468666582	7.741437813	9.544712737	16.9969968	10.35128445	10.3649627	15.90614078	10.27802185	12.05920463	11.60600924	6.157012942	10.64037351	9.244836393	6.757306176	10.6945292	7.219303489	11.03548946
MG-132	0.148526145	0.189273004	0.173505338	0.212584134	0.16307701	0.158934321	0.224629095	0.238885823	0.155744596	0.25401988	0.157987443	0.16465289	0.20768215	0.149550962	0.164242792	0.174804314	0.214762502
KRAS (G12C) Inhibitor	85.54130189	82.68437382	54.93546595	134.8771978	80.09398505	111.1268303	87.641057	103.1489459	83.81015135	25.36851509	26.95223862	51.6102624	83.78325873	35.76075453	63.06151651	82.68061061	126.9829742
MN-64	115.5848237	105.0535745	92.45486207	140.9605387	142.4983295	117.5508295	126.9923401	117.5383682	107.3177975	66.39396708	72.29324075	83.06509093	157.0435437	79.77677666	90.40409133	87.77508658	143.2759657
OF-1	88.61844228	66.39798239	68.47491986	97.09153501	68.50623665	65.04553084	68.85740792	69.08101441	59.30323037	31.78572467	35.10696737	43.87942592	91.65608176	47.43776035	56.54075656	52.56427601	79.45697963
LY2109761	151.1919553	183.7467117	155.5874083	228.6815338	155.7474474	196.6990222	160.2468652	182.2292595	139.145919	83.10752135	153.3793188	207.0616155	179.4668002	143.9693841	109.3223889	131.9912811	194.4181179
Sabutoclax	0.916839668	0.909261716	0.490022506	1.138454987	0.672978457	0.909834244	0.910056848	0.541707888	0.664169702	0.560566017	0.346726365	0.683744118	0.731646814	0.49432319	0.630616266	0.611684293	0.714538743
Sinularin	28.02195917	23.21403278	24.97017854	33.67840517	33.13409815	45.56592707	37.04829009	45.83413328	37.37345444	22.87132574	18.87472735	30.7479697	48.49557783	17.76492678	34.96966757	25.78353145	54.58654826
Elephantin	23.46589828	15.42056214	24.10680478	36.5196654	44.8014307	33.20385372	42.42169483	31.55695212	29.36873839	46.69812856	17.879774	27.89410111	43.18683841	19.64722969	39.09992732	25.13782049	48.90679601
Gallibiscoquinazole	14.07871463	11.68865437	11.24248898	15.59868186	12.64172027	16.49141223	17.9209586	21.53905879	11.95270787	8.516619467	10.73894142	12.54331994	21.1079244	10.52366009	12.09691286	10.96577026	18.83162315
Dihydrorotenone	3.05323196	2.497778979	2.414203512	3.624406579	2.725420465	2.526700144	3.208139858	3.224365051	3.015319712	1.3126613	1.471930965	1.549558747	3.898517452	1.597296804	2.648708008	2.744610347	4.167594104
Podophyllotoxin brom	0.369293097	0.232274215	0.47415178	0.586556675	0.827270755	0.872676782	0.644781569	0.736728105	0.675394629	0.374100971	0.202499425	0.489467389	0.634789182	0.290996874	0.655911363	0.514016969	0.899776046
Docetaxel	0.051540463	0.017654767	0.058479343	0.183561129	0.157024025	0.165943573	0.177499031	0.11854741	0.222112392	0.02724536	0.025441966	0.08535237	0.179192157	0.022359704	0.055572511	0.0990173	0.365829604
Vincristine	0.093559523	0.048201952	0.088348896	0.224532451	0.278795923	0.335781199	0.203313074	0.368046031	0.223610524	0.064359768	0.039100505	0.196372482	0.23287446	0.056640909	0.200137411	0.168284933	0.343882907
Fulvestrant	93.70750223	71.62894974	80.39615599	96.1690655	109.13804	104.4247557	107.6792969	112.4178477	93.11612001	50.1727777	60.89709972	95.79582207	116.3598573	51.40415671	91.31332379	86.00341481	159.3652168
Nelarabine	347.9454474	326.2304463	239.8242322	524.9964238	548.7939886	464.7614585	369.9673032	417.0792323	389.1017085	234.3586936	272.158069	476.4613735	427.7659848	315.9330904	328.5774526	296.7316376	572.506162
Fludarabine	132.5249038	159.2394605	158.00885	175.6177481	205.0542466	265.5109163	150.9729507	167.3577993	247.5296995	81.61631786	64.39133671	149.6670942	157.9564166	105.5367527	137.8282541	138.5363292	209.1758943
Dactinomycin	0.056434278	0.061423625	0.045485936	0.097212355	0.098236845	0.12188313	0.120323521	0.091180346	0.143057539	0.096683584	0.036725439	0.111841122	0.088376455	0.049464912	0.07565946	0.078438381	0.098413186
Mitoxantrone	1.775938873	1.659016745	1.070342744	2.011540692	3.565066952	4.48546788	2.954507636	0.991022144	3.720221191	1.919471472	0.420591089	4.534244613	1.990435008	1.008241178	2.193147868	1.91171348	3.968842809
Teniposide	1.377449158	1.509843399	1.171115713	2.793952054	2.567730993	3.586473806	3.313387652	0.946761224	3.397650422	1.263665359	0.46232342	3.064673025	1.934220696	0.919930188	1.539006458	1.172852445	5.163807147
Topotecan	0.944544619	0.891510684	0.878105237	1.152862657	1.009983732	2.479342759	2.426412753	1.106981983	2.110086942	0.822855623	0.257598261	1.847458483	1.038209197	0.719844418	1.705359388	0.778551287	2.323860302
Carmustine	382.2612876	364.2155584	321.4903921	505.6626919	362.800757	504.3065182	535.099156	666.1412739	427.5901844	280.8763936	298.3043913	430.9605501	450.5479938	272.1574953	391.7994761	380.8487826	555.774827
Oxaliplatin	108.8419353	120.511132	113.9735959	156.7958533	136.6509748	233.1779117	186.5088289	136.2784989	284.4523965	284.5723038	57.8087592	120.6525532	96.47577937	68.06256838	178.5225261	117.9464611	198.4769481
Acetalax	106.0238407	71.53795649	113.5973136	88.77235735	164.8163323	44.87585111	211.5119442	54.03365955	73.19368297	81.42375712	80.18398497	67.0934044	196.810967	147.0024133	105.2295954	214.9508758	168.3248975
Zoledronate	37.50026922	35.53940731	34.0009325	54.83531166	39.73366945	46.34185398	42.82267357	53.31636083	37.01753649	27.32827579	30.05981056	34.60583574	43.32014378	27.76139436	32.51515866	30.83570731	55.83132617
Ibrutinib	90.14958499	53.17487179	75.93339301	86.82211145	147.1871281	110.7580533	96.29890976	134.3111661	66.82567004	51.49507756	64.99076648	71.21052313	94.86979629	59.10484653	71.73056042	54.77488568	113.2957781
AZD4547	15.31829134	14.56027375	13.75002287	19.35569441	23.83097966	11.70794803	22.92665174	18.06996195	13.73779867	15.44898853	13.03393223	21.54074171	22.29719537	17.06417149	11.63802852	17.45285393	18.53737053
JAK_8517	13.65226085	12.00563347	16.19198472	24.02127285	31.5622051	13.75731652	14.28714837	12.81760383	18.72567333	16.22646821	12.26515585	31.79463339	18.54618627	19.3878125	11.38587287	18.65016915	19.31512119
IGF1R_3801	4.440165192	3.560943873	3.47168975	4.889302274	6.249365752	3.045319362	2.435896739	3.054057935	5.75337835	7.161275742	2.604787514	8.811212405	3.574061385	4.138420957	2.040169827	6.631322968	6.851732778
Selumetinib	39.58019332	66.97758999	83.01327152	160.742325	125.61056	104.1057822	88.8613457	59.85520553	161.0561643	121.6798136	36.22722253	71.4899581	22.88351567	50.72921521	160.9261746	48.6954061	107.516509
VSP34_8731	9.696420402	6.961244121	10.94348278	25.36098621	12.45191793	11.4791951	14.06100271	11.28491806	9.917771597	8.793384771	5.395768243	7.70763441	11.63332833	7.241170734	15.81090179	7.374203757	18.00924555
ULK1_4989	7.949399114	4.475112703	5.426844487	16.41023414	19.43427675	7.643565315	14.69371201	12.42871179	5.993269436	5.936026901	6.625386233	12.07155229	15.62092092	9.049226445	10.78337289	8.953678014	12.67719033
TAF1_5496	69.40881298	63.09131898	50.83308864	83.42707307	53.02805307	44.64753566	60.14187468	66.88967652	52.76604346	17.81378591	26.98625961	32.35384932	71.63458502	29.54680075	47.83671907	45.87749296	78.15156312
PAK_5339	9.984519929	8.760880956	9.888380239	13.82786344	11.96588583	11.42167153	11.3651685	12.96276013	11.01164438	8.023440997	8.263600136	10.41519702	10.32156274	7.433759903	9.019385932	10.14989464	14.30205548
AZD5991	92.44138879	53.25490603	53.69272805	158.7123441	100.7868441	68.91778877	130.5795573	129.1119401	31.76144984	5.520448149	49.7657015	39.6379663	102.9863784	18.21675288	45.61947605	37.04246561	259.1756379
JAK1_8709	49.95745269	55.54252564	64.3931896	104.5808406	75.71119031	75.68217722	44.55313452	65.58878037	65.76124522	62.3758355	52.57627162	88.83553809	52.91689015	59.75049866	54.20045999	51.04906696	103.1912951
IRAK4_4710	130.4347154	132.2259067	139.1921723	212.0601924	136.6057895	179.5733729	151.1422594	207.8131573	152.0074786	96.5666005	121.2003455	132.154674	157.5930782	99.39747641	117.9509337	119.0287243	215.6037974
ERK_6604	20.20348784	17.90445881	36.77606349	52.24814957	42.94082796	61.91717633	55.30048842	39.75214435	52.83018547	42.95530395	15.01866399	26.27450945	18.92689965	17.10089754	63.28800581	30.62553182	50.34992179
ERK_2440	7.08818722	8.532784667	14.95070303	22.80789976	25.78441198	19.03662658	18.33682067	14.77463638	22.61686646	34.36715732	6.691973661	16.42361581	7.952858386	8.268275105	26.51188416	11.56948903	19.41039415
Eg5_9814	0.042784899	0.029382208	0.030469126	0.047386891	0.060016221	0.075167965	0.053528405	0.057384853	0.074181654	0.023628842	0.016898882	0.041612862	0.069414357	0.01923935	0.037428125	0.046388485	0.108031448
CDK9_5038	0.064891838	0.051187143	0.078454532	0.205006485	0.121864514	0.12783873	0.107222884	0.121361169	0.067659149	0.118747291	0.053971804	0.086243659	0.113346184	0.060411386	0.105127788	0.061847951	0.14673485
CDK9_5576	0.518114731	0.483319668	0.652738102	1.460383388	0.70025683	0.875815359	1.04080334	0.844171785	0.768206231	0.67191247	0.36030478	0.505735149	0.762200319	0.4130611	0.739174934	0.520901556	1.12639577
AZD5153	5.213152726	3.875011786	5.123805677	7.141825534	7.875281322	5.245394876	4.904605318	5.136394238	6.989288561	6.979160547	3.051568771	5.770229312	5.523967348	1.545790981	8.352805868	6.412915049	8.806308497
Picolinici-acid	140.4765084	147.995866	158.9796469	220.9650828	179.2863376	195.7397616	190.4527554	210.9840386	162.0953857	129.1134981	143.7349239	151.4701615	183.1087717	140.2109128	172.3525462	155.2089337	216.0906851
AGI-6780	73.38678332	63.90739538	72.51582392	74.30064461	73.10358677	62.25303251	77.86633529	83.54694429	48.55175334	38.59095073	43.94716649	50.40740001	71.32988962	40.80880161	63.75339311	55.74373072	91.9862086
Ribociclib	45.14854381	51.33091347	51.78500872	50.63042989	52.72800767	55.64018773	47.9345417	43.16674838	53.12845033	54.32781491	45.96747544	41.22531636	47.19206309	50.30700125	53.22442923	47.10455268	49.22546316
PRT062607	18.57589259	25.35978039	18.98943228	28.91877907	26.21364931	40.84243597	20.95933856	31.10595406	32.40787794	23.75627228	21.30539107	26.20712884	18.5798224	19.20583192	34.3858704	21.13761117	32.54081331
Entospletinib	29.75642068	32.56501268	43.95541121	58.83821985	50.05887742	57.36748839	36.43247091	40.39396112	48.34490341	50.31636726	40.28373464	45.97008551	38.55273348	44.59033116	41.27009457	39.93179219	51.26937345
ML323	99.48726329	90.68139316	92.09516896	108.681906	98.24640166	102.4936379	121.6785746	129.5730126	82.94863325	57.72398964	63.4287975	74.26868489	111.5164213	52.05776672	101.9278147	81.83167029	140.8937265
GSK343	14.75756051	13.91583876	15.70092495	20.66784028	19.30744815	18.31244234	18.26451927	18.98902302	17.04083554	12.74539114	14.60622249	16.75582869	18.75114682	11.98783405	17.02558693	14.62968425	21.36091854
OTX015	9.715769612	7.270993421	10.43249364	14.66300723	20.25679851	11.21044674	12.41245313	9.93814629	11.86517684	10.80075119	10.63492455	15.40964026	9.529393422	4.935807073	14.97293217	12.6492834	15.66716183
RVX-208	108.8809629	73.736607	114.0464942	138.540826	157.9601705	129.7216699	145.9425079	99.35098988	136.1707378	83.38618199	107.122636	120.6061663	114.8713903	55.99828774	128.060677	108.6798119	119.3206181
I-BET-762	25.89968	17.60338199	27.31720578	32.04726975	52.98760022	27.00969776	33.87816328	23.12791203	32.55801781	25.47752221	21.9967616	34.93091429	25.19929843	11.52570226	33.4593526	26.71389493	34.09876968

GSM531298	GSM531299	GSM531300	GSM531301	GSM531302	GSM531303	GSM531304	GSM531305	GSM531306	GSM531307	GSM531308	GSM531309	GSM531310	GSM531311	GSM531312	GSM531313	GSM531314	GSM531319
12.87134707	16.23694202	16.11818316	14.74533007	13.89133788	15.56357885	14.71904792	15.89943993	14.97760998	14.29104545	16.77888512	14.30142428	14.29224911	16.97704287	17.15172067	13.40988836	14.62687313	14.31365922
0.606961708	1.541764537	2.202955108	2.034654969	1.036798157	1.680052435	0.612372782	0.938505955	2.334051534	1.157389845	2.286173053	0.506366133	1.134048446	2.430063647	2.919025276	1.179337049	1.18067707	2.471554077
83.96449494	45.83235428	22.08944272	40.97449289	53.69920986	62.17354612	52.04234496	83.62082615	38.69287111	41.6979758	32.87074567	23.90225197	36.57089153	31.98012193	48.80925961	15.83627984	33.90003174	37.44063018
35.36094351	25.12866859	25.65436052	29.43701	28.83398888	19.53685958	22.08865678	24.43952858	27.87415678	20.45063969	22.53498504	22.26931049	26.74659311	23.91610284	21.7435575	20.77600785	23.44055424	20.98215654
105.6283758	75.04595703	37.87450567	74.32708138	90.20511052	73.65701879	75.5204153	157.5304038	38.72285068	84.16135962	44.96066552	48.1709902	45.6333937	62.516306	33.78343563	39.33540118	75.28266867	64.41630627
231.9806265	92.93142728	68.90720832	109.6221567	153.2549403	111.1963318	112.3828535	163.3872865	85.02633503	96.35172301	85.37108739	86.34605466	91.84466501	101.9440912	78.94341834	63.18413041	91.06849762	71.62960038
6.337990286	17.27367985	13.06574541	16.83847554	14.6203292	9.126726019	18.6611754	12.52817566	13.81151206	13.02223153	11.76663819	11.74223858	11.93865766	12.41467182	17.87417949	8.37614938	10.64402897	11.2543944
238.3513464	208.4527423	129.4920555	194.6811718	166.8241347	160.5886584	204.399638	246.4374501	177.8957718	155.3160818	161.1884147	148.4841198	149.3959607	177.7046084	218.0145868	135.8312525	173.4520155	167.3770026
7.691170198	16.34620595	5.348678747	23.00212775	27.48124263	22.7458704	28.02947707	54.75614033	25.71429209	21.86993368	11.86992646	11.24135283	25.35357257	15.26882136	17.05172043	6.46770681	16.16508812	24.60789846
18.94268249	18.03090701	8.232480878	32.62762294	22.12490983	16.25040799	25.04904604	20.81305952	22.06260128	29.87305129	8.745477211	10.0261627	14.74768308	14.11088444	15.27543654	10.74634604	15.20595398	26.75969399
0.080602904	0.070567273	0.033123747	0.054904802	0.072201712	0.045473798	0.061557756	0.068205407	0.095900844	0.053809035	0.026036032	0.02382975	0.065056042	0.027163654	0.027177111	0.012185244	0.037362529	0.05386545
11.2769441	8.633310236	7.66961203	8.479870839	10.59134154	6.735447977	9.871820025	12.68223816	12.83719456	16.98010774	4.894893129	7.880479024	8.634828902	8.514082708	7.667278236	9.809059148	7.299767728	11.68212295
12.03303195	47.39182118	19.45243941	36.30070805	26.70643754	15.28521396	18.36890284	42.2343971	38.19761693	20.25039548	14.10413323	22.00205746	27.38278505	22.52860876	12.80380512	10.43928026	25.2384619	27.63781573
125.3313916	178.8001003	108.1548469	120.5072786	126.1580342	105.6247981	143.7132416	189.4673485	186.2626823	126.966487	111.0838821	119.6632666	128.7072248	130.6116493	155.5673673	69.66774244	127.663231	135.7202343
19.58635824	27.62274541	25.1577743	25.27861111	42.14070227	49.53307441	33.9172826	58.12433202	36.45633668	32.56694376	28.63881543	29.13476758	39.37940347	31.21031978	30.63598086	21.07113406	27.25334794	26.77352937
73.15545413	182.279488	134.2822595	171.5375402	113.258849	216.0513198	217.974552	266.4465174	153.5339164	130.4340407	131.2787345	172.8233585	168.7558795	153.1690549	148.1947693	108.785871	165.4176043	146.7385071
2.614059578	1.992714381	2.22340872	3.436491455	3.045377059	3.845733985	3.053579414	3.18291708	3.932074549	2.213267433	2.295722742	2.220216885	3.197481018	2.628684616	4.149769507	2.957700053	1.677338345	4.30698784
109.0046816	107.8207389	88.29566095	98.97095048	136.8781108	110.0035438	136.7898132	146.6589553	100.4115669	88.76587541	63.69892763	98.68328183	89.27852764	64.8891665	116.2532149	49.97698758	80.01841732	93.86834429
24.44570127	44.50885432	43.79123257	41.32124345	26.64772977	36.37448896	33.5640791	32.97942473	34.69545136	35.40554116	40.00032207	39.64414622	29.78020671	41.29802637	35.59833017	30.35717529	48.71407151	30.13264961
50.63200017	55.74349547	44.88821185	55.03786531	67.70880124	87.32464983	63.25941087	91.25416741	51.23254386	44.98099708	42.12832161	52.37468871	48.37166603	60.54771951	33.92545123	28.18141333	56.64736337	38.9201598
0.0251415	0.021701663	0.017477295	0.013069642	0.039208615	0.011717529	0.025527978	0.025546233	0.006457075	0.029303079	0.005381935	0.027578297	0.007535062	0.010944414	0.014079847	0.004820816	0.018620254	0.012428459
39.908879	45.29962747	41.5611236	38.34305245	39.29313599	41.15718424	49.66356119	45.1604891	42.8389293	40.1511764	42.23129937	42.92939929	37.96844049	40.48809035	45.00976963	38.03669546	46.95006878	43.59524938
25.29556474	18.74600762	14.71018635	12.61201284	19.84528979	22.26245428	24.04175266	28.306843	14.94503349	17.65353077	15.16169468	18.31953067	13.70087758	17.46968766	15.02054877	11.98617113	19.33393097	10.94692167
11.83491926	13.58363577	12.61635596	18.7933682	16.73831217	15.61315333	17.05710587	33.0626209	16.85685517	10.00245959	14.56921692	16.29914502	17.73348308	12.48719355	13.82518933	11.32732464	10.91296646	11.18079193
83.98117192	129.1377312	65.06116283	82.55358977	92.61367328	144.2238828	118.3486418	232.9101338	91.75688657	117.9801993	60.3152384	97.43794161	120.9219886	84.84942025	64.84498798	43.94693234	89.3724714	78.78453017
8.878697282	23.17356305	13.96812553	15.70213818	21.02831133	35.24994314	17.23077663	31.61506269	12.65147955	25.743166	15.65800176	11.88125068	15.67984873	13.28201841	17.4519104	13.97437759	14.33660213	20.61404931
149.9121957	365.8229407	259.6454441	191.3258924	257.912554	289.4799793	301.26713	488.7845494	228.5300943	271.8467608	181.6496329	265.4217758	191.1419254	197.3875081	248.8242303	110.5852993	232.5257601	228.1912627
1.695233222	1.858841071	1.260438465	1.659167018	2.759843379	2.431948792	2.629134007	3.359672283	1.618519664	2.148226158	1.090026139	1.722181747	1.477055142	1.402837094	1.677199368	0.94628497	1.427631085	1.49511206
99.47958813	110.8137914	83.24478997	85.57849121	125.4120177	117.1569649	106.260105	175.0492679	66.30052633	100.5741313	56.35851858	84.61765683	76.39127966	69.54032932	71.86499153	66.2397463	73.93251867	65.11622909
146.9570685	169.8543018	115.9390244	120.9065571	154.4912879	154.8764503	146.2519522	218.0059945	113.0222565	196.2221641	109.3588177	137.1630961	122.2521587	132.1110637	95.18983575	93.13272775	174.9517432	130.9239952
1.556695185	1.795645171	1.306292881	1.961625551	1.90306116	1.598138063	2.60321164	1.43935491	2.685258627	1.967579809	1.303975132	1.139252826	1.770640921	1.74983413	1.241831512	1.513037453	1.629845955	2.342357519
178.6750877	203.5356039	119.8253466	121.7509846	164.4593559	191.6184724	173.1490081	256.1276424	135.1499567	159.1343453	96.02787862	157.3948407	126.8365337	124.5213879	108.1073743	90.95992908	162.0730274	102.8695847
14.86326629	38.31895045	17.74554717	32.49487707	60.97907002	38.67897676	58.90653988	70.45806071	47.78977134	40.77548011	20.74470937	22.97977474	48.14787684	30.80648002	29.21918411	21.12809574	38.34870524	31.55474664
9.088000634	6.792129926	8.715430669	8.981913216	11.54735166	13.06032341	11.63824145	12.48363398	13.06716677	7.110878768	9.095423214	8.604938341	11.66319616	9.863016131	11.14807678	8.722318607	6.28783192	7.961477473
6.579772737	5.503982429	7.080109131	4.699582157	5.282281612	8.848303932	7.083632648	6.999107011	6.142294991	4.868100992	6.694326528	5.770622742	7.273846266	7.874542229	5.843756931	5.866691998	8.374422607	4.914659681
15.14612554	33.63345388	17.90010317	34.61085735	36.16576634	16.38882466	41.99702806	27.99418297	38.0844769	24.6666714	24.05534947	15.9363297	29.55959727	27.81337622	25.70947297	19.94327172	25.62639055	28.25510754
3.925359828	10.28457578	6.465694798	11.28067001	9.716582972	10.8352142	15.95926151	14.15095256	7.502087733	6.772663997	5.799952264	6.773032628	7.303835203	7.936515766	4.57324011	4.783530318	6.265328736	8.97953641
8.310028466	23.97679062	10.51113234	25.01119752	28.40822625	16.92867984	32.8770808	26.61241484	34.04001809	19.00802511	14.20115502	13.23101025	27.29734911	13.96564972	22.24065523	8.656652343	16.71840115	23.05076149
23.10935016	14.09530312	15.17895065	12.09004913	15.69011713	14.45448409	15.28113176	19.91589688	12.6560093	12.17114898	15.87433886	14.74746186	15.7729402	17.22886211	16.18922899	11.43121683	16.72946611	12.89989332
116.0493793	109.1235646	83.05063253	104.9544439	102.2887852	101.9933608	119.3539361	142.4111331	98.61104946	104.5468181	92.6532238	99.58747928	98.71984554	91.10960837	89.76338112	91.21442173	118.8016075	92.45958374
5.067137796	14.64662816	7.192349703	14.15630164	17.6646072	15.91595704	23.06224746	24.48183901	21.01696686	11.42571776	10.8384427	8.671129256	17.38435984	11.19086958	13.09445542	7.347406393	10.40139904	11.62303709
0.00574359	0.009515797	0.007634504	0.00875865	0.007473201	0.009894795	0.018574107	0.011764169	0.008283031	0.008183841	0.00435344	0.0068084	0.00847206	0.006527122	0.014838146	0.00419089	0.0057493	0.009176091
5.966385054	17.38021731	8.798996095	13.5588818	3.036841887	14.78087436	12.50261488	14.04305971	6.692343631	8.966699172	15.09077471	12.48769957	10.55401653	15.92422918	11.27526444	10.54695445	18.43634813	7.357035034
12.5938808	14.41599492	10.13233365	10.42479152	8.0978364	12.58325627	10.7891169	17.72457354	8.794091053	6.229424788	11.1961727	8.296777223	8.73717828	11.70223567	7.383146734	6.578417047	10.25721545	6.193069462
10.14442618	11.64112438	11.80534192	18.67355906	9.858965015	14.14828561	21.60857846	13.23661284	21.46939806	18.94905052	11.4393608	9.938140904	15.959975	12.40318888	11.73980878	18.56377912	17.94675498	22.28288328
1.980721342	2.640110866	2.375672366	2.404839284	3.074712211	3.025131792	3.836800487	2.941279571	3.939551426	2.69621455	2.283603306	1.902390195	3.242815929	2.466858898	2.11777735	2.082983488	2.178671673	3.082019059
11.88344057	12.83629098	9.244701482	13.01611959	12.47520515	12.25664276	17.80721399	15.58051435	10.54197563	11.6814192	8.278814341	8.698612556	12.00757731	9.618206059	9.696943051	7.109278653	10.74996401	10.89551949
0.265146231	0.176460282	0.189935715	0.205702301	0.209114966	0.185041574	0.283750825	0.208778392	0.180975679	0.222472378	0.229425249	0.156916456	0.221386876	0.199636895	0.23568205	0.136620675	0.154813182	0.224393223
91.47332321	110.6444581	62.93580674	71.23654907	116.9916849	83.98149126	149.4162583	221.0842759	46.42987458	108.8944458	67.29461628	91.40617145	75.62350166	114.9145304	61.14267085	59.10443983	109.4388561	67.01230297
104.8369209	126.7886574	100.1343793	121.3508524	147.1553904	108.3323577	151.3321407	174.0937101	115.7107484	101.057234	141.3918576	124.9015979	98.99689011	134.4720856	137.341026	84.22697772	115.22728	98.6373249
48.24996336	89.53515403	59.97171062	74.32204414	64.36576566	77.00375433	96.60809069	153.0962304	61.51002258	46.29097082	47.88206054	62.50477544	52.44631438	62.84801563	37.62181778	45.8718405	64.87668407	48.06453203
130.1068816	241.6810901	178.520906	149.2702165	209.2483137	196.2247676	292.5482361	351.2664869	205.0888346	154.5226624	170.18505	189.8278775	131.8627021	207.8661968	229.4427655	127.5458633	172.823061	136.5226249
0.336557227	0.683626372	0.487974545	0.629588517	0.799430691	1.084854716	0.885085123	1.434364962	0.864010674	0.616744972	0.514199934	0.602002814	0.724750961	1.169285778	0.473201826	0.405774545	0.530094095	0.694989744
31.64766769	46.40982145	43.18316008	25.7913874	51.11458137	44.7903043	52.98543796	70.39763629	33.14726495	46.28751423	31.52860641	37.2829366	31.98996687	39.00318838	37.89793641	25.47230903	30.67919319	27.79320238
16.61070556	35.06825496	34.34728203	30.32907019	39.87051815	31.68023218	42.12212086	47.45958398	36.89037579	47.34621321	18.33432219	22.0371763	24.64170373	29.68903987	36.56478748	22.77842586	25.78323939	43.66917429
17.54645435	13.38640281	13.74330579	11.09846852	18.70978058	15.1322149	16.87876097	25.89623338	14.62066924	14.83058685	10.82408986	12.33475351	12.90216997	13.59117286	9.965910257	7.013271281	10.5323872	10.37478162
2.259427741	2.809417805	2.110309426	2.282643157	2.422867482	3.239658126	3.068340854	5.098898017	1.916127997	2.978645224	1.532474083	2.602138616	1.959450778	2.309555942	1.141818089	1.566430972	2.329958186	2.198136729
0.925009203	0.73853544	0.520010259	0.392555074	0.68694828	0.412732187	0.549993072	0.555475436	0.589941353	0.704523084	0.334194006	0.309048917	0.538804868	0.380991429	0.466758552	0.16308584	0.472102764	0.660684487
0.211236528	0.123423167	0.119105762	0.043218132	0.260551609	0.091510426	0.188034918	0.126971432	0.117345889	0.141039138	0.079102179	0.053396013	0.097678099	0.073960202	0.083989137	0.0172183	0.070179213	0.151635775
0.184074827	0.432908695	0.257828256	0.141029078	0.263033738	0.122993871	0.228837671	0.238432636	0.204801904	0.234242502	0.085960499	0.080333558	0.165842457	0.098632649	0.166935654	0.022911036	0.125606919	0.263963975
78.83651394	113.0792626	96.25668786	86.10764036	133.4587578	110.8396716	102.7721477	181.2972132	92.42741087	117.7705602	79.68773866	95.6818054	86.54292672	95.88577203	74.65711498	60.36104174	84.09556478	86.34943387
375.1261484	516.6144206	406.915728	316.0332624	447.8631768	404.1918248	513.8825504	731.6770723	553.5937504	448.9916774	498.4269914	362.6937855	418.4000711	493.3332062	555.0399956	270.2969503	363.8129658	315.5025971
215.7181205	195.4332802	143.0505256	204.9333117	104.0148869	167.8590232	132.7193501	298.7954978	238.6897455	121.7799801	174.6572466	169.7649879	186.7959277	128.9508854	136.2231739	104.4139011	175.1689507	153.8739877
0.049095682	0.108069943	0.06661687	0.082512766	0.129484996	0.065158924	0.122312123	0.12934586	0.100925971	0.094513156	0.054015398	0.084198593	0.086783821	0.071766084	0.089869521	0.034109966	0.058389742	0.098261213
0.680454244	5.000984477	2.147932884	1.80671514	1.864197453	1.29654016	2.653322848	1.92326045	3.098806855	0.797126087	1.958408788	1.432733865	1.734898628	2.436904648	2.40942691	0.482064429	1.574922243	2.329424682
1.358729924	3.945372792	2.069033901	1.135716765	1.276324829	1.341068399	2.554704367	1.609276943	2.579836506	0.946585822	1.963063672	1.324136507	1.320466085	1.944606443	2.022344258	0.58410261	1.465675029	1.975639341
0.997544688	3.279789876	1.933960806	0.946971351	1.587041957	1.172346763	2.378718824	1.192600791	1.09896506	0.867994787	0.63541757	1.085513173	0.910786435	1.382495646	1.415647476	0.375022208	1.011180173	1.088020304
433.2873862	534.6518761	444.6208106	351.1264954	634.8914924	528.5992	542.8707599	877.2591717	470.9165581	596.1411519	398.7501808	413.5943047	405.6360412	490.5400491	527.660325	342.1572651	379.7403	346.2407502
31.06892677	210.5067242	146.8710349	161.5589735	191.1501734	131.4901405	224.9253953	188.612737	188.361083	151.8009651	114.7959729	104.5247874	170.3292029	181.1720315	226.2370459	73.92582547	128.8652475	153.2681887
104.9549489	49.35647378	46.72900527	112.8606824	222.5617225	383.6404558	179.2193697	238.3270906	416.6073095	223.4320595	165.6978324	92.68955517	173.5609691	212.0104347	56.77479443	146.7019534	112.001141	170.9046727
34.71198072	65.01703187	49.01928627	41.84160117	61.92180884	54.37327107	67.84030484	91.61770933	45.91806693	54.43347709	48.6264264	37.97623211	40.15520278	56.16081882	50.56615807	30.27825646	46.53895877	36.33388762
110.8399124	104.6508391	94.88117309	82.97753796	148.6257611	141.9087538	98.46910697	158.8588817	84.29965078	98.46994247	74.00311931	97.1870831	79.32603473	158.0332702	141.9044966	51.82793388	69.7216353	81.06118687
14.90747126	21.49784821	23.90233086	19.62005933	16.2353577	38.96290598	24.7647319	43.26442872	19.80243391	14.07977864	17.20016079	18.69634607	23.90179141	23.10719789	17.50289126	11.50164973	12.62054411	14.67397907
10.68410875	21.81031266	28.38713781	16.15900454	13.49690998	33.42550916	22.97246279	23.35749565	35.38032819	14.00529575	30.85464434	19.32774044	24.79027703	34.01255141	28.09766141	10.72030061	22.20860118	20.86667978
2.22103663	3.930930881	6.204531822	4.233677146	5.514652975	10.56901538	5.5334506	5.389648192	11.55193949	3.930163368	13.1990904	4.681107606	5.18835287	10.46387876	11.77506261	3.025888529	3.991598669	6.938534876
61.14802	69.10102714	41.23927375	109.4313841	43.52005524	26.03673185	59.87584593	47.62245155	74.36044279	84.47821993	55.21069515	19.26016725	48.25846878	81.01646147	79.70444548	57.96204908	52.31910003	93.10079307
11.00478947	13.54139669	7.057560844	14.1365977	30.24732217	8.674416361	19.34140981	22.38711243	7.074640754	16.28261814	6.622722335	11.01213316	8.865784084	12.17261709	10.72398496	7.420597399	12.75846722	7.538539808
8.255437824	19.25063486	7.690244042	13.33346679	34.14127202	12.97656781	12.87879593	27.42140037	9.694837044	11.06227149	7.271998957	14.07092266	8.302749178	8.62045765	9.148546298	3.028419341	9.576605555	8.01242523
30.03811588	86.94021623	43.87347717	55.08286935	36.63133174	62.69730448	63.0468104	134.4908875	30.61190453	58.33854962	28.00366744	44.93641879	30.51538713	44.22073326	25.15175298	26.70340966	52.98481731	45.59781677
10.23225649	14.05546643	9.862547121	12.79382785	13.61620091	12.44701116	13.8256668	16.42848746	10.30298158	12.44864647	10.09341589	10.28934322	9.885433936	12.7676812	14.11548536	7.237692592	12.5710855	10.62573012
103.4185177	133.1652323	64.19501599	51.8131197	144.4795274	143.1143366	162.2585814	665.9182132	28.95186822	197.0930888	68.93156857	63.19845943	61.63971208	102.8704511	45.51028846	41.03094261	89.75634655	49.92626417
97.40516301	102.8261226	55.21552928	45.97055027	52.41909573	60.56811392	74.66394799	105.1137635	74.28296386	76.41835429	64.72296311	62.71644329	46.58593504	71.11472669	61.58141271	36.54408844	88.31030167	52.94152904
163.8154183	182.1098909	124.6619499	131.3112372	160.0240171	141.7368975	192.4090365	218.3943265	133.2356275	162.5636903	122.1106338	126.2191964	110.3714935	140.1322347	135.8544243	76.9473111	150.1180438	135.9971294
32.85064013	39.77912032	22.11644963	43.07993402	30.2702216	16.85484809	27.49016771	29.95549045	29.4368323	44.23574983	18.6632641	18.80960566	17.31637906	27.11982324	31.27012357	17.02896294	35.2679908	42.23996822
8.538283907	14.22854863	9.081890581	16.5145907	13.88558755	9.477033031	14.38452981	13.2626176	17.83612034	18.81044864	11.06322757	11.01338094	10.77040093	13.0029632	19.08882065	9.111983965	11.44243539	18.63407967
0.035702076	0.060292833	0.047753081	0.026741087	0.075379456	0.052010498	0.057861736	0.070405445	0.05199649	0.062580432	0.029349443	0.040966618	0.061584846	0.046515702	0.033605188	0.015721776	0.045147801	0.058428258
0.058770586	0.124824021	0.082943791	0.080583025	0.155478823	0.074887071	0.149879608	0.271576036	0.110702731	0.208526768	0.067248971	0.055767126	0.077817359	0.130462746	0.106950454	0.047268494	0.093438699	0.107052135
0.575950192	0.873611833	0.530524315	0.70313166	0.829002443	0.536570177	1.295140535	1.239162244	0.584129141	1.149116327	0.435155497	0.391438945	0.517726392	0.775254311	0.588571448	0.364566825	0.758958834	0.797515262
4.895576661	7.74431952	4.21876289	5.751821673	10.20325571	6.3614027	6.353211967	5.594371012	3.593711858	8.697223929	4.276366484	2.772655405	2.642892724	6.368272035	6.499765163	2.880065727	4.229991219	5.491110608
198.2742252	214.49136	174.8116708	184.1376796	167.6015295	151.4412301	196.296015	234.8865279	145.8236086	177.4030356	159.1355439	128.288167	145.9248309	190.9565887	166.9794983	117.4979831	178.8840127	158.6541386
66.77991576	71.05551949	62.34562433	59.57206859	70.83117666	81.69395527	69.16856315	141.1075263	56.28736872	60.13559392	60.55666131	48.49296384	58.12030271	67.53235863	53.32454872	44.86263128	61.61584433	61.37478174
54.73504874	49.75517147	46.01097924	50.28336579	42.01421799	35.67886278	39.9094127	39.46473823	45.07398408	47.82784206	38.79660567	36.72297663	42.32459823	43.2210453	40.95841685	38.77262321	46.40326488	48.51906573
25.78241117	47.0951464	27.53546808	25.85683415	32.69782663	20.79245408	23.29694325	26.50393087	24.59998521	30.16153452	24.0340195	20.37142454	16.80159235	29.1974625	32.83809804	16.60424286	31.74286074	24.15444443
91.04567808	43.64887564	41.29625945	35.83362527	39.27140748	32.22079124	33.78813653	42.97459553	46.31661769	41.30291746	37.01875916	27.97227811	29.72778837	41.10336535	45.24684646	24.99627342	44.95117014	46.92980058
96.45705388	94.90619279	74.24133571	84.18577045	80.14066761	92.71828538	97.14244832	148.484626	90.15563463	118.3530454	56.36629151	73.05905853	88.80607482	79.61488665	49.26114963	58.90547552	94.17224755	88.80374787
18.46324917	20.88633618	15.72151164	17.67751185	17.27441593	14.50170065	17.29676584	22.42028727	18.02314389	16.23726334	16.81728387	13.03515303	15.68167468	15.68717124	17.66878531	11.89554618	16.49944189	14.98084396
13.52186902	13.35777326	8.683087069	9.868897204	20.35613276	17.4169306	12.14367838	14.43426551	8.074482071	14.62180927	12.16419225	7.254807029	7.283266439	11.92521981	20.50154988	7.907110081	9.481804012	11.89585407
166.3301908	142.9221372	127.6341915	107.7877632	138.3081997	95.03398701	98.41434001	167.3937988	140.5756768	113.3700348	102.0417664	85.18194872	109.698171	111.208812	138.605201	79.95170299	125.576655	101.9503387
47.84739981	29.60077662	24.71697853	27.56682948	36.57224337	34.93294858	22.73438259	37.3827712	24.41772041	27.2813399	26.64547312	17.01857995	24.19852569	26.36687981	37.52795569	20.22904352	22.76713895	25.5894414

GSM531320	GSM531321	GSM531322	GSM531323	GSM531324	GSM531325	GSM531326	GSM531327	GSM531328	GSM531329	GSM531330	GSM531331	GSM531332	GSM531333	GSM531334	GSM531335	GSM531351	GSM531352
14.34136207	14.48619821	14.03236139	14.85959961	14.09617982	15.8349249	14.60716202	14.57294625	14.87748246	14.3754593	15.44352301	14.68659724	16.02764382	16.14247591	14.71577671	13.40654369	15.21307136	15.31834547
0.743540356	1.120188347	2.282871562	0.760887139	0.777808981	1.834954989	0.937299595	2.123012917	1.97583233	1.20896916	1.342653145	0.945066659	0.628906205	1.214451421	1.901138469	0.767575267	1.865381857	2.670383524
20.5590571	27.88318368	37.73740456	43.93348988	39.90567486	45.57051737	65.08927331	26.01333819	32.65287878	34.76947415	24.23519472	31.58952979	67.19261426	42.12564804	31.86489343	33.49217691	18.74257188	75.85691099
20.59703944	18.1973635	25.39716089	22.44519962	25.52585201	26.36979175	21.47087814	26.68264059	19.68775407	28.94283301	21.63975731	35.75489383	28.60911187	26.56951112	26.6198404	22.13678644	20.08071332	28.51866045
46.11220218	55.25759002	58.09113829	88.58946313	36.7500546	60.48398971	38.64535493	27.46572043	86.8513171	51.46363707	55.6444401	89.48300252	105.3450868	65.88720751	70.54442978	81.97876892	42.76136678	90.62348603
70.88223799	90.55323122	78.98882109	80.658441	88.14827267	90.11994966	100.8986021	74.78514953	103.4828288	93.10365218	88.74218531	109.7331452	135.7247677	102.9186989	100.926412	90.97777089	63.36720268	115.8225247
11.17431183	10.24742582	13.19176132	18.36175378	8.51791442	14.939787	10.4952896	8.254937947	13.15973676	10.43247936	11.15640414	13.16814827	15.62803036	14.38777846	11.58288758	9.595927367	8.684453813	14.8372447
124.915628	142.2090264	156.8615618	174.5006535	149.8297482	159.6583023	132.4965656	106.1664082	199.1537794	138.4354179	148.1899558	184.5084502	251.0643852	178.7733438	154.4614279	136.570184	114.542223	180.2135408
8.301095632	22.36387611	32.11943354	19.84426343	10.90169208	12.36691438	16.90954547	16.19893276	40.67332733	7.145784001	13.4034207	11.17329609	16.70056257	24.51543268	13.27221797	19.94541199	8.81780484	51.7465273
8.56742361	13.99932887	24.93771008	20.73145348	11.57529665	17.75044221	17.18025604	15.71088522	37.48337522	24.73406934	13.1267003	12.14786406	16.26519881	25.49249116	21.63659388	16.90868819	12.35110548	28.50617242
0.035824527	0.039063127	0.055338387	0.048553949	0.030829922	0.04472079	0.080340537	0.026130909	0.038991701	0.056479221	0.027053488	0.067100355	0.08154575	0.025250272	0.053506705	0.028111453	0.024925158	0.150069906
4.946206182	9.735311735	10.91830504	10.51464846	9.686702397	7.647952864	8.978098076	9.700497064	18.08672356	5.953743233	7.238670385	5.423664425	17.00665832	11.55350487	12.66626882	13.29031947	8.800655993	13.51314532
20.61151803	16.38418518	41.21481808	30.1107855	17.30962241	21.60998599	20.57010171	14.3207235	33.41732223	19.97534255	27.39105827	39.03679897	30.85891033	20.31315675	31.21688907	21.38447931	26.12653052	92.29352785
99.96318795	94.65432642	116.2978434	139.0188228	124.4929398	156.0176349	149.4606871	145.2183755	143.9911478	94.92548492	119.6453093	153.1607033	174.2288415	121.2555797	135.9355688	87.66794218	113.1103024	234.1270994
26.14117335	34.02591385	31.86840206	36.88267182	28.77552858	23.89162198	25.3584623	20.22714688	31.12493386	15.33177513	31.02238422	30.04264192	47.99403973	31.48786091	29.89692736	24.43545153	23.23147276	61.59423272
123.550567	149.9051558	107.4381972	183.5852907	135.6656552	157.8861506	156.5907745	83.02468223	184.2974228	92.12693954	195.3512822	140.7743259	303.1124855	155.8977733	130.9643053	109.9802028	112.1397783	216.6188315
2.098796299	2.730717011	4.544528151	2.753162023	1.618421562	2.335472732	3.163700254	2.25374356	4.206720172	2.848148626	3.378872423	1.955865848	3.636928569	3.955635896	3.085079859	2.92314033	1.85473656	5.525797057
81.38305422	77.72139156	89.12563128	110.6106125	98.01278156	100.849493	119.0042426	84.68609096	101.8285952	99.15001356	74.14814516	106.969287	144.4761606	80.58056269	87.18483423	84.93525501	56.43835723	151.0161871
47.75992172	43.54250621	23.63674848	54.66098789	36.45105366	29.49347197	28.87865594	17.81474896	41.02049677	20.27127646	46.56539899	40.24554794	57.90714119	55.32389052	19.27974345	29.71813515	38.93764485	44.47402619
41.52875265	44.49147604	36.4385015	55.45781399	54.81339053	49.70992384	55.71520743	31.81703485	51.79550397	27.4447418	53.04729425	49.52298861	87.40801434	44.12935937	45.7331509	42.48497567	36.6428402	63.93968584
0.012149772	0.020532347	0.01369071	0.017456285	0.024718951	0.01868475	0.015498162	0.007152303	0.009895621	0.007947075	0.014239493	0.010823369	0.01590401	0.013116396	0.009687001	0.015663831	0.00925177	0.010460312
46.55734913	38.8682676	39.16465676	43.94437969	43.84775376	44.9104073	46.88990706	30.16196372	43.64550511	37.23035062	45.69288604	40.68062573	49.09905881	39.23720734	41.67606427	38.93930949	32.98856719	43.51020874
11.5899631	24.73947016	9.710531764	21.86973261	14.41691663	16.00475069	14.84282149	9.112498529	16.62914029	8.076624151	18.0696018	11.81946073	40.07374731	22.80210689	14.14612146	14.26667792	14.74900599	13.81946435
13.05669492	13.78465099	13.3285058	14.4178337	13.5514083	15.95260401	13.7464975	7.729799783	13.45214506	10.03670582	12.42119051	16.04303637	21.32433338	16.3043	10.85996309	11.69061127	8.512605329	16.71188786
82.34357153	102.1078011	73.50794651	129.1404787	98.35763767	92.09679336	133.5523512	36.00584001	190.6760914	42.21162418	86.01243147	62.80269083	213.5111959	98.34343821	60.03252132	79.6487284	68.32887042	137.6469024
15.19901378	24.95080205	13.91365245	17.36867006	9.698539569	18.59202776	12.75231206	7.848515566	33.75406395	14.32313703	19.15388874	11.11207503	29.19060901	27.74442346	12.15507037	19.28184491	12.57691788	17.22571438
198.0297597	255.5330593	217.4023882	278.3572423	181.4457594	204.0588379	205.8691436	148.7555951	330.6514748	215.8712626	262.0660487	308.3683556	458.0999198	263.6554695	218.2737881	248.9292023	173.7828173	304.7732368
1.185620284	1.941018826	1.477209759	2.104186825	1.485182481	1.230308966	2.01790599	1.38968714	1.928174658	1.331783019	1.664229547	1.656734472	3.210845286	2.191641061	1.479025247	2.017192119	1.090744551	2.309859433
64.38643097	89.84111155	60.19077306	98.34872246	74.92315411	72.3957604	67.76688763	44.20091308	117.9871428	62.31321319	69.18684825	70.1300463	172.3889815	86.75281034	87.09879579	114.541276	53.34705535	99.37958448
109.28103	144.6088307	110.571356	156.0235291	131.5995451	133.5810929	141.1094021	85.03593545	201.9397036	121.0535213	122.7083741	138.2120651	188.7513612	115.5239119	114.2205854	152.3934916	115.1117683	191.2023048
1.558511687	1.417716632	2.220109352	2.166756257	1.072565638	1.413552738	1.434144079	1.589344123	3.679583034	1.77222602	1.611219765	1.156683933	1.801627954	1.869516672	1.635711316	2.405971415	2.126321342	2.713946164
116.6235672	154.2086173	119.3256911	175.1842319	138.1132278	152.609479	175.9424035	95.27035053	150.3334454	117.182423	149.9428633	124.3341587	213.1143691	110.724518	127.8962944	135.5636359	106.6907295	189.5434991
25.93418745	39.08428999	35.03423989	35.68393607	27.70611395	22.35318852	40.81676474	38.86293755	87.51387655	23.15327077	29.31388211	31.22825851	50.50894202	36.58103058	26.60654018	43.31945887	22.2889843	70.97424858
7.587899428	8.051225099	10.74142198	10.50116987	9.506699073	6.915464831	7.500520617	9.745542764	9.215850494	6.576368886	10.93430678	7.943625334	15.6703214	10.78701066	7.489945906	10.90158731	6.567149942	16.24860858
5.981835802	4.156365085	4.123559265	6.919969644	5.792170623	6.558872723	6.887491545	3.211296939	7.523684719	3.510978244	6.655995613	6.323926477	11.54516079	4.905653523	5.033411179	8.61886501	3.301385553	7.946989565
20.592948	18.16183125	23.91065474	25.25919204	20.71937748	29.50970879	39.36898912	34.2550155	42.17453396	29.58733389	25.94033739	28.2051321	25.42284164	30.4672015	26.03967118	32.54538228	20.59557669	41.98230914
7.651900512	6.519311727	11.07189175	9.421121737	3.96008775	5.638302842	6.019303933	4.128781553	12.11608952	8.483112131	11.10811374	11.40206576	13.76931515	9.711310521	9.169929647	9.35339451	6.172442777	23.06482909
10.82005018	18.1714028	21.25305896	21.58755902	13.41756198	13.41783542	27.57812431	25.99459485	38.91739542	16.86256381	17.4442093	18.64974312	24.06962709	19.21695619	15.3703677	18.41217085	11.70558955	45.01557488
11.69002487	11.28662352	12.06423918	15.35377023	17.47067276	21.49247003	13.20029656	13.39044762	10.83947643	10.30055876	11.17765617	17.3941618	26.77004487	15.00359074	17.6244534	15.05751348	9.110217335	17.35217109
99.28852592	89.96737158	90.39506789	107.5349489	112.3979014	101.0619532	123.6215492	82.88063012	122.9484854	104.2293063	91.75467863	94.70688702	126.8071062	97.06289275	98.33971492	114.0840391	77.62885192	115.2410175
7.3396937	14.78044932	12.2492666	13.08839103	9.93426354	7.808641225	14.39641601	14.34568145	29.53543412	9.824574791	11.72160251	9.666624686	16.2775119	16.10961014	10.41168097	13.69888078	8.971290838	27.70659147
0.006475884	0.006386093	0.009483213	0.010605961	0.005012781	0.009133804	0.012658366	0.00635046	0.014734574	0.006436149	0.007802082	0.007478304	0.020373488	0.007447588	0.005689771	0.008598004	0.003940907	0.015513345
9.943778939	8.981011961	5.270612531	12.97857612	7.968987268	10.48545008	3.920946636	2.508793072	14.33828676	4.895157589	11.17406281	7.737425619	22.07726973	17.32697391	8.195991805	5.298089425	7.051165046	7.139542179
7.35039438	6.850740772	4.843684379	9.501423647	7.964415769	11.13652855	8.274513602	4.955212149	8.435989608	9.276690583	9.299233884	8.848400557	16.16434443	8.98827257	10.34036091	5.133720913	4.86045142	9.132704456
12.42913385	10.72597206	22.55444636	13.60472158	14.05553098	14.11121796	20.4638511	16.03906104	36.134566	16.46691216	14.90365133	8.720388805	23.03834494	18.20771927	24.11984863	20.27274729	13.39921481	29.82658408
2.489464721	2.505343424	2.759964786	2.779507595	2.000855666	2.068042495	3.344289391	2.064305606	4.572010592	2.792383313	2.500612374	2.08070197	3.350388051	2.695346448	2.415583094	3.138513761	2.633635901	3.777067018
10.44982859	8.165235648	8.390929172	14.46717946	9.098691098	10.15902908	10.5723437	8.008468198	13.43869046	8.981751127	10.39211944	10.5494972	15.65109334	9.406014671	12.05937089	8.827695304	7.766472373	19.71955571
0.174799033	0.229031863	0.197757304	0.333525507	0.182786882	0.176361777	0.198535096	0.203087837	0.230288293	0.16427817	0.174029262	0.183672795	0.244979142	0.250940512	0.181626748	0.242554565	0.183356952	0.244329592
61.42565735	86.23966425	61.99868723	102.2074942	69.42217209	54.19030718	123.1575191	35.79881368	148.0382966	79.41687628	84.69053929	65.60280816	148.4235507	94.72293654	63.3223619	92.13044299	63.08340897	141.5783841
93.84435273	98.27881532	91.39785158	141.0832441	128.7974646	118.2104046	152.7096649	66.27091919	145.7325692	82.01266769	95.20035199	112.7331165	168.4123303	109.9309293	101.6313556	81.89692948	88.82484246	156.3347771
63.5178183	58.56630249	44.35980454	76.24196604	78.77778631	50.28636176	61.13515164	31.4636492	56.25552333	47.30980871	58.28829962	79.18515298	82.28946089	59.47677931	64.30810058	47.05996667	38.98497441	80.99784216
142.7546898	178.0453868	109.6039525	197.2150568	137.5992008	170.4879239	153.81497	117.6483133	233.0313756	159.4596353	215.3399218	180.1931224	284.0195089	224.8430238	171.8403336	190.7758489	142.2067588	199.9388719
0.534006219	0.84809634	0.598160405	0.899741692	0.44163825	0.731090817	0.79192404	0.340061537	0.856906395	0.438796325	0.555709857	0.61688506	0.874915611	0.795313412	0.61905308	0.62759568	0.53011395	0.967154654
36.74139784	40.24120879	25.62537354	57.09546898	41.94440462	33.89615577	50.01549569	27.50438671	42.24688877	28.39044633	38.13491134	34.62569673	66.88411584	39.83910745	31.8953769	36.17291055	31.51792805	40.84410113
27.88291036	32.03275744	28.1371071	45.3713722	27.57322931	31.36341778	48.72009588	33.01430055	40.2478837	23.40755086	25.48624487	27.48700448	51.97342096	28.91267638	26.23292093	41.61895572	30.57080593	58.85040275
12.08906633	14.55034959	10.55072645	19.76697184	13.1288918	12.7890938	15.39012907	11.26650961	15.93441338	8.189719396	10.98557359	14.79947407	26.42924955	13.85772301	13.42098647	13.16358803	10.52409022	16.34661903
3.240874567	3.282594972	1.923612842	3.569836104	2.414070496	1.623528543	2.744715102	1.609589578	3.130312681	1.76855029	2.568609815	2.624380054	2.873337344	2.122218812	2.449021628	2.303718678	2.219683402	2.916031332
0.333286499	0.484300756	0.625160587	0.540778609	0.489258341	0.619044182	1.150110352	0.561343579	0.420043898	0.707891589	0.274350026	0.490247507	0.722751557	0.287812743	0.73802751	0.452661928	0.405180101	1.10504421
0.061693472	0.09859278	0.102715556	0.094576239	0.100093917	0.110983332	0.425369906	0.05863853	0.061033463	0.115952519	0.043605568	0.16884251	0.222977388	0.034186917	0.138890495	0.076052466	0.048147896	0.43403703
0.099485026	0.14624412	0.206341346	0.200979783	0.121316349	0.20671284	0.383782558	0.124891458	0.11131559	0.229752687	0.061598066	0.226538558	0.451888533	0.057629787	0.228509035	0.122159212	0.08583109	0.619155455
87.19432817	108.2203066	80.3520253	114.5313862	102.4972959	92.78687225	108.7743569	67.15089677	106.2389112	60.53943668	89.7398161	107.0088573	137.1664999	86.98826895	93.43614932	85.11960886	73.40080208	112.9726003
357.9386499	437.2122632	322.8171328	497.6479675	364.7739904	476.1258565	450.5959724	360.4929591	458.2632888	336.5257763	386.3974395	504.8381411	841.1154719	501.9715704	451.3496406	373.7653761	348.8465262	595.6566664
150.0403519	80.61009317	141.1336009	136.5586472	188.233834	142.250478	156.6519987	101.6740325	104.9864565	171.0352011	109.597241	181.8439356	266.5515458	130.7850977	200.2288103	120.2084653	100.1188966	274.7858719
0.070491133	0.070919129	0.081995954	0.109582256	0.062317824	0.098201006	0.16556985	0.087523414	0.113803012	0.084810591	0.05263336	0.094603862	0.15732439	0.072682499	0.095054383	0.077575557	0.063232849	0.174688186
1.419686244	0.671378102	1.542320987	1.884611914	0.895702265	2.264332153	2.892702521	1.39162433	0.972417296	2.249618511	0.947258669	3.273422086	1.792265032	1.005548565	3.730638012	0.932769325	1.221351714	3.208912928
1.484309352	1.017638082	1.160945364	1.909696647	1.195243634	1.621090838	1.759274597	1.099763215	0.59664565	1.259623452	1.094334454	3.461915537	1.613976119	1.556431128	2.765461155	1.349610825	0.743031702	2.066189308
0.944925609	0.679163899	0.596706205	1.073238332	0.789342641	0.940901029	1.164022341	0.961837815	0.64412525	0.906153195	0.891006113	1.811443403	1.382632861	0.744638447	1.705992267	0.53391671	0.746319983	0.926861464
397.1614433	525.2635885	328.2166145	551.5835363	412.5120232	426.6531265	565.5297356	305.412882	563.7996004	295.5727074	385.3846702	522.6923261	971.3743874	562.6947181	438.0650703	487.3229137	379.1721222	551.5023383
117.9108944	86.1653817	152.9150622	214.0558405	96.33344163	205.8799818	291.3090066	152.17472	188.5848704	138.2518682	118.1097325	243.290757	274.4392436	153.100439	228.0659951	134.3679723	87.8578411	275.727464
124.5333974	239.9943484	201.4128891	187.7481964	176.3259386	43.71240285	315.7617865	46.03091409	501.738237	33.78657446	113.671095	52.04475142	182.4027638	179.0062868	146.1649424	143.7944663	200.4464181	324.0648933
36.59979929	44.25089164	32.99198219	57.22500919	37.99343791	49.54682021	40.70839766	26.38264061	55.2490926	33.21033492	38.58818615	47.78711283	69.39777003	47.39235683	39.86375476	36.59003708	33.53423876	51.98500938
82.35905021	85.87790384	64.04394895	149.8151199	108.3139083	77.7023692	133.7927141	43.7243324	119.9935127	49.05448015	96.90524427	99.48481721	185.1307982	92.17739324	62.51326951	81.49673275	62.9377795	161.6633054
16.52208401	21.53236469	16.15676655	21.65582373	16.46338361	11.64901131	25.75315376	11.36955524	21.60166876	9.307504876	25.6493248	15.69734267	33.34460189	15.66947182	20.89816383	17.13992025	12.70084214	19.50828519
18.78829223	17.59181355	18.61074986	29.53198968	11.31624959	16.66270254	26.87196982	17.4763379	17.69564818	24.84456058	19.99565232	20.03832353	24.570365	19.05792424	26.15729148	18.85168318	15.24651025	43.26911867
4.228351604	8.025274234	7.715836053	6.137497199	2.910350944	6.513941875	9.654470248	4.476640336	4.298388903	4.680037312	4.499652825	3.210653276	6.306095616	10.11338714	6.417775252	7.337804421	5.279905259	11.33527431
24.49872693	19.54299048	66.47099612	51.38730719	40.97403976	73.91898552	86.31101795	129.4855548	129.2750575	170.5189296	34.17919185	39.39835294	62.78397727	77.73894173	69.45273405	64.09143433	59.32001227	122.3422555
8.502817799	9.588314823	8.210668347	17.07413494	11.29040244	11.74471426	12.48002371	7.15612907	11.1843746	7.829337103	9.819991936	12.93691927	11.93360273	9.036161445	8.820518168	11.49755355	5.661623712	12.94793465
8.070151423	9.796780695	8.654722436	14.32372048	13.77004674	9.342181861	13.99164564	5.409846667	5.956795327	6.965365591	10.31296642	8.88384075	14.89706016	6.73021957	6.407382895	7.626737254	4.474969634	14.62681499
55.81566494	73.62081912	30.17934444	68.62595889	55.99046425	39.57624478	48.14249105	22.23299031	43.73011331	34.61971049	52.62877565	79.93951786	51.04813999	49.93120986	49.27578361	39.77536198	30.20913536	49.20238333
10.92164726	9.376927748	9.436032394	13.66453792	10.00678773	11.85661053	11.60895502	8.032580095	12.9584311	9.635157065	11.18468719	8.919195497	16.4639605	8.855074976	10.6566033	10.32870172	7.431120706	14.57273437
48.9927516	142.5001852	24.18298513	148.3172811	104.2494527	84.10932684	94.9171542	14.83480789	128.1879321	35.09493989	70.04178449	95.75937969	225.0517566	93.44632641	85.92995565	48.0696591	28.73547879	68.74948491
47.2975951	54.53488891	31.56709181	94.27844161	40.50190686	70.31157898	78.52655107	64.20687387	81.66817698	112.3552351	52.28599325	64.59684501	77.84609574	60.253441	73.20202017	65.2861283	53.04428273	49.96132148
116.2817267	134.3662368	73.61550017	206.6926732	125.6261738	105.7132643	175.3733417	132.4748929	174.2969746	149.1084626	120.2417192	130.0610925	191.0962422	106.291597	162.2175173	121.3215649	108.1820157	121.521853
17.91396982	15.07238967	24.50298511	36.4724307	25.4566243	32.91788843	48.54933242	56.0857992	37.28013536	54.21119504	19.98133236	23.51817843	34.21071349	18.71718818	47.99420485	30.83798003	25.24561055	40.32473502
8.399119242	8.773470257	12.53842663	18.1047194	12.95095725	16.12282979	28.21791949	28.64375637	15.34443067	26.58157756	9.688236913	11.40360566	11.6451677	13.23965237	16.31558921	17.09038212	11.31662883	27.56779364
0.034887524	0.046936049	0.047163309	0.064003834	0.037272479	0.054961859	0.099353292	0.030265518	0.027158444	0.059480643	0.028294124	0.064023029	0.095847964	0.021450048	0.074366523	0.050836062	0.02676051	0.082872941
0.053859721	0.079073372	0.054784413	0.169806793	0.081369947	0.089791808	0.144237069	0.102348096	0.139791608	0.070427255	0.067491121	0.055291735	0.167334619	0.080634175	0.09686826	0.091320376	0.069043646	0.137395167
0.475860329	0.520487086	0.368568556	1.084532025	0.584190484	0.719329043	0.979427553	0.678987098	0.825689081	0.724442153	0.495925107	0.535258301	0.980253334	0.46071103	0.781347094	0.541551917	0.485459643	1.129779112
3.942028154	5.069051627	4.953685383	7.490747774	5.265921176	7.249402656	10.57097253	5.415236777	6.92116165	7.885288118	3.742749923	2.813099773	6.929393925	5.204848938	6.506792624	7.520134896	3.363721233	6.277964315
141.4294931	123.6105684	142.4309764	187.1619012	144.1236407	179.9101217	174.9531392	166.9842541	172.6482049	215.2977386	164.169101	191.5699079	220.1550364	150.4545663	187.6141626	166.0656067	140.1917694	195.3646177
46.79646341	55.7299059	47.77211543	77.78238971	69.58195553	64.43069844	62.67817953	52.73441442	62.02921382	46.4950484	51.67326773	66.86540822	90.03877119	55.92844041	63.58971452	45.98034809	44.97639139	62.80058471
44.8216444	38.09696822	46.08888696	41.77239897	41.49216984	47.84273316	47.45734118	53.17575451	49.96187096	53.94624645	41.46166746	48.40528325	41.99688545	41.36707621	49.26735234	45.44674636	43.4178402	48.68430474
20.64280413	17.34064956	17.63905967	29.83837176	15.38181869	36.75791653	20.40051547	22.99574943	38.54980872	39.53077047	23.46613394	25.06055732	34.93234525	27.54321929	27.61491064	26.32096455	23.25328997	29.45758362
33.8803317	25.95311449	27.66486731	35.43911705	33.52520177	48.37230364	57.60440413	62.98258267	44.84629144	83.6816963	29.60516007	42.11990811	43.56716507	30.26087278	39.14553595	39.87006368	39.59114272	41.90931676
79.36304028	95.1531652	67.03641948	104.0033963	97.52497343	95.5199255	107.2403537	70.50339124	98.73502196	70.31514567	76.57541828	94.92014375	123.4124474	64.27810426	87.54565962	76.45943035	69.37388044	97.77814879
13.7652951	13.20527894	13.53289729	17.24109871	15.84745729	18.6863858	17.88087512	14.88618717	17.41308223	20.04232921	13.9677774	18.44883519	23.45449181	15.10216902	16.86037253	15.56716057	12.56828859	19.57579911
8.542099331	13.14397634	10.05807351	13.03948229	12.12251679	16.37286903	20.44732931	12.39775703	13.51151897	15.64405559	11.418655	8.218416941	16.82776829	14.16207749	12.30695671	14.93910491	6.943209071	14.30812384
108.7175607	88.43819708	90.23568552	142.0654832	110.9506001	142.3119509	155.9390299	102.2961284	120.7415913	173.7125996	85.29431534	123.2247001	166.4095493	84.91552998	148.9763332	90.04846199	90.91951436	136.7369886
23.22549339	22.92759182	27.49458681	28.31020698	29.63825515	36.47530439	36.61719278	28.31171333	23.89309912	30.62123845	20.86379404	19.03055191	38.63993016	28.14721673	30.85691908	29.3168656	16.56770831	36.02443819

Drug	GSM530667	GSM530899	GSM531283	GSM531284	GSM531285	GSM531286	GSM531287	GSM531288	GSM531289	GSM531290	GSM531291	GSM531292	GSM531293	GSM531294	GSM531295	GSM531296	GSM531297
**Wnt-C59**	72.64176875	57.52035098	68.36393775	118.0220696	84.0530169	88.55633125	75.73963912	105.76397	87.13656185	39.22012844	49.0403659	61.34121553	99.01389685	42.89855969	82.77391332	65.62780729	124.3152505
**PCI-34051**	87.99775699	75.49572475	63.84411777	142.0007916	76.2707716	105.2946861	96.48650025	106.8572028	82.9355857	55.42563149	44.30931755	82.73382903	72.27948733	43.98258921	77.64299941	86.70621791	136.443187
**PFI3**	180.1723488	195.8986722	153.5300559	233.6043992	200.6844818	186.2858907	205.152081	258.1357737	167.5553702	132.1252403	174.1906647	172.3090412	173.1535174	138.0383528	192.1564467	155.9156695	222.1159571
**GSK2606414**	42.88492503	33.53080665	42.85627536	52.33329794	54.97392033	59.24815152	45.44331193	49.55216005	45.63364263	35.49953203	29.42758892	43.19700498	53.32433792	40.25005717	41.06125382	36.98412211	61.90594404
**AZD5582**	7.909491989	4.568622297	10.70844347	51.28152608	41.17414321	9.524110726	21.20259121	5.505719093	18.14760547	7.389352545	9.68682316	4.907698292	19.78421632	6.457559112	17.66904361	5.999319268	29.5790882
**CZC24832**	135.7493679	144.9479023	130.1933632	197.0342837	163.8725828	184.4819601	148.2574436	203.2901229	146.620655	120.628316	122.0209734	123.8431826	153.0512036	124.8142897	153.5348042	114.7963607	170.5312713
**WZ4003**	27.24509816	32.34950174	41.62130141	48.20691893	39.91287597	75.03981585	32.63361138	50.92560996	41.00137936	51.86858738	30.25474455	32.69293029	33.62731409	28.80375109	52.80393619	31.55770191	57.11002241
**VE-822**	20.80419945	20.00506847	21.61849391	52.7007963	26.13520737	40.52225792	40.64968691	35.47750148	32.79890662	7.883698668	9.176015851	26.78233774	19.0567892	10.31910481	39.76790872	27.06926499	56.04955004
**LGK974**	59.85006912	76.47778376	55.71099995	72.35782407	61.60561149	79.64259356	61.3886633	68.55617306	72.94697084	44.12748836	37.88353786	46.81566068	71.96598418	40.05842737	55.53746648	46.57705203	115.5363262
**OSI-027**	90.58204371	93.5535614	132.0041548	176.4385178	169.1325184	112.4035935	131.645015	111.5142753	218.0427697	159.5099106	100.2806096	52.88293639	185.9089185	61.07881093	130.0073225	135.1996583	193.4393902
**Entinostat**	7.516330401	5.012486928	5.750678843	17.59362466	14.14388808	16.82238472	11.68289357	8.625736827	15.94033295	4.52599608	3.784104787	12.35284097	6.819263105	2.753030131	12.37435076	6.638831716	16.40247366
**Leflunomide**	154.6171684	143.3769885	153.9637436	173.7433377	139.3869992	225.2172814	185.5602963	185.0113769	178.4252314	125.7753762	81.54879933	122.4897051	184.1557354	117.8474376	179.7971768	144.8366772	246.8824892
**IWP-2**	14.28418807	13.20379136	15.10343634	21.47574233	20.47199175	21.74898768	16.05073794	19.80694233	17.92366637	10.31181546	11.10709484	16.05555228	15.90552185	10.66900419	16.27334987	11.84558995	23.57753944
**SCH772984**	7.476176198	12.7251659	13.27682401	28.9343027	21.28514527	22.22826895	26.8513659	15.22214874	24.7628922	20.66333025	3.648516987	19.59348205	5.521276773	9.057022335	27.46894796	11.12090149	20.88258824
**EPZ5676**	185.435856	275.9881286	183.0283173	302.672719	224.3183284	325.714708	306.9429781	332.2705587	275.2557966	188.9639079	187.4226701	228.3436933	259.2149349	153.3351203	218.7621471	210.3038035	371.7586501
**Taselisib**	4.848079551	5.730541403	4.217893611	5.703248936	21.12592265	8.595621118	3.776464749	3.646596313	10.37015218	9.456775702	9.840156107	16.8789115	8.270708225	12.54301213	4.566563592	6.048672752	9.07092638
**Alpelisib**	24.89064657	18.94398865	19.68657673	32.00496024	53.84677769	41.34030469	22.33831775	22.21506953	41.6771646	22.72496731	31.67432125	52.36607196	36.36413082	27.71381058	17.75464746	31.50105413	44.59704115
**Luminespib**	0.058551973	0.107348063	0.066075492	0.107645858	0.077239757	0.199622519	0.11408271	0.122118032	0.140247298	0.089562238	0.049113349	0.175574956	0.0770553	0.098364356	0.083282165	0.081017897	0.109007781
**Lapatinib**	12.16542367	10.99691456	15.09621076	10.92838688	20.19769046	28.36636475	22.14935388	23.14807883	19.97248019	12.24819323	9.801928403	19.76697724	25.03687465	8.781747427	19.58981777	10.81581249	19.35833974
**LCL161**	129.7653872	109.529856	142.0639953	257.7463805	200.5817981	147.8135909	202.510533	152.2899117	124.5692973	101.9202106	103.7242807	118.658689	186.2491758	123.2018263	162.9150178	112.8668441	257.8439632
**Uprosertib**	21.6382202	18.54150704	20.53820951	24.43783614	58.30890556	23.75773379	15.90023016	12.09211165	26.96441673	36.4986851	26.72622376	25.01265366	25.32758345	27.49065916	24.70443251	16.29084458	32.60694546
**Sapitinib**	39.29662675	36.36506411	38.02864479	41.11000762	63.20707461	90.3748391	66.76940211	59.19137453	43.82537247	46.06253274	60.74752222	51.9237479	59.78648708	55.66154578	45.0662436	39.15207611	53.41704199
**Pevonedistat**	2.00586941	4.30007141	1.643341711	2.495447155	1.78956653	2.158206356	4.021152776	2.490447574	2.018312408	2.057522062	0.636489467	1.9472707	2.175008211	1.014057709	2.209372789	1.338074145	3.941967947
**Cyclophosphamide**	151.4127588	143.1404297	154.8456041	221.0415367	162.0074233	223.2597363	220.9379714	241.0426086	179.715129	103.5765955	115.5393818	125.999906	192.7043452	103.5984217	162.0759053	134.5485988	198.5894014
**Epirubicin**	0.243518121	0.254673998	0.252012743	0.385395934	0.354832155	0.478293814	0.401684674	0.198987696	0.433715407	0.693437331	0.127070321	0.508850897	0.270448865	0.240458273	0.290599155	0.243659556	0.34419059
**Linsitinib**	30.93067098	38.89047319	34.54296219	43.40458562	38.14541882	41.10437526	28.73836283	60.97444566	47.49572862	17.86626516	33.3806369	62.15498813	29.99863749	20.13827647	29.63150376	48.76289458	60.85927472
**Ruxolitinib**	112.1494125	119.8729413	132.2780947	139.9940859	165.7369814	164.9015664	157.0774657	133.8598471	167.2128586	102.1181046	99.12375872	140.4758924	144.9456677	105.1779861	157.5366886	105.5263022	192.7128807
**AZD1332**	26.2972937	34.86811575	36.52740607	63.67038122	51.75595722	31.6559396	32.69760686	31.57472206	44.93220585	73.74129368	35.52009786	50.62070502	43.26921991	53.78585543	35.63980502	45.7513968	39.92344051
**AZD1208**	184.799968	163.3600608	180.3152618	285.2710824	211.2842682	220.2568728	186.1906424	221.9199293	180.1927942	154.4069978	182.6684693	182.833312	216.7337348	136.2301743	161.2252967	158.7449786	242.7547045
**AZD2014**	7.943329399	7.950120243	10.33756847	9.102647405	9.63097277	9.338293742	8.946233429	5.090975302	8.681897839	14.13479694	7.034870682	5.046463752	9.33815039	7.10278965	8.7595578	8.776357201	7.570911261
**IAP_5620**	174.3212042	167.5153805	240.5131615	472.8823621	319.1697647	178.6183603	239.1361433	163.6948794	234.0250995	186.8952641	154.9825379	108.69276	336.7491153	146.294388	202.0777515	119.177146	321.4288521
**AZD5438**	7.83109347	7.382863185	11.79988858	26.19341577	10.73385075	10.3322866	11.74728904	11.31312393	8.591035053	8.031733815	3.921031166	6.070079372	9.891979806	5.31783532	9.4978528	7.173256286	17.58875794
**Temozolomide**	279.5842033	322.9055668	315.7866638	396.1918872	330.9059629	790.1471975	448.6230257	476.2068483	466.276156	218.859952	211.9925988	295.2904096	434.7234735	327.1311652	438.445466	261.9422255	520.5795531
**Dabrafenib**	54.6454935	82.38693399	109.8101685	89.43624175	116.9331089	182.6174218	161.798958	155.9337198	136.9540531	89.81974518	38.99514482	61.08647028	74.9329796	56.61793756	132.1734239	68.86802384	188.7294795
**Trametinib**	0.913712602	2.431237122	1.992840585	3.649371035	2.699149556	3.421858755	3.317482096	1.755380951	4.271338704	3.985768013	0.608906805	2.286597855	0.698830198	1.416494997	3.875972009	1.542520227	1.955516368
**XAV939**	75.71476204	69.85504958	79.71269417	75.02626322	93.23166133	107.6899073	98.06730389	84.83796633	89.56068048	80.89529988	61.7937656	74.75761129	108.0819922	67.5316653	88.08026279	72.42655867	104.1358566
**Talazoparib**	20.14614054	24.97232203	21.39227766	23.02229064	25.9142329	42.48415289	32.72685451	15.90792198	31.91840957	38.75975254	7.627585263	17.57576885	18.77478218	11.34038794	22.77188685	17.99529947	22.97549038
**AZ960**	5.364807765	5.082089281	8.944046504	7.679014049	10.19478105	12.05434276	7.685993484	6.078412497	7.727035924	10.0442379	4.624721316	8.23662395	7.471669328	7.13377045	6.494404602	6.727227429	10.39169073
**BMS-345541**	30.39594851	29.29520209	22.93237486	57.04884811	20.24701211	35.30116226	43.31089807	50.35819879	30.80394267	12.98591138	15.17895552	31.00408804	29.02977932	14.56864501	28.04591274	21.82634019	96.26075738
**Daporinad**	0.015666346	0.009612579	0.006281672	0.011902357	0.006460615	0.011493011	0.009074727	0.035862725	0.009322923	0.005013996	0.005107304	0.022245212	0.032312615	0.005823721	0.005953283	0.010451563	0.017582051
**YK-4-279**	5.784808952	4.841749231	6.887310346	10.53331913	12.31621177	17.2306988	15.41787874	10.26632024	23.72386765	8.354739545	3.440917804	11.13668919	10.83115915	5.278967819	7.674875981	7.971781807	13.6146949
**EPZ004777**	122.8227202	158.1434282	132.2083491	188.086348	178.8712818	200.9913238	148.804088	189.0682805	216.8904635	137.4262016	125.7959365	172.3024871	153.487078	110.5084892	141.7057724	149.2856802	204.1722552
**Fulvestrant**	14.88846279	14.0913046	17.30683265	17.64724977	15.74738356	20.81310659	17.40270035	23.53432245	18.39704131	14.31502342	12.83444969	18.06873158	22.98813347	14.57848406	15.52305729	14.00653629	18.67742228
**Tamoxifen**	34.16914101	37.57526554	30.70716796	26.69446901	31.97377276	44.96360187	39.58345858	39.16004124	35.04780148	29.5913083	23.71218499	29.51966997	41.09185845	20.0425382	31.65906875	29.96908603	30.80516405
**SB505124**	10.38483367	9.442162346	10.08666014	9.749161112	9.569995774	9.42271731	9.564872486	9.351524793	9.431182626	10.06720045	9.882997076	9.29764375	9.736320346	10.18348971	9.045057203	9.931577186	9.5578081
**GSK269962A**	13.61921586	20.35461104	16.70106484	21.55229451	22.9544101	17.27460655	18.05568031	18.03141636	17.38189458	17.62583991	19.25541757	13.79410944	16.5974914	19.93015153	17.30189488	18.23663661	17.32625229
**Bortezomib**	0.005870303	0.00811202	0.006403787	0.007687578	0.006871497	0.006217531	0.009316636	0.008082053	0.0063029	0.009124997	0.006083193	0.005199163	0.008336286	0.00640384	0.007451518	0.006522462	0.008199462
**Gemcitabine**	0.229255415	0.437288424	0.465072241	0.440198961	0.383567364	1.959869741	0.704277364	0.360223527	1.669496531	0.946674902	0.050931405	0.604536096	0.452972671	0.199763588	0.445443703	0.247611887	1.380941483
**Dinaciclib**	0.061506066	0.032497288	0.064942084	0.08902922	0.060243015	0.072014164	0.066044569	0.100101864	0.057671494	0.048029516	0.039572459	0.050495522	0.067196167	0.040553853	0.061100579	0.04680295	0.101569632
**MK-1775**	1.095279908	1.0723066	0.994967316	2.342831128	1.445920941	3.592959036	1.797981877	1.98880568	3.619040511	0.596744444	0.618521909	2.449763113	1.789819501	1.312334104	1.626618518	1.326122586	3.098822359
**Niraparib**	70.06713525	68.78540258	59.4978699	88.01945603	98.29590023	125.5566873	77.33686804	68.66418903	110.2211974	62.37120328	34.18017904	65.17695317	63.62311883	41.72431635	79.50250874	74.14409225	116.1239729
**Erlotinib**	15.03058043	13.66320097	12.12604952	14.69809512	13.93399548	24.54810965	23.35246189	24.19099753	14.98685495	9.377608692	10.94009972	13.30712806	16.7408194	11.3833763	12.2069388	9.729579081	15.2504579
**PRIMA-1MET**	76.76694129	67.25182599	88.75449672	87.03728757	148.5314412	101.9865493	96.8215105	86.40876714	118.2614887	113.0252909	45.53356926	114.139457	119.837369	62.36562452	137.6947698	80.81646148	181.3763162
**PF-4708671**	38.95941047	71.58339122	49.31058952	59.98348313	52.99947657	60.7502297	48.96509041	54.34190812	57.26894647	56.59718613	58.95678059	30.13173326	46.04572661	58.54508496	60.40176663	45.70536776	56.29557249
**Tozasertib**	16.31687249	19.557273	10.01449597	20.26921912	20.11869707	15.10564967	20.6401424	21.57961538	14.52688946	12.60110476	18.11134863	15.8860265	16.87030321	14.87993744	10.82146627	18.76655566	17.9319091
**GSK1904529A**	82.57443225	77.04477091	73.05328311	75.33093322	88.84251302	71.65670959	88.01401725	92.45154027	75.91759159	34.69257322	40.34889467	56.68684479	98.11648292	40.46814825	73.16878169	93.79422547	113.5723377
**BMS-536924**	4.909089171	7.51750457	6.074520908	7.145347572	6.553327419	8.560760568	6.395139964	8.276311425	11.39239018	6.37484207	6.93715993	14.04779039	6.013300058	6.879050743	6.631267264	7.764178539	9.397549652
**Oxaliplatin**	29.94087666	35.10730553	35.00791569	40.95383704	44.54434452	71.7119738	70.97144902	35.34158915	78.9928161	103.7297658	12.63858459	28.85710647	36.41193205	23.05104767	46.39848325	29.9999579	54.55873111
**Irinotecan**	7.806325757	11.11722543	8.476038849	8.669636593	13.42978153	16.11865472	23.35850629	10.40013472	17.75671675	27.66195417	3.409262574	14.82670159	13.45220314	7.914688649	11.4271056	7.920992083	22.19155773
**BI-2536**	1.48918089	0.907893213	0.592592511	1.209227948	1.014552887	0.641542816	1.33092442	1.312519943	0.491386654	0.240034353	0.992515333	1.83895669	0.951430171	0.536724213	0.367145091	1.244083326	0.666796425
**Sorafenib**	12.77761521	18.64758754	13.29235113	17.40902548	14.93695531	19.71516885	13.46798086	19.05610402	17.58159249	16.22055361	6.334574856	12.26447961	12.57645942	7.271431948	13.64527916	10.99650463	19.58274553
**Rapamycin**	0.108768026	0.11285123	0.116206084	0.11127451	0.103223676	0.155068453	0.143129247	0.119785467	0.133527616	0.210429169	0.068406035	0.066536325	0.11040304	0.090343638	0.108264514	0.093439785	0.100196297
**Crizotinib**	19.11528088	20.38583899	19.0418696	28.22990458	37.40976537	18.17663643	28.23977581	17.75188773	24.28373408	18.86222082	15.52453092	28.58706787	21.04266917	10.43283175	24.84598989	19.061527	43.97407726
**Paclitaxel**	0.036895207	0.021180714	0.033189285	0.05040645	0.05290049	0.122933601	0.078855209	0.076657355	0.113628346	0.04729735	0.016829585	0.043239087	0.097403125	0.017611637	0.050260226	0.035602748	0.104997005
**Dasatinib**	1.907617896	2.480860411	3.329386318	3.522342611	4.584236462	11.65916064	6.006607833	5.205729632	6.021972575	10.03042032	5.195219394	4.846081821	9.139567155	10.12204065	4.246017614	3.885137074	2.542500022
**5-Fluorouracil**	55.54127672	65.54052926	128.800901	161.4464258	100.6411856	253.2026944	246.6874344	58.82622517	318.0073071	195.3565638	45.89169923	60.13985999	127.4691629	65.08952263	171.2593082	68.32423627	313.3077389
**Obatoclax Mesylate**	3.549263	4.091775712	4.152042645	5.296304607	3.623897561	5.438447439	5.892236511	4.365190786	4.95551842	2.119815152	2.123064792	3.293605553	3.340062729	3.092511512	4.55865375	3.895007062	4.466787519
**PD0325901**	1.01360999	1.922484717	1.792695249	3.775425275	3.05674814	2.462999977	2.730657102	1.484378433	3.726783177	3.126963167	0.776401788	1.952518044	0.550020991	0.957302321	3.075108421	1.142856376	3.038569895
**AZD8055**	0.81334986	0.831434397	0.847855503	0.876075584	0.82875991	0.92771055	0.832361438	0.831304204	0.877936173	0.880677706	0.78786498	0.678513161	0.836700911	0.859038682	0.835212669	0.863474509	0.783828538
**Pictilisib**	3.622263031	4.488431494	3.192256151	3.454957422	4.704991066	4.801816495	2.755293297	3.125793196	4.599135493	5.044972401	3.401373368	4.268675255	5.195562802	4.540816773	2.984639513	3.439388308	4.173137996
**Dactolisib**	0.205311796	0.229451474	0.138175125	0.169601764	0.136147793	0.292897008	0.197118409	0.179849319	0.215559003	0.26801398	0.092732625	0.105620282	0.227296702	0.173739468	0.199102553	0.187457955	0.183724768
**Palbociclib**	20.64815698	26.93264534	26.66836956	35.11165826	26.15429581	88.88743634	47.37641657	37.13227422	60.1828643	40.23875119	12.11797519	27.84755416	29.09426192	24.50663721	41.01963929	43.16166402	40.37361024
**MK-2206**	20.17766347	20.51680603	15.72657452	15.57504053	31.22620791	28.77822029	16.65264968	14.62632462	21.54495825	21.77065392	15.64449754	15.50041354	33.26934984	16.32723799	17.5817474	16.14118131	25.57018434
**RO-3306**	20.12711845	21.45010404	17.10655391	20.72659591	17.4066122	19.51465023	20.29187538	21.80262839	18.63970916	15.12675301	19.95179449	18.93040852	18.32560753	18.61729938	17.16597468	18.68930952	16.76955682
**Alisertib**	5.701538357	4.215623267	4.107212563	11.65781487	12.58340289	9.086018405	9.95219856	4.647829624	7.997652633	5.224242163	1.813477144	11.79701038	3.902232541	3.897114925	2.738626786	5.282904613	16.06454964
**ZM447439**	13.39978959	21.07868297	13.67991311	26.62231305	26.69843553	16.55012713	20.41888678	19.24977953	14.18441478	13.43199265	25.02576466	16.15458325	16.58586016	25.15028891	16.11810989	15.489331	22.02636951
**PD173074**	42.59039183	36.16880108	42.13569923	50.49498685	75.76689816	64.41584842	49.68784575	60.80046538	53.47547035	39.21788403	39.07179297	56.69404753	70.40838958	48.39117149	47.02323051	43.90166637	53.8523389
**Mirin**	108.6363872	91.49971975	109.1284893	168.1465501	139.525536	161.9357106	135.8587623	101.6319325	151.4236551	117.6538069	69.73940568	154.9974934	101.2796923	94.81019702	132.9001328	98.77095804	161.688683
**Nutlin-3a (-)**	85.68902446	75.06674628	116.8491897	86.50505618	102.9178943	236.3177622	85.7202352	47.47260933	124.716072	351.4116759	27.1482313	126.4263772	94.16467398	128.5948634	110.8835973	103.7877935	45.48186904
**Wee1 Inhibitor**	5.006174115	5.586372547	4.061487351	10.1758506	6.549598499	15.77516081	7.738456937	8.807090384	11.03746237	2.965776205	2.036191087	10.73164666	6.299913028	4.12753039	8.637572409	5.010808296	11.43457181
**Doramapimod**	95.33255909	97.61111846	103.1743988	110.2810374	115.3376201	77.46300982	83.77835645	73.67891262	77.87823913	89.53767747	96.74134096	92.25892748	81.58914896	102.598466	84.13916805	81.28998645	81.84235682
**NU7441**	13.48615546	13.88244774	13.7599188	15.69634511	15.86717744	12.78478655	13.35899373	12.93181741	12.31059639	12.79709144	14.59795107	13.83375228	13.21819012	16.70064973	12.60179289	13.51349095	11.59232671
**PLX-4720**	49.98455542	51.44822467	65.15666315	99.95204165	94.02229235	157.9165947	86.86367362	111.0789966	76.1221753	115.0226515	51.20589148	48.78563089	84.06192845	78.45743447	80.72901067	56.55159353	136.5703444
**Staurosporine**	0.021762138	0.021810904	0.028428172	0.069345552	0.053838668	0.053970897	0.056505007	0.045362135	0.049571591	0.039082836	0.021664882	0.061154783	0.043089933	0.053356434	0.034056158	0.031545143	0.031616947
**Afatinib**	3.88648132	3.420167494	4.323120219	5.172116209	10.80980347	9.493067092	8.19505843	8.140554591	6.804582797	4.208228967	4.395906994	6.849595955	5.881455556	4.851186888	4.608553532	4.054997345	6.428308997
**KU-55933**	71.16554586	78.79105163	83.29802215	109.7879922	102.526644	83.0485331	94.70957274	67.11180616	85.76543228	70.96651982	93.35668819	61.26459245	77.67525994	108.4372891	74.43775249	71.90306053	65.80071407
**SB216763**	208.9632982	234.792167	298.1023357	264.9189257	250.0752778	237.1775799	172.0715747	148.1463009	250.5858433	251.7198192	135.7408535	102.7867593	163.0775573	170.8847313	175.0922411	158.6235475	145.6937231
**AZD7762**	0.555046927	1.121144586	0.7511003	1.652981803	1.447298897	1.719907807	1.341014481	0.932419889	1.701443178	0.6421044	0.532740318	1.236500579	0.940902296	1.025501785	1.61106588	0.700793972	1.446750578
**Axitinib**	17.30024387	28.55845683	18.9471988	22.9936609	33.0808749	21.19658302	26.38678356	25.17903829	22.50822242	20.85322265	21.58777289	15.24481196	21.29745654	19.17924614	21.68572871	20.98673748	23.35701183
**Olaparib**	60.06744768	69.03001004	49.36827825	85.65173889	83.54639959	94.01027981	73.23502789	72.59633147	73.8194654	55.06946117	35.11221918	57.21542189	68.96121586	45.59422076	47.73552719	52.81583244	88.00927692
**Nilotinib**	35.87431783	41.84996398	27.50770933	34.62163641	34.71659609	44.68905275	37.31777406	46.4955226	50.08757531	21.26661657	14.04515686	24.7636588	44.51924626	10.13046204	34.71827468	31.60124903	44.2164835
**Vorinostat**	3.546682576	2.861044535	3.008588895	3.831380398	4.277821175	4.270312597	4.468637055	3.343894565	5.441807614	2.872321506	2.010780059	4.613456901	3.733522999	1.624869439	4.167278123	3.298786691	4.825894745
**Navitoclax**	5.106137914	5.891096135	3.581365271	2.541463207	4.429626399	17.53493957	2.948112331	10.76475974	10.80610588	2.992400027	2.357805041	6.527431602	4.478968438	3.891378993	3.296989043	2.553607252	7.407480669
**Gefitinib**	19.89470573	19.60651742	21.04870064	23.05328751	27.67123058	36.54760468	37.46891746	39.34054443	28.4585511	20.81140235	20.50804717	25.54956261	28.59801931	18.62417152	21.25573276	16.03245691	29.38513345
**Docetaxel**	0.006983466	0.005522944	0.006246647	0.010311806	0.009704234	0.016266195	0.01644542	0.010585011	0.015826334	0.007994453	0.004654247	0.010343262	0.015396129	0.004892832	0.007648175	0.008060099	0.011741034
**Cytarabine**	2.862901356	7.098578764	4.408310087	3.662755838	5.262055292	13.44408188	6.416073212	4.130887707	9.66883268	5.489376994	1.034443274	5.161340175	3.809840368	2.422024043	8.79685365	4.130627228	6.473104157
**Cisplatin**	14.67737467	15.2251499	18.42216359	20.1829824	20.71085509	88.91234553	52.4208761	32.8517743	24.90892879	33.39468277	7.522149976	19.53802669	21.41984615	9.81281132	30.29315528	10.75832134	50.28606398
**Vinblastine**	0.01242116	0.012288195	0.013965689	0.022086041	0.024725267	0.042187195	0.027924564	0.041000583	0.030825759	0.014446557	0.006880207	0.031529829	0.025070094	0.009687718	0.022396893	0.020861903	0.029080664
**Camptothecin**	0.054602286	0.05918026	0.075596391	0.06517549	0.075515458	0.182862974	0.206855266	0.074380862	0.166537136	0.147209425	0.023571505	0.101068773	0.096345214	0.055611479	0.109737107	0.046807977	0.172411385

GSM531298	GSM531299	GSM531300	GSM531301	GSM531302	GSM531303	GSM531304	GSM531305	GSM531306	GSM531307	GSM531308	GSM531309	GSM531310	GSM531311	GSM531312	GSM531313	GSM531314	GSM531319
76.18789641	95.25205548	60.35868678	66.48372126	61.74209943	68.85387612	65.14858938	120.0521109	53.48068319	110.2566789	46.52639249	51.68488783	51.63892217	72.57471916	42.08885929	41.33396439	74.97062901	71.05913948
87.89500011	128.1421039	127.0350753	87.71508968	103.3154059	99.89154375	128.3291366	153.9182371	81.52947902	114.6177786	98.43314989	71.25800016	87.22892785	139.7169994	101.8860851	68.08308767	135.1599475	87.39743546
216.5594747	228.6106998	190.0451556	182.2337016	259.1250719	196.2474836	243.7655409	319.3142479	191.8662889	205.7364065	180.6608461	154.3684287	172.7736594	208.8530381	206.4139238	135.3220163	183.2123416	175.9531765
67.6723004	45.5021959	33.00526787	42.37588231	34.06663265	36.45491145	50.45217635	45.30257245	63.16707531	43.93921614	37.51472259	21.70382147	36.42714944	41.779026	38.31128737	29.08258261	53.67897102	44.16002178
12.71845	17.40777388	6.978670013	6.202608256	22.47124469	3.181816189	6.982473162	25.39121028	19.51791929	8.797652902	11.58794752	3.690026862	8.581993384	7.744803495	12.75069026	3.044402618	8.457397789	3.521161756
194.6830874	204.0473612	151.1911326	151.8276921	202.1419941	141.496044	180.1306756	233.6389169	149.879908	167.5254055	167.468155	120.6949923	142.2367851	179.6131177	213.4644841	110.4095196	165.8165869	146.8729127
49.23901613	52.65700786	36.31329153	35.58171533	50.24092631	30.05487639	26.64829907	47.95229409	47.67044664	75.44925369	39.30318878	24.5595173	28.81476189	37.04282274	71.65616231	35.14521267	44.3106781	56.54118232
19.62109052	40.55178049	22.85755667	40.48121565	53.67065597	31.40450116	48.69062071	104.5561609	18.27805182	32.22298655	20.26633868	32.09789751	27.18178634	43.7486563	23.2942283	15.63054574	25.75008198	27.69927576
53.90483795	76.45465468	56.38081766	52.83619157	40.14745888	49.94335104	65.29233875	109.701048	60.85270949	71.91026207	36.21883001	48.43572677	43.25663219	56.58131678	32.95337314	36.18317249	57.89741219	50.26928567
110.5107046	106.4629046	94.11531366	113.906501	132.8481539	119.8088859	99.35584129	150.01397	70.25667268	146.2833949	106.6287354	107.5830783	114.2664134	94.13044106	73.79814261	75.64608328	128.3779986	157.4762961
7.503547006	25.47532634	9.71899129	13.68505006	17.4721988	9.019055584	8.362402134	20.75701612	7.047190347	9.01143976	9.892075952	7.405460659	8.00247094	9.074934575	9.850754731	5.246507562	8.658460581	7.560439287
146.4028916	212.1395745	170.5897893	183.2844348	159.3047073	137.403842	139.4826927	181.2818394	138.9732421	152.9771327	107.9745371	121.0420418	123.6364731	158.3187959	122.4326366	91.65152581	114.8029414	160.3401985
11.09384846	24.77145177	17.32495742	15.97720376	17.03059708	13.78725367	16.95604523	23.9507323	17.68269136	16.21783733	16.36541693	15.02669532	14.54540696	16.75690294	17.23985178	10.54636287	15.20946376	13.7390571
11.38209431	14.95389099	8.938904581	26.03314107	9.492058889	5.79434342	13.41558978	9.867957846	26.70451648	16.28585628	7.754485888	6.647248879	11.70622465	12.89463422	12.01694138	11.20261636	12.77899224	22.21161015
207.0107819	327.1982857	254.6197061	278.4258993	294.3646228	253.0901135	301.4760269	511.4040321	252.0176768	266.6135389	208.1715837	199.4264441	275.702998	255.7815749	287.5400167	138.8514034	231.1406413	208.6092041
2.317458598	12.11764692	4.997865231	5.27532158	9.766172888	8.043896176	9.929888354	13.26873162	18.8599028	9.174698744	9.834183577	3.068614843	7.798260605	10.58605143	8.86518271	4.585192912	5.536189716	11.35600952
11.13188182	61.03924599	33.08331608	25.41835076	57.06146926	42.8826198	50.14768863	66.13790929	61.71291134	35.43979131	55.7695929	21.84128126	29.75341125	50.90641286	46.0428448	25.50737239	29.54881542	34.78335716
0.09130883	0.094644037	0.046412491	0.076907563	0.163918999	0.12561745	0.117544036	0.091449966	0.17535642	0.070310328	0.083956854	0.11961245	0.125264065	0.095572885	0.084399431	0.040391875	0.11293707	0.076151299
20.70188375	23.85660348	16.871019	19.78604448	38.89175454	31.51434511	16.84755015	31.41874184	20.61733565	30.97248666	18.96969384	18.76727146	23.40534073	20.9482121	23.10168861	18.47631714	25.54477236	25.59813922
253.6191109	161.7528826	96.39567942	128.144504	273.0734447	118.1688205	145.5081463	244.381053	171.1724632	123.5443969	98.97967839	128.7854036	131.7788136	116.3909358	120.6952159	86.96797953	144.6715506	91.9223794
3.876448414	19.76775147	10.56154794	15.06801311	29.55034148	12.70409351	17.68010106	34.30249615	36.13510315	22.69392026	17.57232567	7.876614859	21.51043904	20.1381575	16.7394461	6.30940932	16.68071661	21.93572036
39.11237276	53.20604557	70.62688195	38.59964638	49.03565903	66.64111288	51.30652072	41.84467115	71.64265392	50.191986	38.65135268	43.83330141	54.52224732	74.53523	55.69307743	33.98509289	49.54755916	50.55915673
1.714770022	2.354461976	1.754290525	2.09270076	2.593198295	3.138480085	4.251417622	5.297599394	1.36490901	1.715274087	0.852566848	1.59428952	1.879539595	1.417660877	1.89763751	0.676734202	1.412459827	2.668510114
169.0400705	218.4066582	154.8804177	205.9816423	218.2314512	206.561329	227.0009287	361.9762061	156.4496823	212.6689057	140.8683971	178.3766876	190.1308363	183.0735731	181.7961952	132.6723331	197.3346725	150.7269346
0.155151295	0.691484329	0.44869437	0.499335541	0.328830142	0.431177235	0.863757351	0.397515546	0.788625992	0.273308044	0.318113494	0.292532604	0.369098683	0.318985514	0.587536725	0.198433027	0.391051183	0.426779656
23.57838819	61.53179409	66.48344734	41.05029687	54.32343445	81.73913336	43.88154779	74.75125939	29.33069674	59.37078598	56.24063743	49.24432881	38.00801431	47.75379941	42.35303905	36.29186472	53.78208005	47.77705043
135.9437413	190.5651893	164.089823	130.5004951	113.3237866	105.8240087	118.1007639	159.4939947	145.7976434	102.9329581	118.5642494	116.764767	119.3590124	120.5115848	115.0931943	72.62882163	139.0683696	109.1550385
23.59964484	44.51678361	55.83699858	49.35652525	43.20842101	68.85081753	73.79767795	42.50549261	64.23595665	41.9302106	61.26814161	45.74579175	49.20051682	63.68471651	66.41100536	29.57483995	49.80967186	56.30445941
129.3382404	259.0774194	197.1395112	158.3843363	211.3891604	175.7978553	270.3293705	306.332218	188.8925436	280.957488	259.204546	154.6873182	172.3034298	196.8740522	244.5662737	124.0949245	204.3377921	165.9782653
5.464651724	5.454149179	4.277942869	7.274113918	12.53270824	6.046325211	14.51499602	6.90400931	11.54872271	10.9323085	5.33359123	4.976468372	8.180098825	8.021638888	5.004934264	3.970676163	8.740796181	8.985254941
303.7980572	249.8406761	93.6056657	135.1897306	264.1418065	87.04617293	209.4727763	329.1550365	201.4865449	154.26254	148.4592692	123.7160225	170.5046373	115.1671473	136.2748286	58.27265413	190.7394632	69.73611738
8.798190294	9.892161237	6.069543473	10.50728712	16.51685627	8.136916405	19.63195884	21.09691575	9.712840158	11.99036875	6.570455675	6.071493687	7.683181584	11.06162735	9.508108471	4.629702357	10.25973392	9.046862256
423.2737721	633.0123947	487.6696345	389.4222204	457.5587472	312.9239019	570.3718084	613.0454461	414.015229	456.9484182	271.1185693	354.7415087	304.5403791	329.4039299	341.3077545	313.3158211	511.3224209	379.5626157
159.9911116	146.9912089	61.7823032	118.7661687	134.113761	44.247783	123.8598827	137.3585667	68.06149783	180.3357929	40.86519049	75.56755785	76.88350335	54.24913772	94.21618399	66.38138403	140.219441	184.4408623
1.603968125	1.601479987	1.117668702	3.840760125	0.718679485	0.844840107	1.633522231	0.718725827	3.605454298	1.853104679	0.991580495	0.441133434	1.364443366	1.240646665	1.35478916	1.994195639	1.551989779	2.559545573
67.51735048	105.5817748	95.42709977	81.92366619	93.79596419	65.748562	71.82031463	71.26788556	82.01065289	72.56059871	76.8350762	75.40000796	72.21103277	83.7359835	81.36152635	47.98945434	83.45034653	85.16170803
23.91786821	37.06942987	26.65222557	19.86779985	32.17261342	34.94129351	68.44577723	40.5548926	39.60766428	16.34167325	24.53866301	28.03355605	32.1154572	23.68583746	38.81533925	11.98735568	24.89943044	21.99771447
11.11319892	10.93860377	12.39763261	6.336492571	7.974780167	6.092573263	7.446410072	4.501218848	9.738955815	6.512709502	9.98529874	4.538108321	6.68508437	9.370035934	13.65624727	2.999794395	9.623864095	9.147895611
18.48286355	41.02135998	28.09501955	31.47210257	38.87121816	31.11656867	60.58542829	55.62019528	14.85033439	35.54763963	18.82403996	21.53540782	20.79409317	34.40201574	20.55920004	16.35021266	28.53526726	22.75440911
0.006369169	0.01032923	0.016844581	0.012546131	0.016429046	0.03104278	0.014854254	0.035926173	0.007672715	0.013323231	0.010512203	0.016877134	0.013772964	0.020249753	0.007745257	0.011385422	0.005381665	0.009004469
16.61367034	12.44552886	8.954338163	6.005998318	12.32568501	10.24952507	14.22773865	10.20369782	13.61952095	10.76741727	5.620287153	5.432665966	11.50667722	5.953015797	8.894033788	2.97983455	6.678646294	9.425677928
129.4514044	244.8555081	182.0566653	166.8936753	163.5649578	163.5577837	189.8123108	326.2475007	190.5457082	190.1211862	155.6283023	159.9137591	173.1995453	166.558397	199.5156144	115.6714013	172.7973377	154.1958169
21.30236726	23.97331668	14.94467411	14.1088	23.00703675	19.25303486	25.96457857	36.60598239	19.33555116	21.67647694	15.87251609	20.03645222	17.80833901	18.93658847	22.14927526	14.38920797	19.81560773	18.01391846
43.8031232	37.22611791	32.36089466	32.59276414	45.87721845	40.33540959	46.26223779	47.48607508	30.5052714	45.72405399	21.56621595	32.9443853	35.78672668	28.15748498	39.39980121	32.4617914	41.29948754	35.27847556
10.12322729	10.18527276	10.22075745	10.93229564	11.54076193	10.27677231	10.50825567	11.22006051	10.7671165	9.574879953	10.14779867	10.46557979	10.39486503	10.75453535	10.05008197	11.55235649	10.1817149	9.252267153
19.47316062	20.55689696	16.08921221	22.93117929	22.4591154	18.3315918	19.99619608	19.04647156	19.37480813	19.11190565	16.7508922	14.07878759	19.15208815	17.17918837	17.32050481	12.64358721	16.81516407	17.09507983
0.011486407	0.007625587	0.007750829	0.008018444	0.007830045	0.006264332	0.011011637	0.008989444	0.006000513	0.00755291	0.008123095	0.005215048	0.007972199	0.007687488	0.008851388	0.005276101	0.005959231	0.008971716
1.510457029	0.754300635	0.321844335	1.475510617	0.68628314	0.428736613	1.69657024	0.739721228	1.061493967	0.265437129	0.327963845	0.373331941	0.609982229	0.292158471	0.732411274	0.195094401	0.185690338	0.594036569
0.107454517	0.071247992	0.074865642	0.057732545	0.075877043	0.050095347	0.092075703	0.106272464	0.047439231	0.097559156	0.050252729	0.03766361	0.048262213	0.067476615	0.065507661	0.038840534	0.054070092	0.083190645
1.48851592	3.114076158	1.909337536	1.452264401	2.136432563	1.754926436	2.517754546	2.747139848	1.484388825	1.849223994	1.415312679	1.220050654	1.458208447	1.530048612	1.764991273	0.899679429	1.334746546	1.90529706
41.08600171	116.6351568	84.91375823	83.98503977	129.7124905	82.59881823	93.31807234	126.6931363	100.6038849	71.05929908	76.64522884	50.41480766	77.99989196	123.1900154	99.82298836	55.2526873	70.77722081	73.94351887
19.58572109	14.24151277	17.037931	13.63353271	10.39101018	16.70836897	12.28466001	13.70578761	13.74467979	11.71500971	13.20765781	11.37750713	15.73260516	15.55612033	12.12361647	8.551426031	15.66714454	11.68983218
41.54261986	130.9463895	120.3871307	132.7736239	164.6991065	73.57222249	92.22840665	100.0368939	139.6679628	117.5111145	109.9747798	42.25949918	88.67887091	113.3302622	136.3378631	59.09407223	66.9379569	111.0708179
57.72927231	57.15875979	43.85289778	50.49129626	49.20480721	36.4581366	43.67917028	40.96434594	46.40003027	54.88849223	40.43798774	30.78994115	42.95278547	45.4116187	37.88823331	30.83439445	53.48476827	50.65046353
17.30997441	13.59239611	16.07601665	17.23384321	34.23630727	33.21000338	25.25837873	42.66738975	13.72293799	19.9405033	19.71224234	32.7399067	25.35215305	17.05540411	17.53351639	16.62179685	15.83470078	18.10802578
80.69781718	54.00270289	68.56261619	45.74638149	128.1668818	120.3865208	99.29814393	160.57021	62.6565817	97.52107908	66.56583555	69.85515549	75.15739347	87.370918	55.92305929	53.84853544	89.08319611	67.72279166
8.403072726	11.32155689	10.46280425	8.117404114	8.278930607	12.67987719	9.323300705	7.948436281	6.73223945	9.353643879	8.591247466	7.176918787	6.835581592	7.269887431	8.500173055	7.350523243	9.152023428	10.79020297
14.09906092	64.3775293	46.04130918	35.5356263	31.64326009	36.05661457	68.29970961	48.79606392	43.8124087	37.85055297	30.29269452	33.91139195	46.34534688	47.71103096	51.47543769	21.50502139	36.2214122	48.81458514
15.27107754	26.27716139	21.31668373	9.754666739	16.38511879	27.82652792	39.27159805	25.58752669	18.50314233	9.504671945	9.159440973	13.02208452	15.65078881	11.99977632	21.86827766	4.45035388	8.579864881	14.10908203
1.027491869	0.915387732	0.907413232	1.370163806	4.111621693	4.24221683	2.620291959	4.907004624	1.193198637	1.00804115	1.86712169	4.876020144	2.780009304	1.515846155	1.614616932	2.457037765	1.248791102	0.762517089
10.99521173	19.55092503	11.56283617	13.32320702	11.47884292	17.53775889	23.53717415	26.43426108	13.70862895	18.82487908	9.66483818	13.83227895	12.73432491	12.81622019	13.79940192	7.981662896	15.27031229	14.95192255
0.239179786	0.127173325	0.058868671	0.127142391	0.177762836	0.131503292	0.180267165	0.159466747	0.115384835	0.176554834	0.071188492	0.072589503	0.113023683	0.137572247	0.068509467	0.056127377	0.113328797	0.139171023
13.98056481	27.39849251	25.06032386	26.51469319	34.2175002	26.9104892	29.60600508	49.77617399	32.66691831	22.83009101	26.70720109	22.63969142	31.39226595	24.56709855	43.58070939	14.21944982	14.84972669	26.43534617
0.153733605	0.098944955	0.053082846	0.05412756	0.300864581	0.085457577	0.099635718	0.180273736	0.052419228	0.106894809	0.025741449	0.050696951	0.067815779	0.039032321	0.055157528	0.019545364	0.045637399	0.073477467
35.82365485	3.085469775	4.096915359	6.47328029	5.122342374	6.3130936	4.911041471	1.595287415	7.500204929	4.130461731	5.128176584	3.257672241	6.592454959	6.236088822	7.66751582	2.56159115	3.814068145	6.919342688
137.3475013	179.7452494	122.7947847	133.7533342	223.3452202	40.93218965	275.6053451	120.6384217	98.77354075	102.7271043	83.077391	56.07801342	68.26481469	67.08332911	137.8370604	45.53274332	82.71755442	162.9106704
2.641428167	5.780954616	3.57854945	5.393472994	5.073418246	4.197827453	4.45670588	4.089023749	4.256392253	3.990857501	4.493531391	3.178949879	3.729057618	4.288224371	3.981447634	2.382383653	4.00161371	4.269726228
2.162753948	2.000272816	1.335277033	2.589656798	1.138350196	0.802266978	1.894570973	1.311829354	2.041040431	1.737321718	1.170370299	0.61523625	1.14615696	1.506815848	1.954364812	1.458592213	1.382502063	2.05427517
1.042141153	0.825726073	0.804805673	1.01293986	0.867946418	0.735539251	0.69729778	0.774759604	0.838828222	0.810805737	0.802815549	0.699348964	0.818176259	0.759016125	0.66835475	0.663836508	0.797615528	0.899004365
2.11983125	4.331288471	2.501751867	3.543300859	5.968683917	5.11973343	5.943763362	5.086072296	6.289553012	5.740517484	3.238466253	2.491884237	4.29256663	4.666836584	3.016407276	2.48617798	2.926994527	5.658193267
0.238522176	0.171154906	0.105957792	0.193872589	0.287800671	0.228038557	0.382380332	0.196336481	0.305610561	0.314118693	0.087870781	0.116327932	0.212327462	0.190972062	0.137828689	0.12941796	0.207389047	0.292791016
46.24709212	28.26286828	36.30773894	45.92727902	55.90818877	31.61966306	46.4255953	35.58059709	44.18816472	58.12951457	15.23636611	19.41857917	40.61721766	29.50378587	25.3561891	44.16132499	42.83176127	69.22866587
9.948049643	24.16802946	12.29336958	22.39568575	29.13358623	19.28232395	22.04847232	29.35473111	30.5689068	30.22216083	16.33325922	8.96689756	27.35072802	22.85675966	23.32750958	11.38810808	17.34015036	26.04257137
23.23754375	19.71314575	18.88289301	21.85288376	24.95326592	20.46314485	20.89754521	22.88010392	17.64791771	20.19890417	20.53839443	24.59492558	22.32816393	19.2853815	21.03103019	19.85270986	19.84224321	19.02791451
4.074291256	7.907726873	8.232279652	4.638012086	3.598884321	6.54133752	8.926052337	6.151361848	17.78272705	4.520180944	7.901490836	5.586766716	8.070975128	11.45939612	11.78174749	2.755491856	4.23372542	7.569044975
22.49418443	18.02886851	16.5288842	16.03212852	22.53467224	18.82509892	20.72666941	23.76860449	15.97875575	18.93827209	24.81569738	17.03554115	18.95965212	19.6106831	22.12843846	12.9323241	15.55808719	15.76532597
59.3733784	65.41885473	63.13479234	50.33390698	55.95873225	92.92984383	62.88276227	128.0065127	66.50797746	67.09686438	59.01460539	60.22583012	63.88556954	66.98829939	74.76076919	42.1404085	59.59813461	51.23584544
67.61157686	132.32958	91.90680224	99.12593518	121.0809813	99.54166062	151.8372694	112.4153068	177.9463026	137.4553264	112.6086257	83.26545592	89.93952858	132.0038417	86.39136193	56.50540892	88.32607582	142.4996107
15.79470728	124.1234873	82.73231837	119.4600709	51.57010579	62.15873483	119.7937678	75.30946672	342.6013394	107.7335072	100.0484074	69.81723464	119.2519345	145.2513352	230.1775185	115.2372311	67.14461511	150.207912
4.763827588	14.73337958	5.64159117	6.410251653	8.878130299	8.768313863	11.30978404	16.05449021	6.257274967	9.898807846	4.420941653	7.610701354	6.212049169	5.555722156	6.627887273	2.955931582	5.795531311	7.746841458
68.79427197	102.8954313	95.39761101	106.4752883	83.46705384	83.2031644	85.95254555	102.8563813	113.5862606	78.07290423	108.0217692	81.33808133	90.90273614	103.9151506	111.7704047	80.98209314	82.69474838	81.06511798
17.72537687	15.36601336	13.62691264	16.74078979	17.58861075	13.41821058	13.86157421	16.06816534	16.17006399	12.49252619	15.06962479	15.04073095	15.81889316	13.92089021	15.14267117	13.04326733	13.97215945	13.43436261
113.5540372	100.5501826	65.28467117	58.42247969	103.3440898	67.05527675	98.0931406	131.2645835	133.8428861	138.3683908	65.39656366	57.72920598	74.44987005	67.10503935	92.30474852	63.49090535	96.74582493	123.7828064
0.068987989	0.085529148	0.036225387	0.058340971	0.057731089	0.084884196	0.069379837	0.06064881	0.054328087	0.041110389	0.072309411	0.051672048	0.057186295	0.053680916	0.074833446	0.020707651	0.058364677	0.055699182
8.35844674	6.869850196	6.574191393	4.988589537	5.973494623	10.34682698	5.673580045	7.523676115	7.281488264	6.2288297	5.844343152	5.365954743	8.014283854	8.606724755	7.65610681	4.198078425	6.005162905	5.747357955
220.2636971	92.4248338	61.26383442	114.6041405	120.8898933	52.38666455	62.39065496	86.16169712	88.92909653	64.87111402	74.84048097	77.48774647	85.61933712	61.69587556	71.27200813	57.5448442	84.97364445	75.12120946
284.544708	259.6806811	198.4185844	331.3110048	138.5132657	112.6411982	100.3657494	135.3413937	267.1444532	109.2888568	226.072785	127.2848918	185.7630359	183.6775628	247.3845713	124.6249301	162.3363466	158.8881325
0.977517275	1.349730428	0.810587336	0.901602686	1.472142181	1.326943683	1.26802412	1.998807672	0.80800044	0.955902527	0.616467335	0.843505856	0.774915478	1.165770709	0.916426676	0.540988579	0.906355303	1.217210906
20.8792519	21.07410355	18.46050281	23.57828597	28.14797883	21.8217716	18.65722702	30.70284813	15.86039494	23.96194754	20.13737824	22.43669773	25.75685463	17.69506418	21.70736923	15.91027215	18.67675396	24.8548774
68.78251091	114.7110482	86.61181175	60.41692741	62.43605239	121.1119041	124.5133405	165.2419812	113.8087017	69.630393	98.55758512	80.6496275	78.58987264	102.5568952	82.42217465	52.67370893	96.05806583	62.54080869
46.17959756	44.2378547	29.36111646	49.7776264	62.3304353	52.93428528	53.96484609	123.1241366	27.99557778	58.80841922	24.33284862	27.61704149	47.50931338	39.72042058	30.06453916	31.85904335	34.36810936	39.38789388
3.288203367	5.186565911	3.958320285	4.121927574	5.927436427	8.099413637	4.509240123	7.537145932	3.571163678	6.150454605	4.554252891	3.844281553	4.076809535	4.242910994	4.881758735	4.436626576	4.269297374	5.654428297
1.627728145	21.80767072	10.854236	5.237553082	3.695738997	10.34042035	11.72218866	9.851395342	5.536208346	8.392782501	10.94512829	10.55345701	7.210221546	11.81591837	14.10819257	9.003858619	9.833878259	5.909154507
37.34960501	28.82757421	30.33402922	23.51538335	24.52163106	32.03349345	21.60593443	25.97812775	23.40342023	26.36767473	20.77375838	23.75435883	29.48678865	29.42325451	30.16626399	15.7257865	23.82683772	24.96181488
0.022225201	0.010287912	0.009559061	0.009862205	0.02564975	0.014550009	0.016501201	0.018405935	0.013756276	0.013838851	0.010580719	0.007842391	0.013159991	0.007540678	0.008586409	0.004357815	0.007052854	0.011948119
4.42596836	5.710660636	5.964954111	3.622928573	3.363142078	9.08488209	6.693367125	9.11892348	8.037450379	5.64840971	4.372699915	5.058173978	7.165740844	4.126513587	7.407964148	1.864889614	6.248738405	7.341681918
54.67470017	73.33404218	28.13218456	15.10139745	38.73397478	43.74802461	40.54204516	83.15013364	24.18831098	21.84513584	20.0999898	21.86990114	18.82822852	22.13726649	40.57009168	9.291136812	24.5369493	20.67478269
0.024399303	0.029911816	0.029789328	0.024001507	0.035454556	0.029471111	0.041509945	0.036131496	0.037757151	0.029688317	0.009085532	0.014824051	0.035228274	0.015845975	0.020684156	0.0063411	0.014883752	0.030258476
0.106430246	0.210942114	0.132998755	0.074015528	0.112943868	0.110913972	0.226789828	0.107613012	0.122268897	0.068160322	0.058293499	0.073087583	0.08041488	0.075792739	0.154996609	0.035607817	0.063500938	0.084123509

GSM531320	GSM531321	GSM531322	GSM531323	GSM531324	GSM531325	GSM531326	GSM531327	GSM531328	GSM531329	GSM531330	GSM531331	GSM531332	GSM531333	GSM531334	GSM531335	GSM531351	GSM531352
60.75636751	70.33740896	44.7414887	86.32536322	68.62602788	60.42338329	72.29307937	61.52613966	76.04564691	65.11261705	67.66227569	74.21721059	81.6197634	51.26491469	65.75042226	55.4158602	65.57885546	73.64156892
72.18145352	73.81907711	67.15380432	114.486338	87.08374174	156.7175212	85.64016616	54.50634853	97.80532756	71.41271975	93.26961717	99.10902014	151.7192747	88.39114879	72.22769443	74.39412058	69.18695581	103.1565682
150.9119201	172.6031357	150.7086421	217.9566707	173.1759066	214.8899345	191.310746	155.3016	213.9856993	183.7704065	182.7073468	199.8460992	291.680356	202.2162273	171.0772131	171.7914637	138.4865736	222.3586713
32.62404239	26.80791452	26.84294844	45.65118258	44.33631705	45.54178566	53.27784289	43.76502332	44.8839548	52.28678008	28.86558127	49.83372812	58.13346059	25.72392933	39.91236826	36.15362764	29.72731703	52.16363527
4.246785108	4.519955638	3.001271012	15.14290285	14.22902815	10.52176035	17.95004303	11.41677982	5.044689273	11.59719628	3.804849055	13.17340681	13.31356977	6.054176198	4.952779784	4.217053657	4.705376039	11.45249839
129.3141503	137.3441382	127.8460672	165.4447379	138.3522098	213.9468091	145.7057177	135.8416555	167.249696	198.6583576	147.4530135	203.6869668	223.8528996	164.8706026	132.2039612	152.0530665	123.4079349	186.0762827
30.45825318	40.49804039	38.09758508	50.55777665	29.16741871	64.11415365	35.03081558	51.45963881	73.76245799	51.71132041	31.96120833	40.52435731	45.6701133	44.87823814	30.70402387	45.2128153	34.59092621	41.11147687
25.98007449	26.31392123	32.53493835	42.80286083	20.38060015	27.52457793	23.84480012	12.29535208	39.52069376	20.5958795	37.89328053	32.95965426	45.37143765	30.95031711	29.1241541	19.05570262	24.36849844	47.24065041
52.36283263	53.70121553	33.17021269	73.01680456	49.27644404	52.460258	68.72707486	48.08851085	93.47787775	51.74038618	51.45607893	64.98691714	64.09866287	48.90194266	52.68591782	55.44886791	41.73901152	53.85336531
145.6790487	155.8189199	156.0395493	147.1941221	145.1867339	118.9158474	174.3759783	113.4360269	129.7077746	133.0827542	106.7587591	98.28577347	110.0433058	92.41566782	88.21512061	110.9667496	90.41694591	183.6593731
7.072915412	5.795093819	6.761653575	11.87489363	10.07524056	13.81461645	6.332308363	3.76842819	7.790263879	12.59996753	5.554775022	12.37016586	12.2722524	6.952796247	8.03425015	7.425976515	6.518267268	12.18066729
137.1165462	111.1995569	135.7353183	162.9120666	142.4402446	154.063768	163.9585942	122.9956685	176.000853	157.3081339	119.0142376	169.9441094	178.6640353	117.3567848	155.0745167	115.1547183	120.9885994	150.597739
11.83370693	12.94919684	13.31807826	17.36142649	13.48154495	18.46942669	18.38196325	15.47300703	17.25430952	17.88236269	16.61266869	19.14813493	22.64404865	15.64777612	16.87347822	12.40979209	14.41609093	18.41680304
7.241422945	4.921184936	17.81356362	14.40830336	11.03199869	13.43726004	17.78464415	22.45877341	22.88975444	25.26398867	8.793374293	6.772264692	14.91026248	12.61205286	21.00749224	10.22590412	13.20185406	29.30234678
212.9194782	221.6418753	212.7508362	338.1255185	191.2992601	307.5352517	331.7028511	204.0951534	365.7132235	213.3109748	222.5889717	322.8013325	581.2791814	288.1666185	229.2017238	227.6598957	177.0707169	314.9041771
6.015930474	10.02854017	11.91686664	9.600938468	3.689580219	5.659510405	11.07685969	9.425867823	28.71979714	9.586483147	4.792072362	4.909912632	7.870616711	10.44958295	9.246849342	11.79307451	10.8640073	15.97764257
31.67987096	43.70687495	41.0830917	40.50707944	22.59266061	30.33475966	43.5026738	21.26008311	83.92753249	43.69563126	30.41995688	34.19292448	44.95194668	47.27493165	27.74612236	46.04837175	36.29961806	47.73958204
0.077977747	0.077624152	0.095195459	0.09287966	0.066584764	0.097196071	0.165082887	0.079722178	0.109808868	0.141000025	0.102714557	0.089663772	0.212403651	0.111712448	0.082440919	0.162259589	0.046255261	0.190194268
22.77777069	31.52755909	19.97474388	31.86999813	26.29547734	16.87835531	20.01550941	8.405179977	36.38162389	11.98104624	21.96910623	17.21262228	35.76870866	18.47455259	17.08584224	31.86645362	18.30222583	26.12070602
92.99038249	100.4962498	106.1265005	152.7831436	141.9266243	157.9790377	186.8077463	132.0564096	119.4946597	151.245996	106.7398586	173.3839164	219.9408361	111.3359847	106.743289	100.6706924	90.92484072	147.6352407
9.410769554	21.81152611	21.45660143	15.96299152	13.97436314	15.48656389	34.42819316	49.9678894	34.25364102	17.46515884	11.40522263	15.01499456	17.01143914	18.83134844	17.85408703	22.29686117	17.4476834	36.89695727
63.46558361	31.62105566	48.55892629	64.03125952	55.29272377	61.92927664	74.34609494	43.9658378	58.68674444	45.19273275	59.64592168	56.28895994	96.28590484	36.36004576	43.02625247	64.51734495	40.39851952	67.64403085
1.667456504	1.318035375	1.96928523	3.60116759	1.97402546	1.508006116	2.564764743	1.937436287	1.960253163	1.197195681	2.0113722	1.642910481	3.084737531	1.928740781	1.637682683	1.519844531	0.782903913	3.457563316
132.5558559	156.0022461	150.3747388	217.3513363	194.2060073	181.7343998	159.6352626	105.6896941	188.2005647	139.9386395	176.0134138	169.9710484	355.6871386	152.4902334	144.5381659	168.8041706	121.3132061	229.1046382
0.294938748	0.267187563	0.461868078	0.408748271	0.248558698	0.379512283	0.346712786	0.229404484	0.322606986	0.279814156	0.331442376	0.477625519	0.581971912	0.31720399	0.364370932	0.395301439	0.201054416	0.733858323
40.00523443	69.12191205	48.79340369	38.69925268	29.07563415	54.96295581	39.44117939	18.09517144	69.2071019	32.81551153	46.61264486	39.57362815	69.7651444	56.48831493	50.9797438	57.83415191	41.15047215	43.37159773
100.26627	76.52565355	84.12627539	138.0618437	115.4038488	123.6776652	138.5043418	111.6987551	120.3125509	166.5664735	100.1407555	168.7416521	187.1373183	106.8054079	162.6194136	92.25451471	83.16313039	149.4019869
46.12387849	42.65661699	49.18024182	65.31907381	29.03648044	35.09023483	64.7611837	60.88581377	49.89254052	65.16087405	51.85856723	41.57496847	41.62290762	49.59249018	63.39355462	52.98796542	46.36897696	77.62397329
151.8617589	210.2844837	151.1233156	234.9461851	174.8397021	221.4166715	230.5944604	167.8611314	278.6670272	203.8346778	166.7913743	209.3137348	299.2782558	247.3198688	193.1594769	193.2959586	180.936707	229.0776348
5.63999808	6.544583647	13.92134506	10.12118847	6.228918978	5.399258246	11.72426276	9.198899995	17.60050466	10.81335986	4.642219516	4.466498694	5.325818481	5.571104514	8.238345677	12.66402836	9.022273052	17.35320635
101.5600414	94.17158747	80.5503073	221.9061829	216.7150713	204.6281157	313.882369	186.3397023	141.1363933	255.0893215	103.3323742	210.0277044	225.2832119	137.6999722	117.4363379	97.89115452	73.97498899	201.3371659
5.410287857	6.770832218	5.338014164	14.65639777	6.685224737	8.94931065	11.58689952	7.906902505	10.9395689	9.283390161	6.837894358	8.22116729	11.39530965	8.073189334	9.179714211	7.749427768	4.941431946	11.50356783
377.1957025	322.1570688	326.0274121	567.9099062	342.7383291	429.3708179	349.0177046	275.1172393	472.7656559	446.5149851	398.6509134	610.0125642	808.4922221	417.2596335	472.5625061	420.1979638	223.5159459	534.6477223
66.10471303	95.03396654	103.3916849	96.52144268	89.97971305	153.2101289	143.2820406	100.9278846	173.1283507	104.0703712	68.1968585	146.3035751	148.4384176	61.46993386	125.6694	114.7565902	50.23632059	102.8345454
1.025098074	0.422452002	2.476102645	1.634766983	1.122431403	2.008496616	1.657099871	3.443199309	2.988776017	5.913978174	1.126892796	1.020179871	1.597875889	2.141038757	3.614313147	1.698390913	1.448113899	4.658878984
83.686099	56.0674181	74.58690035	87.15919635	80.32612454	82.47726374	102.6547396	81.66911905	81.59097118	112.2130261	75.23757329	103.2090742	89.0925681	59.92990413	83.60768487	56.48474609	70.43416736	94.51018042
19.18285982	16.5517694	22.01641121	30.01089195	13.73132994	19.3796183	33.46492746	17.87043059	25.29786086	25.96681353	30.31593052	28.19147202	43.70967267	28.80513901	24.12814249	22.1225649	13.84385089	29.03704415
6.738975693	5.050598477	6.274140866	6.899982786	5.471226103	10.57811489	13.82562618	10.11529974	5.254011816	9.023656908	5.037234085	7.225370782	9.949953758	5.021071872	8.549700803	5.977355635	5.922423085	12.87399351
23.41509091	21.79355372	17.15188171	46.92871715	27.41644734	27.28341236	33.6105063	16.38057288	33.96297384	26.85371517	32.91261112	23.52470409	46.01733024	30.97752522	32.49021247	29.58373479	20.2586407	36.98926051
0.015384472	0.012426827	0.009580075	0.027519392	0.021649055	0.006299703	0.010641962	0.003601598	0.012653474	0.00264453	0.025474339	0.004864868	0.04400331	0.019510033	0.011162199	0.009519165	0.007509602	0.025161415
6.811166559	7.592457198	8.423367203	9.16911456	6.566598262	8.64432118	25.10736517	8.61515066	12.69822872	18.45190796	6.437752638	10.32140213	20.99069507	7.369160238	8.561977385	7.730667934	6.050575175	22.19473709
129.4404406	170.2034209	151.5345353	187.2870255	107.1483088	184.8717644	219.7657627	143.1415582	263.4584894	212.0250867	178.7531492	198.2662306	307.590209	205.4437259	149.7131197	156.448926	131.7698005	203.8416847
13.69798205	18.50163467	16.62148043	18.32410628	17.10090297	17.98407273	20.26407802	13.97018605	22.86126226	13.97795177	18.17484039	18.00130632	30.21079592	21.30994625	18.64698198	19.17709123	13.40546761	24.83705058
36.90902179	44.10840169	31.61798289	40.40875453	30.72087477	30.13280769	39.4625273	30.19767174	64.37662346	30.11407492	37.00244414	33.96522572	53.21066356	37.21111181	36.82064159	47.46510706	27.95076978	37.29119004
10.1463189	9.94731006	10.2379979	9.802690586	10.49414265	10.65436786	9.366052085	9.060356578	9.71710414	9.559970993	9.869273645	9.462379078	10.7189308	10.37353596	10.53729658	9.865749769	9.776916387	11.00345163
14.60641767	21.16105422	18.99444714	16.73328362	16.53059573	21.08436326	21.40568138	15.17463205	20.52088418	22.22869095	17.76685232	21.87334265	20.34244664	18.88274472	16.73463158	16.51660662	14.61927878	23.68585977
0.006920613	0.006964088	0.007308801	0.011249555	0.006868201	0.006554728	0.008901278	0.008681477	0.010361441	0.007468305	0.005578177	0.007885877	0.010160822	0.008954962	0.006244337	0.009757714	0.006837489	0.010285971
0.430912169	0.210593205	0.53606007	0.80305086	0.335433307	0.26950495	0.408266363	0.231073284	0.171137275	0.511712184	0.2551308	1.424613745	0.843389913	0.888209865	1.875186366	0.55312015	0.166184232	0.903414441
0.034494894	0.048386221	0.045271928	0.089675922	0.061641579	0.057764244	0.068187035	0.061464412	0.059163538	0.052102686	0.044448514	0.047469558	0.114496896	0.049252178	0.058047258	0.050014914	0.046850166	0.084784629
2.20535603	1.720654844	2.422642895	2.089119168	1.782755642	2.148583432	2.137528108	0.825887935	1.566759601	1.727286308	1.919152936	2.408983283	2.845053381	1.742583272	1.913496279	1.313186776	1.336265201	3.358989808
54.52477002	49.98649767	53.26997178	83.22304329	46.1702947	71.29670011	79.58540413	57.73901076	90.99346905	65.15064894	62.30985496	62.03105507	127.8108352	65.18912301	77.95606993	61.34364662	57.25960122	93.01891361
12.60835279	8.589023064	10.33690957	14.9346136	16.29743913	19.58923196	15.78495129	8.698273821	12.98465981	10.55445263	11.48975148	16.96085555	26.95800093	11.39083469	14.21613993	11.17024684	7.200415684	11.55461889
68.04342981	65.456726	83.10451964	112.3139614	72.3091181	90.87159415	132.5127514	123.5183394	92.23814506	104.0572575	54.42752141	92.64623693	98.07345465	85.85489044	87.17412219	106.8488491	123.0345588	221.441323
41.61234746	42.59114494	46.54984237	42.32932131	39.39954184	59.2781784	55.32747827	51.02660976	63.26892782	77.36766491	38.01688753	58.60025779	42.6748139	42.58410953	40.02270663	47.15972588	37.64250051	52.92145309
14.87269045	28.05605606	19.16892892	20.35588317	20.43951727	16.05520277	20.83866807	15.17994433	21.28912371	12.03108963	20.9677049	19.24982727	31.90835979	29.00213583	16.11454861	18.59447566	14.01496015	19.53338124
59.85988968	96.2021919	63.15807029	69.78451219	75.68518454	57.25626253	115.8734134	57.86167364	134.2565414	55.02277115	71.51381409	54.38285427	118.3962996	63.07668275	62.00740915	88.04244708	65.82250934	77.18539369
8.916420297	8.035614453	7.574167748	7.204915482	5.032839308	9.674719565	9.010391269	5.954420662	14.09191648	9.483964972	9.264610455	7.761571631	11.63259693	10.1662513	11.66228749	9.273975818	6.467895111	10.33307635
35.88316315	20.4777016	49.60425961	52.85898703	19.87413402	47.38145687	76.56947132	65.81624199	61.21473674	62.47693474	34.2350598	59.36300563	73.72805425	33.97026106	71.24284956	31.62327523	29.14224253	70.95722834
10.36471371	10.62813727	9.086163846	14.1545468	8.276681085	8.617558535	20.91560157	15.39564279	10.70849213	11.483095	12.7673583	18.62785126	26.82920425	12.58476612	17.92357031	9.868751356	6.287449124	13.45622732
1.091832053	2.983191747	1.687952822	1.861515183	3.029536122	1.021693725	0.985348015	0.278871378	0.931879111	0.395915431	2.300875369	1.391307682	3.356122833	2.817482694	1.509614845	0.955514174	0.756530956	1.408895933
9.979155261	13.03535875	11.67722342	17.70347602	11.08282421	15.68025179	16.95654134	9.282815521	27.85229512	14.08980386	13.24820978	10.94220809	23.62300223	14.09557999	12.97241663	15.2015098	9.99157591	20.40527175
0.09699814	0.139343143	0.143096395	0.151294694	0.09599645	0.077408759	0.114544996	0.092774777	0.275237734	0.131460869	0.076074919	0.104582558	0.109448796	0.12806586	0.119510141	0.181852573	0.114350473	0.205505568
16.61998835	27.99815097	34.20602931	26.14765278	19.70374239	25.76685586	32.65950135	24.23988953	29.76318227	22.45591934	21.94473392	27.29423281	43.03486488	40.51459285	25.90623094	19.65180026	18.1877154	40.56414563
0.041774062	0.059292292	0.064802199	0.101833547	0.060683662	0.039607827	0.113893241	0.024753483	0.147592559	0.044589393	0.043668619	0.064280313	0.221511503	0.069369819	0.042138903	0.05460145	0.024286908	0.122451326
6.907294857	2.121125928	6.493467503	5.01096673	4.11477872	7.563433393	6.085359982	4.906963647	5.164674451	6.918862599	4.255670392	5.197699551	9.720274719	4.535990721	5.183673568	4.849357151	4.114592395	10.04272787
100.7852143	47.88833147	108.7944005	177.3282419	73.59973692	66.66335054	117.3825584	119.1863921	216.2616785	205.7744167	81.14763399	303.7864832	102.2605165	84.4129471	123.1544903	111.4800378	53.12768396	205.0504627
3.498122132	4.17873749	3.497032657	4.25935586	3.088201546	5.039466927	4.733093561	3.06114801	3.710830166	5.055161727	4.381045021	5.172735208	5.120840665	3.275666812	4.578484286	3.289771619	3.74444977	6.091535472
0.819066799	0.642449191	1.327966466	1.329548982	0.850486869	1.998435748	1.752342637	2.631339853	2.563467121	4.089589255	1.007750854	1.454965636	1.927240102	2.070599068	2.0869914	1.394073764	1.391326632	2.373133333
0.728948942	0.685575668	0.918951863	0.740687114	0.80983053	0.867503362	0.754514136	0.82608055	0.803723817	0.920308789	0.695471603	0.900515663	0.761466244	0.699492543	0.775710496	0.792543492	0.751264924	0.951972799
3.561166022	5.06512818	4.952942975	5.082393923	2.761498699	3.114306294	4.828494128	3.455441098	13.08848624	3.239911018	3.700719407	2.6394203	4.665017432	5.592351934	3.179518143	6.735486665	4.630672262	6.498089937
0.129871884	0.188291058	0.245381002	0.281045113	0.107370001	0.127516975	0.172894644	0.218065711	0.910833222	0.200556477	0.165458655	0.129454923	0.270452327	0.229877689	0.188310636	0.453276703	0.25253218	0.351745714
49.65064909	36.37828833	72.65339099	43.68020476	21.7268508	41.08637087	37.2614208	22.83228548	136.334677	45.87707318	38.89291577	40.70442592	70.71894209	33.00143233	66.20033776	81.72340521	25.46502455	74.99205164
13.17164778	23.67350287	26.70315469	28.79630637	14.47573977	15.57255096	20.11030286	24.47059609	62.07543168	17.0804022	19.59203822	18.7870927	20.62167023	24.32498962	16.60997491	25.85455893	16.59430289	42.49225656
17.32292937	21.6794882	20.15097537	20.28028478	21.20469015	21.66598695	17.88549095	16.80021432	19.83323189	19.75753628	19.96868329	21.98835416	23.95249443	24.29984069	20.63669711	20.4645182	16.86822219	19.8081016
4.426600961	6.040697626	10.12209182	9.490871328	3.472226297	8.592833092	13.54069217	6.060728401	7.033862921	4.128251283	6.723776313	5.779051817	10.64802282	9.691275552	10.87368335	6.556392587	4.810553623	14.82540112
13.01364005	21.72247833	14.79459667	18.9909924	18.47159652	17.8606438	20.53600404	18.97672304	21.15014444	20.35960646	18.88174123	21.02435905	25.31961845	23.62829485	15.88881458	16.26739589	15.99767328	17.97585332
45.83017112	72.08193613	44.85010443	74.3799646	63.9787032	49.40850429	72.41152856	46.09003119	91.15170815	39.78944027	68.38844001	47.06446062	110.5931827	62.01122809	55.96116438	57.69600817	44.73844357	75.48854055
78.58874269	91.49746702	90.16074465	145.3099326	82.68133413	114.2393754	158.8985578	144.4161637	144.1646334	180.256904	88.77441357	113.8454391	108.9071313	97.12837865	80.58336595	109.6188856	111.8602111	173.135246
67.75421713	61.98484564	180.1191085	132.0660367	22.04709224	169.9643733	131.6962716	209.9844599	304.252455	227.9348325	70.36677387	146.6614684	173.3653049	124.7487879	83.5147275	166.7585227	76.93124899	191.6168013
6.224375204	7.040827396	8.170147006	9.955571445	6.422834194	7.255623987	7.644386926	3.751551164	10.02477165	7.156456569	8.317667847	9.698346693	17.78387795	8.594874385	9.039100188	5.409030797	7.289308418	15.4435052
72.39535752	80.76448206	88.61464304	84.80989037	79.01237824	99.63620288	71.4541537	92.71424166	72.41887146	88.01095491	94.64486752	88.98948521	94.47669492	102.41446	91.0907762	78.52614062	89.98958847	99.49201555
12.31543963	13.67105904	13.96640534	13.54290215	15.11240729	15.56888069	13.22713141	13.93763586	12.25806563	15.79312622	13.70233413	16.3869582	15.90153163	15.63083521	14.88030445	12.31238079	12.35467112	15.68077177
44.50330506	111.1495436	67.4811822	87.60560655	65.93759385	93.37165206	127.0248355	111.7238632	147.9364565	98.35297367	56.70730345	101.0495315	157.026358	68.61686215	79.37418935	95.05905186	78.86362968	88.22787044
0.037663483	0.02705951	0.04017004	0.062416823	0.047487936	0.057249375	0.061110728	0.02855727	0.041586514	0.049011382	0.067851611	0.043597892	0.094922365	0.036172852	0.066988625	0.032084484	0.025264669	0.104143845
5.927141478	5.635174743	5.838112088	7.749741927	6.954953912	8.386769556	8.020838198	3.01249006	7.309493011	4.435688217	5.834803326	4.909484153	14.32631176	4.946487504	6.543742068	5.444931105	3.502270694	8.232691283
62.20551987	63.48370196	65.91911949	69.10756629	86.39442036	84.03727729	75.30528257	84.03481645	67.08247367	111.9909372	60.07500851	128.7097683	82.40019057	71.99536415	83.88216292	59.00900753	54.06353972	87.09621917
109.2471808	71.32933003	183.187355	134.7812058	163.1075904	272.3908316	130.7619231	169.9989151	134.7428504	266.122309	100.0294945	199.6383361	211.7164082	138.5491897	171.2350873	104.5665807	111.5018393	272.8488541
0.961345143	0.678964665	1.167646851	0.994002716	0.944280724	0.812253522	1.216527544	0.967338211	1.552835962	1.178042162	1.436399774	1.169349102	1.954049726	1.153837884	1.371990907	0.837486918	0.690341141	2.787018199
16.95169905	25.04199834	20.70783913	19.70375537	22.17791581	22.11589497	25.2298294	24.56321101	25.38875156	20.76934316	20.06862427	20.44611052	22.9314983	22.45928861	20.28830261	19.03289887	16.27564763	24.00025423
64.05868433	69.02233524	52.36084301	101.795222	57.45483182	73.90364279	92.66861093	40.30986703	98.61787928	52.12983657	79.0994674	74.78967645	136.771243	66.18518267	57.32183101	61.79673766	45.66056886	118.4983115
30.67050513	36.26383567	36.45205155	36.65973085	39.93852683	28.545455	37.21005688	13.540461	56.16051829	41.12868837	37.03054523	31.93717745	57.30378584	38.91168924	27.73370006	37.07844961	24.144323	65.43699069
4.050606906	4.751897282	5.093426715	4.985027068	5.08406682	4.20637001	3.581186244	1.768035366	7.900315283	3.417619664	4.226412677	3.313441271	6.754371925	4.803283334	3.59208181	5.596061563	3.448136673	6.985927785
8.715382349	9.561378431	4.663122136	11.833947	4.91932805	7.967377832	2.508442136	2.635182942	14.01696316	3.784716749	10.83436834	7.044860161	17.56238508	15.10904895	8.48316202	4.359854479	8.0858269	5.849674492
21.03937324	21.19960028	19.12350852	28.62901875	25.29917523	31.27100474	26.87243076	17.95792883	32.34201056	25.07322245	22.39515997	28.77053311	52.94065763	24.86708143	28.78758252	28.75557503	17.17935574	26.76061882
0.008903656	0.010394688	0.012864684	0.013202372	0.009642194	0.009054076	0.020381775	0.006844938	0.014604106	0.007954933	0.009062384	0.012501779	0.023092242	0.00918245	0.008447463	0.009980121	0.00527076	0.024399892
5.607511779	2.957508915	8.999043305	6.456472377	3.09855283	4.937769302	9.415949951	4.303640609	7.935252768	4.231825546	6.047781739	6.261310365	17.73295004	6.284745807	11.25081745	5.837908789	2.24106686	12.91204418
21.57528782	19.68577327	11.1180421	35.68395126	17.45031503	15.8179548	30.10664827	22.38080535	22.87888501	25.17872578	15.61980257	67.21930622	60.78760419	21.13967904	33.48732654	14.41638517	11.76338769	19.06539607
0.016849406	0.020867894	0.024193904	0.024270187	0.014748275	0.019324729	0.042033621	0.013342195	0.042719206	0.026579362	0.017073901	0.027756407	0.088534248	0.01480811	0.026096176	0.020240108	0.012476787	0.06110464
0.0692724	0.062835725	0.061001252	0.093469585	0.054909752	0.060644489	0.118327517	0.099301749	0.063039391	0.08513267	0.065409202	0.146289684	0.135783649	0.095059812	0.141167004	0.084617214	0.052623357	0.071246122

Supplementary Table 7. The drug names and corresponding targets

Drug Name	Pathway Name	Targets
MN-64	WNT signaling	TNKS1, TNKS2
Venetoclax	Apoptosis regulation	BCL2
AGI-5198	Metabolism	IDH1 (R132H)
AZD3759	EGFR signaling	EGFR
NVP-ADW742	IGF1R signaling	IGF1R
P22077	Protein stability and degradation	USP7, USP47
WEHI-539	Apoptosis regulation	BCL-XL
Foretinib	RTK signaling	MET, KDR, TIE2, VEGFR3/FLT4, RON, PDGFR, FGFR1, EGFR
Rapamycin	PI3K/MTOR signaling	MTORC1
PRIMA-1MET	p53 pathway	TP53 activation
Talazoparib	Genome integrity	PARP1, PARP2
Trametinib	ERK MAPK signaling	MEK1, MEK2
Luminespib	Protein stability and degradation	HSP90
CZC24832	PI3K/MTOR signaling	PI3Kgamma
PFI3	Chromatin other	Polybromo 1, SMARCA4, SMARCA2
Wnt-C59	WNT signaling	PORCN
OTX015	Chromatin other	BRD2, BRD3, BRD4
AGI-6780	Metabolism	IDH2 R140Q mutant
IRAK4_4710	Other, kinases	IRAK4
Uprosertib	PI3K/MTOR signaling	AKT1, AKT2, AKT3
Olaparib	Genome integrity	PARP1, PARP2
Axitinib	RTK signaling	PDGFR, KIT, VEGFR
Buparlisib	PI3K/MTOR signaling	PI3Kalpha, PI3Kdelta, PI3Kbeta, PI3Kgamma
Afuresertib	PI3K/MTOR signaling	AKT1, AKT2, AKT3
Osimertinib	EGFR signaling	EGFR
Savolitinib	RTK signaling	MET

Pyridostatin	DNA replication	G-quadruplex stabiliser
Ulixertinib	ERK MAPK signaling	ERK1, ERK2
Vinorelbine	Mitosis	Microtubule destabiliser
ZM447439	Mitosis	AURKA, AURKB
AZD8055	PI3K/MTOR signaling	MTORC1, MTORC2
Crizotinib	RTK signaling	MET, ALK, ROS1
Temozolomide	DNA replication	DNA alkylating agent
AZD1208	Other, kinases	PIM1, PIM2, PIM3
LCL161	Apoptosis regulation	XIAP, IAP1, IAP2
Vincristine	-	-
Podophyllotoxin bromide	Unclassified	-
OSI-027	PI3K/MTOR signaling	MTORC1, MTORC2
Ibrutinib	Other, kinases	BTK
Docetaxel	Mitosis	Microtubule stabiliser
Vorinostat	Chromatin histone acetylation	HDAC inhibitor Class I, IIa, IIb, IV
Gallibiscoquinazole	Unclassified	-
OF-1	Chromatin histone acetylation	BRPF1B, BRPF2
Cediranib	RTK signaling	VEGFR, FLT1, FLT2, FLT3, FLT4, KIT, PDGFRB
Telomerase Inhibitor IX	Genome integrity	Telomerase
UMI-77	Apoptosis regulation	MCL1
Sepantronium bromide	Apoptosis regulation	BIRC5
MIM1	Apoptosis regulation	MCL1
5-Fluorouracil	Other	Antimetabolite (DNA & RNA)
Niraparib	Genome integrity	PARP1, PARP2
BMS-345541	Other	IKK-1, IKK-2
XAV939	WNT signaling	TNKS1, TNKS2
AZD1332	RTK signaling	NTRK1, NTRK2, NTRK3
Sapitinib	EGFR signaling	EGFR, ERBB2, ERBB3
Taselisib	PI3K/MTOR signaling	PI3K (beta sparing)
Staurosporine	RTK signaling	Broad spectrum kinase inhibitor
NU7441	Genome integrity	DNAPK

ULK1_4989	Other, kinases	ULK1
VSP34_8731	Other	VSP34
BMS-754807	IGF1R signaling	IGF1R, IR
Camptothecin	DNA replication	TOP1
Vinblastine	Mitosis	Microtubule destabiliser
Gefitinib	EGFR signaling	EGFR
SB216763	WNT signaling	GSK3A, GSK3B
Daporinad	Metabolism	NAMPT
Tozasertib	Mitosis	AURKA, AURKB, AURKC, others
ABT737	Apoptosis regulation	BCL2, BCL-XL, BCL-W, BCL-B, BFL1
Dactinomycin	Other	RNA polymerase
GNE-317	PI3K/MTOR signaling	PI3Kalpha
WIKI4	WNT signaling	TNKS1, TNKS2
AMG-319	PI3K/MTOR signaling	PI3K (beta sparing)
Pictilisib	PI3K/MTOR signaling	PI3K (class 1)
Sorafenib	Other, kinases	PDGFR, KIT, VEGFR, RAF
Oxaliplatin	DNA replication	DNA alkylating agent
GSK1904529A	IGF1R signaling	IGF1R, IR
Erlotinib	EGFR signaling	EGFR
MK-1775	Cell cycle	WEE1, PLK1
Gemcitabine	DNA replication	Pyrimidine antimetabolite
YK-4-279	Other	RNA helicase A
Teniposide	DNA replication	-
Fulvestrant	Hormone-related	ESR
CDK9_5576	Cell cycle	CDK9
IGF1R_3801	IGF1R signaling	IGFR1
JAK_8517	Other, kinases	JAK1, JAK2
Zoledronate	Other	
AT13148	PI3K/MTOR signaling	AKT1
AZD7762	Cell cycle	CHEK1, CHEK2
AZD6482	PI3K/MTOR signaling	PI3Kbeta
SB505124	RTK signaling	TGFBR1, ACVR1B, ACVR1C
Obatoclax Mesylate	Apoptosis regulation	BCL2, BCL-XL, BCL-W, MCL1
Dinaciclib	Cell cycle	CDK1, CDK2, CDK5, CDK9

Fulvestrant	Hormone-related	ESR
AZ960	Other, kinases	JAK2, JAK3
Alpelisib	PI3K/MTOR signaling	PI3Kalpha
Wee1 Inhibitor	Cell cycle	WEE1, CHEK1
Nelarabine	DNA replication	-
Dihydrorotenone	Unclassified	-
PRT062607	Other, kinases	SYK
Ribociclib	Cell cycle	CDK4, CDK6
Picolinici-acid	Other	Inflammatory related
PAK_5339	Cytoskeleton	PAK1, PAK2
Acetalax	Unclassified	-
GSK591	Chromatin histone methylation	PMRT5
VE821	Genome integrity	ATR
JQ1	Chromatin other	BRD2, BRD3, BRD4, BRDT
Navitoclax	Apoptosis regulation	BCL2, BCL-XL, BCL-W
Elephantin	Unclassified	-
Sinularin	Unclassified	-
Sabutoclax	Apoptosis regulation	BCL2, BCL-XL, BFL1, MCL1
AZD5363	Other, kinases	AKT1, AKT2, AKT3, ROCK2
GSK2578215A	Other, kinases	LRRK2
MK-8776	Cell cycle	CHEK1, CHEK2, CDK2
Mirin	Genome integrity	MRE11
Alisertib	Mitosis	AURKA
Dactolisib	PI3K/MTOR signaling	PI3K (class 1), MTORC1, MTORC2
Selumetinib	ERK MAPK signaling	MEK1, MEK2
BMS-536924	IGF1R signaling	IGF1R, IR
Bortezomib	Protein stability and degradation	Proteasome
EPZ004777	Chromatin histone methylation	DOT1L
Dabrafenib	ERK MAPK signaling	BRAF
Pevonedistat	Other	NAE
PLX-4720	ERK MAPK signaling	BRAF
LGK974	WNT signaling	PORCN
VE-822	Genome integrity	ATR
GSK2606414	Metabolism	PERK

ML323	Protein stability and degradation	USP1, UAF1
JAK1_8709	Other, kinases	JAK1
TAF1_5496	Other	TAF1
Oxaliplatin	DNA replication	DNA alkylating agent
Nilotinib	ABL signaling	ABL
Afatinib	EGFR signaling	EGFR, ERBB2
LY2109761	Other	TGFB1
MG-132	Protein stability and degradation	Proteasome, CAPN1
BDP-00009066	Cytoskeleton	MRCKB_HUMAN
AZD8186	PI3K/MTOR signaling	PI3Kalpha, PI3Kbeta
Ipatasertib	PI3K/MTOR signaling	AKT1, AKT, AKT3
GDC0810	Hormone-related	ESR1, ESR2
BPD-00008900	Other	-
GSK343	Chromatin histone methylation	EZH2
BIBR-1532	Genome integrity	TERT
Nutlin-3a (-)	p53 pathway	MDM2
Tamoxifen	Hormone-related	ESR1
Cyclophosphamide	DNA replication	Alkylating agent
Lapatinib	EGFR signaling	EGFR, ERBB2
Topotecan	DNA replication	TOP1
Mitoxantrone	DNA replication	TOP2
Dactinomycin	Other	RNA polymerase
AZD5582	Apoptosis regulation	XIAP, cIAP
I-BET-762	Chromatin other	BRD2, BRD3, BRD4
AZD5153	Chromatin other	BRD4
Eg5_9814	Other	KSP11
ERK_2440	ERK MAPK signaling	ERK1,ERK2
AZD5991	Apoptosis regulation	MCL1
AZ6102	WNT signaling	TNKS1, TNKS2
Cisplatin	DNA replication	DNA crosslinker
Cytarabine	Other	Antimetabolite
KRAS (G12C) Inhibitor-12	ERK MAPK signaling	KRAS (G12C)
Ulixertinib	ERK MAPK signaling	ERK1, ERK2
AZD6738	Genome integrity	ATR
I-BRD9	Chromatin other	BRD9
MIRA-1	p53 pathway	TP53
PD173074	RTK signaling	FGFR1, FGFR2, FGFR3

RO-3306	Cell cycle	CDK1
MK-2206	PI3K/MTOR signaling	AKT1, AKT2
Palbociclib	Cell cycle	CDK4, CDK6
PD0325901	ERK MAPK signaling	MEK1, MEK2
Dasatinib	Other, kinases	ABL, SRC, Ephrins, PDGFR, KIT
Paclitaxel	Mitosis	Microtubule stabiliser
BI-2536	Cell cycle	PLK1, PLK2, PLK3
Irinotecan	DNA replication	TOP1
AZD5438	Cell cycle	CDK2
IAP_5620	Other	IAP
AZD2014	PI3K/MTOR signaling	mTORC1, mTORC2
Ruxolitinib	Other, kinases	JAK1, JAK2
Linsitinib	IGF1R signaling	IGF1R
Epirubicin	DNA replication	Anthracycline
Uprosertib	PI3K/MTOR signaling	AKT1, AKT2, AKT3
Doramapimod	JNK and p38 signaling	p38, JNK2
Fludarabine	DNA replication	Antimetabolite
Docetaxel	Mitosis	Microtubule stabiliser
EPZ5676	Chromatin histone methylation	DOT1L
SCH772984	ERK MAPK signaling	ERK1, ERK2
IWP-2	WNT signaling	PORCN
Leflunomide	DNA replication	Pyrimidine synthesis inhibitor
Entinostat	Chromatin histone acetylation	HDAC1, HDAC3
WZ4003	Other, kinases	NUAK1, NUAK2
PCI-34051	Chromatin histone acetylation	HDAC8, HDAC6, HDAC1
RVX-208	Chromatin other	BRD4
Entospletinib	Other, kinases	SYK
CDK9_5038	Cell cycle	CDK9
ERK_6604	ERK MAPK signaling	ERK1,ERK2
AZD4547	RTK signaling	FGFR1, FGFR2, FGFR3
Carmustine	-	-
VX-11e	ERK MAPK signaling	ERK2
LJI308	PI3K/MTOR signaling	RSK2, RSK1, RSK3
KU-55933	Genome integrity	ATM
PF-4708671	PI3K/MTOR signaling	S6K1
GSK269962A	Cytoskeleton	ROCK1, ROCK2

Supplementary Table 8. The samples included in the analyses

TARGET-OS cohort	GSE21257 cohort
TARGET-40-PASKZZ-01A	GSM530667
TARGET-40-PALWWX-01A	GSM530899
TARGET-40-PAMEKS-01A	GSM531283
TARGET-40-PAMLKS-01A	GSM531284
TARGET-40-PAMRHD-01A	GSM531285
TARGET-40-PAMYYJ-01A	GSM531286
TARGET-40-PANMIG-01A	GSM531287
TARGET-40-PANPUM-01A	GSM531288
TARGET-40-PANZHX-01A	GSM531289
TARGET-40-PAPIJR-01A	GSM531290
TARGET-40-PAPKWD-01A	GSM531292
TARGET-40-PAPNVD-01A	GSM531293
TARGET-40-PAPWWC-01A	GSM531295
TARGET-40-PAPXGT-01A	GSM531296
TARGET-40-PARBGW-01A	GSM531297
TARGET-40-PARDAX-01A	GSM531299
TARGET-40-PARFTG-01A	GSM531300
TARGET-40-PARGTM-01A	GSM531301
TARGET-40-PARJXU-01A	GSM531302
TARGET-40-PARKAF-01A	GSM531303
TARGET-40-PASEBY-01A	GSM531305
TARGET-40-PASEFS-01A	GSM531306
TARGET-40-PASFCV-01A	GSM531307
TARGET-40-PASSLM-01A	GSM531308
TARGET-40-PASUUH-01A	GSM531309
TARGET-40-PASYUK-01A	GSM531310
TARGET-40-PATAWV-01A	GSM531311
TARGET-40-PATJVI-01A	GSM531312
TARGET-40-PATKSS-01A	GSM531313
TARGET-40-PATMIF-01A	GSM531319
TARGET-40-PATMPU-01A	GSM531320
TARGET-40-PATPBS-01A	GSM531321
TARGET-40-PASNZV-01A	GSM531322
TARGET-40-PASRNE-01A	GSM531323
TARGET-40-PATEEM-01A	GSM531324
TARGET-40-PATUXZ-01A	GSM531325
TARGET-40-PAUBIT-01A	GSM531326
TARGET-40-PAUTWB-01A	GSM531327
TARGET-40-PAUTYB-01A	GSM531328
TARGET-40-PAUUML-01A	GSM531329
TARGET-40-PAUVUL-01A	GSM531330
TARGET-40-PAUXPZ-01A	GSM531331

TARGET-40-PAUYTT-01A	GSM531332
TARGET-40-PAVALD-01A	GSM531333
TARGET-40-PAVCLP-01A	GSM531334
TARGET-40-PAVDTY-01A	GSM531335
TARGET-40-PAVECB-01A	GSM531351
TARGET-40-PATMXR-01A	
TARGET-40-0A4HLD-01A	
TARGET-40-0A4HMC-01A	
TARGET-40-0A4HX8-01A	
TARGET-40-0A4HXS-01A	
TARGET-40-0A4HY5-01A	
TARGET-40-0A4I0Q-01A	
TARGET-40-0A4I0W-01A	
TARGET-40-0A4I3S-01A	
TARGET-40-0A4I42-01A	
TARGET-40-0A4I48-01A	
TARGET-40-0A4I4M-01A	
TARGET-40-0A4I4O-01A	
TARGET-40-0A4I5B-01A	
TARGET-40-0A4I6O-01A	
TARGET-40-0A4I8U-01A	
TARGET-40-PAKFVX-01A	
TARGET-40-PAKXLD-01A	
TARGET-40-PAKZZK-01A	
TARGET-40-PALECC-01A	
TARGET-40-PALFYN-01A	
TARGET-40-PALHRL-01A	
TARGET-40-PALKDP-01A	
TARGET-40-PALKGN-01A	
TARGET-40-PALZGU-01A	
TARGET-40-PAMHLF-01A	
TARGET-40-PAMHYN-01A	
TARGET-40-PAMJXS-01A	
TARGET-40-PAMTCM-01A	
TARGET-40-PANGPE-01A	
TARGET-40-PANGRW-01A	
TARGET-40-PANSEN-01A	
TARGET-40-PANVJJ-01A	
TARGET-40-PANXSC-01A	
TARGET-40-PANZZJ-01A	
TARGET-40-0A4I65-01A	
TARGET-40-0A4I9K-01A	

Supplementary Table 9. The cancer immunity cycle and representive genes

Genes	Steps	Cycle	Type	Immune cell type
IL10	1	Release of cancer antigens	positive	Multiple
TGFB1	1	Release of cancer antigens	positive	Multiple
HMGB1	1	Release of cancer antigens	positive	Multiple
ANXA1	1	Release of cancer antigens	positive	Multiple
CALR	1	Release of cancer antigens	positive	Multiple
CXCL10	1	Release of cancer antigens	positive	Multiple
PDIA3	1	Release of cancer antigens	positive	Multiple
HSPA1A	1	Release of cancer antigens	positive	Multiple
HSPA1B	1	Release of cancer antigens	positive	Multiple
HSPA2	1	Release of cancer antigens	positive	Multiple
HSPA8	1	Release of cancer antigens	positive	Multiple
HSPA4	1	Release of cancer antigens	positive	Multiple
HSPA14	1	Release of cancer antigens	positive	Multiple
HSPA5	1	Release of cancer antigens	positive	Multiple
HSPA6	1	Release of cancer antigens	positive	Multiple
HSPA9	1	Release of cancer antigens	positive	Multiple
HSPA13	1	Release of cancer antigens	positive	Multiple
HSPA7	1	Release of cancer antigens	positive	Multiple
HSPA8	1	Release of cancer antigens	positive	Multiple
HSPA12A	1	Release of cancer antigens	positive	Multiple
HSPA12B	1	Release of cancer antigens	positive	Multiple
HSP90AA1	1	Release of cancer antigens	positive	Multiple
HSP90AB1	1	Release of cancer antigens	positive	Multiple
HSP90B1	1	Release of cancer antigens	positive	Multiple
IFNA2	1	Release of cancer antigens	positive	Multiple
IFNA1	1	Release of cancer antigens	positive	Multiple
IFNA13	1	Release of cancer antigens	positive	Multiple
IFNA6	1	Release of cancer antigens	positive	Multiple
IFNA21	1	Release of cancer antigens	positive	Multiple
IFNA4	1	Release of cancer antigens	positive	Multiple
IFNA8	1	Release of cancer antigens	positive	Multiple
IFNA5	1	Release of cancer antigens	positive	Multiple
IFNA7	1	Release of cancer antigens	positive	Multiple
IFNA14	1	Release of cancer antigens	positive	Multiple
IFNA16	1	Release of cancer antigens	positive	Multiple
IFNA10	1	Release of cancer antigens	positive	Multiple
IFNA17	1	Release of cancer antigens	positive	Multiple
IFNB1	1	Release of cancer antigens	positive	Multiple
IFNE	1	Release of cancer antigens	positive	Multiple
IFNW1	1	Release of cancer antigens	positive	Multiple
TNF	2	Cancer antigen presentation	positive	Multiple

IL1A	2	Cancer antigen presentation	positive	Multiple
IL1B	2	Cancer antigen presentation	positive	Multiple
IFNA2	2	Cancer antigen presentation	positive	Multiple
IFNA1	2	Cancer antigen presentation	positive	Multiple
IFNA13	2	Cancer antigen presentation	positive	Multiple
IFNA6	2	Cancer antigen presentation	positive	Multiple
IFNA21	2	Cancer antigen presentation	positive	Multiple
IFNA4	2	Cancer antigen presentation	positive	Multiple
IFNA8	2	Cancer antigen presentation	positive	Multiple
IFNA5	2	Cancer antigen presentation	positive	Multiple
IFNA7	2	Cancer antigen presentation	positive	Multiple
IFNA14	2	Cancer antigen presentation	positive	Multiple
IFNA16	2	Cancer antigen presentation	positive	Multiple
IFNA10	2	Cancer antigen presentation	positive	Multiple
IFNA17	2	Cancer antigen presentation	positive	Multiple
CD40LG	2	Cancer antigen presentation	positive	Multiple
CD40	2	Cancer antigen presentation	positive	Multiple
NT5C	2	Cancer antigen presentation	positive	Multiple
HMGB1	2	Cancer antigen presentation	positive	Multiple
TLR1	2	Cancer antigen presentation	positive	Multiple
TLR2	2	Cancer antigen presentation	positive	Multiple
TLR3	2	Cancer antigen presentation	positive	Multiple
TLR4	2	Cancer antigen presentation	positive	Multiple
TLR5	2	Cancer antigen presentation	positive	Multiple
TLR6	2	Cancer antigen presentation	positive	Multiple
TLR7	2	Cancer antigen presentation	positive	Multiple
TLR8	2	Cancer antigen presentation	positive	Multiple
TLR9	2	Cancer antigen presentation	positive	Multiple
TLR10	2	Cancer antigen presentation	positive	Multiple
HLAA	2	Cancer antigen presentation	positive	Multiple
B2M	2	Cancer antigen presentation	positive	Multiple
TAP1	2	Cancer antigen presentation	positive	Multiple
IL10	2	Cancer antigen presentation	negative	Multiple
IL4	2	Cancer antigen presentation	negative	Multiple
IL13	2	Cancer antigen presentation	negative	Multiple
CD3D	3	Priming and activation	positive	Multiple
CD3E	3	Priming and activation	positive	Multiple
CD3G	3	Priming and activation	positive	Multiple
CD247	3	Priming and activation	positive	Multiple
CD28	3	Priming and activation	positive	Multiple
TNFRSF9	3	Priming and activation	positive	Multiple
TNFSF9	3	Priming and activation	positive	Multiple
TNFRSF4	3	Priming and activation	positive	Multiple
TNFSF4	3	Priming and activation	positive	Multiple

CD27	3	Priming and activation	positive	Multiple
CD70	3	Priming and activation	positive	Multiple
TNFRSF14	3	Priming and activation	positive	Multiple
TNFSF14	3	Priming and activation	positive	Multiple
CD40	3	Priming and activation	positive	Multiple
CD40LG	3	Priming and activation	positive	Multiple
TNFRSF18	3	Priming and activation	positive	Multiple
TNFSF18	3	Priming and activation	positive	Multiple
TNFRSF25	3	Priming and activation	positive	Multiple
TNFSF15	3	Priming and activation	positive	Multiple
TNFRSF8	3	Priming and activation	positive	Multiple
TNFSF8	3	Priming and activation	positive	Multiple
HAVCR1	3	Priming and activation	positive	Multiple
TIMD4	3	Priming and activation	positive	Multiple
SLAMF7	3	Priming and activation	positive	Multiple
SLAMF6	3	Priming and activation	positive	Multiple
SLAMF1	3	Priming and activation	positive	Multiple
SLAMF9	3	Priming and activation	positive	Multiple
SLAMF8	3	Priming and activation	positive	Multiple
CD2	3	Priming and activation	positive	Multiple
CD48	3	Priming and activation	positive	Multiple
CD58	3	Priming and activation	positive	Multiple
CD226	3	Priming and activation	positive	Multiple
ICOS	3	Priming and activation	positive	Multiple
ICOSLG	3	Priming and activation	positive	Multiple
KLRK1	3	Priming and activation	positive	Multiple
MICA	3	Priming and activation	positive	Multiple
MICB	3	Priming and activation	positive	Multiple
RAET1E	3	Priming and activation	positive	Multiple
RAET1G	3	Priming and activation	positive	Multiple
CRTAM	3	Priming and activation	positive	Multiple
CADM1	3	Priming and activation	positive	Multiple
CTLA4	3	Priming and activation	negative	Multiple
PDCD1	3	Priming and activation	negative	Multiple
PDCD1LG2	3	Priming and activation	negative	Multiple
CD274	3	Priming and activation	negative	Multiple
CD160	3	Priming and activation	negative	Multiple
TNFRSF14	3	Priming and activation	negative	Multiple
BTLA	3	Priming and activation	negative	Multiple
VSIR	3	Priming and activation	negative	Multiple
LAIR1	3	Priming and activation	negative	Multiple
HAVCR1	3	Priming and activation	negative	Multiple
HAVCR2	3	Priming and activation	negative	Multiple
LGALS9	3	Priming and activation	negative	Multiple

TIMD4	3	Priming and activation	negative	Multiple
CD244	3	Priming and activation	negative	Multiple
CD48	3	Priming and activation	negative	Multiple
TIGIT	3	Priming and activation	negative	Multiple
NECTIN3	3	Priming and activation	negative	Multiple
LAG3	3	Priming and activation	negative	Multiple
IL2	3	Priming and activation	positive	Multiple
IL12A	3	Priming and activation	positive	Multiple
IL12B	3	Priming and activation	positive	Multiple
CXCR5	4	immune cell recruitment	positive	B cell
CXCL13	4	immune cell recruitment	positive	B cell
CCL24	4	immune cell recruitment	positive	Basophil
CCL26	4	immune cell recruitment	positive	Basophil
CCL19	4	immune cell recruitment	positive	CD4 T cell
CX3CL1	4	immune cell recruitment	positive	CD4 T cell
CXCL16	4	immune cell recruitment	positive	CD4 T cell
CCR5	4	immune cell recruitment	positive	CD8 T cell
CXCR3	4	immune cell recruitment	positive	CD8 T cell
CXCL10	4	immune cell recruitment	positive	CD8 T cell
CXCL9	4	immune cell recruitment	positive	CD8 T cell
CCL20	4	immune cell recruitment	positive	CD8 T cell
CXCL11	4	immune cell recruitment	positive	CD8 T cell
CX3CL1	4	immune cell recruitment	positive	CD8 T cell
CXCL16	4	immune cell recruitment	positive	CD8 T cell
CCR7	4	immune cell recruitment	positive	Dendritic cell
CCL3	4	immune cell recruitment	positive	Dendritic cell
CCL4	4	immune cell recruitment	positive	Dendritic cell
CCL5	4	immune cell recruitment	positive	Dendritic cell
CCL21	4	immune cell recruitment	positive	Dendritic cell
CCL11	4	immune cell recruitment	positive	Eosinophil
CCL24	4	immune cell recruitment	positive	Eosinophil
CCL26	4	immune cell recruitment	positive	Eosinophil
CSF1	4	immune cell recruitment	positive	Macrophage
CCL2	4	immune cell recruitment	positive	Macrophage
CCL3	4	immune cell recruitment	positive	Macrophage
CCL4	4	immune cell recruitment	positive	Macrophage
CCL5	4	immune cell recruitment	positive	Macrophage
CXCR2	4	immune cell recruitment	positive	MDSC
CXCL5	4	immune cell recruitment	positive	MDSC
CCL2	4	immune cell recruitment	positive	Monocyte
CCL7	4	immune cell recruitment	positive	Monocyte
CX3CL1	4	immune cell recruitment	positive	Monocyte
CXCL1	4	immune cell recruitment	positive	Neutrophil
CXCL2	4	immune cell recruitment	positive	Neutrophil

CXCL3	4	immune cell recruitment	positive	Neutrophil
CXCL8	4	immune cell recruitment	positive	Neutrophil
CXCL6	4	immune cell recruitment	positive	Neutrophil
CXCL5	4	immune cell recruitment	positive	Neutrophil
CXCR3	4	immune cell recruitment	positive	NK cell
CXCL10	4	immune cell recruitment	positive	NK cell
CXCL9	4	immune cell recruitment	positive	NK cell
CCL3	4	immune cell recruitment	positive	NK cell
CCL4	4	immune cell recruitment	positive	NK cell
CCL5	4	immune cell recruitment	positive	NK cell
CXCL11	4	immune cell recruitment	positive	NK cell
CX3CL1	4	immune cell recruitment	positive	NK cell
CXCR5	4	immune cell recruitment	positive	T cell
CCR7	4	immune cell recruitment	positive	T cell
CXCL9	4	immune cell recruitment	positive	T cell
CCL3	4	immune cell recruitment	positive	T cell
CCL4	4	immune cell recruitment	positive	T cell
CCL5	4	immune cell recruitment	positive	T cell
CCL19	4	immune cell recruitment	positive	T cell
CCL21	4	immune cell recruitment	positive	T cell
CX3CL1	4	immune cell recruitment	positive	T cell
CXCL13	4	immune cell recruitment	positive	T cell
CXCR3	4	immune cell recruitment	positive	TH1 cell
CXCL10	4	immune cell recruitment	positive	TH1 cell
CXCL9	4	immune cell recruitment	positive	TH1 cell
CXCL11	4	immune cell recruitment	positive	TH1 cell
CCR6	4	immune cell recruitment	positive	TH17 cell
CCL20	4	immune cell recruitment	positive	TH17 cell
CXCL12	4	immune cell recruitment	positive	TH17 cell
CXCR4	4	immune cell recruitment	positive	TH17 cell
CCL1	4	immune cell recruitment	positive	Th2 cell
CCL17	4	immune cell recruitment	positive	Th2 cell
CCL22	4	immune cell recruitment	positive	Th2 cell
CCR6	4	immune cell recruitment	positive	TH22 cell
CCL20	4	immune cell recruitment	positive	TH22 cell
CCR4	4	immune cell recruitment	positive	Treg cell
CCR10	4	immune cell recruitment	positive	Treg cell
CCL1	4	immune cell recruitment	positive	Treg cell
CCL17	4	immune cell recruitment	positive	Treg cell
CCL22	4	immune cell recruitment	positive	Treg cell
CCL28	4	immune cell recruitment	positive	Treg cell
STAT1	5	infiltration of immune cells into tumours	positive	T cell

IRF5	5	infiltration of immune cells into tumours	positive	T cell
KLF2	5	infiltration of immune cells into tumours	positive	T cell
ITGB2	5	infiltration of immune cells into tumours	positive	T cell
ICAM1	5	infiltration of immune cells into tumours	negative	T cell
EZH2	5	infiltration of immune cells into tumours	negative	T cell
DNMT1	5	infiltration of immune cells into tumours	negative	T cell
VEGFA	5	infiltration of immune cells into tumours	negative	T cell
EDNRB	5	infiltration of immune cells into tumours	negative	T cell
CD28	6	Recognition of cancer cells by T cells	positive	Multiple
ICOS	6	Recognition of cancer cells by T cells	positive	Multiple
ICOSLG	6	Recognition of cancer cells by T cells	positive	Multiple
TNFRSF9	6	Recognition of cancer cells by T cells	positive	Multiple
TNFSF9	6	Recognition of cancer cells by T cells	positive	Multiple
CD27	6	Recognition of cancer cells by T cells	positive	Multiple
CD70	6	Recognition of cancer cells by T cells	positive	Multiple
TNFRSF4	6	Recognition of cancer cells by T cells	positive	Multiple
TNFSF4	6	Recognition of cancer cells by T cells	positive	Multiple
TNFSF14	6	Recognition of cancer cells by T cells	positive	Multiple
CD40	6	Recognition of cancer cells by T cells	positive	Multiple
CD40LG	6	Recognition of cancer cells by T cells	positive	Multiple
HLAA	6	Recognition of cancer cells by T cells	positive	Multiple
B2M	6	Recognition of cancer cells by T cells	positive	Multiple

TAP1	6	Recognition of cancer cells by T cells	positive	Multiple
BIRC5	6	Recognition of cancer cells by T cells	positive	Multiple
MDM2	6	Recognition of cancer cells by T cells	positive	Multiple
MAGEA4	6	Recognition of cancer cells by T cells	positive	Multiple
TP53	6	Recognition of cancer cells by T cells	positive	Multiple
PDCD1	6	Recognition of cancer cells by T cells	negative	Multiple
PDCD1LG2	6	Recognition of cancer cells by T cells	negative	Multiple
CD274	6	Recognition of cancer cells by T cells	negative	Multiple
CTLA4	6	Recognition of cancer cells by T cells	negative	Multiple
BTLA	6	Recognition of cancer cells by T cells	negative	Multiple
VTCN1	6	Recognition of cancer cells by T cells	negative	Multiple
IFNG	7	Killing of cancer cells	positive	Multiple
GZMB	7	Killing of cancer cells	positive	Multiple
PRF1	7	Killing of cancer cells	positive	Multiple
PDCD1	7	Killing of cancer cells	negative	Multiple
SMC3	7	Killing of cancer cells	negative	Multiple
VTCN1	7	Killing of cancer cells	negative	Multiple
HAVCR2	7	Killing of cancer cells	negative	Multiple
MICA	7	Killing of cancer cells	negative	Multiple
MICB	7	Killing of cancer cells	negative	Multiple
BTLA	7	Killing of cancer cells	negative	Multiple
VSIR	7	Killing of cancer cells	negative	Multiple
LAG3	7	Killing of cancer cells	negative	Multiple
IDO1	7	Killing of cancer cells	negative	Multiple
IDO2	7	Killing of cancer cells	negative	Multiple
ARG1	7	Killing of cancer cells	negative	Multiple
ARG2	7	Killing of cancer cells	negative	Multiple
NOS1	7	Killing of cancer cells	negative	Multiple
NOS2	7	Killing of cancer cells	negative	Multiple
NOS3	7	Killing of cancer cells	negative	Multiple
TGFB1	7	Killing of cancer cells	negative	Multiple
IL10	7	Killing of cancer cells	negative	Multiple
CCL28	7	Killing of cancer cells	negative	Multiple

CXCL12	7	Killing of cancer cells	negative	Multiple
CCL2	7	Killing of cancer cells	negative	Multiple
CXCL8	7	Killing of cancer cells	negative	Multiple

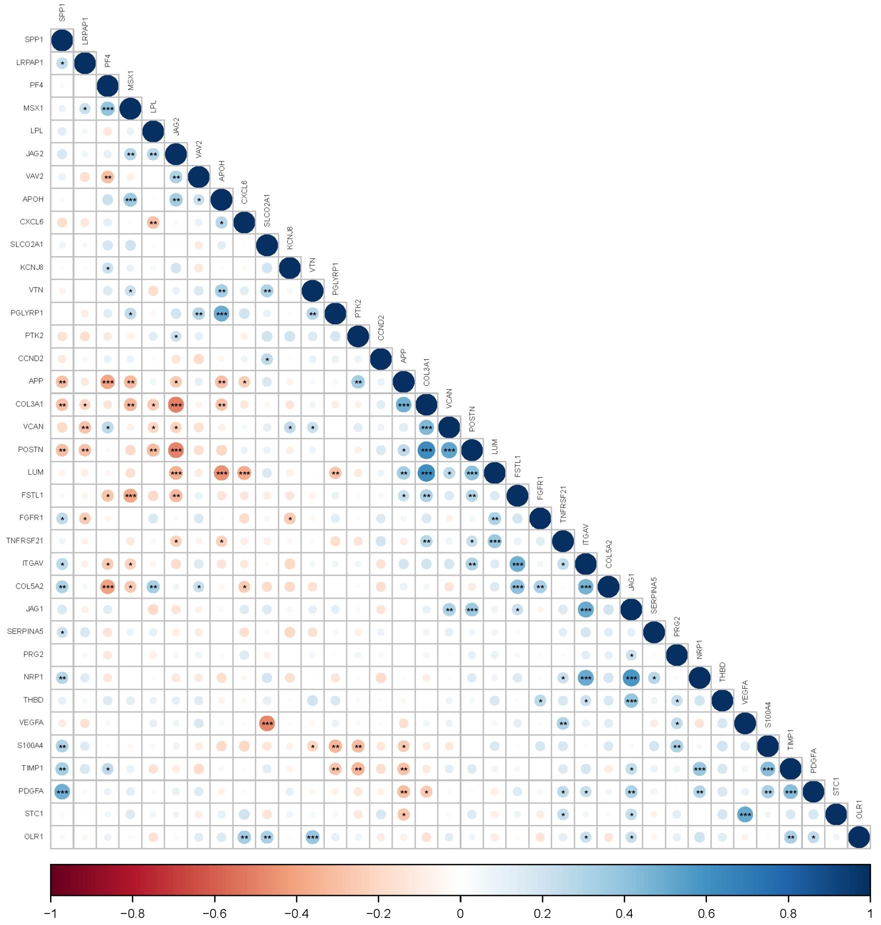

Supplementary Figure 1. Correlation of angiogenesis genes in TARGET-OS database.

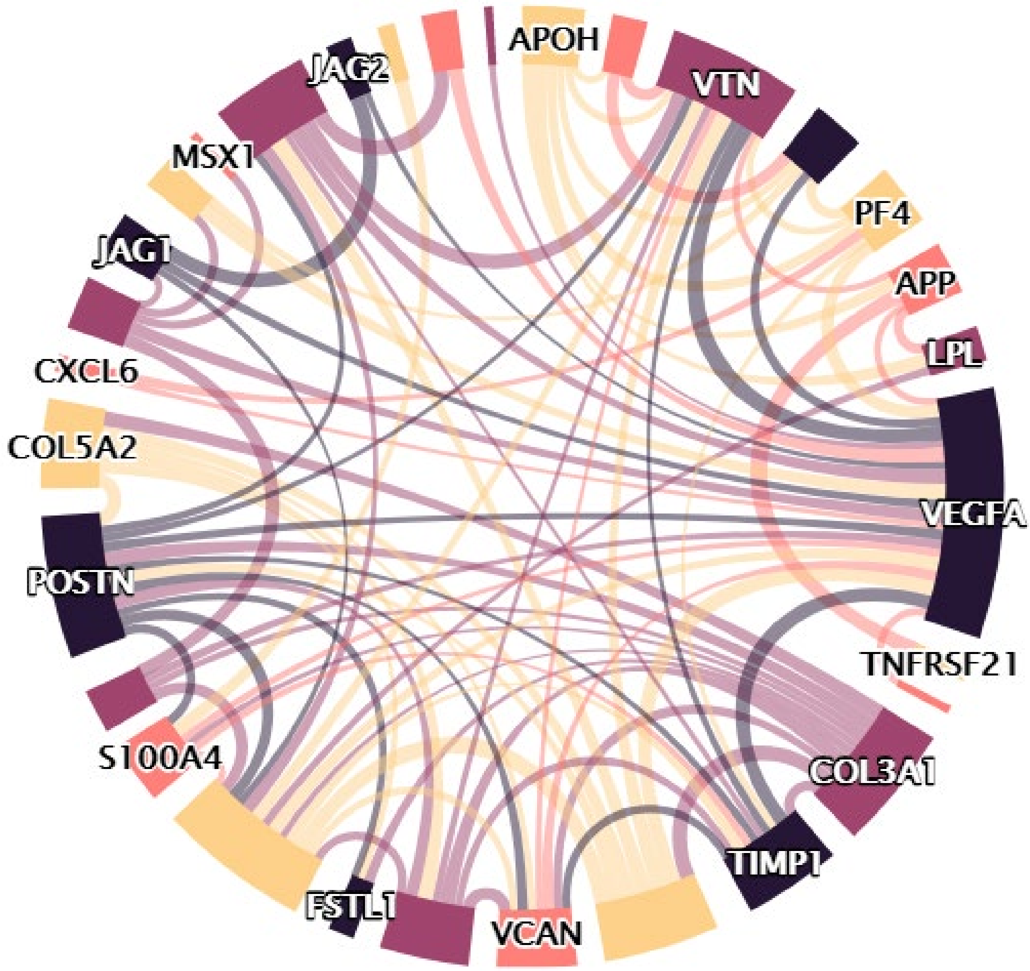

Supplementary Figure 2. Interaction of angiogenesis genes in the TARGET-OS database.

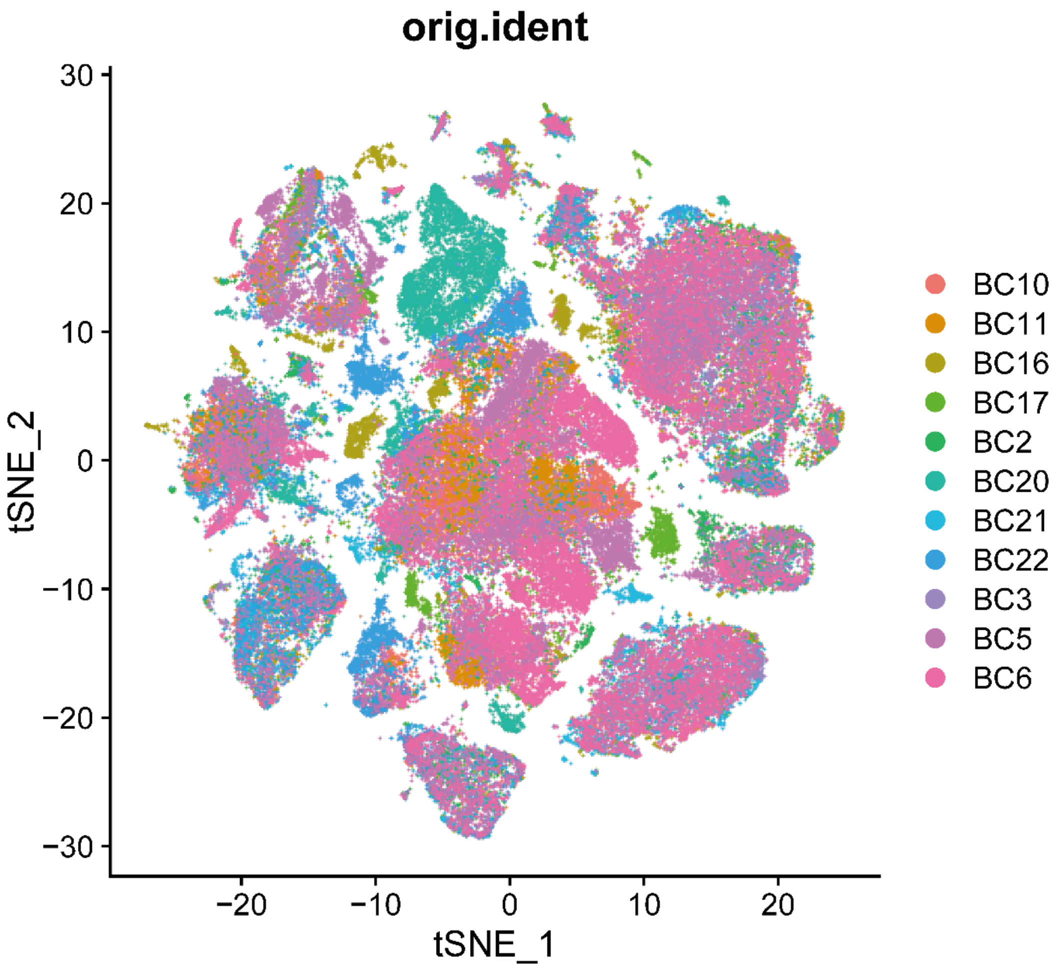

Supplementary Figure 3. The t-SNE plot showing the batch effect after correction by harmony algorithm.

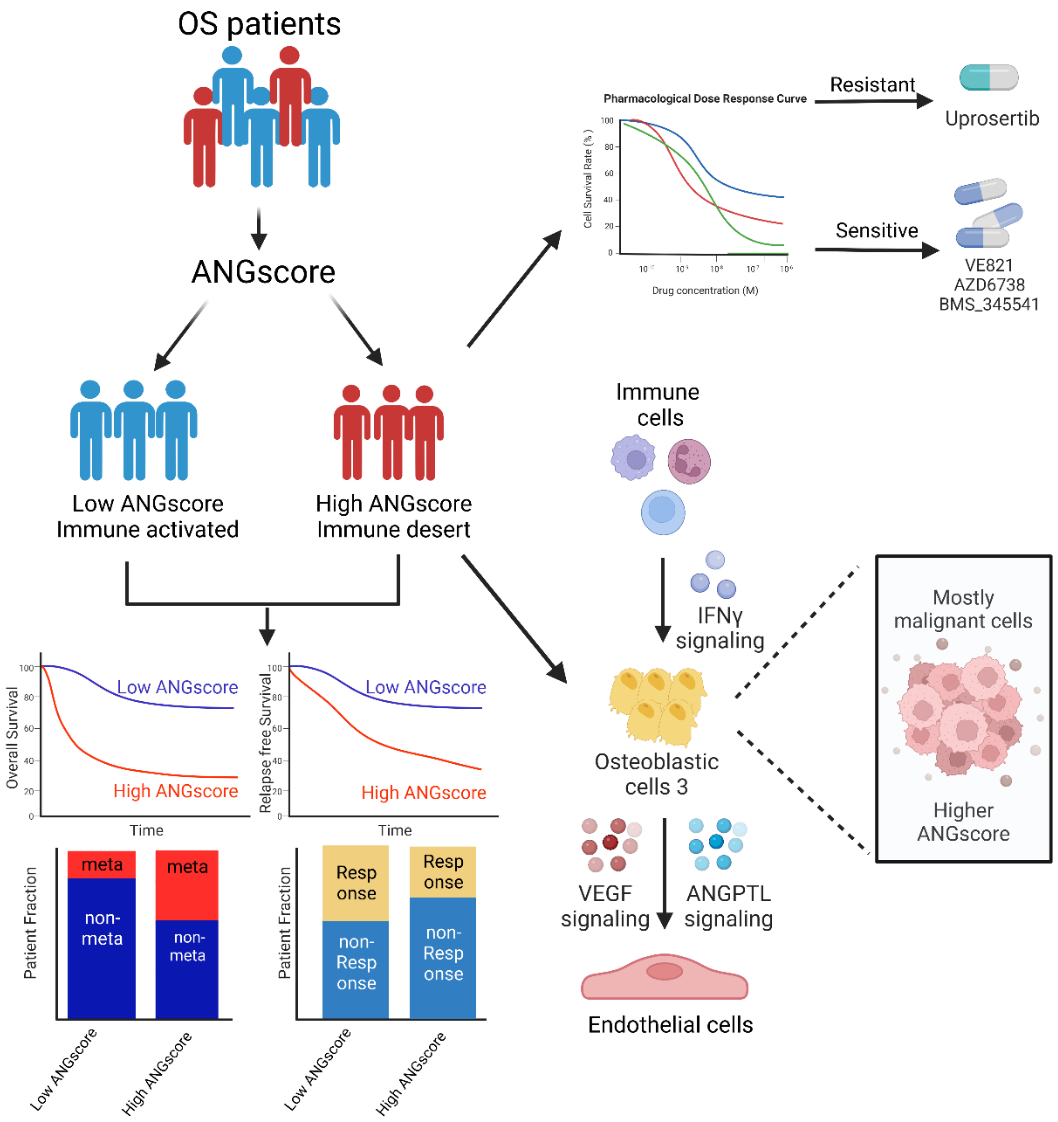

Supplementary Figure 4. The mechanism figure of the study. (Created with Biorender.com)
